# Metal-hydroxyls mediate intramolecular proton transfer in heterogeneous O–O bond formation

**DOI:** 10.1038/s41557-025-01993-8

**Published:** 2025-11-14

**Authors:** Hao Yang, Fusheng Li, Shaoqi Zhan, Yawen Liu, Tianqi Liu, Linqin Wang, Wenlong Li, Mårten S. G. Ahlquist, Sumbal Farid, Rile Ge, Junhu Wang, Marc T. M. Koper, Licheng Sun

**Affiliations:** 1 Department of Chemistry, School of Engineering Sciences in Chemistry, Biotechnology and Health, KTH Royal Institute of Technology, Stockholm, Sweden; 2 State Key Laboratory of Fine Chemicals, Frontier Science Center for Smart Materials, Dalian University of Technology, Dalian, China; 3 SINOPEC (Dalian) Research Institute of Petroleum and Petrochemicals Co., Ltd, Dalian, China; 4 Department of Chemistry-Ångström, Molecular Biomimetics, Uppsala University, Uppsala, Sweden; 5 Department of Chemistry-Ångström, Physical Chemistry, Uppsala University, Uppsala, Sweden; 6 Center of Artificial Photosynthesis for Solar Fuels and Department of Chemistry, School of Science, Westlake University, Hangzhou, China; 7 Department of Theoretical Chemistry and Biology, School of Engineering Sciences in Chemistry Biotechnology and Health, KTH Royal Institute of Technology, Stockholm, Sweden; 8 CAS Key Laboratory of Science and Technology on Applied Catalysis, Mössbauer Effect Data Center, Dalian Institute of Chemical Physics, Chinese Academy of Sciences, Dalian, China; 9 Leiden Institute of Chemistry, Leiden University, Leiden, the Netherlands

**Keywords:** Catalytic mechanisms, Heterogeneous catalysis, Electrocatalysis

## Abstract

Metal (hydro)oxides are among the most effective heterogeneous water oxidation catalysts. Elucidating the interactions between oxygen-bridged metal sites at a molecular level is essential for developing high-performing electrocatalysts. Here we demonstrate that adjacent metal-hydroxyl groups function as intramolecular proton–electron transfer relays to enhance water oxidation kinetics. We achieved this using a well-defined molecular platform with an aza-fused *π*-conjugated microporous polymer that coordinates molecular Ni or Ni–Fe sites that emulate the structure of the most active edge sites in Ni–Fe materials for studying the heterogeneous water oxidation mechanism. We combine experimental and computational results to reveal the origin of pH-dependent reaction kinetics for O–O bond formation. We find both the anions in solution and the adjacent Ni^3+^–OH site act as proton transfer relays, facilitating O–O bond formation and leading to pH-dependent water oxidation kinetics. This study provides significant insights into the critical role of electrolyte pH in water oxidation electrocatalysis and enhancement of water oxidation activity in Ni–Fe systems.

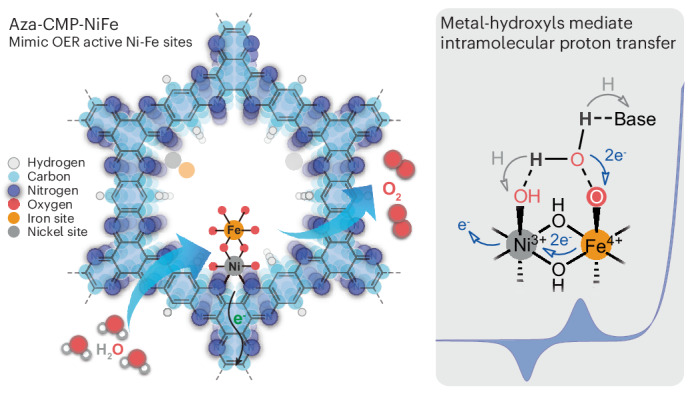

## Abstract (web summary)

Intramolecular proton relays are proposed to enhance oxygen evolution for heterogeneous (hydro)oxide electrocatalysts, but molecular-level evidence remains limited. Now it has been shown, using an aza-fused microporous polymer with Ni–Fe sites, that adjacent Ni^3+^–OH sites relay protons from Fe⁴⁺=O, accelerating the water nucleophilic attack pathway and achieving high turnover frequencies with pH-tunable kinetics.

## Main

**Fig. 1:**
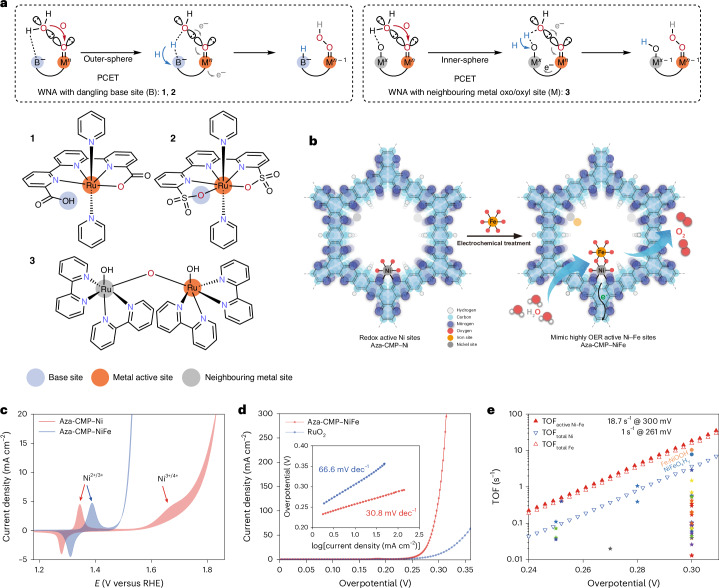
Schematic structures of the Aza-CMP–Ni and Aza-CMP–NiFe electrocatalysts and their electrochemical characterization. **a**, Representative O–O bond formation steps via the WNA pathway with proton relays and corresponding representative WOCs. **b**, Schematic diagram of molecular Ni sites in Aza-CMP–Ni and molecular Ni–Fe sites in Aza-CMP–NiFe. **c**, CV curves of Aza-CMP–Ni and Aza-CMP–NiFe in 1.0 M NaOH solution (scan rate: 50 mV s^−1^, the applied potential (*E*) is in RHE scale, without resistance compensation in solution (*iR*)). **d**, LSV curves of Aza-CMP–NiFe and reference RuO_2_ in 1.0 M KOH (scan rate: 1 mV s^−1^). Inset: corresponding Tafel plots. **e**, TOFs of Aza-CMP–NiFe based on the redox-active Ni–Fe sites, total Ni and Fe contents (1.0 M KOH) in comparison with selected state-of-the-art catalysts. The tabulated values of TOFs and overpotentials were obtained from ref. ^34^, with the complete dataset displayed in Supplementary Fig. 46. Source data

The kinetics of the oxygen evolution reaction (OER) is the catalytic bottleneck in acidic and alkaline water electrolysis. The challenging requirement of bringing the two oxygen atoms in close proximity to each other to form the O–O bond often serves as the main obstacle of the OER^1–3^. The formation of metal-oxo (M=O) species is followed either by the interaction of two metal-oxos or by the water nucleophilic attack (WNA) pathway for the crucial O–O bond formation step. The two metal-oxos interaction mechanism necessitates an optimal spatial arrangement of the bimetallic centre during the coupling process. The corresponding oxyl and/or oxo coupling mechanisms have been proposed across biological systems^4^, molecular catalysis^5,6^ and materials-based catalysis^7,8^. In the WNA pathway, the pivotal step involves the nucleophilic attack of water molecules on electron-deficient M=O, with simultaneous proton transfer to external and/or internal acceptors, resulting in hydroperoxide intermediates (M–OOH). This solution-mediated oxygen atom–proton transfer (APT) is strongly affected by the basicity of proton acceptors^9^. Rate enhancements with external proton buffers in solution facilitate water oxidation kinetics. In Mn_4_CaO_5_ systems, carboxylate side-chain-assisted deprotonation of an internal Mn–OH species is identified as the key step in forming a reactive Mn–O∙ radical^10^. Likewise, the strategic placement of intramolecular proton transfer (IPT) sites, often referred to as proton relays, near the metal centres in the secondary coordination sphere is proposed to markedly accelerate proton transfer and stabilize charged intermediates through the intramolecular APT mechanism (Fig. 1a). In homogeneous systems, molecular water oxidation catalysts (WOCs) with dangling Brønsted-base sites for enhancing OER have been explored in various reports, including carboxylate^11–15^, phosphate^16–19^, sulfonate^20,21^ and pyridine^22^. The neighbouring Ru-oxo or oxyl site in the ‘blue dimer’ has also been demonstrated to effectively shuttle the proton from the water molecule on the parallel metal-oxo site during its nucleophilic attack^23,24^. Although structurally more complicated, heterogeneous WOCs integrating proton acceptors, such as carboxylate^25^ or sulfonate^26^ groups, proximal to the catalytic centres, have been suggested to facilitate OER via a dangling site-mediated IPT process. Alternatively, neighbouring redox-active metal centres can also act as mediators for proton transfer. These centres potentially trigger a more thermodynamically and kinetically favourable pathway for inner-sphere proton-coupled electron transfer (PCET) between the relay site and reaction-active centre, a concept that has yet to be thoroughly explored and demonstrated.

Here we proposed that the metal-hydroxyl-mediated IPT can boost water oxidation kinetics on a well-defined polymeric catalyst with uniform molecular Ni–Fe sites. This experimentally demonstrates the internal proton transfer in heterogeneous dual-atom systems and elucidates the controversial synergistic interaction between Ni and Fe centres (Supplementary Discussion 1) by highlighting the potential proton relay functions of the metal-oxo, oxyl and/or hydroxyl sites in binary or multimetal material WOCs. This finding provides strong evidence for possible cooperative effects between the Ni–Fe structure on the surface of heterogeneous (oxy)hydroxide-based NiFe catalysts, where high-valent Fe^4+^ species act as active sites for WNA and adjacent OH-bridged Ni^3+^ species function as IPT relay sites that accelerate deprotonation kinetics during O–O bond formation.

## Water oxidation activities of molecular sites

The layered aza-fused *π*-conjugated microporous polymer (Aza-CMP) was prepared following a reported method in ref. ^27^. This compound contains phenanthroline-like structures that serve as coordination sites for metal ions^28^. Ni^2+^ ions were chelated into Aza-CMP by ultrasonic treatment, yielding nano-porous Aza-CMP–Ni (Fig. 1b). The complexation and single-site nature of Ni species were investigated by various spectroscopic and electrochemical techniques (Supplementary Discussion 2). The redox response of the Aza-CMP–Ni catalyst was investigated in an Fe-free 1.0 M NaOH solution^29^ on a carbon paper (CP) electrode. Two redox peaks corresponding to Ni^2+/3+^ and Ni^3+/4+^ transitions were identified for Aza-CMP–Ni/CP at 1.31 V and 1.63 V versus a reversible hydrogen electrode (RHE), respectively, before OER onset (Fig. 1c and Supplementary Fig. 18). Trace Fe species are known to influence the structure and performance of Ni-based catalysts in alkaline media^29–32^. For catalysts containing molecular Ni sites or Ni-based single-atom catalysts, operation with Fe^3+^ ions in solution could yield molecular Ni–Fe sites by bonding the Fe centre near the Ni sites during electrolysis^30,31,33^. For Aza-CMP–Ni, this transformation (Fig. 1b) was examined using various physical and electrochemical techniques (Supplementary Discussion 3), confirming the molecularly dispersed nature of Ni–Fe sites in Aza-CMP–NiFe. The formation of Ni–Fe sites enhances OER activity and lowers OER overpotential (*η*). Figure 1c shows the comparison of cyclic voltammetry (CV) curves for Aza-CMP–Ni and Aza-CMP–NiFe in a 1.0 M NaOH solution; the positive shift of the Ni^2+/3+^ redox peak demonstrates the successful integration of electron-withdrawing Fe^3+^. Simultaneously, Ni^3+/4+^ redox is suppressed by reduced OER onset, suggesting strong catalytic promotion by Fe sites.

The electrocatalytic performance of Aza-CMP–NiFe was investigated at a scan rate of 1.0 mV s^−1^ in a 1.0 M KOH solution, with an initial mass loading of 0.25 mg cm^−2^ (Aza-CMP–Ni) on a CP electrode. A RuO_2_ catalyst was loaded onto a CP substrate (RuO_2_/CP) for comparison. As shown in Fig. 1d and Supplementary Fig. 43, the onset overpotential of Aza-CMP–NiFe is 222 mV (defined as the potential at 1.0 mA cm^−2^), compared with 231 mV for RuO_2_/CP. The Tafel plot of Aza-CMP–NiFe, extracted from the polarization curve, displays a slope of 31 mV dec^−1^, whereas RuO_2_/CP exhibits a higher value of 67 mV dec^−1^. This indicates that Aza-CMP–NiFe exhibits a different rate-determining step (RDS) in the OER pathway from RuO_2_. As shown in Fig. 1e, the turnover frequencies (TOFs) were calculated from redox-active Ni–Fe sites (TOF_redox-active_) (Supplementary Fig. 45), total Fe content (TOF_total-Fe-content_) and total Ni content (TOF_total-Ni-content_). Aza-CMP–NiFe exhibits outstanding TOFs; a TOF_redox-active_ of 1.0 s^−1^ is achieved at an overpotential of 261 mV, reaching 18.7 s^−1^ at 300 mV. The TOF_total-Fe-content_ values are slightly lower than TOF_redox-active_; a TOF_total-Fe-content_ of 1.0 s^−1^ is attained at 265 mV, which further reaches 15.7 s^−1^ at 300 mV. Moreover, the TOF_total-Ni-content_ value of Aza-CMP–NiFe reaches 3.6 s^−1^ at 300 mV, which still surpasses most nickel-iron-oxy-hydroxide-based catalysts^34^. Specifically, the measured TOF_redox-active_ and TOF_total-Fe-content_ values are comparable to the TOF_surface_ of NiFeO_x_H_y_ nanoparticles (6.2 s^−1^, *η* = 300 mV)^35^ and Fe:NiOOH (10.4 s^−1^, *η* = 300 mV)^36^, where TOF_surface_ assumes only surface atoms are active^35^. The results demonstrate that the activity of Ni–Fe sites in Aza-CMP–NiFe is consistent with that of Ni–Fe oxy-hydroxides.

Postelectrolysis characterization is crucial for assessing structural stability and is a prerequisite for mechanistic investigations. Various spectroscopic techniques were used to illustrate potential structural evolution, as detailed in Supplementary Discussion 4. In brief, Fe incorporation not only enhanced catalytic activity but also improved stability and inhibited Ni site aggregation. Aza-CMP–NiFe maintained good structural stability over hours of electrolysis, whereas Aza-CMP–Ni underwent aggregation of Ni sites into clusters due to the high operating voltage required.

## Chemical and spectroscopic recognition of high-valent species

**Fig. 2:**
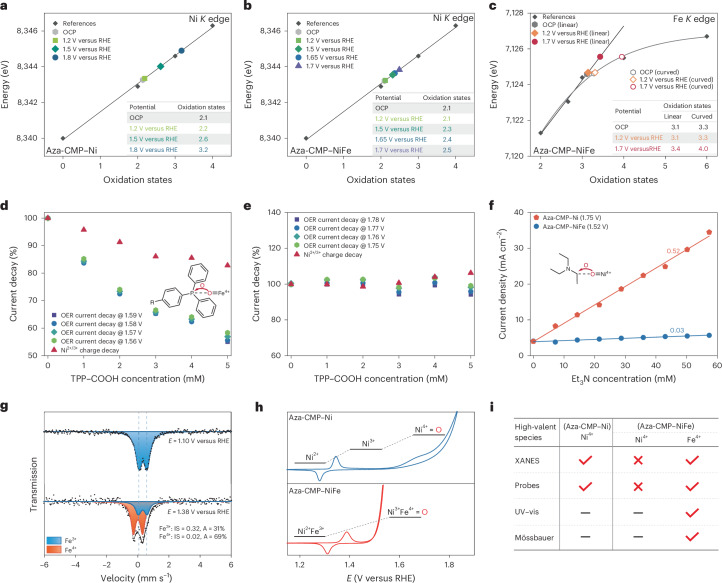
Recognition of high-valent species in the Aza-CMP–Ni and Aza-CMP–NiFe catalysts. **a**,**b**, Edge-jump energies (at 0.5 absorbance) of Ni *K*-edge XANES spectra for Aza-CMP–Ni (**a**) and Aza-CMP–NiFe (**b**) at various potentials. Tabulated values for samples and Ni references are provided in Supplementary Table 2. **c**, Edge-jump energies (at 0.6 absorbance) of Fe *K*-edge XANES spectra for Aza-CMP–NiFe at various potentials. Tabulated values for samples and Fe references are provided in Supplementary Tables 3 and 4. **d**,**e**, Change in catalytic current densities and redox peaks of Aza-CMP–NiFe/CP (**d**) and Aza-CMP–Ni/CP (**e**) electrodes with the titration of 0–5 mM TPP–COOH probe in 1.0 M NaOH, LSV data are presented in Supplementary Fig. 71. **f**, Et_3_N concentration–catalytic current density relationship of Aza-CMP–NiFe/CP and Aza-CMP–Ni/CP electrodes, with data sourced from Supplementary Fig. 110. **g**, Comparison of operando ^57^Fe Mössbauer spectra of Aza-CMP–NiFe collected before (1.10 V versus RHE) and after (1.38 V versus RHE) the Ni^2+^/Ni^3+^ redox transition, where IS stands for isomer shift (mm s^−^^1^) and A is the area fraction (%). **h**, CV curves of Aza-CMP–Ni and Aza-CMP–NiFe and corresponding metal redox states as concluded from the chemical and spectroscopic results. **i**, Summary of operando characterization results for high-valent species. Source data

Detecting intermediate species during redox reactions, which helps identify the RDS, is crucial for understanding water oxidation mechanisms and designing efficient catalysts. The valence states of metal centres were evaluated using operando X-ray absorption near edge structure (XANES) spectra, calibrated by the edge-jump energies of reference compounds (Supplementary Discussion 5.1). As shown in Fig. 2a and Supplementary Fig. 63, Ni centres in Aza-CMP–Ni show an oxidation state of +2.2 at open circuit potential (OCP) and 1.2 V versus RHE. At a potential of 1.5 V, the Ni oxidation state reaches +2.6, aligning with Ni^2+/3+^ redox peaks in the CV. Since only surface Ni sites are electrochemically active, the calculated average oxidation state underestimates the actual value. At 1.8 V, the Ni oxidation state increases to +3.2, indicating the presence of Ni^4+^ states. These findings agree with electrochemical results (Fig. 1c), suggesting stepwise oxidation from Ni^2+^ to Ni^3+^ and subsequently to Ni^4+^ before water oxidation. Similarly, Ni centres in Ni–Fe sites show oxidation state of +2.1 at OCP and 1.2 V versus RHE (Fig. 2b and Supplementary Fig. 64). Although edge energy increasing with potential, the Ni oxidation state only reaches +2.5 under 1.7 V, implying the absence of Ni^4+^ during catalysis. XANES data at the Fe *K*-edge were collected to evaluate the valence states of distal Fe sites (Supplementary Fig. 65). Assuming a linear correlation with reference compounds, Fe oxidation states are +3.1 at 1.2 V and +3.4 at 1.7 V (Fig. 2c and Supplementary Fig. 67). When using a nonlinear model with references from +2 to +6, these values increase to +3.3 and +4.0. Meanwhile, the pre-edge shift at OER potential further supports the formation of Fe^4+^ species (Supplementary Fig. 65). Overall, operando XANES confirms substantial Fe^4+^ formation and the absence of Ni^4+^ in molecular Ni–Fe systems, consistent with other double-atom Ni–Fe catalysts^33^.

High-valent Fe–O intermediates were probed using 4-(diphenylphosphino)benzoic acid (TPP–COOH), an oxygen atom transfer reagent. Increasing TPP–COOH concentration suppressed OER activity of Aza-CMP–NiFe and reduced the Ni^2+/3+^ redox peak, indicating oxygen-terminated Fe^4+^ as a key intermediate formed concurrently with Ni^3+^ when Ni^2+^Fe^3+^ state is oxidized via a two-electron redox process^37^ (Fig. 2d and Supplementary Fig. 71). In contrast, the OER activity and Ni^2+/3+^ redox remained unaffected when using Aza-CMP–Ni (Fig. 2e and Supplementary Fig. 71). These results are consistent with literature reports^37^, confirming the generation of Fe^4+^=O fragment during OER in the Ni–Fe system. In addition triethylamine (Et_3_N) was used as a probe for Ni^4+^ oxygen intermediates. Et_3_N can undergo selective oxidation by Ni^4+^ species, thereby serving as an effective probe for identifying high-valence Ni during OER catalysis (Supplementary Notes 2). Et_3_N oxidation enhanced the anodic current of Aza-CMP–Ni (reaction order 0.52) but not Aza-CMP–NiFe (reaction order 0.03) (Fig. 2f and Supplementary Fig. 110). This contrast suggests that Ni and Ni–Fe sites produce distinct intermediates during OER, with Ni^4+^ species forming only on Aza-CMP–Ni, while their absence in Aza-CMP–NiFe agrees with operando XANES results.

Operando electrochemical ultraviolet-visible light (UV–vis) spectroscopy was used to verify high-valent intermediates during voltametric cycling (Supplementary Discussion 5.2). The emergence of a new absorption band between 500 nm and 650 nm during OER suggests the formation of higher-valent states, supporting the involvement of Fe^4+^ oxo (Supplementary Fig. 69). Electrochemical operando ^57^Fe Mössbauer spectroscopy was further conducted to investigate the valence evolution of Fe centres during OER (Supplementary Discussion 5.3). Figure 2g shows a comparison of spectra collected at 1.10 V and 1.38 V versus RHE, revealing a distinct feature signal emerging after the Ni^2+/3+^ redox transition. This signal is characteristic of Fe^4+^ species, with 69% of Fe^3+^ sites further oxidized^38–40^. These results provide direct evidence that: (1) a precatalytic two-electron oxidation (Ni^2+^Fe^3+^ to Ni^3+^Fe^4+^) occurs before OER onset (1.45 V versus RHE) and (2) a substantial fraction of Fe^4+^ forms and actively participates in the catalytic cycle (Supplementary Fig. 70).

The potential-induced changes in redox states are summarized in Fig. 2i, showing consistency between spectroscopic and/or chemical probes and CV features. In Aza-CMP–Ni, Ni^3+^ forms at 1.31 V versus RHE from the Ni^2+/3+^ couple, followed by Ni^4+^ oxo species at 1.63 V versus RHE before OER (Fig. 2h). As for Aza-CMP–NiFe, with the integration of the Fe^3+^ ion, the Ni^2+^Fe^3+^ state is oxidized to Ni^3+^Fe^4+^=O via a two-electron redox process at 1.35 V versus RHE, subsequently triggering OER (Fig. 2h). Since states above Ni^3+^ are not observed, further oxidation and O–O bond formation are assigned to the Fe centre.

## Water activation redox over the molecular sites

**Fig. 3:**
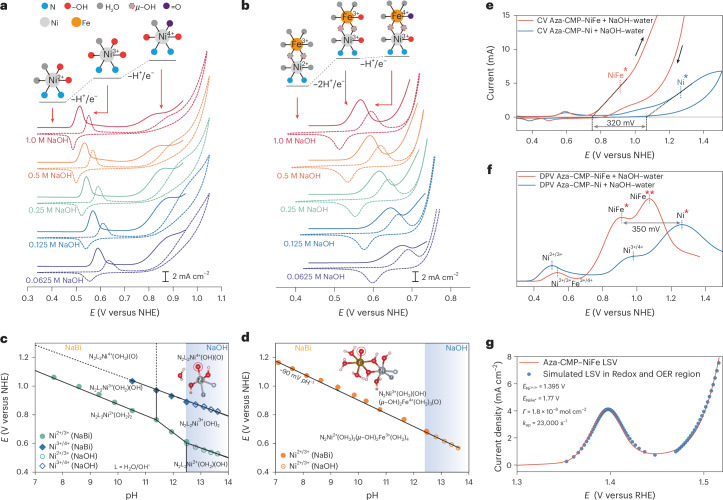
The pH-dependent redox and kinetic studies. **a**,**b**, CV (dashed line) and DPV (solid line) curves of Aza-CMP–Ni (**a**) and Aza-CMP–NiFe (**b**) in different pH conditions (unbuffered Fe-free NaOH solutions; scan rate: 50 mV s^−1^, without *iR* compensation). **c**,**d**, Pourbaix diagrams for Aza-CMP–Ni (**c**) and Aza-CMP–NiFe (**d**) for an extended pH range from pH 7.0 to pH 13.6 (pH ranges below 12.5 were operated under 0.5 M sodium borate (NaBi) buffer solutions). **e**,**f**, CV (**e**) and DPV (**f**) curves of Aza-CMP–Ni and Aza-CMP–NiFe in acetonitrile electrolytes with trace amounts of water–NaOH substrates (scan rate, 50 mV s^−1^). **g**, Simulated forward scan LSV curve in the redox of Ni^2+/3+^ and OER region for Aza-CMP–NiFe ($E_{{\rm{Ni}}^{2+/3+}}$ stands for the redox potential of Ni^2+/3+^, *E*_NiFe*_ is the potential of activated NiFe* site and *Γ* represents the surface coverage of the active species; scan rate, 50 mV s^−1^). Refer to Supplementary Note 3 for calculation details. Source data

The pH-dependent redox studies were performed to investigate water activation at molecular Ni and Ni–Fe sites, with the objective of elucidating the possible structure of reaction intermediates at different valence states. CV and differential pulse voltammetry (DPV) in Fe-free NaOH solutions, referenced at the normal hydrogen electrode (NHE) scale, confirmed proton involvement in the Ni^2+/3+^ and Ni^3+/4+^ redox transitions (Fig. 3a,b). The Pourbaix slopes of −62.5 mV pH^−1^ and −61.6 mV pH^−1^ for Ni^2+/3+^ and Ni^3+/4+^ couples in Aza-CMP–Ni indicate two successive 1H^+^/1e^−^ PCET steps from Ni^2+^ to Ni^4+^, leading to the formation of active Ni=O species (Supplementary Fig. 72). For Aza-CMP–NiFe, the Ni^2+/3+^ redox follows a 3H^+^/2e^−^ transfer process, as evidenced by the Pourbaix slope of −96.4 mV pH^−1^ and charge integration analysis of the redox wave in Supplementary Fig. 44. As established in the previous section, this redox behaviour would lead to the formation of Ni^3+^Fe^4+^ state from Ni^2+^Fe^3+^, featuring an oxygen-terminated Fe site. These studies were extended to near-neutral conditions with borate buffer (Supplementary Fig. 73). In Aza-CMP–Ni, the Ni^2+/3+^ redox between pH 7 and pH 11.5 corresponds to a 1H^+^/1e^−^ PCET from [N_2_L_2_Ni^2+^(OH_2_)_2_] to [N_2_L_2_Ni^3+^(OH_2_)(**OH**)] (where L represents an OH–H_2_O ligand, and N denotes a coordinated nitrogen atom from Aza-CMP), with p*K*_a_ values of 11.5 and 12.5 for subsequent equilibria (Fig. 3c). At pH >10, the Ni^3+/4+^ couple involves a 1H^+^/1e^−^ PCET to form OER-active [N_2_L_2_Ni^4+^(OH)(**O**)]. For Aza-CMP–NiFe, a 3H^+^/2e^−^ process that transitions [N_2_Ni^2+^(OH_2_)_2_L_2_Fe^3+^(OH_2_)_4_] to OER-active [N_2_Ni^3+^(OH_2_)(**OH**)L_2_Fe^4+^(OH_2_)_3_(**O**)] is proposed, evidenced by a slope of −90 mV pH^−1^ over an extensive pH range from 7 to 14 (Fig. 3d and Supplementary Fig. 74).

Density functional theory (DFT) calculations were used to validate the configuration of the metal centres in the different oxidation states (Supplementary Notes 6.2 and 6.4). The p*K*_a_ for the [Ni^2+^(OH)(OH_2_)_3_]^+^_T_–[Ni^2+^(OH)_2_(OH_2_)_2_]_T_ pair is 12.8, close to the experimental value of 12.5 (Fig. 3c). For the Ni^3+^ state, the [Ni^3+^(OH)_2_(OH_2_)_2_]^+^_D_–[Ni^3+^(OH)_3_(OH_2_)]_Q_ pair gives a p*K*_a_ of 11.2, consistent with the experimental value of 11.5. Further oxidation of [Ni^3+^(OH)_3_(OH_2_)]_Q_ in alkaline conditions yields a Ni^4+^ oxo species [**Ni**^**4+**^**=O**(OH)_2_(OH_2_)]_T_, poised for O–O bond formation. For molecular Ni–Fe sites, no p*K*_a_ is observed between pH 7 and pH 14 for Ni^2+^ and Ni^3+^ states. The oxidation potentials of the Ni^2+^Fe^3+^–Ni^3+^Fe^3+^ and Ni^3+^Fe^3+^–Ni^3+^Fe^4+^ couples are 0.89 eV and 0.90 eV, respectively, at a pH of 13. These approximate potentials suggest a uniform oxidation pathway from [Ni^2+^(OH_2_)_2_(*μ*–OH)_2_Fe^3+^(OH_2_)_4_]^3+^_Q_ to [**Ni**^**3+**^**(OH)**(OH_2_)(*μ*–OH)_2_**Fe**^**4+**^**=O**(OH_2_)_3_]^2+^_Q_ via an integrated 3H^+^/2e^−^ process, with the resultant Fe^4+^ oxo trigging the O–O bond formation (Fig. 3d). The agreement between calculated redox features and experimental data underscores the reliability of the computational models and approaches.

The redox properties of Aza-CMP–Ni and Aza-CMP–NiFe were further investigated in nonaqueous acetonitrile with limited substrate (H_2_O, HO^−^) to confirm successive redox states and higher-valent reactive intermediates (Supplementary Figs. 75 and 76). As shown in Fig. 3e, the Ni^2+/3+^ coupling of Ni–Fe sites shifts positively compared with that of Ni sites, consistent with aqueous behaviour. Furthermore, catalytic currents at Ni–Fe sites obviously surpass those at Ni sites. Assuming that the onset of catalytic current aligns with the emergence of Ni* and NiFe* peaks, the onset potential difference between Ni and Ni–Fe sites exceeds 320 mV. DPV curves, presented in Fig. 3f, clearly show that the oxidation peak of NiFe* lies below that of Ni^3+/4+^, indicating that Fe incorporation introduces new OER-active species with reduced energy barriers. The 350-mV potential gap between the NiFe* and Ni* peaks matches the onset potential difference, supports their assignment as the RDS-involved species for Aza-CMP–NiFe and Aza-CMP–Ni, respectively.

Quantitative CV analysis for Aza-CMP–NiFe and Aza-CMP–Ni, as discussed in Supplementary Notes 3.1, aligns with theoretical predictions based on a surface model featuring an irreversibly adsorbed monolayer. The experimental linear sweep voltammetry (LSV) for Aza-CMP–NiFe matches the simulated model (Fig. 3g). Specifically, the Ni^2+/3+^Fe^3+/4+^ redox involving two electrons can be interpreted as arising from adsorbed monolayers (loading of 1.8 × 10^−8^ mol cm^−2^) exhibiting nonideal Nernstian behaviour (Supplementary Fig. 116). At the foot of the OER wave, irreversible oxidation of Ni^3+^Fe^4+^ (that is, NiFe* generation) is presumed as the RDS; an estimated rate constant (*k*_ap_) of around 23,000 s^−1^ for simulated OER currents mirrors the LSV results, underscoring the remarkable intrinsic activity of the Ni–Fe site in alkaline water oxidation (Supplementary Fig. 118).

## Volcano-shaped pH-dependent kinetics over Ni sites in Aza-CMP–Ni

**Fig. 4:**
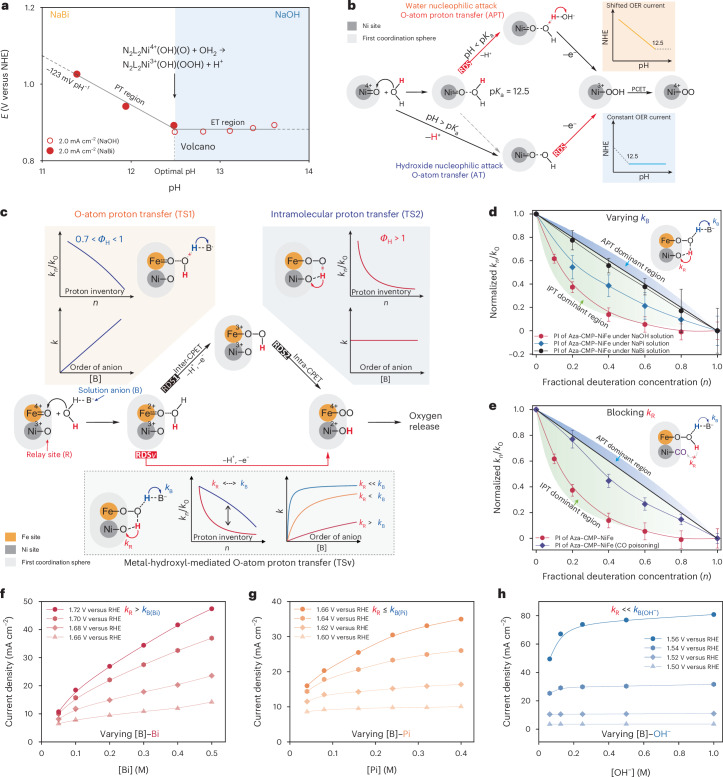
Electrochemical investigations on the formation of O–O bond. **a**, pH-dependent OER activity for Aza-CMP–Ni; a pH range below 12.5 was operated under 0.5 M NaBi buffer solutions. **b**, Schematic diagram of p*K*_a_-controlled decoupled PT–ET process with O–O bond formation and corresponding theoretical pH–activity dependency of each step. **c**, Schematic diagram of O–O bond formation on Ni–Fe sites with a virtual transition state (TS*v*) and corresponding theoretical PI and anion concentration–activity feature of each step. The deprotonation steps during O–O bond formation could be facilitated by the solution anions (RDS1) through inter-concerted proton-electron transfer (CPET) and the IPT relay sites (RDS2) through intra-CPET, with these two pathways showing distinct PI plots and anion concentration dependencies. **d**, Normalized PI plots of Aza-CMP–NiFe in NaOH, NaPi and NaBi solutions, with data sourced from Supplementary Figs. 94–96. In the plot, *n* = [D]/([D] + [H]) and *k*_*n*_ is the kinetic rate constant in a solution containing a deuterium mole fraction of *n*. Bi, borate; Pi, phosphate. **e**, Normalized PI plots of Aza-CMP–NiFe after CO poisoning in NaOH solution, with data sourced from Supplementary Fig. 98. **f**, Bi concentration–activity relationship of Aza-CMP–NiFe, with data sourced from Supplementary Fig. 100. **g**, Pi concentration–activity relationship of Aza-CMP–NiFe, with data sourced from Supplementary Fig. 100. **h**, OH^−^ concentration–activity relationship of Aza-CMP–NiFe, with data sourced from Supplementary Fig. 100. The centres of the error bars represent the average KIE values measured under different applied potentials (obtained from the LSV curves, comprising 100 data points across a 100-mV OER window). The error bars indicate the standard deviation calculated from these 100 potentials, reflecting the variability in the KIE values. Source data

The kinetic features of Ni sites in Aza-CMP–Ni, including the pH dependence, kinetic isotope effects (KIEs) and cation effects, were thoroughly examined (Supplementary Discussion 6). Comparative studies under strongly alkaline (pH >12.5) and mildly alkaline (pH <12.5) conditions reveal unique O–O bond formation behaviour. In light of these findings, Supplementary Fig. 91 catalogues the kinetic features of Aza-CMP–Ni at different pH levels. The pH-dependent behaviour of the Ni^2+/3+/4+^ redox originates from the p*K*_a_ of water-activated species. Cation effects suggest that the RDS on Ni sites follows a decoupled-APT mechanism with a water molecule initiating a nucleophilic attack on M=O. Simultaneously, the different KIEs under strong and weak alkaline conditions indicate a pH-dependent RDS. Combined with the pH–activity dependency analysis (Supplementary Note 4), volcano-shaped pH–activity relationships, peaking at the p*K*_a_, suggest a decoupled proton transfer–electron transfer (PT–ET) step in RDS (Fig. 4b). Below pH 12.5, OER kinetics are limited by deprotonation, showing a potential pH slope of −123 mV pH^−1^. Above pH 12.5, the reaction becomes pH-independent and ET-controlled, as NiOOH species are already saturated (Fig. 4a). Under such conditions, WNA essentially corresponds to hydroxide attack, identifying pH 12.5 as the p*K*_a_ of the key Ni^4+^=O–OH_2_ intermediate. This identification is supported by DFT calculations that suggest a theoretical p*K*_a_ of 13.1 for [Ni^4+^=O(OH)_2_(OH_2_)–OH_2_]_T_–[Ni^4+^=O(OH)_2_(OH_2_)–OH]_T_ transition (Supplementary Fig. 134). These results highlight that the p*K*_a_ of the O–O bond formation intermediate(s) strongly influences activity in a decoupled PCET-controlled OER, aligning with the anticipated volcano activity relationships for PCET reactions in electrocatalysis^41^. Therefore, at the same overpotential, higher pH does not always entail enhanced oxidation activity and the optimum occurs at the p*K*_a_ point of the key intermediate (M–OOH_2_). This underscores the importance of matching electrolyte pH to the catalyst’s optimal activity range when assessing performance from different systems.

## Ni-hydroxyl-mediated IPT

During the OER by Aza-CMP–NiFe, the formation of Fe^4+^=O, together with the absence of Ni^4+^ states, suggests that the O–O bond predominantly forms on the Fe site, mediated by WNA from the solvent’s hydrogen bond network. While Supplementary Discussion 6 highlights differences in redox events and RDS between Ni-only and Ni–Fe sites, as outlined in Supplementary Fig. 91, further experimental evidence is essential to fully clarify the role of Ni sites in Aza-CMP–NiFe. Electrochemical proton inventory (PI) studies were conducted to elucidate the proton transfer within the RDS of Ni–Fe system, providing insight into the contribution of each site to O–O bond formation. As detailed in Supplementary Notes 5.2 and 5.3, the fractionation factor (*Φ*), which determines PI curvature, reflects the bond strength between the host molecule and proton. Figure 4c presents the theoretical PI plots for two successive steps in the formation of the M–OO structure via WNA. With different *Φ* of protons in transit, the formation of MOOH from M=O and solvation water (designated as transition state 1, TS1, with a rate constant of *k*_B_) shows a nearly linear PI curve; while the relay site (R)-assisted intramolecular deprotonation of MOOH to MOO species (designated as transition state 2, TS2, with a rate constant of *k*_R_) exhibits increasing curvature in a bowl-shaped plot. Since the RDS of Ni–Fe system may not be confined to a single reaction step, we use the concept of a virtual transition state (TS*v*) that contains contributions from the transition states of two or more sequential steps, with the properties of the observed transition state being a weighted average of the intrinsic structural features of each microscopic transition state contributing to the overall rate^42,43^. Figure 4c also depicts the distinction between a single rate-limiting state and a virtual transition state in the linear, two-step O–O bond formation reaction; the respective curvatures of PI plots are influenced by the weighting factor (that is, *k*_B_ and *k*_R_) of the two elementary steps, TS1 and TS2.

Since the p*K*_a_ of buffer ions indicates base strength and *k*_B_ in solution-mediated APT, electrochemical PI studies were conducted under different pH conditions with various anions (that is, varying *k*_B_), to probe protonated intermediates and identify the RDS (Fig. 4d and Supplementary Figs. 94–96)^9^. In NaOH and NaOD solutions (p*K*_a_ = 15.7, high *k*_B_), the pronounced nonlinear relation between rate attenuation (*k*_*n*_/*k*_0_) and deuterium fraction (*n*) indicates that deprotonation of MOOH governs the observed RDS. This observation is logical, given that the reaction is governed by the slower TS2 when *k*_B_ is much higher than *k*_R_. By contrast, under near-neutral borate (Bi) buffer (p*K*_a_ = 9.2, low *k*_B_), the PI plot exhibits a semi-linear trend, suggesting that TS1 is the key determinant of the RDS due to the notably lower *k*_B_ compared with the intramolecular *k*_R_ that is less influenced by the solvation environment. Under a mildly alkaline phosphate (Pi) buffer (p*K*_a_ = 12.3), the PI curve falls intermediate, reflecting comparable *k*_B_ and *k*_R_, with both TS1 and TS2 contributing to observed activity.

To demonstrate the synergistic interaction and possible IPT between Ni and Fe centres, carbon monoxide (CO) was used to obstruct H_2_O and/or OH^−^ adsorption at Ni sites (Supplementary Discussion 7.3). Following a 30-minute CO purge, assumed to lead to a saturation of the Ni sites by CO, PI studies in NaOH and NaOD reveal a marked reduction in the downward curvature, transitioning towards a more linear relationship (Fig. 4e and Supplementary Fig. 98). As OH and OD on the Ni sites are displaced by CO, protons crucial to the O–O formation are hindered from transferring to neighbouring Ni sites. Consequently, CO poisoning prompts a transformation of the RDS from a virtual transition state dominated by TS2, to a typical APT step (TS1), thereby yielding a linear PI curve.

The property of proton acceptor plays an important role in the APT-controlled O–O bond formation. When APT is mediated by external proton acceptors (TS1) in solution, the catalytic rate shows a first-order dependence on anion concentration [B] (Fig. 4c). When the proton transfer occurs through a relay within the secondary coordination sphere, proton acceptor in solution would not affect the reaction rate because the proton transfer is not directly related to the mass transport within the double layer, resulting in a zero-order dependency on [B] in the step involving TS2. Given that in the TS*v* approach, TS1 and TS2 steps compete, so the reaction order concerning [B] is expected to demonstrate a complex behaviour, influenced by the ratio between *k*_B_ and *k*_R_ (Fig. 4c and Supplementary Discussion 7.4). In Bi buffer, the reaction order of borate (*ρ*_[Bi]_) ranges from 0.34 to 0.63 across the catalytic potential; the semi-linear trend observed in the current–concentration relationship highlights the predominance of TS1, which is influenced by deprotonation in solution (Fig. 4f and Supplementary Fig. 100). In Pi buffer, *ρ*_[Pi]_ for Ni–Fe sites decreases to 0.06–0.34, showcasing increased curvature in the current–concentration dependency that implies the increasing influence of TS2, due to the higher rate constant *k*_B_ through more effective Pi relays (Fig. 4g and Supplementary Fig. 100). Furthermore, with OH^−^ as a more efficient relay, ${\rho }_{[{\mathrm{OH}}^{-}]}$ approaches zero, underscoring the significance of IPT as the decisive factor for the RDS due to the markedly higher *k*_B_ compared with *k*_R_ (Fig. 4h and Supplementary Fig. 100). These variations in anion dependence and PI illustrate the competing reactions for the apparent RDS, highlighting IPT in dual-metal sites Aza-CMP–NiFe. Furthermore, the pH-dependent Tafel slope shift (63 mV dec^−1^ to 32 mV dec^−1^) suggests a transition from APT-dominant to IPT-dominant RDS with increasing pH (Supplementary Fig. 88 and Supplementary Discussion 7.5).

## Water oxidation catalytic cycle

**Fig. 5:**
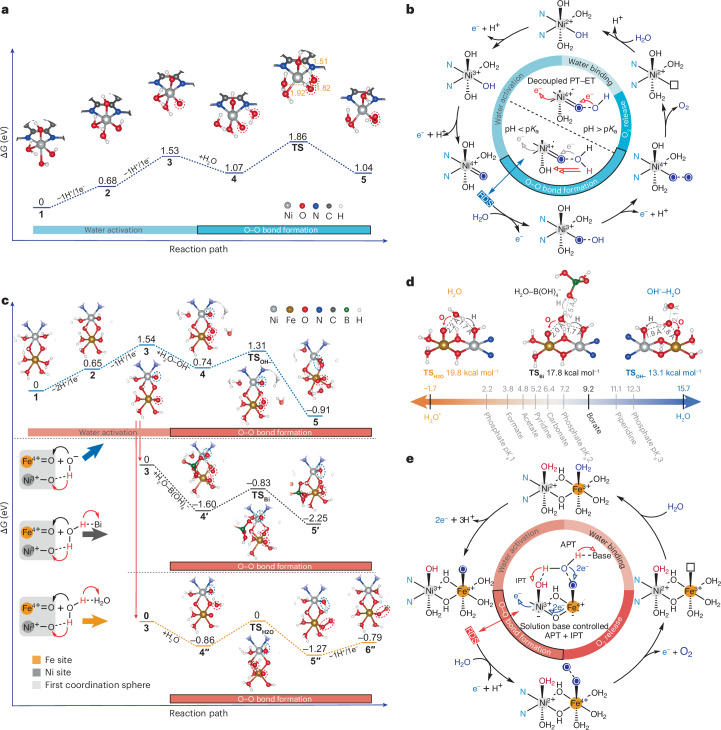
DFT calculations of the water oxidation reaction cycle. **a**, The calculated Gibbs free energy profile of the WNA pathway with optimized structures of Aza-CMP–Ni. The distances at the transition states are given in Ångstroms. **b**, Proposed catalytic cycle for Ni sites under strong alkaline conditions, with the O–O bond formation governed by a decoupled PT–ET process. **c**, The calculated Gibbs free energy profile of the nucleophilic attack pathway with optimized structures of Aza-CMP–NiFe. **d**, Comparison of transition state configurations and corresponding activation energy; the bottom axial ligands are omitted for clarity. The p*K*_a_ values of commonly used buffer components are listed at the bottom. **e**, Proposed catalytic cycle for Ni–Fe sites under strong alkaline conditions, with the O–O bond formation governed by the IPT-assisted APT mechanism.

DFT calculations were conducted to deepen understanding of the proton–electron transfer in the key steps. After the screening of the optimal configuration at different redox states (Supplementary Notes 6.2), O–O bond formation over Ni sites starts with H_2_O nucleophilically attacking [Ni^4+^=O(OH)_2_(OH_2_)]_T_ (**4**, Fig. 5a), an exergonic step with a reaction free energy and activation energy of −0.69 kcal mol^−1^ and 18.2 kcal mol^−1^, respectively. At the transition state, H_2_O molecule is stabilized through hydrogen bonds (1.51 Å) with the coordinating –OH group (**TS**, Fig. 5a). The resulting proton from WNA transfers to the –OH group, forming [Ni^2+^–OOH(OH)(OH_2_)_2_]_T_ (**5**, Fig. 5a). On the basis of experimental and computational results, a catalytic cycle incorporating four PCET processes is proposed in Fig. 5b. In alkaline conditions, deprotonation occurs during the conversion of Ni^2+^ to Ni^4+^, and two protons are detached when pH exceeds the p*K*_a_ of the Ni^2+^ complex, forming a Ni^4+^=O fragment. The O–O formation mechanism changes markedly when pH crosses the p*K*_a_ of Ni^4+^=O–OH_2_ species, reflecting the p*K*_a_-induced, decoupled PT–ET processes between pH 11 and pH 14. The reaction pathway becomes ET-controlled as Ni^4+^=O–OH reaches saturation under strongly basic conditions, resulting in a volcano-shaped pH-dependent kinetics. Since OER is triggered by Ni^4+^ states, this process necessarily requires higher operational potentials.

The catalytic pathway involving Ni–Fe sites featuring protonated bridging-oxygen atoms was modelled computationally (Fig. 5c and Supplementary Notes 6.4). O–O bond formation via nucleophilic attack on Fe=O within [Ni^3+^(OH)(OH_2_)(*μ*–OH)_2_Fe^4+^(OH_2_)_3_ = O]^2+^_Q_ (**3**, Fig. 5c) was simulated under three scenarios: OH^−^–H_2_O, H_2_O–B(OH)_4_^−^ and H_2_O alone. Specifically, TS1 with *k*_B_ in the presence of OH^−^–H_2_O was supposed to be sufficiently fast to not limit the overall reaction, simulating strong alkaline conditions with an effective proton acceptor. Here OH^−^ is stabilized by donating a hydrogen to Ni^3+^–OH and accepting a proton from solution H_2_O, lowering the activation free energy to 13.1 kcal mol^−1^ (${{\bf{TS}}}_{{{\bf{OH}}}^{-}}$, Fig. 5d). On O–O bond formation, the hydrogen from Fe–OOH readily transfers to Ni^3+^–OH through IPT (yielding a reaction free energy of −37.1 kcal mol^−1^), with the –OO group coordinating to Fe (**5**, Fig. 5c). When H_2_O molecule is used as the reactant, the model simulates strongly acidic conditions with an ineffective proton acceptor (**4′′**, Fig. 5c). The transition state is still stabilized by a hydrogen-bonding network (1.7 Å) between Ni^3+^–OH and attacking H_2_O (**TS**_**H2O**_, Fig. 5d). Lacking effective relays in solution and adjacent IPT sites, this pathway exhibits a reaction free energy of −9.5 kcal mol^−1^ and a high activation energy of 19.8 kcal mol^−1^. On forming the O–O bond, one hydrogen from H_2_O is transferred to the Ni^3+^–OH through the IPT, while another is removed by the solvent, yielding a reaction free energy of 11.1 kcal mol^−1^ (**6′′**, Fig. 5c). Furthermore, WNA facilitated by a borate anion was modelled to simulate buffered conditions (**4′**, Fig. 5c). Here H_2_O is stabilized by Ni^3+^–OH group and by hydrogen-bonding with B(OH)_4_^−^, similar to the case with OH^−^–H_2_O complex (**TS**_**Bi**_, Fig. 5d). With a proton acceptor, this pathway shows a reaction free energy of −15.0 kcal mol^−1^ and an activation energy of 17.8 kcal mol^−1^. Furthermore, hydrogen from the Fe–OOH_2_ species is efficiently transferred to the Ni^3+^–OH via IPT (**5′**, Fig. 5c). The difference in computational activation energy matches experimental activity trends (Supplementary Fig. 100), highlighting the importance of IPT sites and external proton acceptor(s) in stabilizing intermediates and promoting proton transfer.

Figure 5e proposes the complete catalytic cycle, incorporating decoupled PCET steps, for OER at molecular Ni–Fe sites. The bimetallic redox-active centres avoid high-valence Ni^4+^ formation, enabling OER to proceed efficiently at Fe sites. Crucially, during the transition from Ni^2+^Fe^3+^ to Ni^3+^Fe^4+^, Ni^3+^–OH formation promotes Fe–OOH generation via synergistic APT with IPT, where adjacent Ni^3+^–OH and solution anions act as proton transfer relays that facilitate O–O bond formation, resulting in complex anion reaction order and pH-dependent kinetics.

The previously discussed experimental and computational results establish a fundamental connection between the structure of heterogeneous catalysts with molecular single or dual-metal sites and proton transfer processes, as well as electrolyte pH. This connection, revealed through protocols such as PI, chemical probes, pH-dependent effects, solution anion or cation effects, KIEs, APT studies, quantitative CV analysis, activation energy measurements and theoretical calculations, can be adapted to various transition-metal materials for kinetics and mechanisms studies. In this work, CMP-based molecular systems enable in-depth mechanistic studies by providing clearer and more uniform active structures. The proposed Ni-hydroxyl-mediated IPT mechanism provides strong evidence for the attribution of Fe active site during OER and offers guidance for developing binary or multimetal catalysts across diverse electrochemical applications.

## Conclusions

In conclusion, molecular Ni–Fe sites anchored on Aza-CMP act as effective OER centres, with the resulting Aza-CMP–NiFe catalyst displaying higher TOFs than state-of-the-art material-based catalysts under alkaline conditions. Comparing this dual-metal catalyst with Aza-CMP–Ni that features only single Ni sites, experimental and theoretical analyses suggest that Ni–Fe sites initiate a unique metal-hydroxyl-mediated WNA pathway. The solution anions and adjacent Ni^3+^–OH group serve as proton transfer relays that facilitate O–O bond formation on Fe–O site, indicating that the reaction kinetics are governed by both interfacial deprotonation and IPT. This mechanism elucidates the pH-dependent kinetics and reveals multisite synergistic cooperation at the molecular level. Moreover, Aza-CMP–NiFe avoids the formation of high-valent Ni species, clarifying the origin of the enhanced activity in Ni–Fe system. Equally important, this study presents kinetic tools developed on a bimetallic centre model, particularly PI techniques and anion reaction order, providing strong evidence for assessing proton transfer mechanisms. In addition, it introduces triethylamine as a probe to identify Ni^4+^ species. These protocols and the proposed metal-hydroxyl-mediated IPT mechanism could be applicable to binary or multimetal catalysts, offering new opportunities for mechanistic studies in heterogeneous electrocatalysis.

## Methods

### Chemicals

All reagents and solvents were used as received from commercial sources unless otherwise noted. 1,2,4,5-benzenetetraamine tetrahydrochloride (technical grade), nickel(II) acetate tetrahydrate (≥99.995%), iron(III) chloride (≥99.9%) and anhydrous *N*,*N*-dimethylformamide (DMF, ≥99%) were purchased from Sigma-Aldrich. Hexaketocyclohexane octahydrate (≥98.0%) was purchased from TCI Chemicals. Sodium hydroxide (semiconductor grade, ≥99.99%), potassium hydroxide (semiconductor grade, ≥99.99%), lithium hydroxide (≥99.9%), deuterium oxide (D_2_O), deuterium sodium oxide solution (NaOD in D_2_O, 40 wt. %), boric acid (≥99.5%) and sodium tetraborate (anhydrous, ≥99.95%), potassium tetraborate (anhydrous, ≥99.5%), lithium tetraborate (anhydrous, ≥99.9%), potassium phosphate tribasic (≥98%), 4-(diphenylphosphino)benzoic acid (≥98.0%) and triethylamine (≥99.5%) were purchased from Sigma-Aldrich and used as received without further purification. Nickel(II) hydroxide (≥99.9%) was purchased from Alfa Aesar. High-purity water (18.2 MΩ cm^−1^) supplied by a Milli-Q system (Millipore, Advantage A10) was used in all experiments. Carbon fibre paper (Toray, TGP-H-60) substrate was purchased from Avantor; before use, the carbon fibre paper was sequentially ultrasonically cleaned in concentrated nitric acid, deionized water, ethanol and acetone for 20 min, respectively. All other reagents were commercially available and used as received. Organic solvents were of analytical reagent grade and were obtained from commercial suppliers and used without further purification.

### Synthesis of Aza-CMP

Aza-CMP was prepared according to a literature-reported route^27^. Here 532.5 mg (1.875 mmol) of 1,2,4,5-benzenetetramine tetrahydrochloride in 15 ml of anhydrous DMF and 390 mg (1.25 mmol) of hexaketocyclohexane octahydrate in 5 ml of anhydrous DMF were mixed and refluxed for 48 h under argon. The dark brown solid was purified via hot extraction with methanol for 48 h using a Soxhlet extractor. The resulting solid was dried under vacuum at 150 °C for 24 h.

### Synthesis of Aza-CMP–Ni

Aza-CMP was modified with Ni^2+^ using nickel(II) acetate. Here 20 mg of Aza-CMP was immersed in 10 ml of anhydrous DMF, and the suspension was ultrasonically dispersed and kept stirring for 30 min with argon bubbling through the solution. Then, 84 mg (1.5 eq. with respect to phenanthroline units) of nickel(II) acetate was dissolved in 10 ml of anhydrous DMF under an argon atmosphere. Ni^2+^ solution was slowly added to the suspension under the argon atmosphere, and the mixture was kept dispersed by ultrasonication for 8 h at room temperature. The solid was filtered using a polytetrafluoroethylene membrane and then washed with DMF, water, methanol and ethanol sequentially until the filtrate became colourless. Finally, the product was dried at 60 °C under vacuum conditions overnight.

### Preparation of Fe-free MOH and MOD solutions

Fe-free MOH solutions (where M = Li, Na, K) were prepared according to a previously established method^29^. In brief, 2 g of ultra-pure Ni(NO_3_)_2_·6H_2_O (99.999%) was mixed with 20 ml of 1.0 M MOH in a 50-ml polypropylene centrifuge tube to yield Ni(OH)_2_ precipitates. These were then washed three times with distilled water and 1 M MOH before being isolated via centrifugation. The resultant Ni(OH)_2_ precipitates served as iron absorbers: 40 ml of standard MOH was added and mixed with Ni(OH)_2_ in a 50-ml polypropylene centrifuge tube and stirred for 5 h to produce Fe-free MOH solutions. For experiments involving deuterium, Ni(NO_3_)_2_ was combined with 20 ml of 1 M NaOD to generate Ni(OD)_2_ solids. These were then washed three times with D_2_O and 1 M NaOD before undergoing centrifugation. The collected Ni(OD)_2_ solids were similarly used as Fe absorbers.

### Preparation of Fe-saturated NaOH solutions

Here 1 mg of FeCl_3_ was mixed with 10 ml of 1.0 M NaOH in a 50-ml propylene centrifuge tube. The yellow suspension was agitated and filtered with a 0.2-μm syringe filter. The obtained colourless filtrate was noted as an Fe-saturated NaOH solution.

### Preparation of the catalyst inks and the catalyst-loaded electrode

The ink was prepared by immersing 2.5 mg of the catalyst in a solution containing 0.2 ml of water and 0.2 ml of ethanol. The binding glues, such as strongly acidic Nafion, were avoided when attaching the catalyst powder to the substrate since they may influence the redox features of catalysts^28^. The prepared catalyst ink underwent sonication for 30 min before use. Subsequently, a volume of 10 μl of the ink was carefully dispensed onto a 5 mm × 5 mm CP electrode. The electrode bearing the deposited ink was then maintained under an argon atmosphere to facilitate drying, continuing until the film was completely dry.

### Synthesis of Aza-CMP–NiFe

Aza-CMP–NiFe was fabricated via electrochemical treatment in Fe-saturated NaOH solutions under OER conditions. Aza-CMP–Ni was first deposited onto the CP electrode by the drop-casting method. The obtained Aza-CMP–Ni/CP electrode was used as a working electrode in a standard three-electrode cell. CV of Aza-CMP–Ni/CP was conducted in Fe-saturated NaOH at a scanning window of 0.25–0.75 V versus Hg–HgO using common glassware as the electrochemical cell. The gradual formation of Ni–Fe sites is accompanied by a noticeable increase in OER current and the significant evolution of gas bubbles. After the current reached the steady state, the electrocatalyst on the CP was denoted as Aza-CMP–NiFe.

### Physical characterization methods

The morphology and composition of the fabricated films were analysed using a Hitachi field-emission scanning electron microscope (Regulus 8230), supplemented by an energy-dispersive X-ray spectroscopy detector (Oxford Ultim EXTREME). The high-angle annular dark-field scanning transmission electron microscopy images and energy-dispersive X-ray spectroscopy mapping were obtained on a JEM ARM-200F and FEI Talos F200X field-emission transmission electron microscope operating at 200 kV. The surface composition of the electrode films was measured using X-ray photoelectron spectroscopy on an ESCALAB Xi^+^ (Thermo Scientific). X-ray diffraction studies were performed on a Bruker D8 Advance Power X-Ray Diffractometer (Cu–Kα radiation, *λ* = 1.5418 Å). Raman spectroscopy was collected by a DXR Microscope (Thermo Fisher). The infrared spectra of the fabricated films were characterized by a Bruker Vertex 70 V Fourier transform–infrared (FT–IR) spectrometer by the sampling methodology of attenuated total reflectance with background correction. Solution ^1^H nuclear magnetic resonance (NMR) spectra of the compounds were recorded with a Bruker Avance DMX 500 NMR spectrometer. High-resolution solid-state NMR spectra were recorded on a Bruker Avance III spectrometer at a Larmor frequency of 125.7 MHz using a 4-mm broadband cross-polarization magic angle spinning probe head. Alanine was used as an external reference (178 ppm)^44^. The magic angle spinning sample spinning rate was 8 kHz. Cross-polarization was applied at a contact pulse duration of 1 ms and a recycle delay of 5 s. A total of 2,740 transients were needed for a good signal-to-noise ratio. The metal concentration was calculated using inductively coupled plasma optical emission spectroscopy with a Thermo Scientific iCAP 6000 series instrument. The samples on electrodes were digested in 1 ml of aqua regia with ultrasonication in a closed vessel and then diluted 10 times to make a sample solution. Metal standard solutions were measured before the samples to calibrate and obtain the standard curve. Ni concentrations were determined using three different wavelengths: 216.5 nm, 230.3 nm and 361.9 nm; Fe concentrations were determined using three different wavelengths: 238.2 nm, 273.0 nm and 371.9 nm. The average concentrations obtained at different wavelengths were taken for data evaluation. The measurements for Faraday efficiency were performed using a laboratory-made H-cell and an Omega PXM409 pressure transducer. Atomic force microscopy was conducted in dynamic mode using a Cypher ES system at room temperature under ambient air conditions. An Oxford probe (model no. HQ-150-Au), with a nominal force constant of 8 N m^−1^, was used. SiO_2_–Si served as the substrate for the atomic force microscopy experiments. Subsequent images were processed using Gwyddion software. The XANES and extended X-ray absorption fine structure measurements were carried out at beamline P64 of Deutsches Elektronen-Synchrotron (DESY). The incident beam energy was monochromatized by a Si(111) monochromator. The end-station was equipped with ionization chambers and a Lytle detector for transmission and fluorescence mode X-ray absorption spectroscopy. The data were collected in fluorescence mode using a Lytle detector, while the corresponding reference sample was collected in transmission mode. The powder samples were ground and uniformly smeared on special adhesive tape. The electrochemical operando XANES tests were carried out in a laboratory-made setup. The catalyst-loaded CP electrodes were used in a laboratory-made, three-electrode Teflon cell with a Kapton polyimide window. The HgO electrode and platinum mesh served as reference and counter electrodes, respectively. The *K*-edge energy in XANES spectra was used to determine the oxidation states of the metal centres within the sample. The edge energy is defined by the energy position corresponding to a 0.5 absorbance value in a normalized edge jump^45^. Standard samples with energy positions at 0.5 absorbance were linearly fitted to establish a calibration curve. This curve was then used to ascertain the oxidation states of the samples under analysis. The extended X-ray absorption fine structure raw data were processed according to the standard procedures with ATHENA software packages. The quantitative curve fittings were carried out in the *R*-space (1.0–3.0 Å) with a Fourier transform *k*-space range of 2.5–12 Å^−1^ by using the module ARTEMIS of IFEFFIT. The amplitude reduction factor (*S*_0_^2^) was fixed at 1.0. The energy shift (Δ*E*_0_) was constrained to be the same for all scatters. The path length *R*, coordination number (*N*) and Debye–Waller factors *σ*^2^ were left as free parameters. The wavelet transform for extended X-ray absorption fine structure was calculated by HAMA FORTRAN software. The Ni and Fe *K*-edge XANES simulations were carried out with the FDMNES packages^46^. Green’s formalism for multiple scattering was used. The energy-dependent exchange-correlation potential was calculated in the real Hedin, Lundqvist and Von Barth potential. The absorbance was calculated within a radius of 8 Å.

### Characterization of the used samples

Postcharacterization of the electrode after OER electrolysis was implemented according to the following protocols. The catalyst-loaded electrodes were aged in 1.0 M NaOH electrolyte (potential window 1.15–1.85 V versus RHE for Aza-CMP–Ni and 1.2–1.60 V versus RHE for Aza-CMP–NiFe, 50 mV s^−1^) for 200 CV scans, respectively. For X-ray photoelectron spectroscopy and Raman measurements, the used electrodes were rinsed in water and dried in ambient air before the tests. For transmission electron microscopy measurements, the sample dispersions were obtained by ultrasonic treatment of used electrodes in EtOH. The sample dispersions were directly used by following standard transmission electron microscopy characterization procedures.

### Operando UV–vis

The electrochemical operando UV–vis spectra of electrodes were measured using a laboratory-made three-electrode setup consisting of a Hg-Xenon lamp (Biologic ALX-250), a photometer (TIDAS S MSP-400) and an Autolab electrochemical workstation. The prepared catalysts, loaded on a laboratory-prepared mesoporous indium tin oxide conductive glass^47^, were used as the working electrode. A platinum wire served as the counter electrode, and a saturated Ag–AgCl electrode acted as the reference electrode. The spectrometer was synchronized with the electrochemical workstation through a connection cable.

### Operando ^57^Fe Mössbauer spectroscopy

Operando ^57^Fe Mössbauer spectroscopy measurements were performed using a Topologic Systems MFD-500AV-02 spectrometer equipped with a ^57^Co(Rh) γ-ray source. Spectral processing and fitting were conducted using the WMOSS4F program and OriginLab, with isomer shift values referenced to an *α* iron. The electrochemical operando setup was based on a previously reported custom-built ^57^Fe Mössbauer system^39^. During measurements, the Aza-CMP–Ni^57^Fe catalyst was synthesized by substituting natural FeCl_3_ with enriched ^57^FeCl_3_, and the resulting material was uniformly coated onto CP to serve as the working electrode. An Hg–HgO electrode and a platinum wire were used as reference and counter electrodes, respectively.

### Computational details

All DFT calculations for the estimation of electronic energies were carried out with the Jaguar v.8.3 program package by Schrödinger LLC^48^. Molecular geometry was optimized using Minnesota 2006 local functional (M06-L)^49^ with the LACVP** basis set^50^. The use of M06-L functional in this work has been rationalized by comparing it with several functionals, including GGA (PBE-D3, BLYP-D3), meta-GGA (M06-L), hybrid-GGA (PBE0-D3, B3LYP-D3, wB97X-D) and meta-hybrid-GGA (M06, M06-2X, wB97M-V) exchange-correlation functionals in p*K*_a_ and redox potential calculations. To identify the transition states for O–O bond formation, we searched the potential energy surface by scanning the terminal O–O bond distance. Single-point energy corrections were performed with the M06-L functional using the LACV3P**++ basis set augmented with two *f*-functions on the metal. On the basis of the gas-phase optimized geometries, the implicit solvation energies were estimated by single-point calculations using the Poisson–Boltzmann reactive field (PBF) (implemented in Jaguar) in water. The free energy of the standard hydrogen electrode of −4.44 eV was used as recommended by the International Union of Pure and Applied Chemistry. The Gibbs free energy was defined by the following equation: *G* = *E*(M06-L/LACV3P**++2f on Ru) + *G*_solv_ + ZPE + *H*_298_ – *TS*_298_ + 1.9 kcal mol^−1^ (the value 1.9 kcal mol^−1^ is a concentration correction to the free energy of solvation, which by default is calculated at 1 M_(g)_ to 1 M_(aq)_ in Jaguar).

Further details about electrochemical characterizations are available in Supplementary Information.

## Online content

Any methods, additional references, Nature Portfolio reporting summaries, source data, extended data, Supplementary Information, acknowledgements, peer review information; details of author contributions and competing interests; and statements of data and code availability are available at 10.1038/s41557-025-01993-8.

## Supplementary information

Supplementary Information Supplementary Figs. 1–143, Discussions 1–7, Notes 1–6 and Tables 1–14.

Supplementary Data 1 The atomic coordinates of key intermediates.

## Source data

Source Data Fig. 1 Source data underlying the figure.

Source Data Fig. 2 Source data underlying the figure.

Source Data Fig. 3 Source data underlying the figure.

Source Data Fig. 4 Source data underlying the figure.

## Data Availability

All the data that support the findings of this study are available within the paper and its Supplementary Information and are also available from the corresponding author upon reasonable request. Source data are provided with this paper.
