# Publisher Correction: Metal-hydroxyls mediate intramolecular proton transfer in heterogeneous O–O bond formation

**DOI:** 10.1038/s41557-025-02043-z

**Published:** 2025-12-08

**Authors:** Hao Yang, Fusheng Li, Shaoqi Zhan, Yawen Liu, Tianqi Liu, Linqin Wang, Wenlong Li, Mårten S. G. Ahlquist, Sumbal Farid, Rile Ge, Junhu Wang, Marc T. M. Koper, Licheng Sun

**Affiliations:** 1 Department of Chemistry, School of Engineering Sciences in Chemistry, Biotechnology and Health, KTH Royal Institute of Technology, Stockholm, Sweden; 2 State Key Laboratory of Fine Chemicals, Frontier Science Center for Smart Materials, Dalian University of Technology, Dalian, China; 3 SINOPEC (Dalian) Research Institute of Petroleum and Petrochemicals Co., Ltd, Dalian, China; 4 Department of Chemistry-Ångström, Molecular Biomimetics, Uppsala University, Uppsala, Sweden; 5 Department of Chemistry-Ångström, Physical Chemistry, Uppsala University, Uppsala, Sweden; 6 Center of Artificial Photosynthesis for Solar Fuels and Department of Chemistry, School of Science, Westlake University, Hangzhou, China; 7 Department of Theoretical Chemistry and Biology, School of Engineering Sciences in Chemistry Biotechnology and Health, KTH Royal Institute of Technology, Stockholm, Sweden; 8 CAS Key Laboratory of Science and Technology on Applied Catalysis, Mössbauer Effect Data Center, Dalian Institute of Chemical Physics, Chinese Academy of Sciences, Dalian, China; 9 Leiden Institute of Chemistry, Leiden University, Leiden, the Netherlands

**Keywords:** Catalytic mechanisms, Heterogeneous catalysis, Electrocatalysis

Correction to: *Nature Chemistry* 10.1038/s41557-025-01993-8, published online 14 November 2024.

In the version of this aricle initially published, there were typographical errors in Fig. 5b, e, where in the upper left-hand Ni^3+^ structure of Fig. 5b, “OH_2_” appeared originally as “OH”; in Fig. 5e, “OH_2_” in the upper-right Fe^3+^ structure appeared as “OH”; “OH_2_” in the middle-right Ni^2+^ structure appeared originally as “OH.” The figure has been amended in the HTML and PDF versions of the article.

