# Supplementary material for: Metal-hydroxyls mediate intramolecular proton transfer in heterogeneous O–O bond formation
Source: Nat Chem. 2025 Nov 14;18(2):335–44. doi: 10.1038/s41557-025-01993-8 (PMC12872446; doi:10.1038/s41557-025-01993-8)
Supplement: Supplementary file 1 — Supplementary Figs. 1–143, Discussions 1–7, Notes 1–6 and Tables 1–14. [file 41557_2025_1993_MOESM1_ESM.pdf]

nature chemistry

Supplementary information https://doi.org/10.1038/s41557-025-01993-8

Metal-hydroxyls mediate intramolecular proton transfer in heterogeneous O–O bond formation

In the format provided by the authors and unedited

Table of contents: 143 figures; 14 tables; 80 equations; 169 pages.

1. Electrochemical Methods

1.1 Electrochemical characterizations 3

1.2 KIEs experiments 3

1.3 pH-dependence electrochemistry 4

1.4 Cation dependence electrochemistry 4

1.5 Anion dependence electrochemistry 5

2. Supplementary Discussion

Supplementary Discussion 1: The controversial synergistic interaction between Ni and Fe centers 6

Supplementary Discussion 2: Construction of Ni^{2+} ion chelated interface 9

Supplementary Discussion 3: Formation of OER active molecular Ni-Fe sites 27

Supplementary Discussion 4: Post-characterizations 49

Supplementary Discussion 5: Spectral detection of high-valent species

5.1 XANES 61

5.2 UV-Vis 66

5.3 Mössbauer spectroscopy 68

Supplementary Discussion 6: Water oxidation mechanism clarification

6.1 Water oxidation kinetics under strong alkaline conditions 77

6.2 Water oxidation kinetics under weak alkaline conditions 82

Supplementary Discussion 7: Proton inventory, anion concentration-activity relationship, and Tafel slope

7.1 Overpotential calculation for proton inventory measurement 92

7.2 Proton inventory data for Aza-CMP-NiFe 93

7.3 Proton inventory measurements under CO poisoning conditions 98

7.4 Anion (base) effects for Aza-CMP-NiFe 101

7.5 Tafel slope 104

7.6 O–O *bond formation on Ni-Fe sites* 105

3. Supplementary Notes

Supplementary Notes 1: EXAFS simulation

1.1 Aza-CMP-Ni simulation 107

1.2 Aza-CMP-NiFe simulation 110

Supplementary Notes 2: Chemical probing of Ni^{4+}=O by Et_3N 115

Supplementary Notes 3: Quantitative CV analysis

3.1 $Ni^{2+/3+}$ *redox* 121

3.2 OER at the foot of the wave area 123

Supplementary Notes 4: pH dependency of OER activity

4.1 Theoretical explanation of pH-dependent OER activity 125

4.2 pH dependence of a decoupled PT-ET system 127

4.3 Pre-RDS oxidation limited pH dependence 131

Supplementary Notes 5: Shapes of proton inventory curves

5.1 Proton inventory of O–O bond formation process 134

5.2 Curves of "$MOOH_2$" step 135
5.3 Curves of "MOOH" step 136
5.4 Curves of successive deprotonation steps 140
5.5 Evaluating the potential impact of equilibrium isotope effects 142
Supplementary Notes 6: DFT calculations
6.1 Testing of DFT functionals 146
6.2 Calculations on Aza-CMP-Ni 147
6.3 Calculations on Aza-CMP-NiFe with unprotonated bridging oxygen atoms 150
6.4 Calculations on Aza-CMP-NiFe with protonated bridging oxygen atoms 152

Electrochemical Methods

1 Electrochemical Methods

1.1 Electrochemical characterizations. All the electrochemical characterizations were carried out at 25°C with a thermostatic water bath unless otherwise noted. Electrochemistry was studied in a standard three-electrode cell connected to a CHI 660e workstation, using the prepared catalysts loaded carbon paper as the working electrode, a Pt mesh as the counter electrode, and a Hg/HgO (1.0 M KOH) or Ag/AgCl (saturated KCl) as the reference electrode. The homemade cell is made of PTFE to avoid possible iron contamination from glassware. All the measured potentials were converted to the values relative to a reversible hydrogen electrode (RHE) according to the equation: Potential = $E_{\text{ref}} + E_{(\text{ref vs. RHE})}$; $E_{(\text{ref vs. RHE})}$ is the potential difference between the reference electrode and RHE at 25°C for the electrolytes, which is corrected by a commercialized RHE (HydroFlex of Gaskatel). All linear sweeping voltammetry (LSV) curves were measured with iR-correction (95%) unless otherwise mentioned. The correction was done manually according to the equation: $E_{corr} = E_{meas} - iR_u$, where E_{corr} is the iR-corrected potential, E_{meas} is an experimentally measured potential, and R_u is the equivalent series resistance extracted from the Nyquist plots.[7, 8, 9] The R_u values in our test system were in the range of ca. 3.0 Ω (1.0 M NaOH and KOH) for CP substrate.
Tafel slopes were calculated based on the polarization curves by fitting to the ***Supplementary Eqn. 1***.

$$\eta = b\log(j) + a \qquad (1),$$

where η is overpotential (mV), j is the current density (mA cm^{-2}), and b is the Tafel slope. Chronopotentiometry was recorded under the same experimental setup without iR drop compensation. The TOF of the catalyst was calculated by ***Supplementary Eqn. 2***:

$$\text{TOF} = \frac{jA}{4\text{F}\Gamma A} \qquad (2),$$

where j is the OER current density, which was obtained from LSV under a low scan rate; A is the surface area of the electrode; F is the Faraday constant (96485 C mol^{-1}), and Γ is the amount of electroactive Ni^{2+} (or Fe^{3+}) cation obtained from the linear relationship between loading and the redox peak current i_p or total metal content from the ICP-OES test.[10] Specifically, the loading of Ni^{2+} (Γ, mol cm^{-2}) has a linear relationship with the peak current i_p (the redox peak of $Ni^{2+/3+}$) given by ***Supplementary Eqn. 3***:[11]

$$i_{p(Ni^{2+/3+})} = \frac{n_e^2\text{F}^2\upsilon A\Gamma}{4\text{R}T} \qquad (3),$$

where n_e is the number of electrons (for $Ni^{2+/3+}$, $n_e = 1$), v is scan rate (V s^{-1}), A is surface area (cm^2), F is the Faraday constant, R is the ideal gas constant (8.314 J K^{-1} mol^{-1}), T is the temperature (298 K). The loading of redox-active Ni^{2+} could be estimated according to the linear relationship between the peak current of $Ni^{2+/3+}$ redox wave and the scan rates (***Supplementary Eqn. 4***).

$$\text{Slope} = \frac{i_{p(Ni^{2+/3+})}}{\upsilon} = \frac{n_e^2\text{F}^2 A\Gamma}{4\text{R}T} \qquad (4)$$

The Aza-CMP-Ni/CP electrodes were activated by cyclic voltammetry (CV) in 1 M NaOH solutions (50 mV s^{-1}, 100 scans, potential window: 1.15–1.85 V vs. RHE) before CV and differential pulse voltammetry (DPV) were performed in a non-aqueous acetonitrile solution (0.1 M $^{n}Bt_4NPF_6$). DPV measurements were carried out using 50 mV amplitude, 50 ms pulse width, 0.5 s pulse period, and 2 mV increment, with the scan directed anodically.

1.2 KIEs experiments. Kinetic isotope effects (KIEs) were assessed using electrochemical methods.

Electrochemical Methods

The LSV and CV curves were recorded with a scan rate of 5 mV s^{-1} and *iR* compensated (95%). In alkaline conditions, experiments were carried out in 1.0 M NaOH in aqueous solutions and 1.0 M NaOD in D_2O solutions. In near-neutral conditions, anhydrous sodium tetraborate (NaBi) solutions in H_2O ([Bi] = 0.2 M) and D_2O ([Bi] = 0.2 M) were employed. The pH/pD of the H_2O and D_2O borate buffers were measured to be 9.3 and 9.8, respectively. The solution was adjusted to pH/pD of 12.0 using concentrated NaOH/NaOD. The current densities at a specified overpotential, η, were denoted as j_{H2O} and j_{D2O}. $KIEs_{(H/D)}$ were then calculated based on a specific equation.

$$KIEs_{(H/D)} = \left[\frac{j_{H_2O}}{j_{D_2O}}\right]_{\eta} \qquad (5)$$

The overpotential was corrected according to our previously reported method.[12, 13] $KIEs_{(H/D)}$ as a function of fractional deuteration concentration n (n = $[D_2O]/([D_2O]+[H_2O])$), i.e., proton inventory measurement, was measured following a similar protocol, and the electrolytes were made by mixing different proportions of aqueous solutions and D_2O solutions.

1.3 pH-dependence electrochemistry. The LSV or CV curves were recorded with iR compensated (95%) in NaOH solutions with different concentrations of 0.0625 M, 0.125 M, 0.25 M, 0.5 M, and 1.0 M. The pH values of NaOH solutions are obtained using a pH-electrode pHydrunio; the measured pH values of 0.0625 M, 0.125 M, 0.25 M, 0.5 M, and 1 M NaOH solutions are 12.49, 12.81, 13.11, 13.37, and 13.61, respectively. The pH values of NaBi (0.5 M) buffered solutions were adjusted by H_3BO_3 (0.5 M) and NaOH (0.5 M) solutions; the pH values were obtained directly by a conventional pH-meter. The reaction order based on OH^- can be calculated according to ***Supplementary Eqn. 6***, where j is the current density (mA cm^{-2}) at a certain overpotential η, $[OH^-]$ is the concentration of hydroxide in electrolytes (mol L^{-1}).

$$\rho_{[OH^-]} = \left[\frac{\partial log j}{\partial(-14 + pH)}\right]_{\eta} = \left[\frac{\partial log j}{\partial pH}\right]_{\eta} \qquad (6)$$

1.4 Cation dependence electrochemistry. The presence of ions in water seriously affects the H-bond network. K^+ is more efficient in breaking the H-bond network than Na^+ and Li^+.[14] Aside from modifying the hydrogen bonding network, the cations also possess different solvation strengths ($Li^+ > Na^+ > K^+$). The kinetics associated with the nucleophilic attack of water on oxidized electrophilic species is decreased when increasing the solvation strength (***Supplementary Eqn.*** *7*).[15]

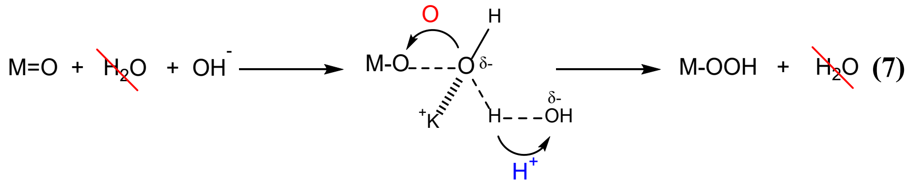

(7)

For experiments under strongly basic conditions, LSV or CV curves were obtained with 95% *iR* compensation in 1.0 M solutions of sodium, potassium, and lithium hydroxide. The pH values of these 1.0 M NaOH, KOH, and LiOH solutions were determined using a pHydrunio pH-electrode, yielding measured pH values of 13.61, 13.63, and 13.34, respectively. In near-neutral conditions, LSV or CV curves were acquired with 95% *iR* compensation in 0.5 M borate buffer solutions of sodium, potassium, and lithium. These 0.5 M NaBi, KBi, and LiBi solutions had pH values of 9.28, 9.43, and 9.10, respectively. At pH around 12, LSV or CV curves were recorded with 95% *iR* compensation in 0.5 M

borate buffer solutions of sodium, potassium, and lithium. The pH of these 0.5 M NaBi, KBi, and LiBi solutions was adjusted with 1.0 M NaOH, KOH and LiOH, respectively. All measured potentials were converted to the RHE scale for comparative analysis of current densities.

1.5 Anion dependence electrochemistry. In the water oxidation pathway that follows a concerted solution atom-proton transfer (APT) with the O-atom of a water molecule transferring to the M=O coupled with a proton transfer to the anion-based acceptor(s) in solution (***Supplementary Eqn. 8***), the current density is linearly related to the concentration of anions, as outlined in ***Supplementary Eqn. 9***.

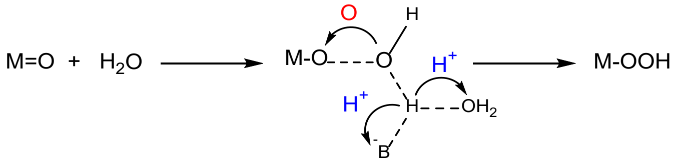

(8)

$$\left[\frac{jA}{4F\Gamma}\right]_{\eta} = k_{H_2O} + k_B[B] \quad (9)$$

Where A is the surface area (cm^2) of the electrode, F is the Faraday constant, Γ is the number of electroactive species (mol cm^{-2}), j is the current density (mA cm^{-2}), and [B] is the concentration of additional anions (mol L^{-1}). Thus, the reaction order for additional anions (external proton acceptor) in solutions, denoted as ρ[B], can be determined using the following equation:

$$\rho_{[B]} = \left[\frac{\partial \log j}{\partial \log[B]}\right]_{\eta} \quad (10)$$

In the measurements, the total concentration of buffer components increased while pH was held constant. Changes in solution ionic strength were compensated by using K_2SO_4 to reach the same cation concentration. Borate buffers at near-neutral conditions (pH = 9.5) with different concentrations were prepared by solubilizing potassium borate in water, with pH adjusted using 10 M KOH and H_2SO_4. For phosphite solutions at moderate basic conditions (pH = 12.3) with different concentrations, tri-potassium phosphate (Pi) was dissolved in water, and pH was also adjusted with 10 M KOH and H_2SO_4. Then ρ[Bi] and ρ[Pi] were calculated accordingly. Performance variations attributed to changes in hydroxyl concentration are also considered indicative of anion dependence. Solutions of NaOH/Na_2SO_4 with varying [OH$^-$] while maintaining a constant [Na$^+$] were prepared to measure the ρ[OH$^-$]. The reaction order was calculated relative to the RHE to ensure that current measurements under varying pH were compared under equivalent driving forces. It should be noted that ***Supplementary Eqn. 9*** applies only to RDS controlled by a single proton transfer process. For RDS involving multiple proton transfer processes, refer to the derivation provided in ***Supplementary Discussion 7*** for the equations expressing the reaction order relative to the concentration of the external anion.

Supplementary Discussion 1

Supplementary Discussion 1: The controversial synergistic interaction between Ni and Fe centers

Prior to the in-depth exploration of layered double hydroxides (LDHs) for water oxidation, single metal hydroxides, especially $Ni(OH)_2$, were considered catalytically active materials.[20] Yet, as research progressed, it became evident that iron impurities from the KOH electrolyte could be incorporated into the $Ni(OH)_2$ film during the electrochemical aging process, while this incorporation played a pivotal role in enhancing the water oxidation performance of $Ni(OH)_2$.[2, 21] Inspired by this finding, researchers developed a range of high-performance WOCs based on Ni-Fe LDH materials.[22, 23, 24] While the incorporation of Fe enhances the catalytic performance, the reaction mechanism and the assignment of catalytic active sites remain contentious. Recent findings suggest that Ni undergoes oxidation to the +4 state, and the active oxygen species responsible for OER are believed to be associated with Ni sites.[25, 26, 27, 28] Conversely, high valent Fe^{4+} has also been identified as active in OER, proposing catalyst cycles based on redox-active Fe sites.[29, 30, 31, 32, 33] Despite significant advancements in recent years, the catalytic mechanism of the Ni-Fe system, including water nucleophilic attack with Ni-Fe bridged *O species,[28] hydroxyl nucleophilic attack with Fe-Fe bridged *O species,[29] hydroxyl nucleophilic attack with Fe oxo species,[30, 31] coupling of Fe oxo species,[32] coupling of Ni oxo species,[33] remains a topic of significant interest. However, most of the investigations have been conducted using a material system, typically NiFe oxy-hydroxide, which is hindered by dynamic structure evolution with phase transformation,[28] Fe leaching,[34, 35] and undesirable phase segregation[36, 37]. Additionally, factors such as the metal ratio,[38, 39, 40] morphology,[41, 42] and intercalated anions[43, 44] contribute to a complex system, inevitably introducing non-uniform active structures, including edge sites, defect sites, dangling sites, surface sites, bulk sites, and separated phases (***Supplementary Fig. 1a***), complicating the interpretation of specific experimental phenomena from statistical data. These variables render molecular-level insights into the catalytic mechanism significantly challenging.

It is now well accepted that metal (hydro)oxides comprising redox-active 3*d* transition metal centers serve as the most active heterogeneous OER sites in alkaline media.[35] Even in the lattice oxygen oxidation mechanism (LOM) with redox-active oxygen centers, the high valent metal centers are required to increase the covalency of the M–O bond. Nonetheless, diverse catalytic sites within an uncertain chemical environment in a single catalyst sample introduce a multiplicity of potential catalytic pathways, even for crystalline materials. This complexity impedes the elucidation of the heterogeneous WOC mechanism. Consider, for instance, the NiFe catalytic system; within the material, highly active, low-coordinated defect and edge sites coexist with low-active, high-coordinated bulk sites.[36] Given the phase separation of FeOOH and $Ni(OH)_2$ in NiFe hydroxide, the detection of Ni^{4+} or Fe^{4+} in NiFe hydroxide does not necessarily denote their identification as highly active catalytic sites. The formation of Ni–O–Fe motifs plays a pivotal role in enhancing OER activities, which is one of the concepts widely acknowledged across numerous studies. Thus, a single Fe atom bonding to a single Ni site is anticipated to elicit similar augmentation effects, substantially simplifying the complexity of the research model. The ideal single-site and dual-site systems, devoid of phase separation in oxides, are more appropriate as a template for studying the catalytic mechanism. Notwithstanding recent publications on several atomically dispersed catalysts, including dual-atom Ni-Fe catalysts,[37, 38, 39] their overall heterogeneity, resulting from high-temperature pyrolysis, non-uniform support materials, and ambiguous active site structures/environment, limited their application in unraveling catalytic mechanisms.[40, 41] Catalytic systems capable of providing definitive coordination environments to facilitate the formation of structurally defined transition-metal active sites are

Supplementary Discussion 1

essential for trustworthy mechanistic studies of heterogeneous OER.

We recently discovered that the periodic pyridinic nitrogen sites within microporous Aza-fused π-conjugated microporous polymers (Aza-CMP) can effectively bond with cobalt sites, thereby producing the molecularly well-defined Aza-CMP-Co catalyst, providing an excellent mechanistic study platform.[3] Herein, we would like to extend the concept to double-site systems and report a facile synthesis of Aza-CMP-coordinated single Ni sites and double Ni-Fe sites as heterogeneous WOCs (***Supplementary Fig. 1b***). Aza-CMP with phenanthroline-like structural units was used as the matrix to introduce single nickel sites onto heterogeneous surfaces, yielding molecularly well-defined Aza-CMP-Ni. The double-site Aza-CMP-NiFe is derived from the single-site Aza-CMP-Ni through an in-situ electrochemical treatment, which exhibits a high turnover frequency (TOF) of 18.7 s^{-1} at a low overpotential of 300 mV and long-term operation stability under alkaline electrolytes. By mimicking the structure of highly active edge sites in Ni-Fe materials, the as-fabricated heterogeneous Aza-CMP-Ni and Aza-CMP-NiFe serve as well-defined molecular platforms for elucidating the synergistic effect between nickel and iron in the regulation of O–O bond formation. The structure and OER kinetic features of the Ni sites and Ni-Fe sites on Aza-CMP under aqueous and non-aqueous conditions were systematically investigated. To concisely highlight the novelty of our study, the principal ideas and progressions of our research are outlined in ***Supplementary Fig. 2***. This includes our demonstration of a heterogeneous O–O bond formation mechanism characterized by metal-hydroxyl-mediated intramolecular proton transfer (IPT).

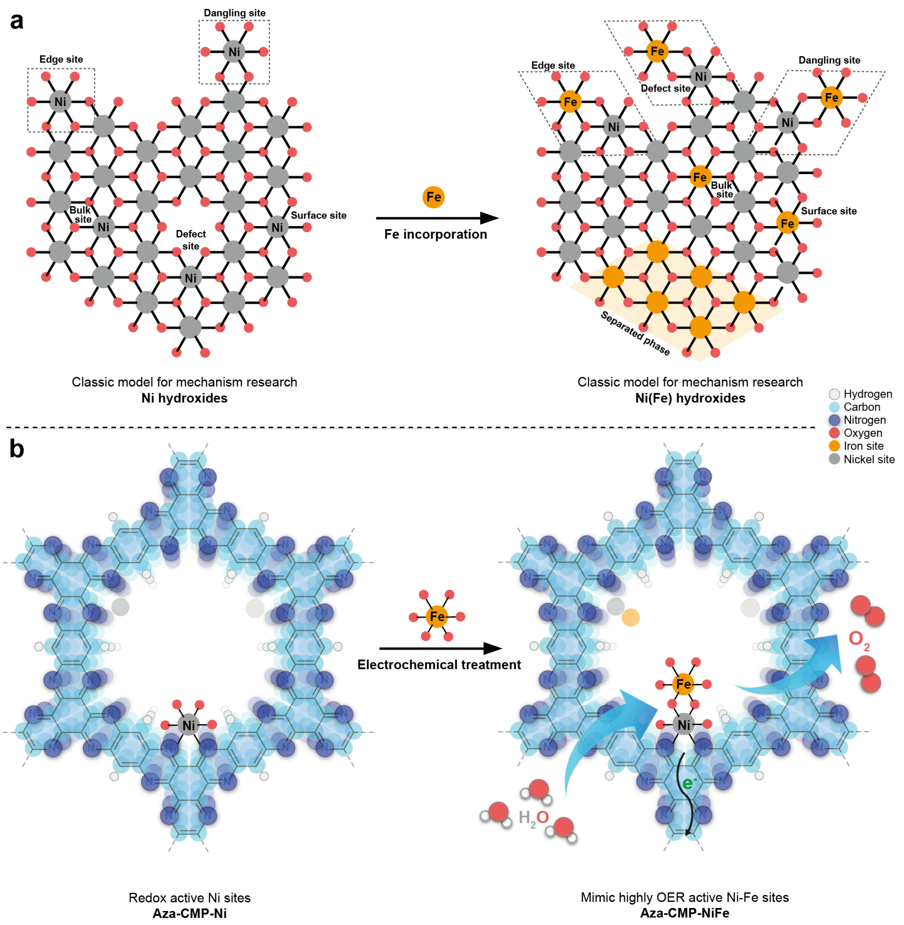

Supplementary Fig. 1 Catalysts in this work. (a) Schematic diagram of active sites in Ni and Ni(Fe) hydroxides. (b) Schematic diagram of Ni sites in Aza-CMP-Ni and Ni-Fe sites in Aza-CMP-NiFe.

Supplementary Discussion 1

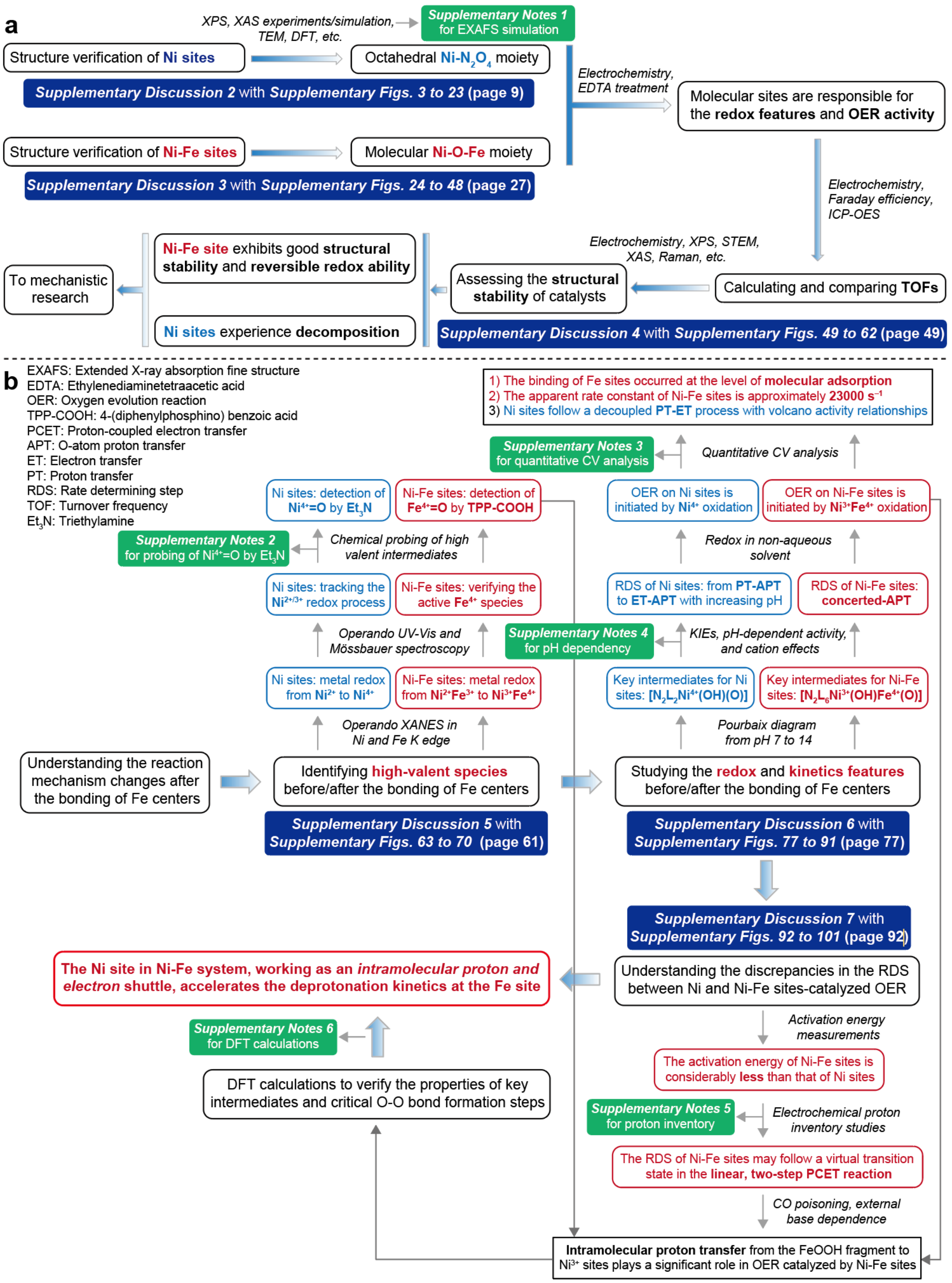

Supplementary Fig. 2 The organization of this work. (a) Experimental idea flow to verifying catalyst structure. (b) Experimental idea flow to understanding the metal-hydroxyl-mediated IPT.

Supplementary Discussion 2

Supplementary Discussion 2: Construction of Ni^{2+} ion chelated interface

Aza-CMP is a two-dimensional covalent organic framework composed of repeating Aza-fused units (***Supplementary Fig. 3***). While CMP materials are ideally expected to form single-layer structures, they typically adopt a multi-layer stacked configuration due to strong interlayer interactions, similar to graphite. As confirmed by XRD data (***Supplementary Fig. 4***), Aza-CMP exhibits a multi-layer arrangement with an interlayer spacing of 0.329 nm. The framework contains a single type of pyridinic nitrogen functionality, and its periodic microporous structure ensures high chemical stability and good conductivity. These properties are crucial for developing transition-metal-grafted heterogeneous catalysts with uniform chemical environments and minimal structural evolution. Aza-CMP was synthesized following a previously reported method.[1]

The complexation between Ni^{2+} and Aza-CMP was investigated using various spectroscopic techniques (***Supplementary Fig. 5***). The X-ray photoelectron spectroscopy (XPS) spectra of Aza-CMP-Ni in the Ni 2p region, shown in ***Supplementary Fig. 6a***, exhibit doublet peaks centered at 855.9 ($2p_{3/2}$) and 873.5 eV ($2p_{1/2}$), respectively. These peaks correspond to those observed in Ni^{2+} complexes containing nitrogen ligands rather than nickel hydroxides (855.5 eV) or oxides (853.7 eV). The O 1*s* peak centered at 531.2 was attributed to surface OH/H_2O groups, respectively (***Supplementary Fig. 6b***). Compared to nickel oxides, the absence of an O 1*s* signal from metal oxides at approximately 529.7 eV suggests that no metal oxide phase was formed on the surface. As presented in ***Supplementary Fig. 6c***, the N 1*s* peak positively shifted to 398.7 eV after the treatment with Ni^{2+}, which indicated the successful bonding between nitrogen sites and Ni^{2+} ions.[42] The similar Raman spectra obtained for Aza-CMP-Ni and Aza-CMP suggest that the complexation process did not generate any metal oxide phases (***Supplementary Fig. 7***). The Ni^{2+} content of the as-fabricated Aza-CMP-Ni was determined to be 3.27 wt% using inductively coupled plasma optical emission spectroscopy (ICP-OES), which corresponds to a nickel loading amount of 5.6 x 10^{-7} mol $mg_{Aza\text{-}CMP\text{-}Ni}^{-1}$. ***Supplementary Fig. 8*** demonstrates that the paramagnetic Ni^{2+} complexation caused a broadening of the carbon signals in solid-state ^{13}C cross-polarization/magic angle spinning nuclear magnetic resonance (CP-MAS NMR) spectra, while maintaining the identification of chemical shifts from the CMP matrix in the Aza-CMP-Ni sample. The Fourier-transform infrared spectroscopy (FT-IR) spectra of Aza-CMP-Ni exhibit C–C, C=C, and C=N vibrations, indicating the successful preservation of the CMP backbone after chelating with Ni^{2+} ions (***Supplementary Fig. 9***). The blue shift observed in ν(C=N) and ν(C–C) peaks confirmed the successful bonding between Ni^{2+} ion and the phenanthroline-like structure in Aza-CMP. Remarkably, the FT-IR spectrum of Aza-CMP-Ni is akin to that of our previously published Aza-CMP-Co, indicating a similar coordination configuration.[3]

Supplementary Fig. 10 shows the normalized X-ray absorption near-edge structure (XANES) data of the Aza-CMP-Ni catalyst and $Ni(CH_3COO)_2$ tetrahydrate. The spectra reveal that the valence states of the Ni sites in both materials are +2, as indicated by their similar absorption threshold. The similar pre-edge peak observed for Aza-CMP-Ni and $Ni(CH_3COO)_2$ tetrahydrate suggests that the Ni sites in Aza-CMP-Ni have a similar octahedral configuration.[43, 44] ***Supplementary Fig. 11*** displays the results of the extended X-ray absorption fine structure (EXAFS) analysis. The prominent peaks observed at around 1.60 Å can be attributed to the Ni–N/O scattering path; the absence of strong intensity peaks in the second-shell region implies the lack of the aggregated phase with Ni–O–Ni bonds, providing further evidence of the formation of single-site Ni ions in the Aza-CMP-Ni. The EXAFS spectrum was subjected to fitting using Ni–N and Ni–O paths via the inset density functional theory (DFT) model. The best-fitting analysis reveals that the primary peak originates from Ni–N and Ni–O first-shell coordination. The shorter path, with a distance of 2.00 Å, is ascribed to Ni–N bonds, and the

coordination numbers are constrained to 2. The longer bond, with a distance of 2.13 Å, is attributed to Ni–O bonds, where the O atom derives from the adsorbed hydroxyl or water group. The associated coordination number for the longer Ni–O path is calculated to be 3.9 (as detailed in ***Supplementary Table 1***), signifying an octahedral Ni–N_2/O_4 moiety, as illustrated in ***Supplementary Fig. 5***.

Given the numerous atoms at varying distances within the second coordination shell, accounting for all distinct paths using data fitting becomes impractical. Consequently, employing an EXAFS calculation based on the ideal structure model emerges as the optimal approach for analyzing and comparing the local structures. ***Supplementary Fig. 12a*** presents the theoretical EXAFS pattern of the Aza-CMP-Ni based on the DFT structure (see ***Supplementary Notes 1*** for the details of simulation methods). The peaks arising from the first and outer coordination spheres closely match the experimental results, emphasizing the scattering contributions from CMP ligands in the proposed coordinating configuration in the inset figure of ***Supplementary Fig. 12a***. The simulated and experimental EXAFS wavelet transforms (WTs) detect only one intensity maximum at 4.9 $Å^{-1}$, and 4.7 $Å^{-1}$, respectively, which further corroborates the reliability of the proposed molecular nature of as-fabricated Aza-CMP-Ni (***Supplementary Figs. 13a*** and ***13b***). WT analysis was also performed on a dimeric Ni–Ni model. As shown in ***Supplementary Figs. 13c***, the dimeric Ni–Ni model exhibits strong intensity at higher k-values than the single-atom Ni model and differs significantly from the experimental WT map. The establishment of Ni sites with an octahedral Ni–N_2/O_4 moiety is further substantiated by the strong correlation between the calculated and experimental XANES spectra, utilizing the FDMNES code[6] and DFT structure (***Supplementary Fig. 13b***). To further evaluate the possibility of Ni–Ni structures, EXAFS spectra were simulated for a series of hypothetical di- and multi-nuclear Ni clusters (***Supplementary Fig. 14***). In the case of a dimeric Ni–Ni model with a 2.8 Å bond length (***Supplementary Fig. 14a***), the second-shell peak intensity reached 51% of that of the first shell, with approximately 60% of the amplitude originating from the neighboring Ni atom and the remaining 40% from C and N atoms in the Aza-CMP framework. In contrast, the experimental data show that the second-shell peak is only 26% of the first-shell intensity. After accounting for the estimated contribution from C/N atoms, the signal attributable to Ni–Ni interactions is negligible. Simulated spectra of larger Ni clusters (e.g., Ni_3, Ni_4) yield even stronger second-shell peaks, deviating further from the experimental observations (***Supplementary Figs. 14b-f***), thereby excluding the presence of such multi-nuclear species.

Electron microscopy was utilized to investigate the morphology of Aza-CMP-Ni. As shown in ***Supplementary Fig. 15***, high-magnification scanning electron microscopy (SEM) images demonstrate the presence of a porous structure. Additionally, the corresponding elemental mapping images of Aza-CMP-Ni confirm the homogeneous distribution of Ni, C, N, and O elements throughout the material; the total Ni^{2+} content in Aza-CMP-Ni is measured to be 3.75 wt%, which is consistent with the results from ICP-OES. Atomic-resolution high-angle annular dark-field scanning transmission electron microscopy (HAADF-STEM) images exhibited a high density of bright dots, confirming the presence of abundant single metal sites (***Supplementary Fig. 16a***). At the thinner edge regions of the sample, most observed Ni atoms are separated by more than 0.5 nm, which effectively excludes the possibility of significant Ni–Ni bonding. Meanwhile, no spectral features attributable to bridging oxygen species (Ni–O–Ni) were observed in the XPS spectrum of Aza-CMP-Ni (***Supplementary Fig. 6b***). These observations strongly support that Ni exists in an isolated single-site form in the Aza-CMP matrix. Correspondingly, the elemental mapping images showed a uniform and clear distribution of Ni, C, and N elements, suggesting that the Ni sites in Aza-CMP-Ni are at the molecular scale rather than being aggregated oxides (***Supplementary Fig. 16b***). The morphologies of Aza-CMP-Ni were further investigated using atomic force microscopy (AFM). Evident in ***Supplementary Fig. 17***, AFM images

of catalyst films on a silicon wafer revealed a layered structure with an approximate thickness of 4 nm, which suggests that the CMP matrix retained its layered structure following Ni^{2+} complexation.

The electrocatalytic properties of the Aza-CMP-Ni catalyst were investigated in iron-free 1.0 M NaOH solution using a mass loading of 0.25 mg cm^{-2} on a CP electrode (Aza-CMP-Ni/CP). The total Ni concentration of Aza-CMP-Ni/CP is measured to be 1.38×10^{-7} mol cm^{-2}_{geo} from ICP-OES results (based on geometric area). After the activations in 1.0 M NaOH, the Ni concentration decreased to 1.27×10^{-7} mol cm^{-2} as part of the loosely stacked material was peeled off from the CP electrode. Two redox peaks corresponding to the reduction-oxidation of the $Ni^{2+/3+}$ couple at approximately 1.31 V vs. reversible hydrogen electrode (RHE) and the $Ni^{3+/4+}$ redox couple at approximately 1.63 V vs. RHE were observed for Aza-CMP-Ni/CP (***Supplementary Fig. 18a***). The different structural characteristics between nickel-hydroxide and Aza-CMP-Ni were demonstrated by their distinct redox features (***Supplementary Fig. 18b***). The OER activities of Aza-CMP-Ni/CP were investigated by linear sweep voltammetry (LSV) with a low scan rate of 1 mV s^{-1} in Fe-free 1.0 M KOH (***Supplementary Fig. 19a***). The onset overpotential (η) of the Aza-CMP-Ni/CP is 294 mV (defined as the overpotential at 0.5 mA cm^{-2}); the overpotential requirements at current densities of 10 and 40 mA cm^{-2} are 449 and 530 mV, respectively. Aza-CMP-Ni/CP exhibited a Tafel slope of 138.8 mV dec^{-1}, indicating sluggish OER kinetics. Ethylenediaminetetraacetate (EDTA) ligand in solution leads to the detachment of Ni sites from Aza-CMP-Ni. As shown in ***Supplementary Fig. 20***, Aza-CMP-Ni/CP that was treated with EDTA lost its redox features and OER activity within 10 minutes, which confirms that the molecular Ni sites in Aza-CMP-Ni are responsible for the redox features and OER activity.[45] The catalytic stability of Aza-CMP-Ni/CP was evaluated by conducting a 5-hour electrolysis at a current density of 10 mA cm^{-2} in 1.0 M KOH solution (***Supplementary Fig. 21***). The overpotential requirements of the electrode remained almost constant at approximately 490 mV during the test, indicating good catalytic stability under the given operating conditions. The amount of oxygen produced by Aza-CMP-Ni/CP during water oxidation was determined using a pressure transducer; yields of 94.5% and 97.6% are obtained at current densities of 5 and 10 mA cm^{-2} in 1.0 M KOH, respectively, indicating that the accumulated charge is nearly quantitatively consumed in OER (***Supplementary Fig. 22***). Integrating the charge of CV redox peaks is a valid and effective approach for calculating the loading of redox-active species. When assuming that the oxidation of a single Ni site involves one electron transfer (as it is highly uncommon for a single metal center to transfer multiple electrons during a single oxidation event), the amount of redox-active Ni^{2+} sites is calculated to be 2.25 to 1.9×10^{-8} mol cm^{-2}_{geo} at different scan rates, which is consistent with the range observed for molecules immobilized on the surface of porous substrates (***Supplementary Fig. 23***).[46] The turnover frequencies (TOFs) are calculated based on the redox-active and total-content Ni^{2+} sites (***Supplementary Fig. 19b***). The logarithm of the TOFs displayed a linear correlation with the overpotentials ranging from 390 to 500 mV. A $TOF_{redox\text{-}active}$ of 1.0 s^{-1} was observed at an overpotential of 439 mV, and it further increased to 2.72 s^{-1} at an overpotential of 500 mV. The OER performance of Aza-CMP-Ni is comparable to nickel hydroxide-based mono-metal electrocatalysts under alkaline conditions, although its performance lags significantly behind other bimetallic catalysts.[2, 36]

Supplementary Discussion 2

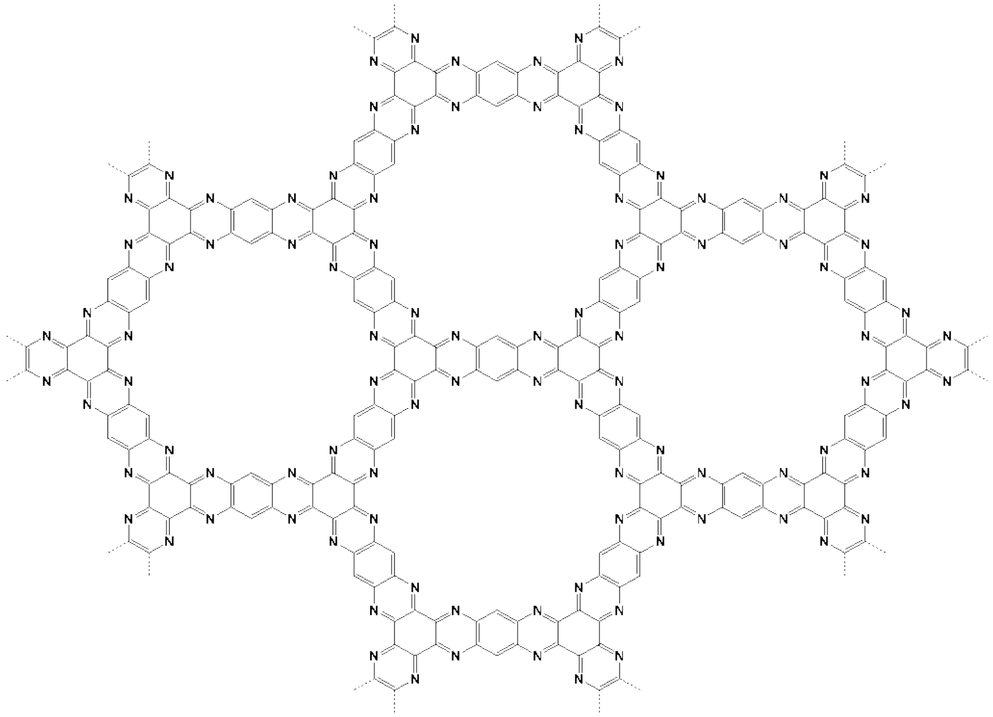

Supplementary Fig. 3 The chemical structure of Aza-CMP.

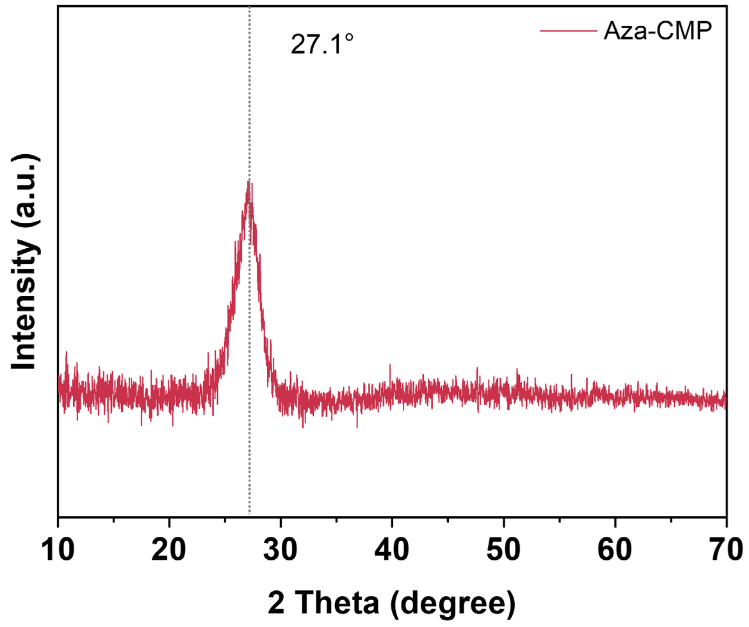

Supplementary Fig. 4 Powder XRD pattern of as-fabricated Aza-CMP.

Supplementary Discussion 2

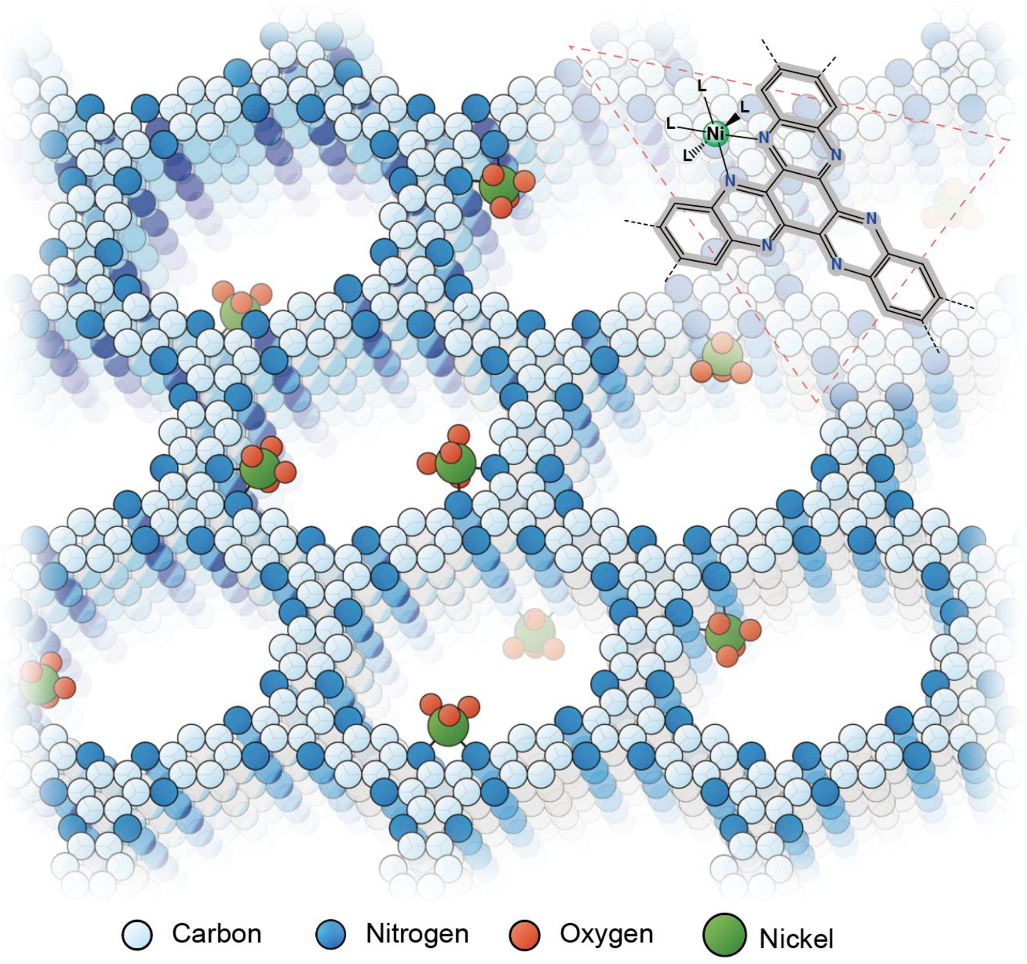

Supplementary Fig. 5 Schematic structure of the Aza-CMP-Ni catalyst. L represents coordinated H_2O/OH^- ligands.

Supplementary Discussion 2

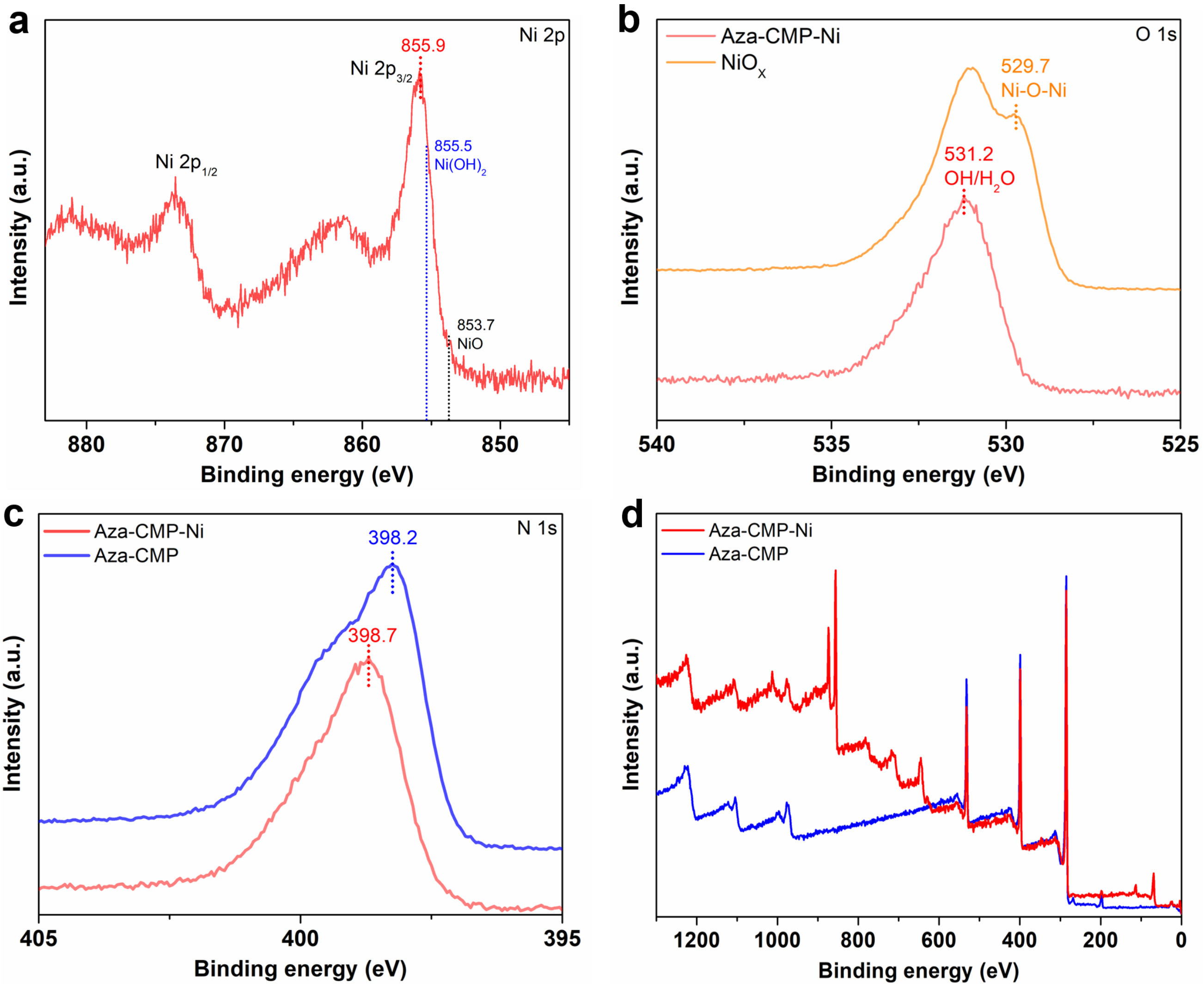

Supplementary Fig. 6 XPS spectra of Aza-CMP and Aza-CMP-Ni. (a) Ni 2*p*, (b) O 1*s*, (c) N 1*s*, and (d) survey regions.

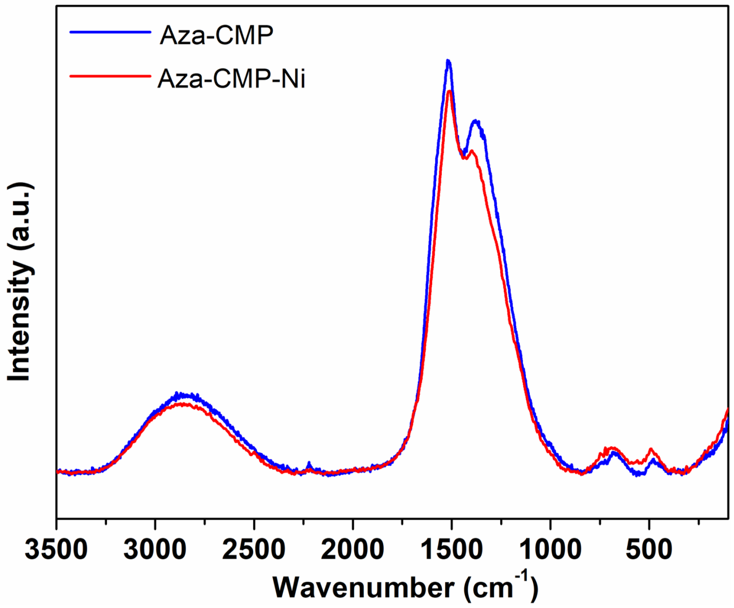

Supplementary Fig. 7 Raman spectra of Aza-CMP and Aza-CMP-Ni.

Supplementary Discussion 2

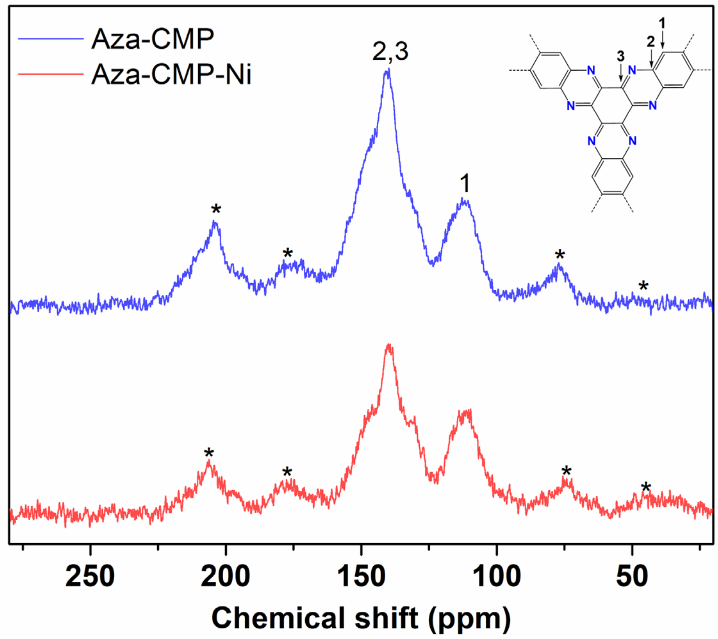

Supplementary Fig. 8 Solid-State ^{13}C CP-MAS NMR spectra of Aza-CMP and Aza-CMP-Ni. Three signals correspond to the three different carbon atoms observed in the polymer. (* = sidebands of main peaks). The loading of paramagnetic Ni^{2+} causes broadening and weakening of the NMR peaks; however, the chemical shifts from the CMP matrix remain identifiable in the Aza-CMP-Ni sample. This observation indicates that the CMP matrix structure is preserved after metalation.

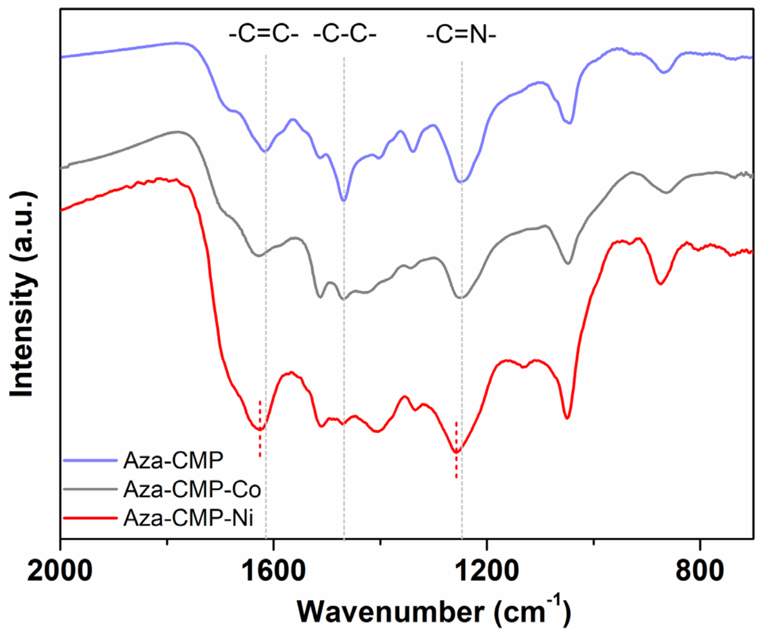

Supplementary Fig. 9 FT-IR spectra of Aza-CMP, Aza-CMP-Ni, and Aza-CMP-Co[3], recorded in ATR mode using powder samples.

Supplementary Discussion 2

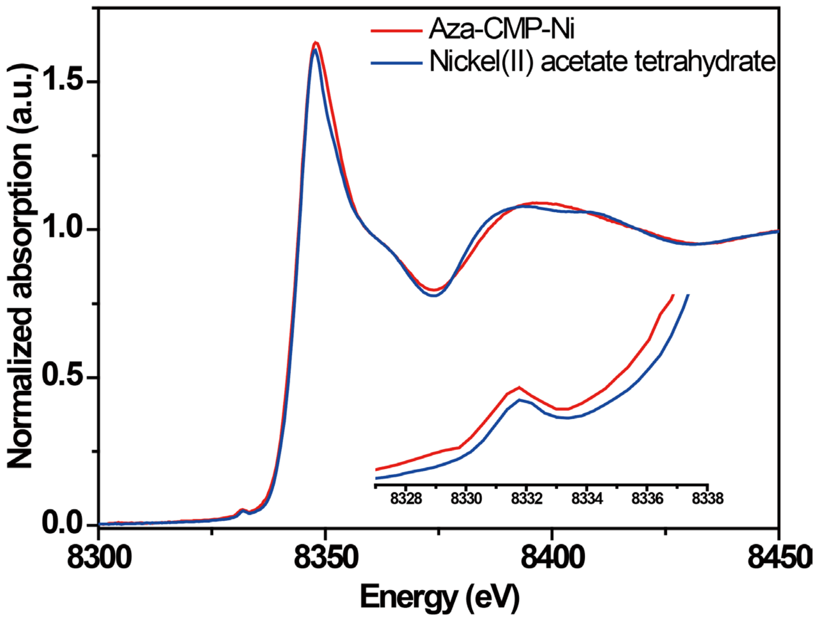

Supplementary Fig. 10 Normalized Ni K-edge XANES spectra of Aza-CMP-Ni and referenced material. The inset figure highlights the pre-edge region.

Supplementary Discussion 2

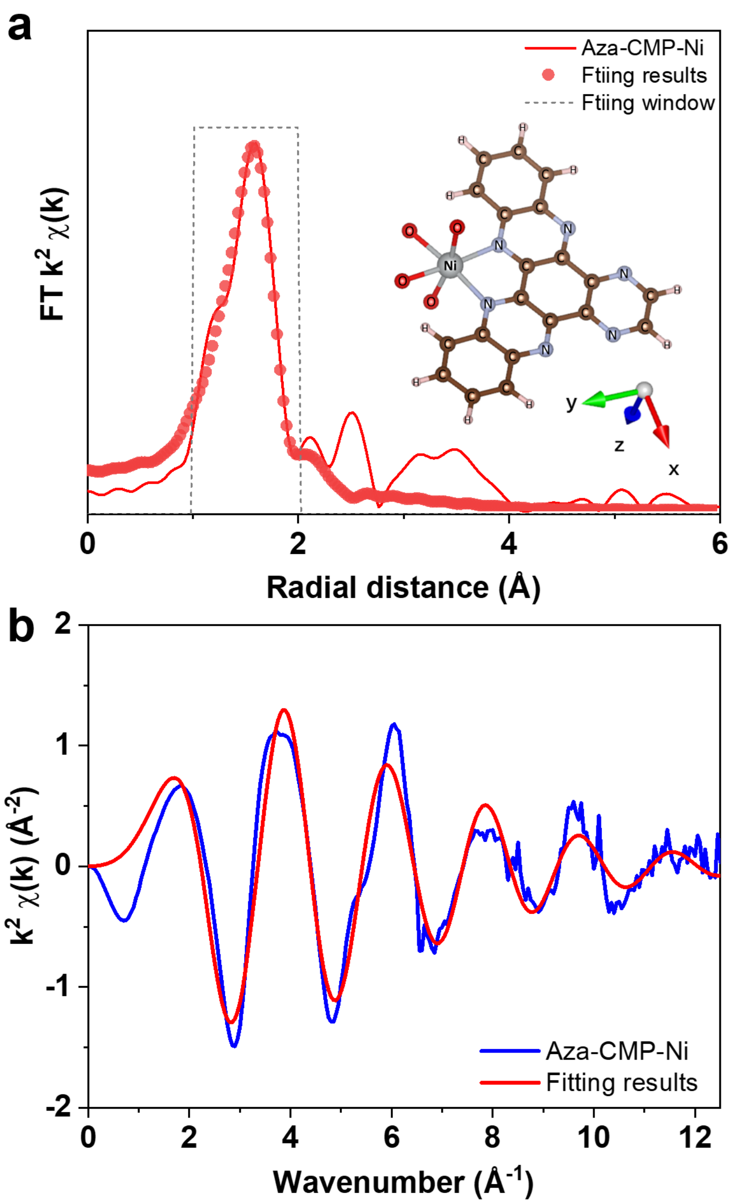

Supplementary Fig. 11 Ni K-edge EXAFS fitting of Aza-CMP-Ni. (a) Experimental and first-shell fitted k^2-weighted Ni K-edge EXAFS curves of Aza-CMP-Ni. Inset: the DFT model of the Aza-CMP-Ni for FEFF calculation. (b) Measured and first-shell fitted Fourier-transformed magnitude of Ni K-edge EXAFS spectra in k-space. The fitting parameters are presented in ***Supplementary Table 1***.

Supplementary Table 1 First shell fitting parameters of Ni K-edge EXAFS curve. [a]

Aza-CMP-Ni

Path	d [b] (Å)	N	R (Å)	σ^2 (Å^2)	R-factor
Ni–N	2.00	2.00 [c]	2.00 ± 0.03	0.0040 ± 0.002	0.0044
Ni–O	2.13	**3.89** ± 0.6	2.10 ± 0.02	0.0017 ± 0.001	

[a] N, coordination number; R, the distance between absorber and backscatter atoms; σ^2 Debye-Waller factor to account for thermal and structural disorders; R-factor, indicates the goodness of the fit. S_0^2 was fixed to 1.0. Data range: $2 \leqslant k \leqslant 12$ Å^{-1}, $1 \leqslant R \leqslant 2$ Å. [b] The distances for Ni–N and Ni–O are from the calculated DFT model of Aza-CMP-Ni. [c] These coordination numbers were constrained as *N(Ni–N)* = 2 based on the DFT model of Aza-CMP-Ni.

Supplementary Discussion 2

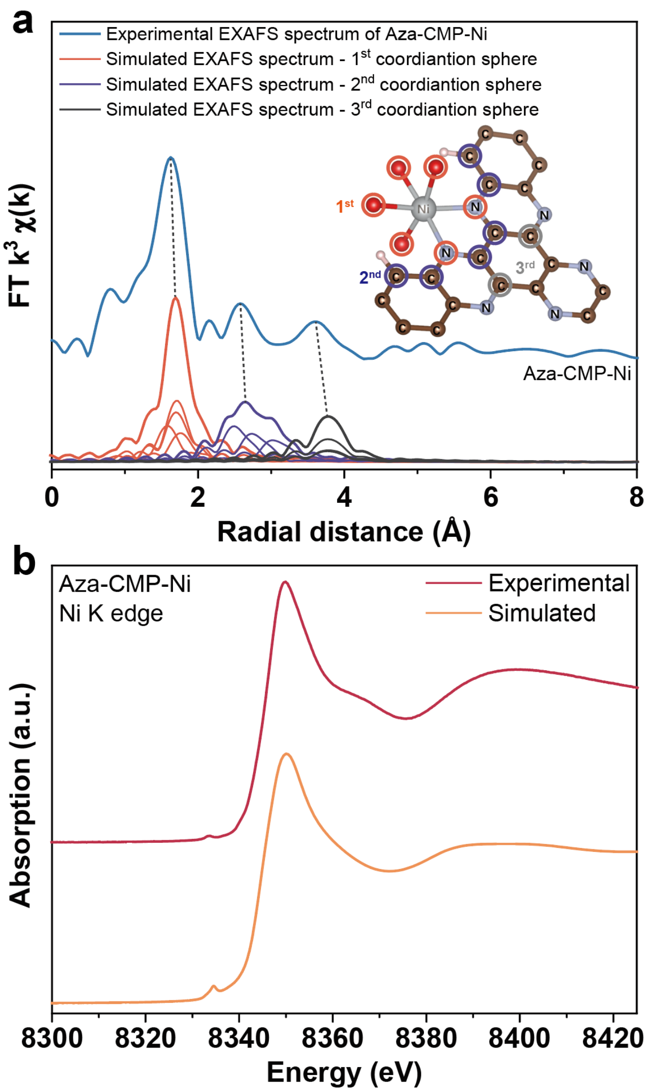

Supplementary Fig. 12 Calculated XAS spectra. (a) Comparison between the simulated and experimental EXAFS curves in R space. The inset model indicates contributions from carbon atoms in the second shell (2-3 Å). (b) Comparison between the simulated and experimental Ni K-edge XANES spectra of Aza-CMP-Ni.

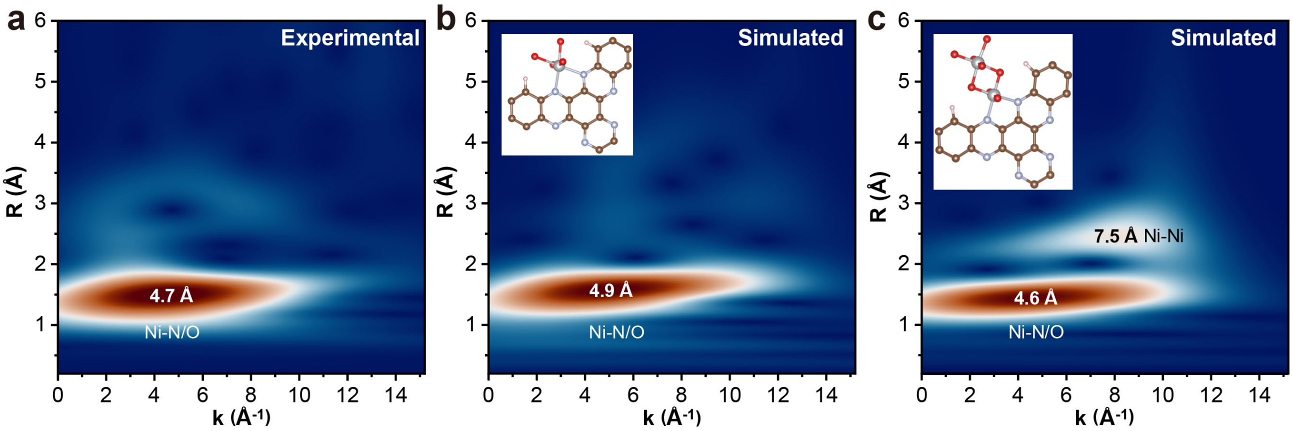

Supplementary Fig. 13 Calculated Ni K-edge EXAFS wavelet transform. (a) Experimental Aza-CMP-Ni sample, (b) simulated single-site Ni model, and (c) simulated dual-site Ni model (see ***Supplementary Notes 1*** for the details of the corresponding simulation method).

Supplementary Discussion 2

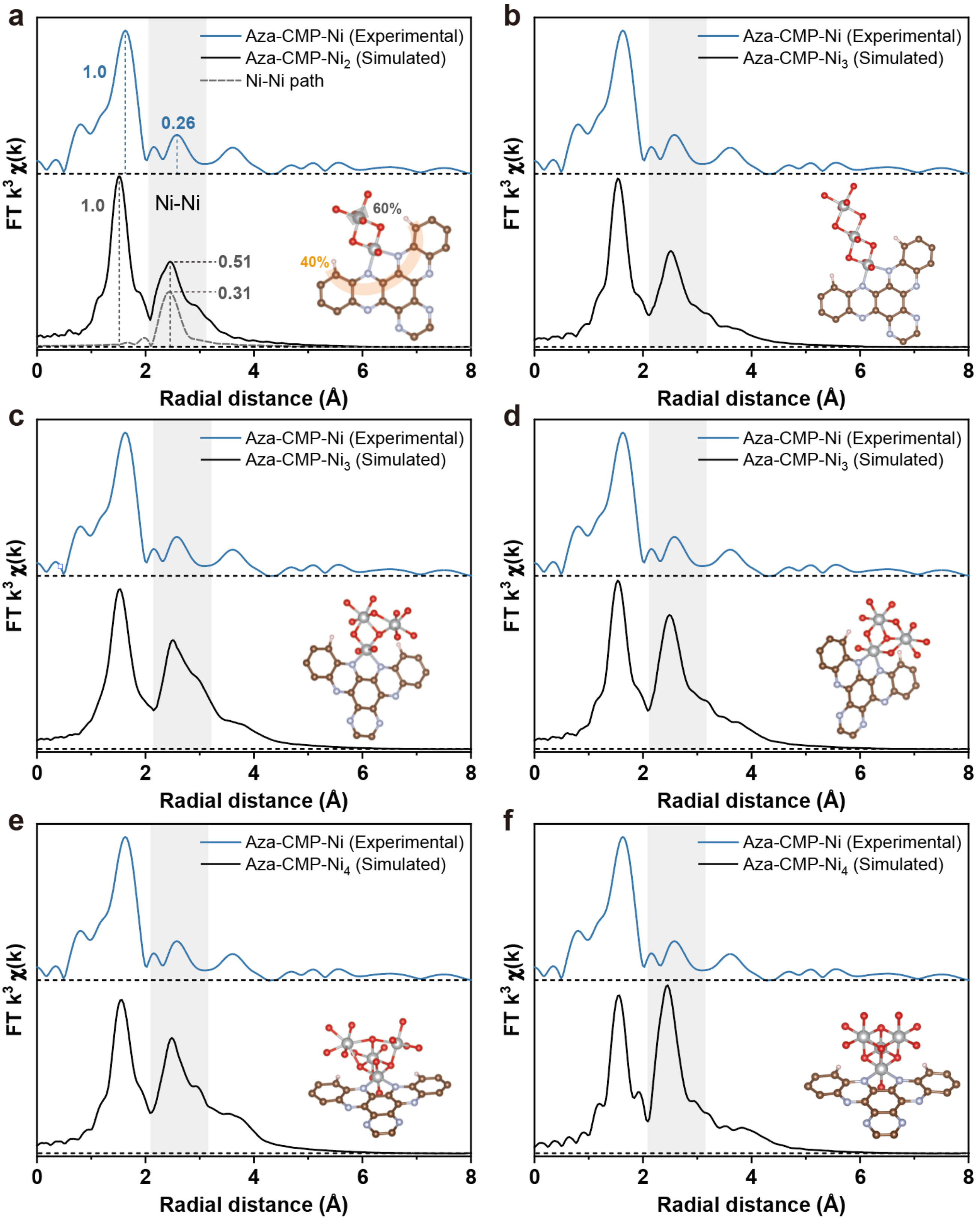

Supplementary Fig. 14 Calculated EXAFS spectra in R space for different double/triple/quadruple atom models. Inset: molecular structures for simulation.

Supplementary Discussion 2

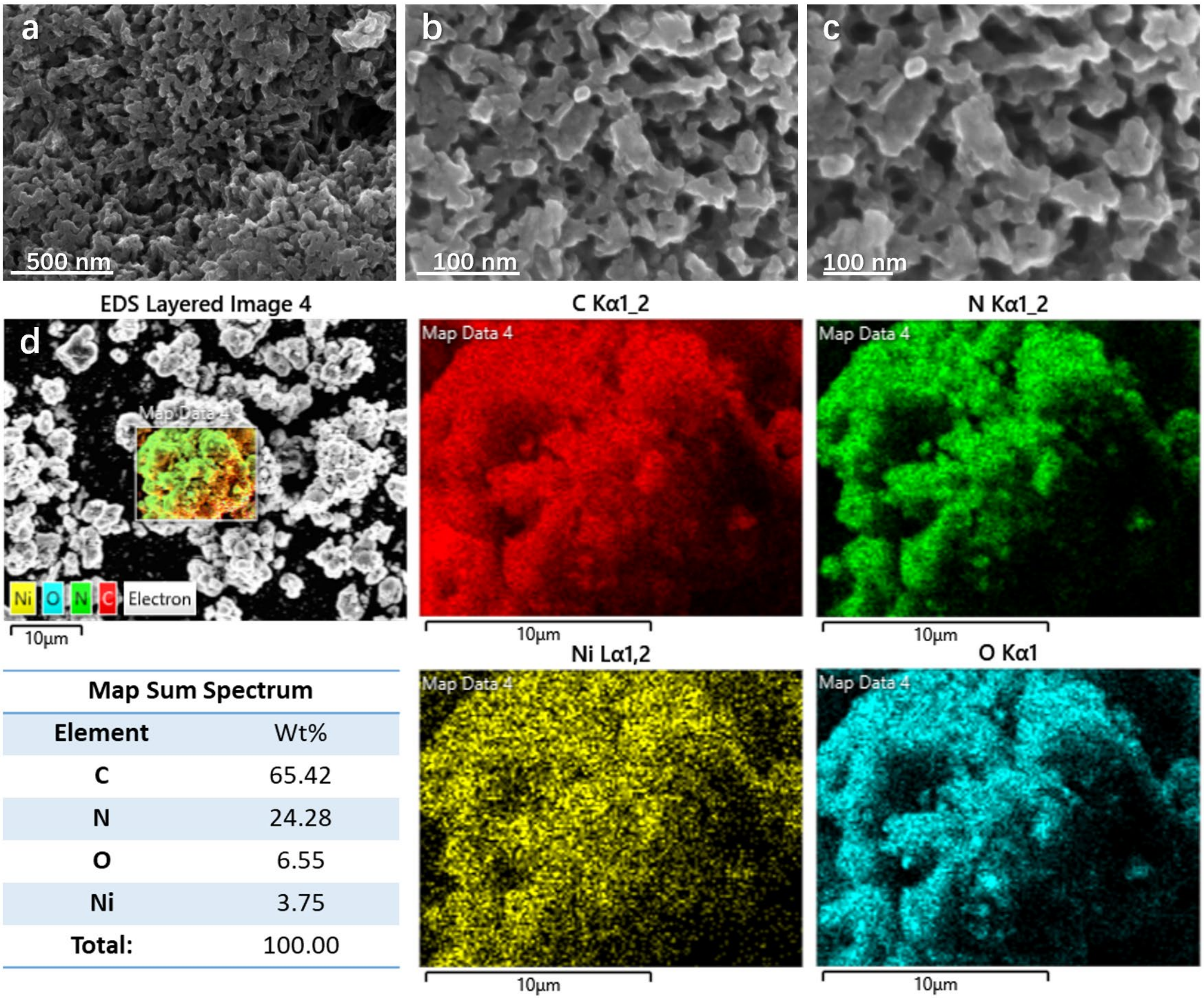

Map Sum Spectrum	
**Element**	Wt%
**C**	65.42
**N**	24.28
**O**	6.55
**Ni**	3.75
**Total:**	100.00

Supplementary Fig. 15 SEM characterization. (a-c) SEM images of Aza-CMP-Ni. (d) EDS mapping results of Aza-CMP-Ni.

Supplementary Discussion 2

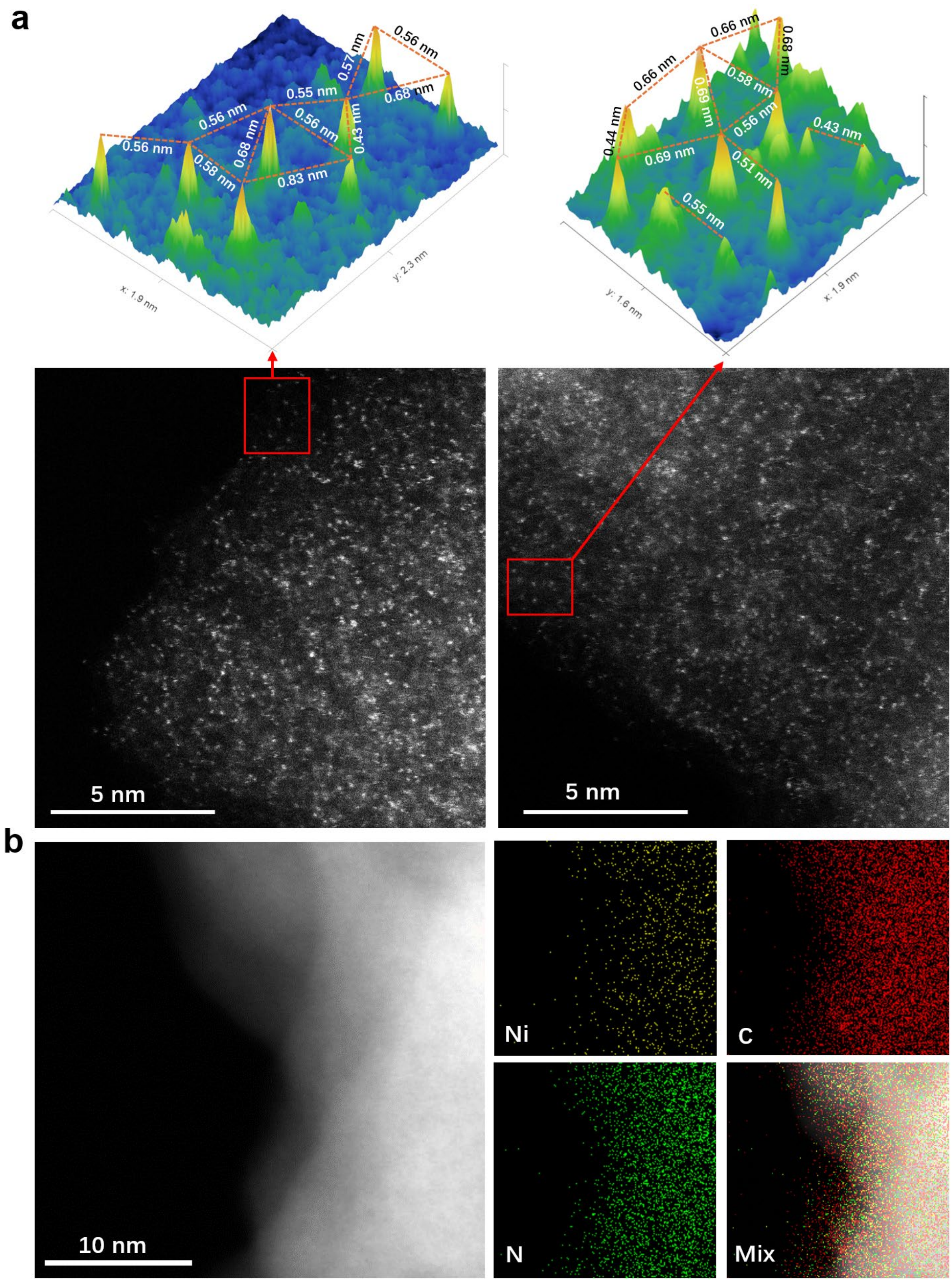

Supplementary Fig. 16 STEM characterization. (a) Atomic-resolution STEM images of Aza-CMP-Ni. The upper images show the 3D view of the red-framed areas. (b) HAADF and corresponding elemental mapping images of Aza-CMP-Ni.

Supplementary Discussion 2

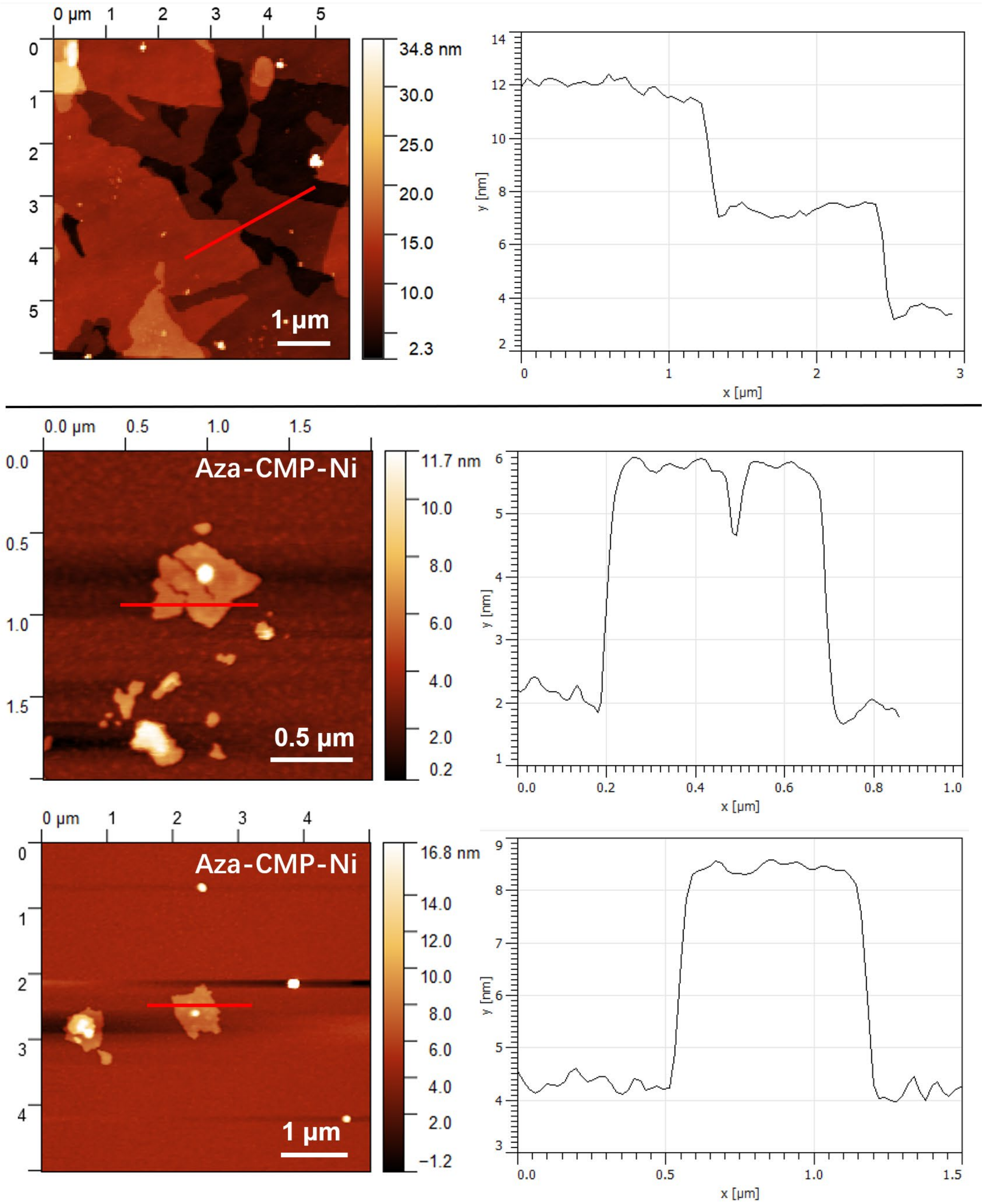

Supplementary Fig. 17 AFM characterization. AFM topographic images of a diluted (ethanol/water, 1:1 v/v) suspension of Aza-CMP and Aza-CMP-Ni deposited on SiO_2 and its corresponding height profiles along the red lines. A layered thickness of approximately 4 nm was observed in both Aza-CMP and Aza-CMP-Ni films, suggesting that the layered CMP matrix maintained its structure after Ni^{2+} complexation.

Supplementary Discussion 2

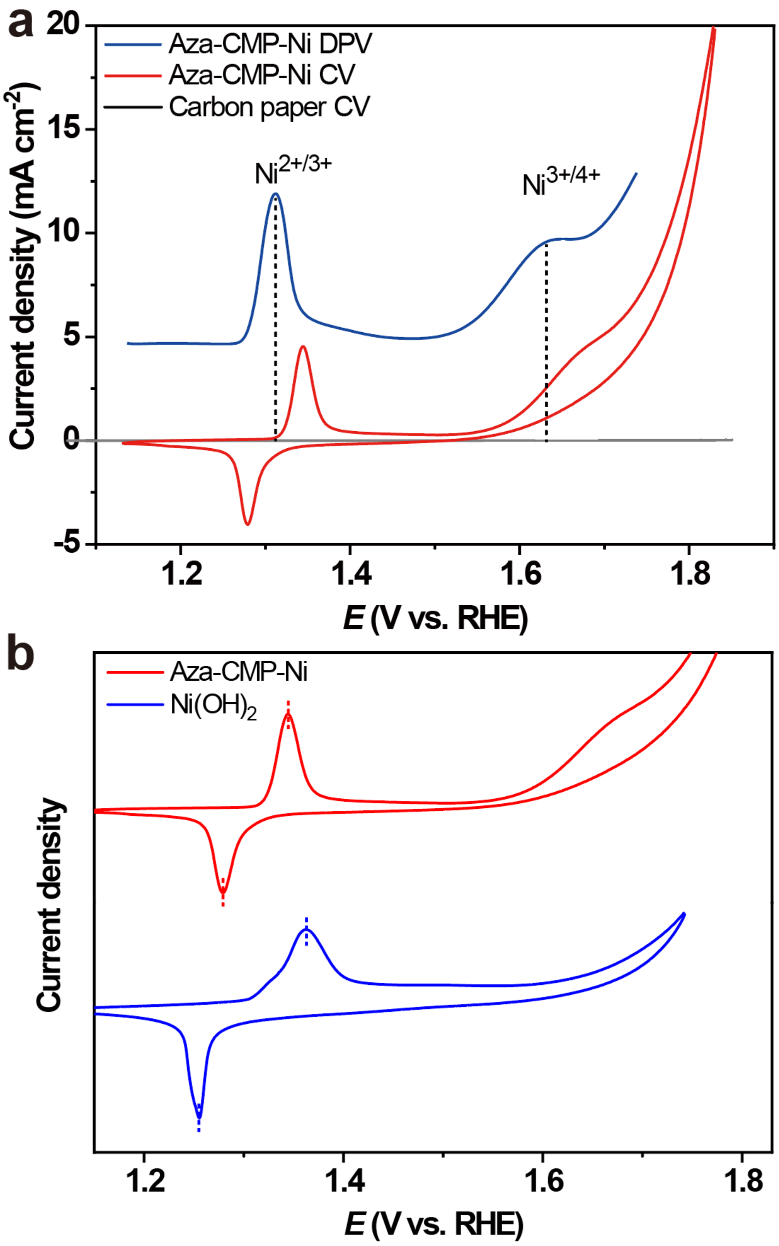

Supplementary Fig. 18 The redox response of the Aza-CMP-Ni. (a) CV and DPV curves of Aza-CMP-Ni in 1.0 M NaOH solution. (b) CV curves of Aza-CMP-Ni and nickel(II) hydroxide recorded in 1.0 M NaOH (scan rate: 50 mV s^{-1}, without iR compensation).

Supplementary Discussion 2

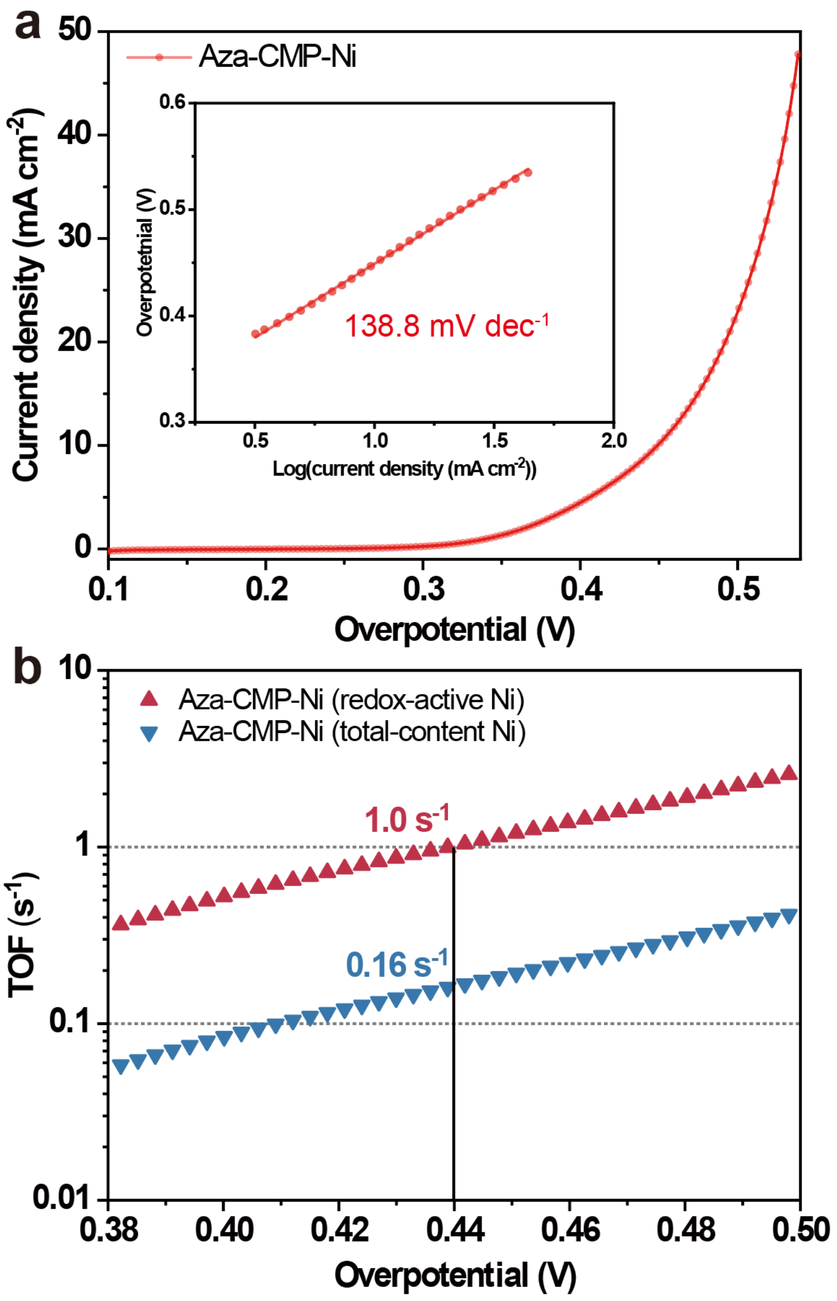

Supplementary Fig. 19 The OER activities of Aza-CMP-Ni/CP. (a) LSV curve of Aza-CMP-Ni in 1.0 M KOH (scan rate: 1 mV s^{-1}). Inset: corresponding Tafel slope. (b) TOFs of Aza-CMP-Ni based on the LSV curve in 1.0 M KOH. TOF values were calculated by ***Supplementary Eqn. 2***.

Supplementary Discussion 2

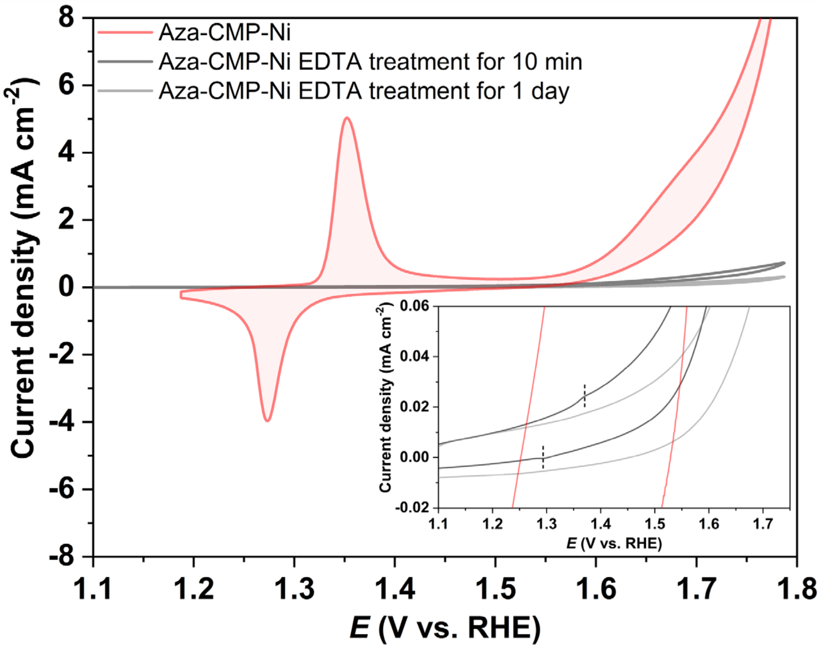

Supplementary Fig. 20 EDTA treatment. CV curves of Aza-CMP-Ni/CP before and after being treated with 0.01 M Na-EDTA aqueous solution (1.0 M NaOH, 50 mV s^{-1} scan rate, without iR compensation). Inset: magnified figure of the redox region.

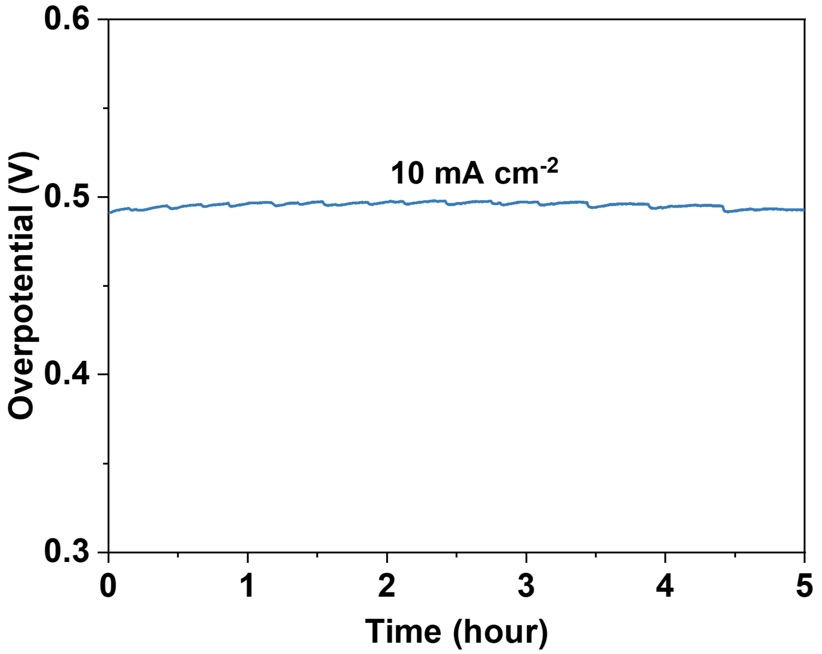

Supplementary Fig. 21 Catalytic stability test. Chronopotentiometry measurement of Aza-CMP-Ni at a current density of 10 mA cm^{-2} for 5 hours in 1.0 M KOH.

Supplementary Discussion 2

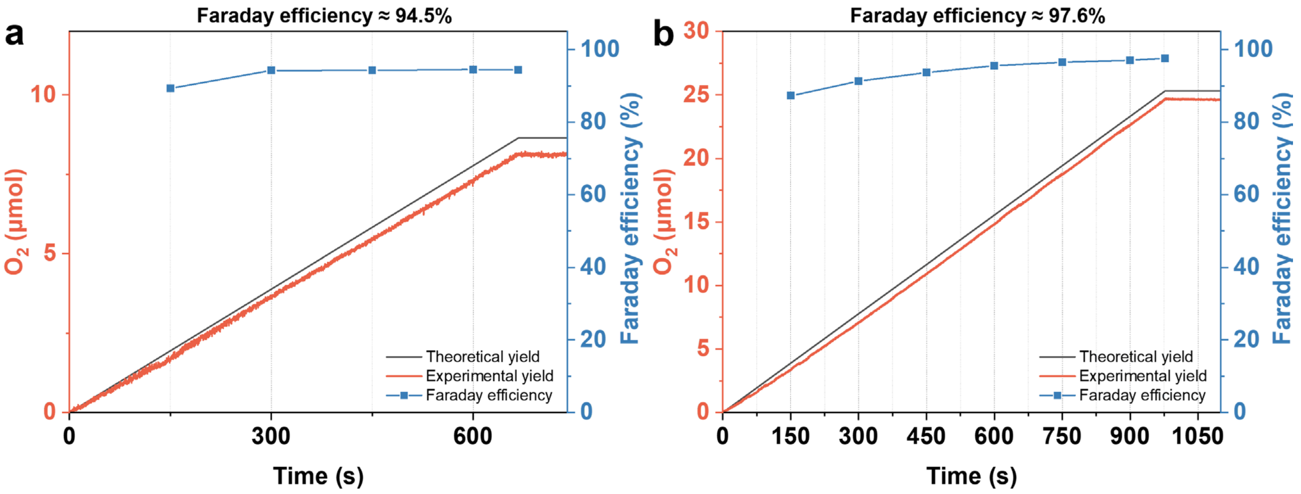

Supplementary Fig. 22 Faradaic efficiencies of Aza-CMP-Ni/CP electrode for OER in 1.0 M KOH. Theoretically calculated and experimentally measured amounts of O_2 were shown as functions of electric charge for water oxidation at a current density of (a) 5 mA cm^{-2} and (b) 10 mA cm^{-2}.

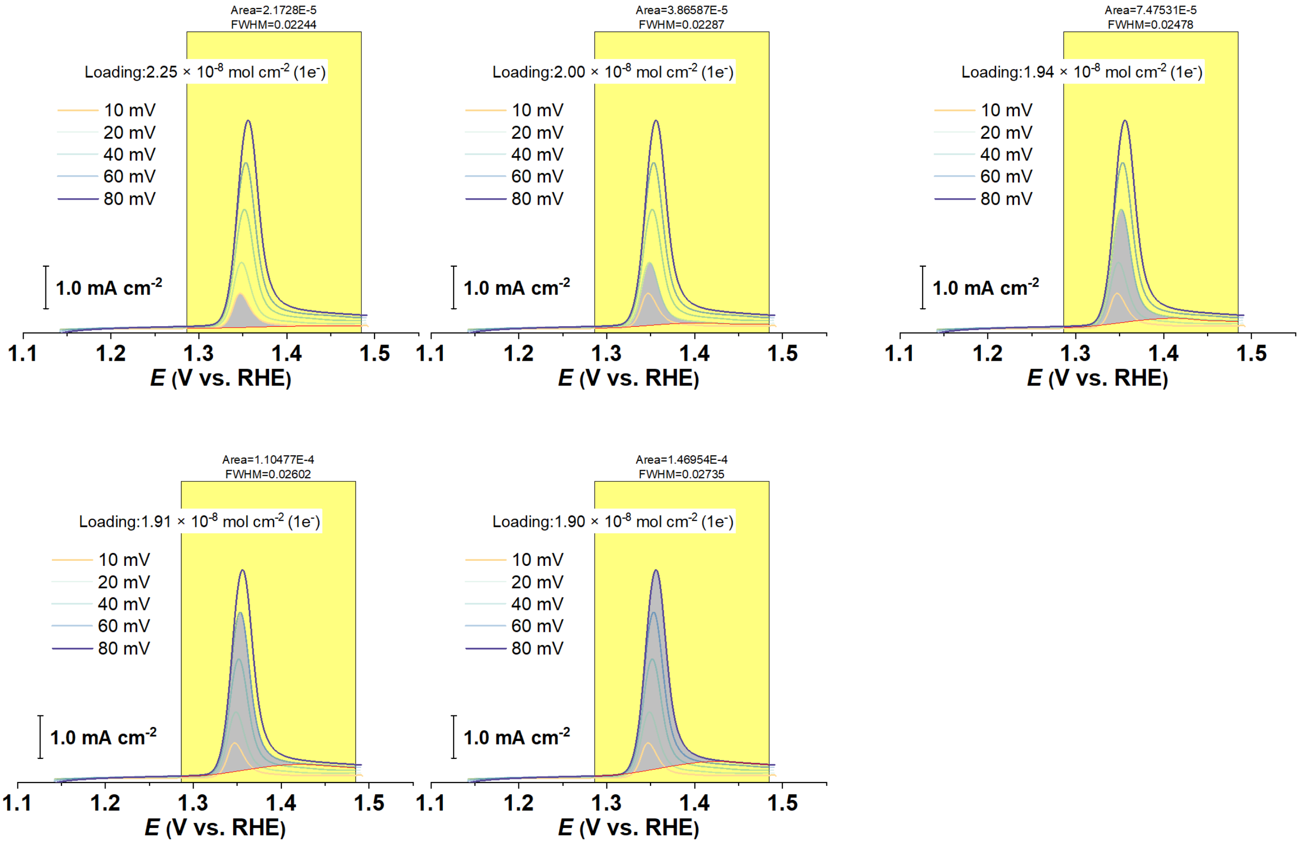

Supplementary Fig. 23 Loading of redox-active Ni site. Five parallel experiments for the loading calculation of redox-active Ni sites via the charge integration of oxidation peaks at different scan rates in 1.0 M NaOH. The electron transfer number of $Ni^{2+/3+}$ oxidation was set to 1.

Supplementary Discussion 3

Supplementary Discussion 3: Formation of OER active molecular Ni-Fe sites on Aza-CMP

High-temperature pyrolysis of metal-organic precursors is a widely used approach for synthesizing carbon-supported single-atom catalysts (SACs) and dual-atom catalysts (DACs), as highlighted in recent reviews.[47, 48] While researchers have explored ways to control precursor structures, compositions, and pyrolysis parameters to selectively form heteronuclear DAC structures, this approach often yields a mixture of species (e.g., M_1, M_2, M_1–M_1, M_2–M_2, M_1–M_2) rather than a single, well-defined M_1–M_2 structure. In contrast, the electrochemical synthesis method employed in this work follows a stepwise process that transforms atomically dispersed Ni sites into Ni-Fe active sites, enabling the selective formation of heteronuclear DAC structures. Synthesizing active structural species directly under applied potential and catalytic conditions helps mitigate potential catalyst structure evolution during operation. Furthermore, electrochemically induced formation of Ni-Fe active sites on SACs has been systematically investigated in other systems, demonstrating the broad applicability and feasibility of this fabrication strategy.[37, 38]

The formation of molecular Ni-Fe sites from Ni sites on Aza-CMP is studied using various techniques (***Supplementary Fig. 24***). The golden evidence to probe the transformation of single-site catalysts in Fe-contained electrolytes is to perform CV during catalysis. ***Supplementary Fig. 25*** illustrates the evolution of redox and OER currents on Aza-CMP-Ni during continuous CV scanning in Fe-free and Fe-saturated 1.0 M NaOH solutions. When activating Aza-CMP-Ni in a Fe-free NaOH solution, the redox currents increase until a stable value, while the oxidation ($E_{\text{Ni2+/3+, Aza-CMP-Ni}}$ = 1.36 V vs. RHE) and reduction ($E_{\text{Ni3+/2+, Aza-CMP-Ni}}$ = 1.28 V vs. RHE) peaks remain at constant values, which indicates the increased number of active Ni species (***Supplementary Fig. 25a***). However, when using Fe-saturated NaOH electrolyte, the catalytic performance improves rapidly along with a significant anodic shift in the redox potential (***Supplementary Fig. 25b***). Continuous CV scanning was also performed using a blank Aza-CMP/CP electrode in a Fe-saturated 1.0 M NaOH solution (***Supplementary Fig. 26***). The results show that only a slight increase in current is observed after 100 CV cycles, which excludes the potential current contribution from single Fe^{3+} sites bonded with Aza-CMP. Additionally, as shown in ***Supplementary Fig. 27***, the potential-controlled CV experiment was conducted in Fe-saturated NaOH electrolyte to confirm that the binding of Fe sites is not reliant on the generation of Ni^{3+} ions. In other words, Fe atoms can spontaneously bind after the Ni^{2+} site is activated by the solvent. ***Supplementary Fig. 28*** presents the intermediate LSV during continuous scanning (denoted as Aza-CMP-NiFe$_x$, $0 < x < 1$), in which two reduction peaks could be clearly identified during the evolution: The first reduction peak at 1.28 V vs. RHE belongs to Aza-CMP-Ni; the emerging reduction peak at 1.31 V vs. RHE is attributed to the generated Ni-Fe sites in Aza-CMP-NiFe$_x$. The forward scanning LSV contour plots in ***Supplementary Figs. 25c*** and ***25d*** visualize the evolution of oxidation currents with the number of scans. The current profile of Aza-CMP-Ni (at 1.36 V vs. RHE) shows an increase in $Ni^{2+/3+}$ peak currents that stabilized after 120 cycles, indicating gradual activation of surface Ni sites during operation. On the other hand, for the current profile of Aza-CMP-NiFe$_x$, there is an initial increase and subsequent decrease in $Ni^{2+/3+}$ peak currents at 1.36 V vs. RHE, while those at 1.385 V keep growing and stabilize after 200 cycles. During activation in solution, the surface Ni sites are first activated and gradually transformed to Ni-Fe sites, which leads to a competition between activation and transformation and subsequently results in the vertex of the current profile at 1.36 V vs. RHE. The plots of peak current vs. scan number for the reduction peaks are depicted in ***Supplementary Fig. 29***, exhibiting a similar trend to that of the oxidation current. To the best of our knowledge, it would be the first time that the transformation of Ni-Fe sites from Ni sites was intuitively presented via electrochemical redox evolution. As the $Ni^{2+/3+}$ redox and OER current stabilized after

CV cycles, the catalyst on carbon paper is denoted as **Aza-CMP-NiFe/CP**.

The formation of Ni-Fe sites was examined using various spectroscopic techniques. XPS spectra of Aza-CMP-NiFe in the Ni 2*p* region display doublet peaks centered at 855.7 ($2p_{3/2}$) and 873.0 eV ($2p_{1/2}$), corresponding to those observed in electrochemically activated Aza-CMP-Ni (***Supplementary Fig. 30a***). In the Fe 2*p* region, a dominant peak at 710.9 eV indicates the presence of Fe^{3+} species in Aza-CMP-NiFe (***Supplementary Fig. 30b***). The O 1*s* region displays two peaks at 530.8 and 531.8 eV, attributed to surface hydroxyl and water groups, respectively, while the peak at 533.0 eV corresponds to superficial C=O species on the carbon paper support (***Supplementary Fig. 30c***).[49, 50, 51] Notably, Aza-CMP-NiFe exhibits an additional O 1*s* signal at 529.8 eV, suggesting the formation of a M–O–M structure on the catalyst. Furthermore, Aza-CMP-NiFe shows a peak in the N 1*s* region similar to that of Aza-CMP-Ni, indicating a retained coordination environment following Fe species bonding (***Supplementary Fig. 30d***). The similar Raman spectra obtained for Aza-CMP-Ni and Aza-CMP-NiFe suggest the absence of metal oxide phases during the activation process (***Supplementary Fig. 31***).

The normalized Ni K-edge XANES absorption threshold of Aza-CMP-NiFe exhibits a slight positive shift compared to Aza-CMP-Ni, suggesting a decrease in electron density at the Ni sites following the insertion of Fe sites (***Supplementary Fig. 32***). The comparable pre-edge peaks of Aza-CMP-Ni and Aza-CMP-NiFe indicate that the incorporation of Fe sites does not dramatically alter the coordination geometry of Ni sites. In the normalized Fe K-edge XANES spectra, the pre-edge peak of Aza-CMP-NiFe displays a relatively low intensity, particularly revealing symmetry between six-coordinated iron(III) acetylacetonate and five-coordinated iron(III) meso-tetra(4-carboxyphenyl)porphine chloride (TCPP) at the Fe sites in Aza-CMP-NiFe, indicating a distorted octahedral geometry (***Supplementary Fig. 33***).[52, 53] The rising-edge energy of Aza-CMP-NiFe implies a +3 valence state for the Fe sites, consistent with XPS results. The Ni K-edge Fourier transform EXAFS spectra of Aza-CMP-Ni and Aza-CMP-NiFe present nearly identical dominant peaks in the first-shell region between 1 and 2 Å, signifying that the Ni sites in both materials have the same local coordination configuration (***Supplementary Fig. 34***). Concurrently, an additional peak in the second-shell region for Aza-CMP-NiFe suggests the presence of another metal center (i.e., Fe) surrounding the Ni sites after electrochemical treatment in Fe-saturated electrolyte.[37, 54] The outer shell of Fe sites is centered at R = 2.64 Å, distinct from the contributions of M–O–M units in Ni-based materials (R = 2.76 Å for $Ni(OH)_2$ and R = 2.55 Å for $LiNiO_2$) and the backbone carbon in Aza-CMP (R = 2.48 Å). The Fe K-edge EXAFS data of Aza-CMP-NiFe is presented in ***Supplementary Fig. 35***. The dominant peak in the second shell is identified by the intensity maximum at R = 2.62 Å, reflecting that Fe sites are likely bonded to neighboring Ni sites with a radial distance of approximately 3.0 Å. In contrast, the second-shell maxima for NiFe hydroxide, Fe_2O_3, and iron(III) TCPP are located at 2.69 Å, 2.57 Å, and 2.47 Å, respectively. Ni K-edge WT analysis of Aza-CMP-NiFe and Aza-CMP-Ni, presented in ***Supplementary Fig. 36***, displays identical intensity contours centered at 4.7 $Å^{-1}$ in the first-shell region, attributed to similar Ni–N/O contributions. The additional intensity maximum at 5.7 $Å^{-1}$ in Aza-CMP-NiFe, derived from the neighboring metal atom(s), is distinct from the metallic Ni-Ni path (nickel foil, k = 7.5 $Å^{-1}$), oxide Ni–Ni path ($Ni(OH)_2$ and $LiNiO_2$, k = 7.3 $Å^{-1}$). Similarly, Fe K-edge WT analysis of Aza-CMP-NiFe reveals two intensity maxima at 4.6 and 5.7 $Å^{-1}$, corresponding to Fe–O and Fe–Ni contributions, respectively (***Supplementary Fig. 37***). These results are clearly distinguishable from the metallic Fe–Fe path (iron foil, k = 7.95 $Å^{-1}$), oxide Fe–M path (NiFe hydroxide, k = 7.2 $Å^{-1}$), and oxide Fe–Fe path (Fe_2O_3, k = 7.1 $Å^{-1}$), suggesting the presence of a unique dimer Ni–O–Fe oxo structure for the Ni-Fe sites. The corresponding simulated EXAFS WT of the DFT model displays two intensity maxima at approximately 4.8 $Å^{-1}$ and 6.3 $Å^{-1}$ (see ***Supplementary Notes 1*** for the details of simulation methods), attributed to the Ni–O/N and Ni–Fe contributions, respectively (***Supplementary***

Fig. 38a). The position of the second shell peak (6.4 $Å^{-1}$) is lower than the metallic Ni–Ni path and oxide Ni–Ni path, further confirming the unique Ni-Fe dual-metal atom feature in the experimental structure. In the R space, both simulated and experimental curves exhibit a single intensity maximum in the second-shell region. The relatively lower $1_{st}/2_{nd}$ peak intensity ratio suggests a non-integer coordination number of Fe, as not all Ni atoms in the framework form Ni-Fe sites of Aza-CMP-NiFe (***Supplementary Fig. 38b***). The simulation outcomes for the Ni and Fe K edge XANES spectra, predicated on the DFT structural model, align closely with the experimental findings, further underscoring the formation of dual-metal-atom NiFe sites (***Supplementary Fig. 39***). All simulation EXAFS and XANES results consistent with experimental spectra and support the molecular Ni-Fe site nature of the as-fabricated Aza-CMP-NiFe, enabling further OER kinetic studies.

The atomic-resolution HAADF-STEM image reveals a high density of bright dots, confirming the abundance of metal sites in Aza-CMP-NiFe (***Supplementary Fig. 40a***). Although the profuse presence of metal sites within the porous structure hinders Ni-Fe site identification, some potential double-metal sites with distances between 2 to 3 Å remain visible in the thinner regions of the sample. As shown in ***Supplementary Fig. 40b***, the distances of two selected metal pairs are 2.99 and 2.96 Å. Furthermore, following phase correction in the EXAFS spectra, the outer shell of the Fe centers is identified by the prominent peak at R = 2.96 Å in ***Supplementary Fig. 40c***. This finding aligns with the distance observed at the Ni centers, suggesting that the Fe site is bonded to neighboring sites of the Ni center with a spatial distance of around 3.0 Å in Aza-CMP-NiFe. Additionally, the corresponding TEM elemental mapping demonstrates a clear and uniform distribution of Ni, Fe, C, and N elements, indicating that the Ni-Fe sites in Aza-CMP-NiFe are at molecular scales and devoid of aggregated nanoparticles (***Supplementary Fig. 41***). EDTA ligand in solution would lead to the detachment of molecular sites from Aza-CMP-NiFe. As illustrated in ***Supplementary Fig. 42***, after immersing Aza-CMP-NiFe in an EDTA solution for 10 minutes, the $Ni^{2+/3+}$ redox becomes indistinct, and the OER current significantly decreases; following a 24-hour treatment, all redox and OER currents diminish. These findings indicate that the Ni-Fe sites remain molecularly dispersed and can be removed from Aza-CMP by using EDTA with strong chelating ligands.[37, 45]

The total metal loadings of nickel and iron on the Aza-CMP-NiFe/CP electrode, as determined by ICP-OES, are 8.81×10^{-8} and 2.23×10^{-8} mol $cm^{-2}{}_{geo}$, respectively. This indicates that the maximum loading of Ni-Fe sites is 2.23×10^{-8} mol $cm^{-2}{}_{geo}$. The number of the transferred electrons during the $Ni^{2+/3+}$ redox is then estimated by the charge of the oxidation peak (***Supplementary Fig. 44***). Based on the LSV curves under different scan rates, the charge transfer can be calculated under different scenarios, depending on how the metal content is defined: 1) If all Ni atoms (8.81 × 10-8 mol cm^{-2}) are assumed to undergo oxidation, the number of electrons transferred per Ni site is calculated to be 0.44-0.55; 2) If all Fe atoms (2.23×10^{-8} mol cm^{-2}) are assumed to be oxidized, the charge transfer per Fe site is 1.74-2.13 electrons; 3) If all the Fe atoms and neighboring bonded Ni sites (i.e., Ni-Fe units) participate in the oxidation, the average number of electrons transferred per metal site is 0.87-1.07. Given the unfavorable thermodynamics of a two-electron redox event at a single metal center, the last scenario is the most plausible, suggesting that each metal in the Ni-Fe unit undergoes a one-electron oxidation, collectively accounting for the observed two-electron transfer. The two-electron transfer feature of $Ni^{2+/3+}$ redox in Aza-CMP-NiFe is further supported by Pourbaix analysis. The slope of the redox potential versus pH plot directly reflects the proton-to-electron transfer ratio (H^+/e^-) involved in the redox process. In our case, the $Ni^{2+/3+}$ redox potential exhibits a slope of −96.4 mV pH^{-1}, corresponding to a $3H^+/2e^-$ transfer process (theoretical slope = −59 × 3/2 = −89 mV pH^{-1}) rather than a single electron transfer (−59 mV pH^{-1} for $1H^+/1e^-$; −118 mV pH^{-1} for $2H^+/1e^-$) (***Supplementary Fig. 72***). Moreover, across a broad pH range from near-neutral to strongly alkaline conditions (pH 7-

14), the $Ni^{2+/3+}$ redox couple exhibits a consistent pH dependence of approximately −90 mV pH^{-1}, further supporting a $3H^{+}/2e^{-}$ transfer process during the oxidation (***Supplementary Fig. 88c***).

Based on the two-electron transfer involved in the $Ni^{2+}Fe^{3+}$ oxidation process, the density of redox-active Ni-Fe sites on the electrode is calculated to be 1.80×10^{-8} mol $cm^{-2}{}_{geo}$, using the linear relationship between the $Ni^{2+/3+}$ peak current and the scan rate (***Supplementary Fig. 45***). This value closely matches the measured Fe content, suggesting that most Fe atoms are redox-active and coordinated with surface Ni sites to form Ni-Fe active centers. The surface redox-active Ni-Fe content would constitute around 20% of the total Ni content. From a structural perspective, the pristine Aza-CMP framework has a pore size of approximately 1.2 nm. Upon incorporation of NiFe sites, the pore size is further reduced to the sub-nanometer range, which would hinder electrolyte diffusion and limit the electrochemically active species to only a fraction of the total metal content. For example, in the similar Aza-CMP-Co system,[3] the electrochemically active Co species account for 10% of the total Co content.

This conclusion is further supported by subsequent *operando* electrochemical XAS measurements. As shown in ***Supplementary Table 2***, the Ni K edge XANES spectra reveal that the oxidation state of Ni in Aza-CMP-NiFe increases from 2.12 at 1.2 V to 2.32 at 1.5 V vs. RHE, corresponding to a change of 0.20. This suggests that approximately 20% of Ni sites (1.8×10^{-8} mol cm^{-2}) are oxidized from Ni^{2+} to Ni^{3+} within this potential range, closely matching the total Fe content. Such consistency supports the hypothesis that only about 1/4 of the Ni sites are electrochemically accessible and actively form Ni-Fe structures during the activation process. Further evidence is provided by the pre-edge features of the Ni-Fe XANES spectra. As shown in ***Supplementary Fig. 64b***, although Ni^{3+} formation is evident at 1.5 V vs. RHE, a substantial fraction of Ni remains in the Ni^{2+} state. Even at 1.7 V vs. RHE, where the applied overpotential is significantly higher, Ni^{2+} features persist. These results suggest that only surface or near-surface Ni sites are electrochemically active, while a significant portion of Ni^{2+} is trapped in the bulk, likely due to the limited accessibility of the electrolyte.

Notably, due to the rapid OER kinetics, the overpotential requirements at current densities of 10 and 50 mA cm^{-2} for the Aza-CMP-NiFe/CP electrode are only 256 and 278 mV, respectively. In sharp contrast, the onset overpotential of the Aza-CMP-Ni/CP (i.e., without Fe) is 341 mV, with a much higher Tafel slope of 139 mV dec^{-1}, indicating sluggish OER kinetics and a different RDS (***Supplementary Fig. 19a***). To demonstrate the industrial potential of the catalyst, increasing the catalyst loading by five times results in a significant rise in the catalytic current (***Supplementary Fig. 43b***). At 60°C, the high-loading Aza-CMP-NiFe/CP electrode requires only an overpotential of 300 mV to achieve a catalytic current of 2000 mA cm^{-2}, highlighting the suitability of Aza-CMP-NiFe for industrial applications.

The durability of the Aza-CMP-NiFe/CP electrode was assessed in 1.0 M KOH. Following 15 hours of electrolysis at a current density of 10 mA cm^{-2}, the overpotential requirements of Aza-CMP-NiFe/CP remain constant at approximately 275 mV throughout the test, indicating robust catalytic stability under the operating conditions (***Supplementary Fig. 47***). The Faraday efficiency is determined to be 97.2% and 99.5% at current densities of 5 and 10 mA cm^{-2} in 1.0 M KOH solution, respectively, which suggests that the accumulated charge is effectively utilized for oxygen production (***Supplementary Fig. 48***).

Supplementary Discussion 3

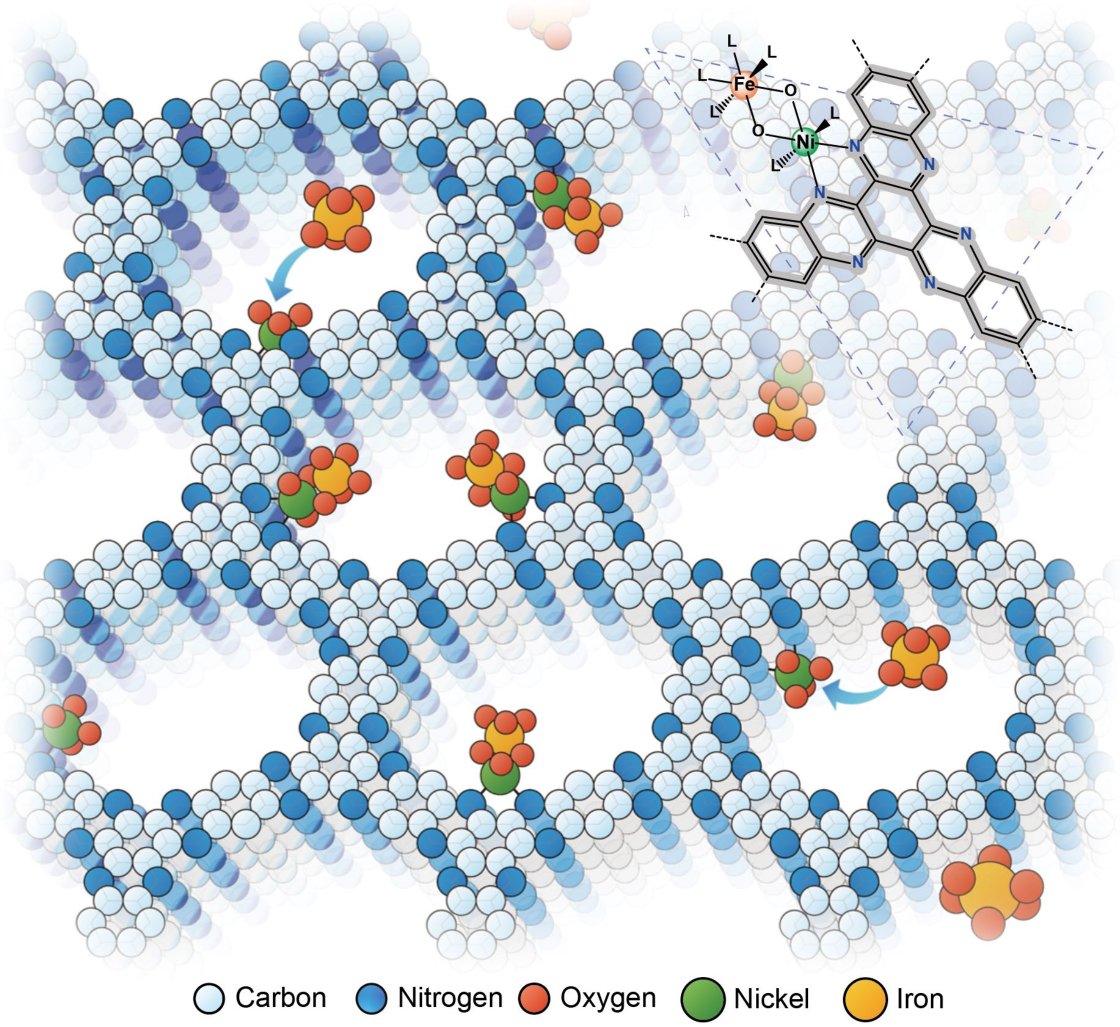

Supplementary Fig. 24 Schematic structure of the Aza-CMP-NiFe catalyst. L represents coordinated H_2O/OH^- ligands.

Supplementary Discussion 3

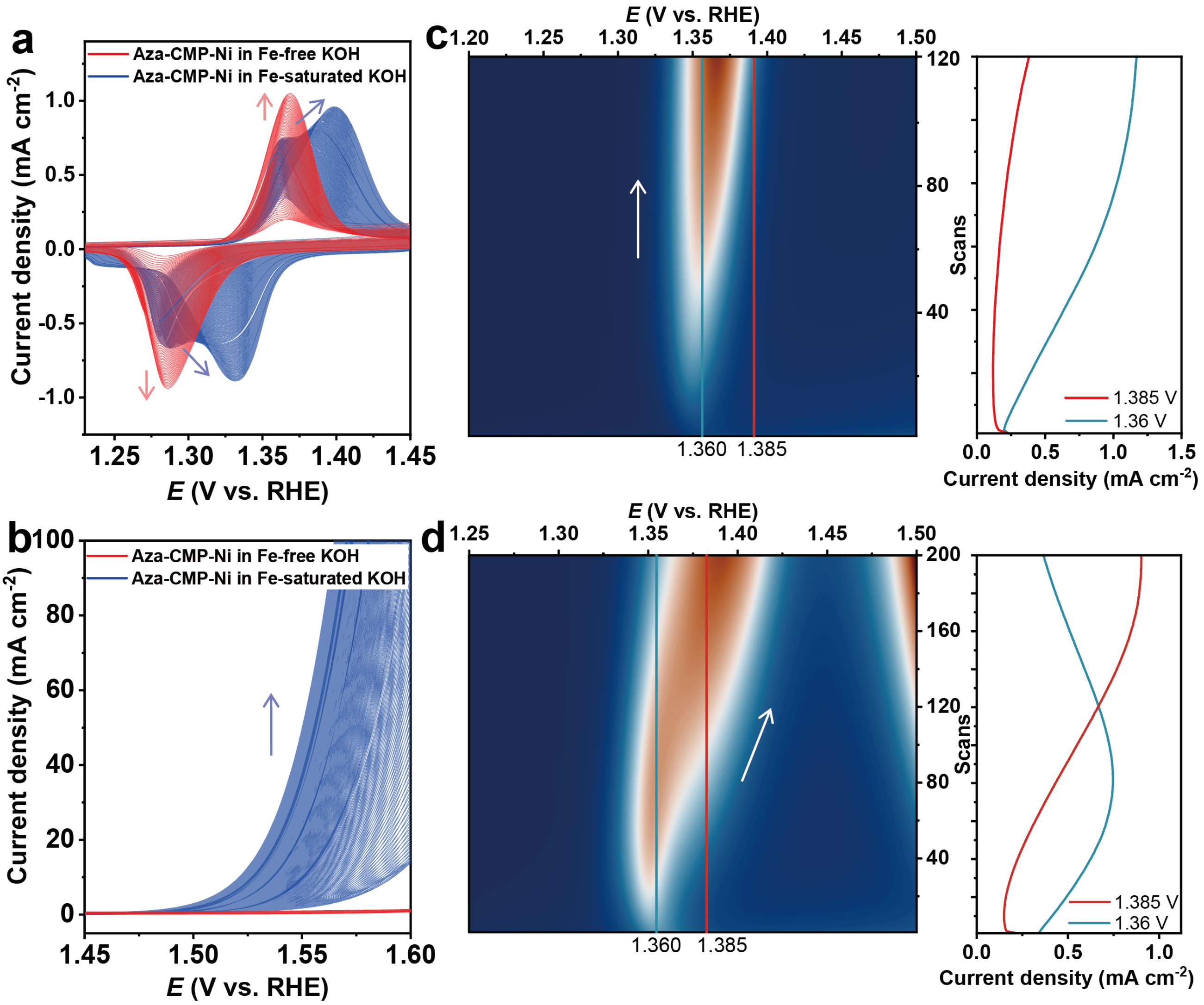

Supplementary Fig. 25 The evolution of CV curves. (a-b) Continuous CV scanning of Aza-CMP-Ni in Fe-free and Fe-saturated 1.0 M NaOH solutions (scan rate: 50 mV s^{-1}, without iR compensation). LSV evolution of Aza-CMP-Ni in (c) Fe-free and (d) Fe-saturated 1.0 M NaOH solutions, along with corresponding peak current profiles; the x-axis and y-axis represent potential and scan numbers, respectively, while the z-axis denotes the current intensity.

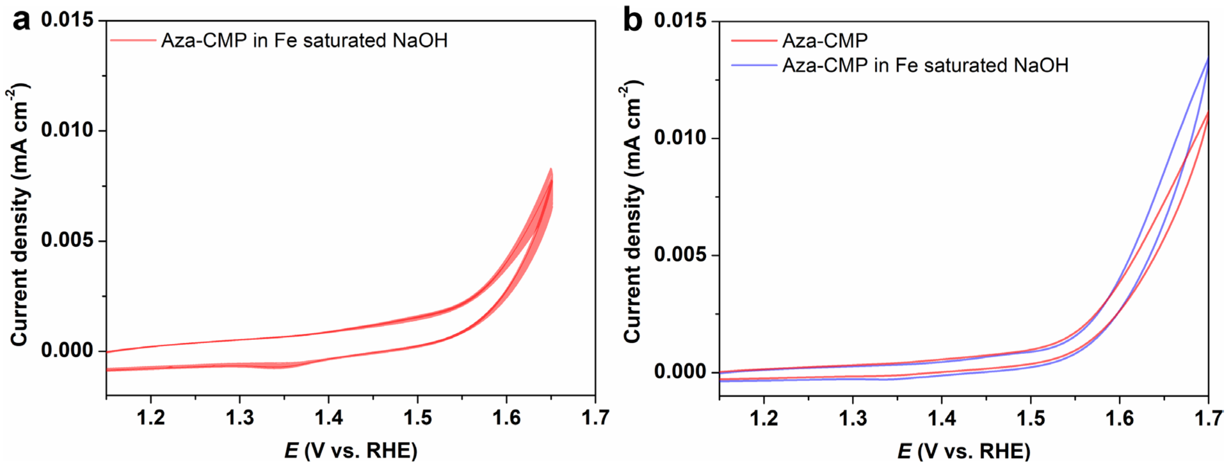

Supplementary Fig. 26 CV curves of Aza-CMP in Fe-saturated electrolyte. (a) CV curves of Aza-CMP in Fe-saturated 1.0 M NaOH solution (scan rate: 20 mV s^{-1}). (b) CV curves of pristine Aza-CMP and CV-treated Aza-CMP after 100 cycles in Fe-saturated 1.0 M NaOH solution (scan rate: 5 mV s^{-1}).

Supplementary Discussion 3

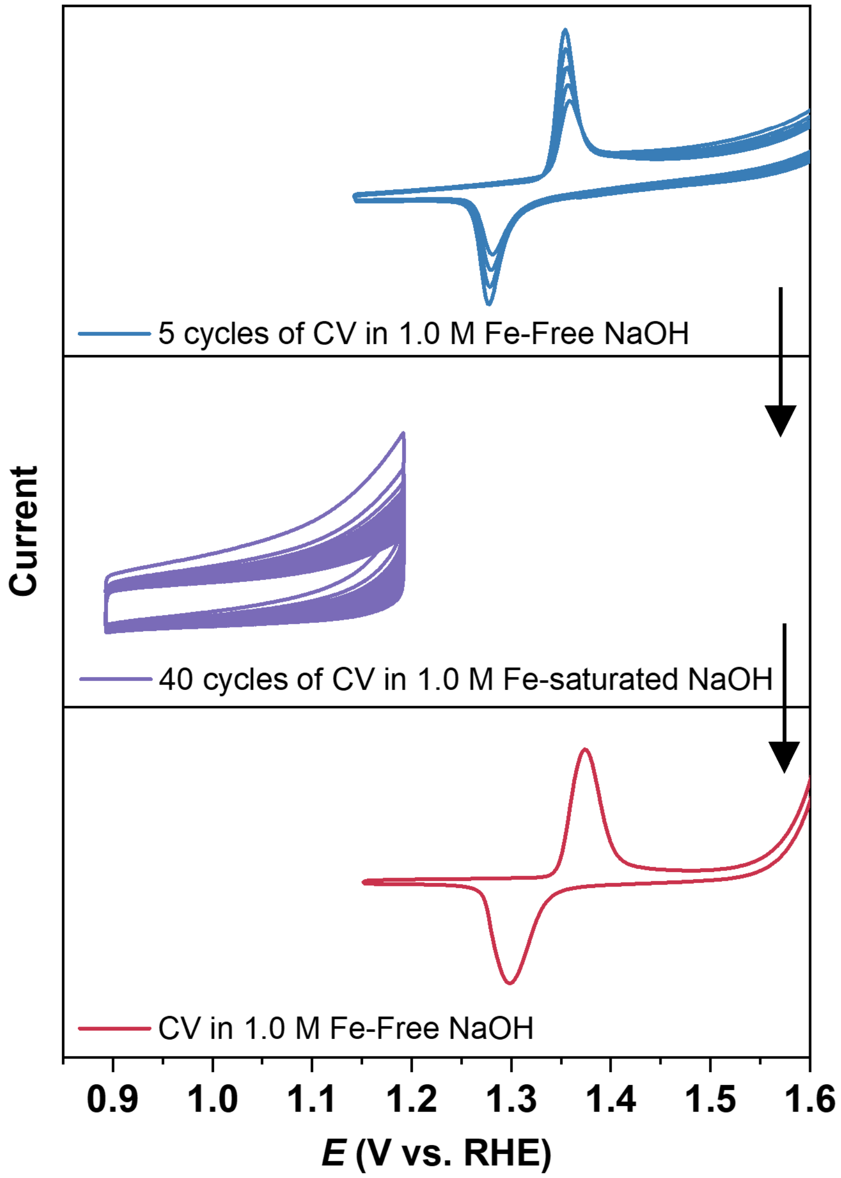

Supplementary Fig. 27 Potential-controlled formation of Ni-Fe sites. The Aza-CMP-Ni/CP electrode was initially activated in Fe-free NaOH (blue curves). Subsequently, the activated Aza-CMP-Ni/CP was transferred to a Fe-saturated electrolyte and subjected to CV under potential windows that prevent the formation of Ni^{3+} (purple curves). Finally, the CV-treated Aza-CMP-Ni/CP was tested in Fe-free NaOH to examine the changes in redox and catalytic onset. The anodic shifted $Ni^{2+/3+}$ redox potential and the cathodic shift in the OER onset potential (red curve) suggest the formation of Ni-Fe sites in the absence of the Ni^{3+} state.

Supplementary Discussion 3

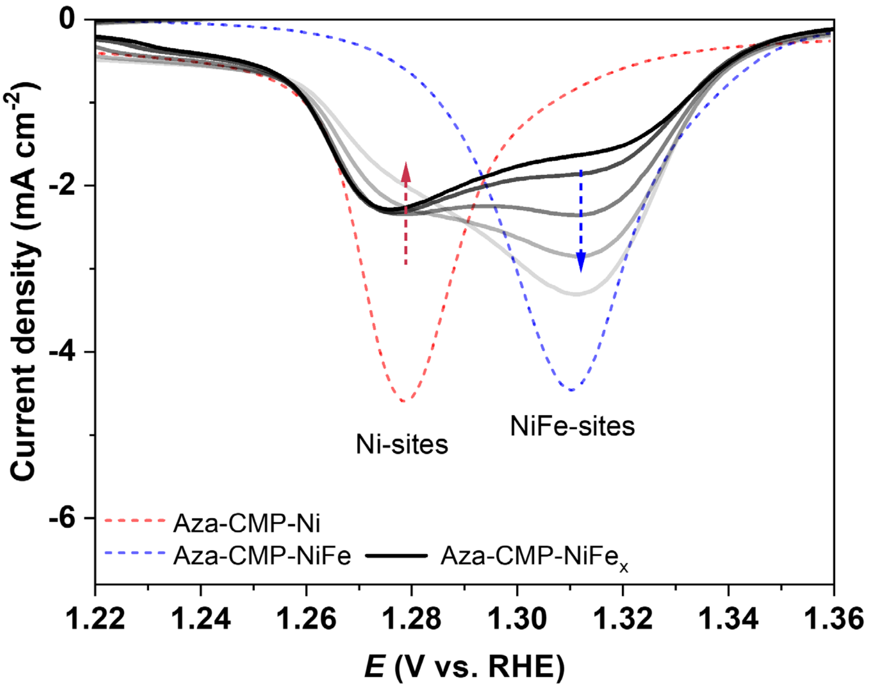

Supplementary Fig. 28 Evolution of reduction peak in CV measurements during the formation of Ni-Fe sites. The LSV curve in the reduction direction is shown. Data was extracted from ***Supplementary Fig. 25a***.

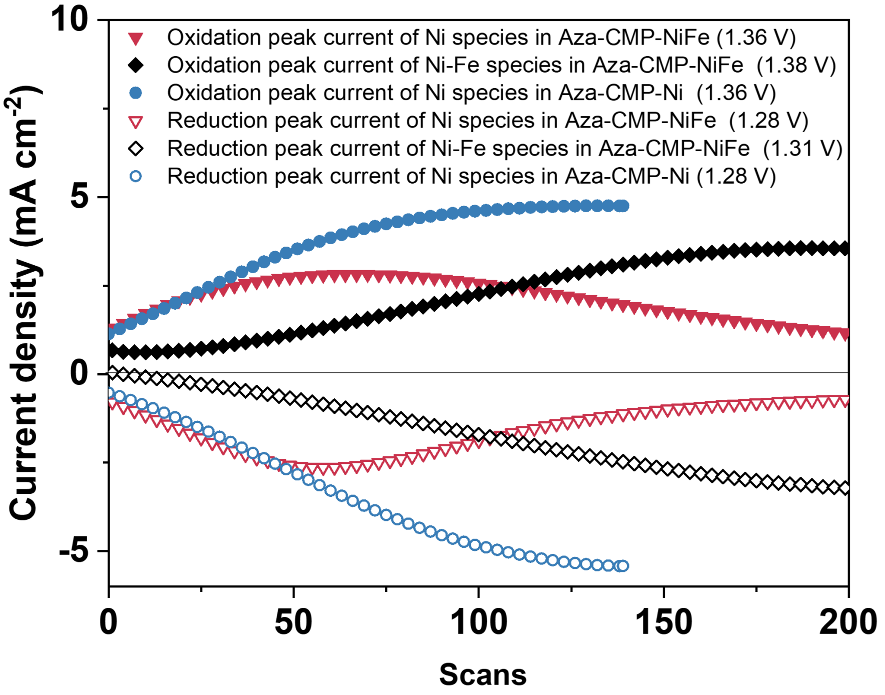

Supplementary Fig. 29 Current densities of oxidation and reduction peaks during the CV activation of Aza-CMP-Ni and Aza-CMP-NiFe. The current density at a specific potential indicates the amount of each redox-active species. Data was extracted from ***Supplementary Fig. 25a***.

Supplementary Discussion 3

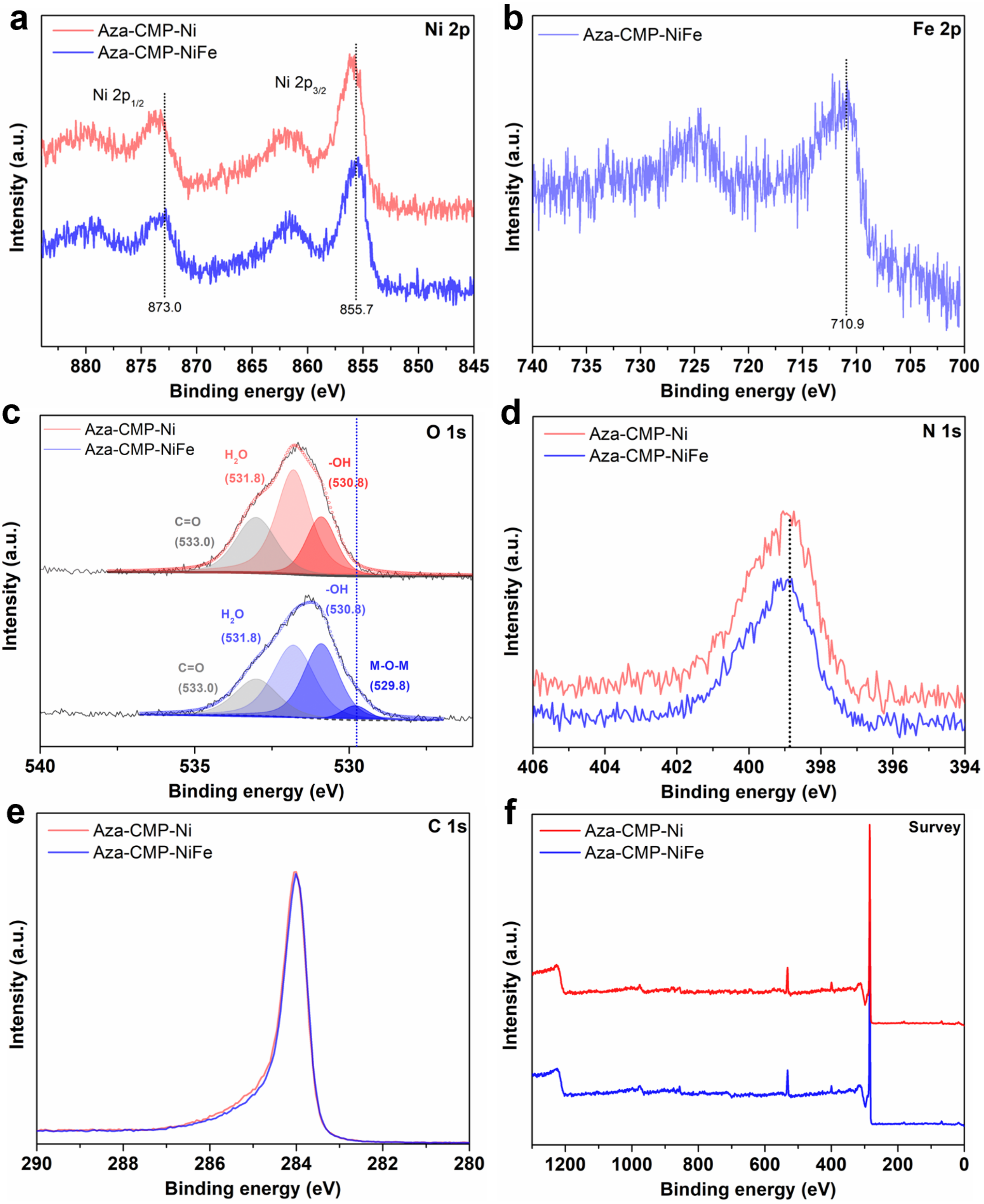

Supplementary Fig. 30 High-resolution XPS spectra of activated Aza-CMP-Ni and Aza-CMP-NiFe. (a) Ni 2*p*, (b) Fe 2*p*, (c) O 1*s*, (d) N 1*s*, (e) C 1*s*, and (f) survey regions.

Supplementary Discussion 3

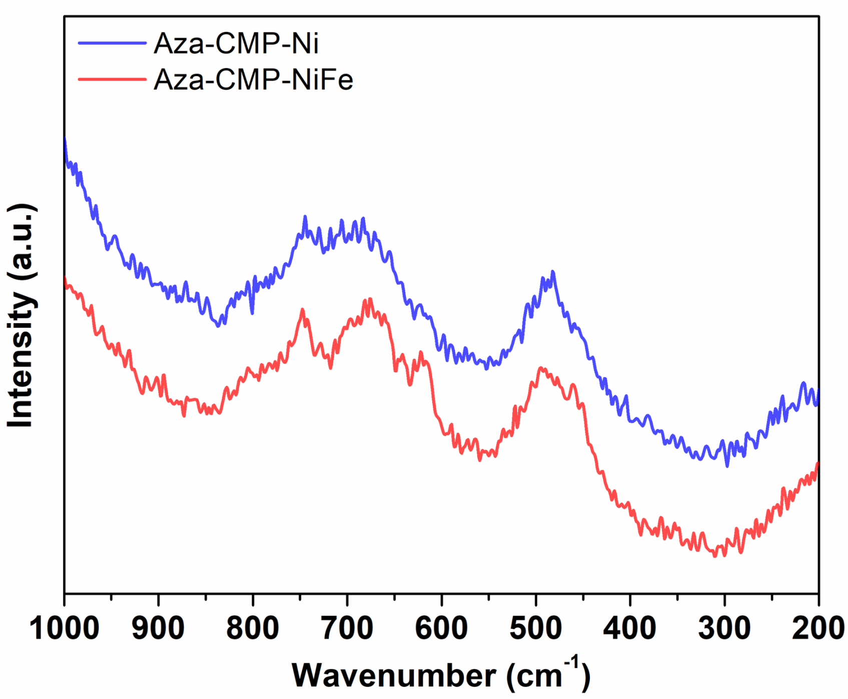

Supplementary Fig. 31 Raman spectra of Aza-CMP-Ni and Aza-CMP-NiFe.

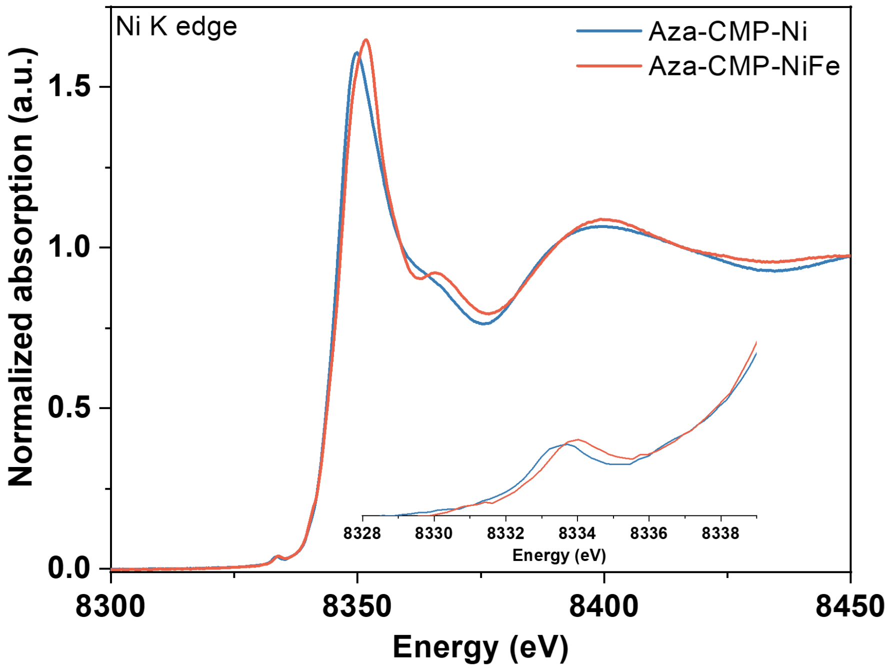

Supplementary Fig. 32 Normalized Ni K-edge XANES spectra of Aza-CMP-Ni and Aza-CMP-NiFe. The inset figure highlights the pre-edge region.

Supplementary Discussion 3

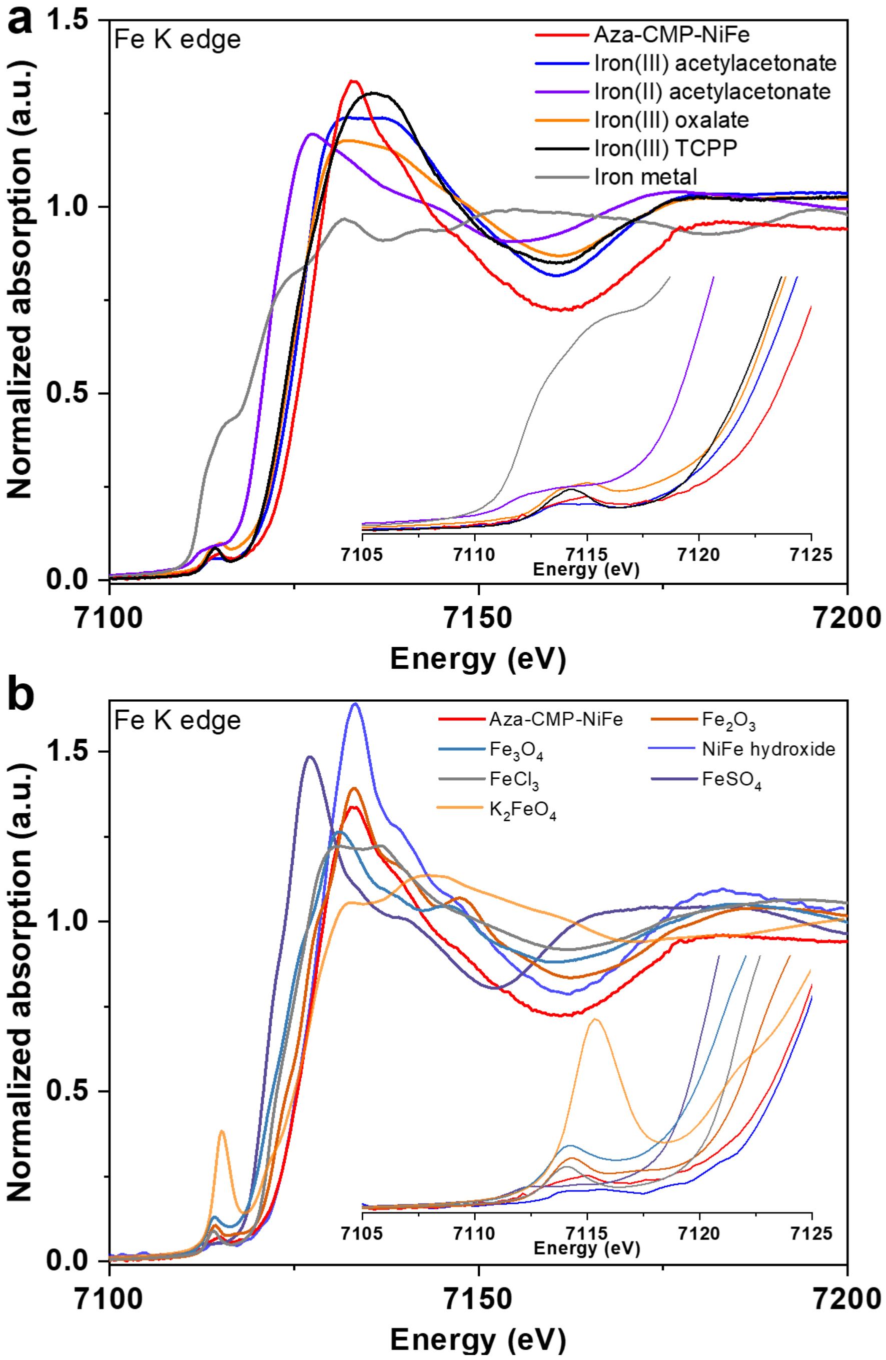

Supplementary Fig. 33 Normalized Fe K-edge XANES spectra of Aza-CMP-NiFe and reference materials. Compare with (a) metal-organic materials and (b) inorganic materials. The inset figures highlight the pre-edge region.

Supplementary Discussion 3

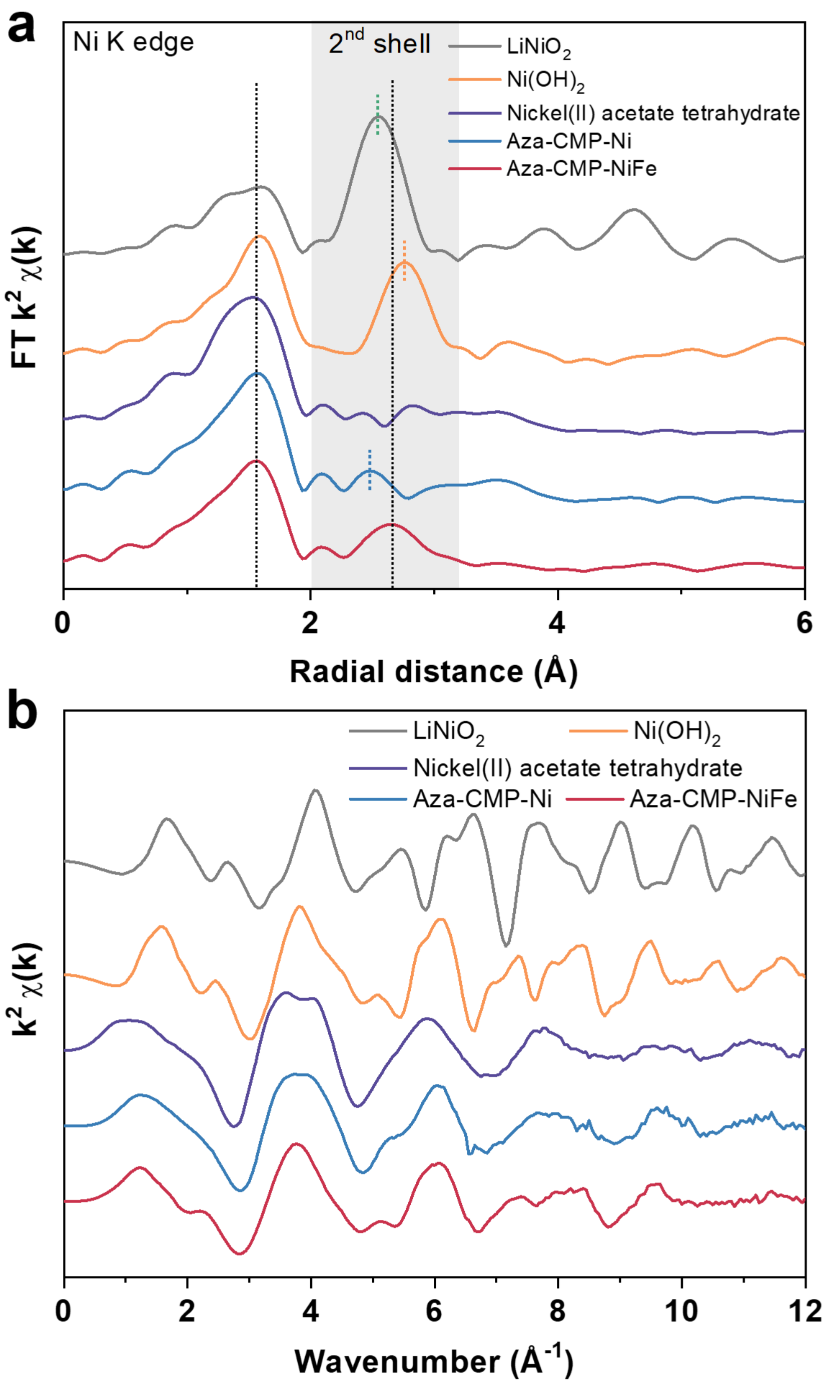

Supplementary Fig. 34 Ni K-edge Fourier transform EXAFS spectra. (a) k^2-weighted Ni K-edge Fourier transform EXAFS spectra of Aza-CMP-Ni, Aza-CMP-NiFe, and reference materials and (b) corresponding Fourier-transformed magnitude in k-space. In the case of Aza-CMP-Ni, the second shell peak is attributed to the backbone carbon and nitrogen atoms, while the second-shell peak for $Ni(OH)_2$ and $LiNiO_2$ originates from adjacent metal atoms in the lattices.

Supplementary Discussion 3

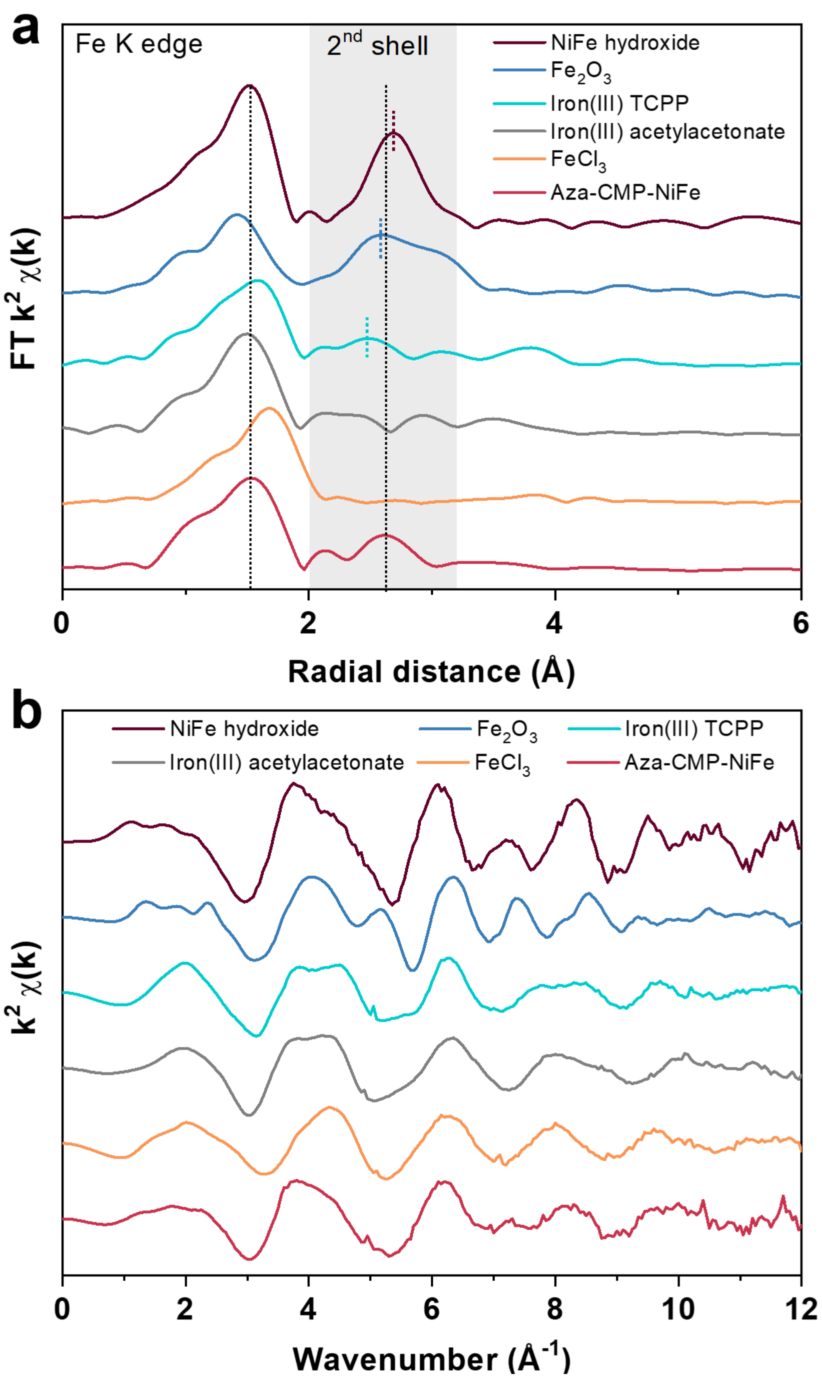

Supplementary Fig. 35 Fe K-edge Fourier transform EXAFS spectra. (a) k_2-weighted Fe K-edge Fourier transform EXAFS spectra of Aza-CMP-NiFe and reference materials and (b) corresponding Fourier-transformed magnitude in k-space. In the case of Fe(III) TCPP, the second shell peak is attributed to the backbone carbon and nitrogen atoms, while the second shell peak for NiFe hydroxide and Fe_2O_3 originates from adjacent metal atoms in the lattices.

Supplementary Discussion 3

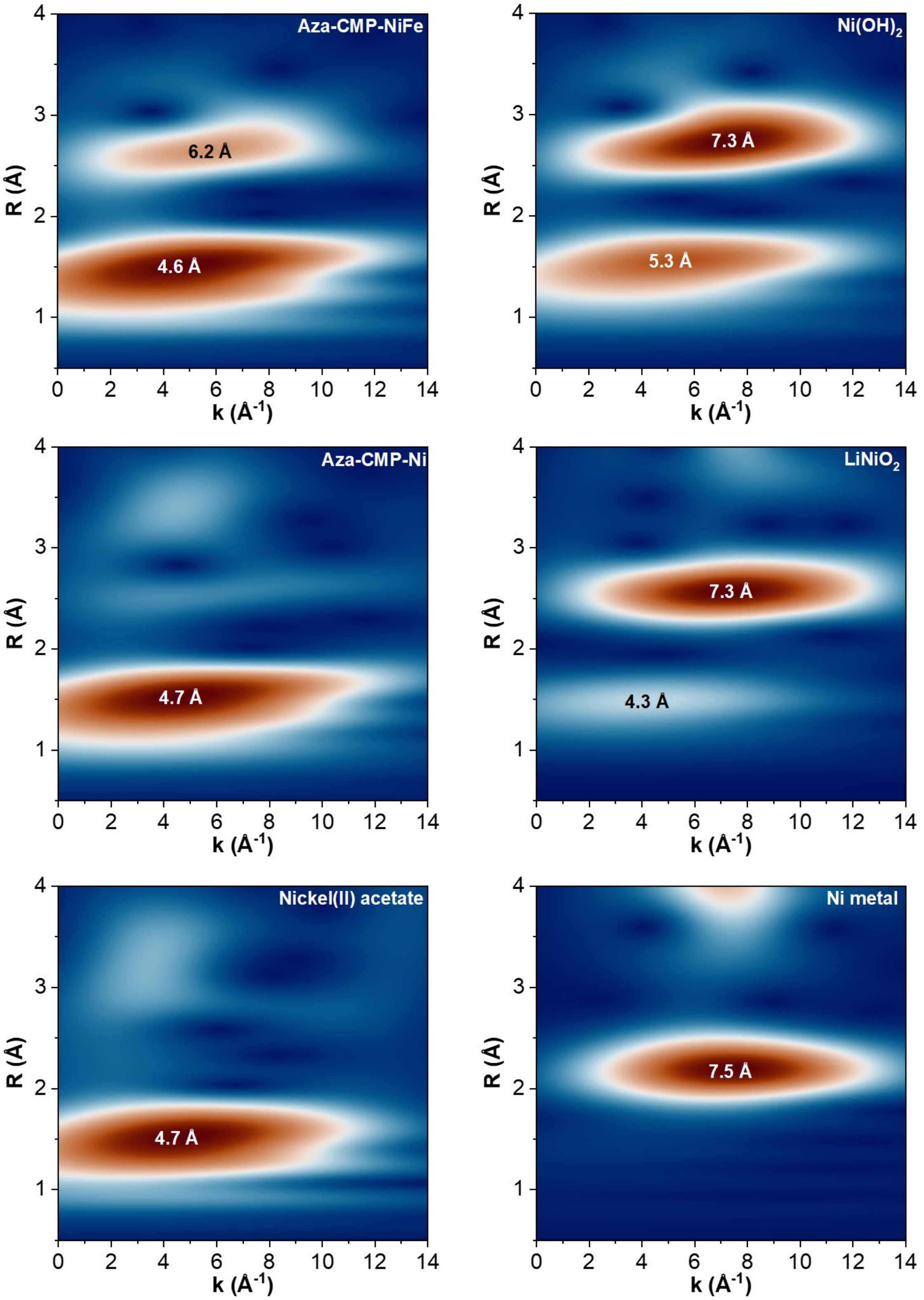

Supplementary Fig. 36 The wavelet transforms of Ni K-edge EXAFS spectra of Aza-CMP-Ni, Aza-CMP-NiFe, and reference materials.

Supplementary Discussion 3

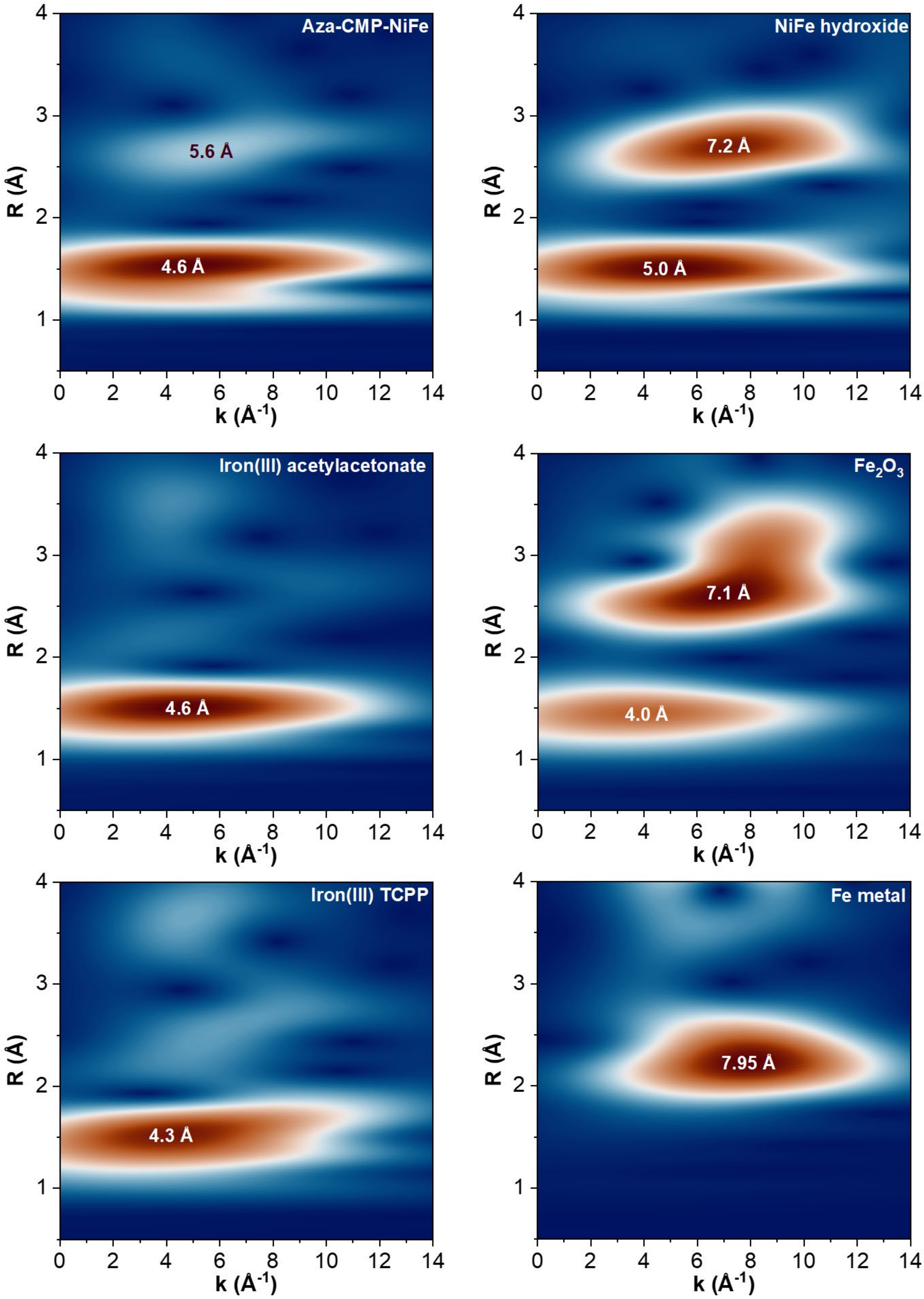

Supplementary Fig. 37 The wavelet transforms of Fe K-edge EXAFS spectra of Aza-CMP-NiFe and reference materials.

Supplementary Discussion 3

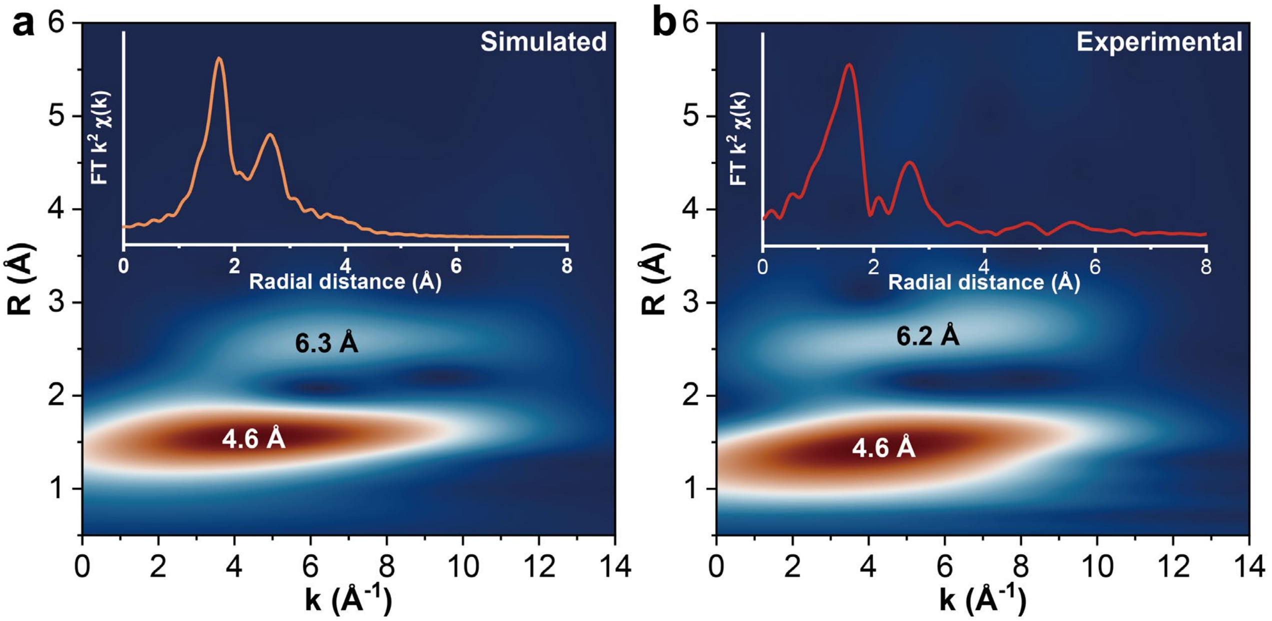

Supplementary Fig. 38 Wavelet transform simulation. (a) The wavelet transforms of the simulated Ni K-edge EXAFS spectrum. Inset: simulated EXAFS curve in R space. (b) The wavelet transforms of the experimental Ni K-edge EXAFS spectrum. Inset: experimental EXAFS curve in R space.

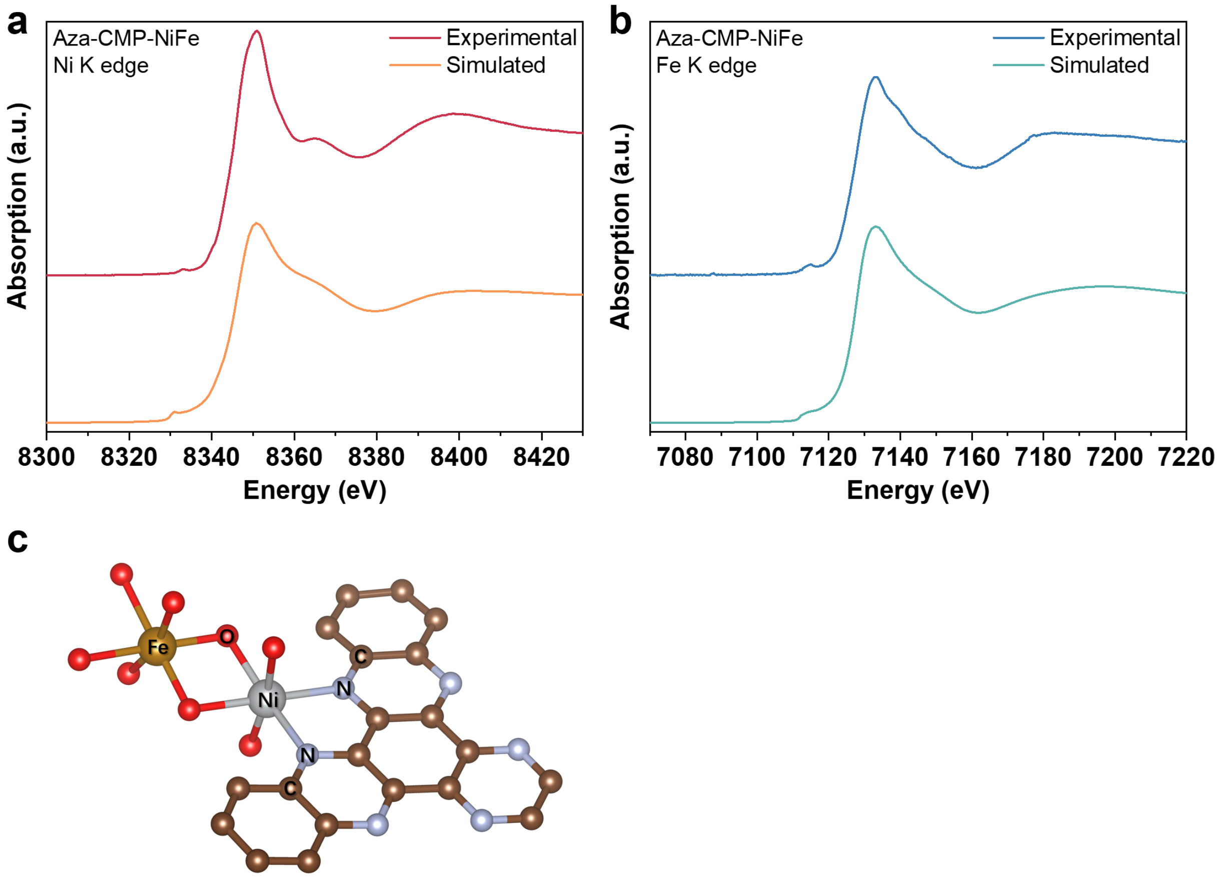

Supplementary Fig. 39 XANES spectra simulation. Comparison between the simulated and experimental (a) Ni K-edge and (b) Fe K-edge XANES spectra of Aza-CMP-NiFe. (c) DFT model of the Aza-CMP-NiFe for FDMNES[6] calculation.

Supplementary Discussion 3

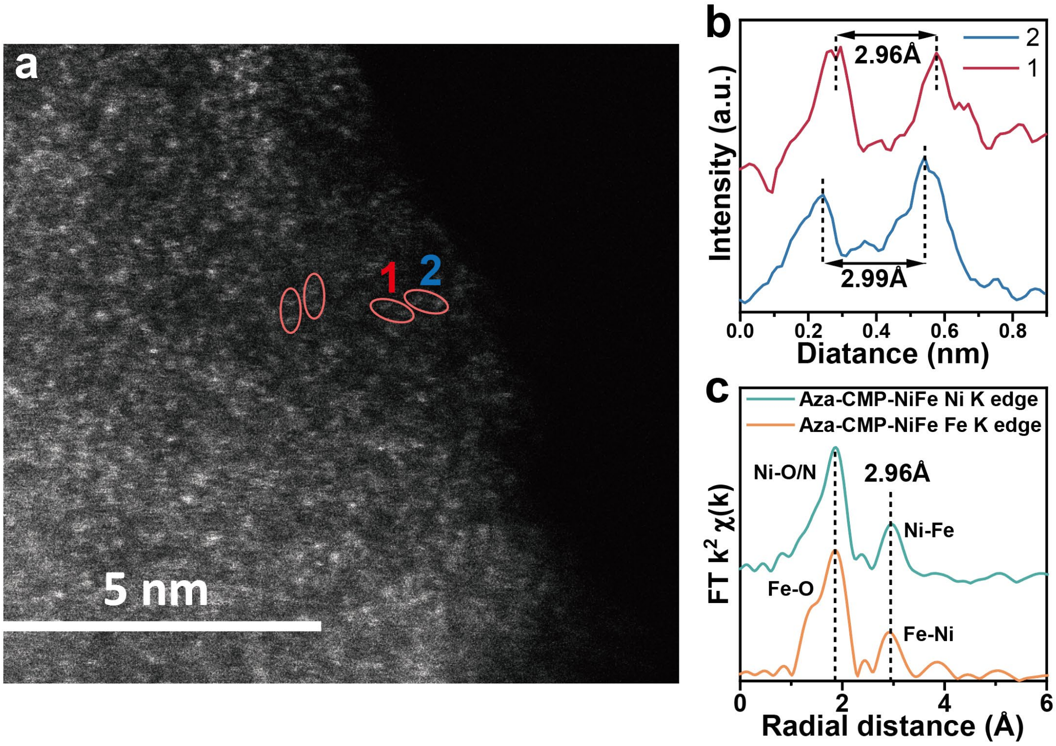

Supplementary Fig. 40 STEM characterization. (a) Atomic-resolution STEM image of Aza-CMP-NiFe (red circles indicate dual-metal sites). (b) The radial distance of two metal centers in Ni-Fe sites from the STEM image. (c) k^2-weighted Ni and Fe K-edge EXAFS curves of Aza-CMP-NiFe with phase correction. The distance between Ni-Fe sites is approximately 2.96 Å.

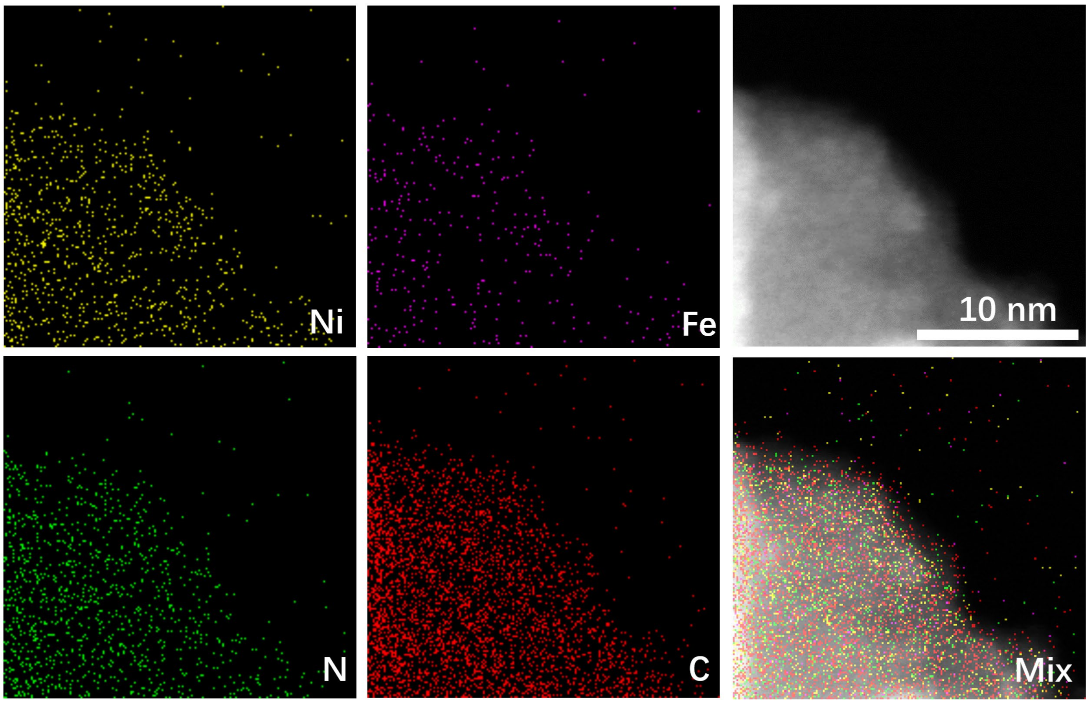

Supplementary Fig. 41 HAADF and corresponding elemental mapping images of Aza-CMP-NiFe.

Supplementary Discussion 3

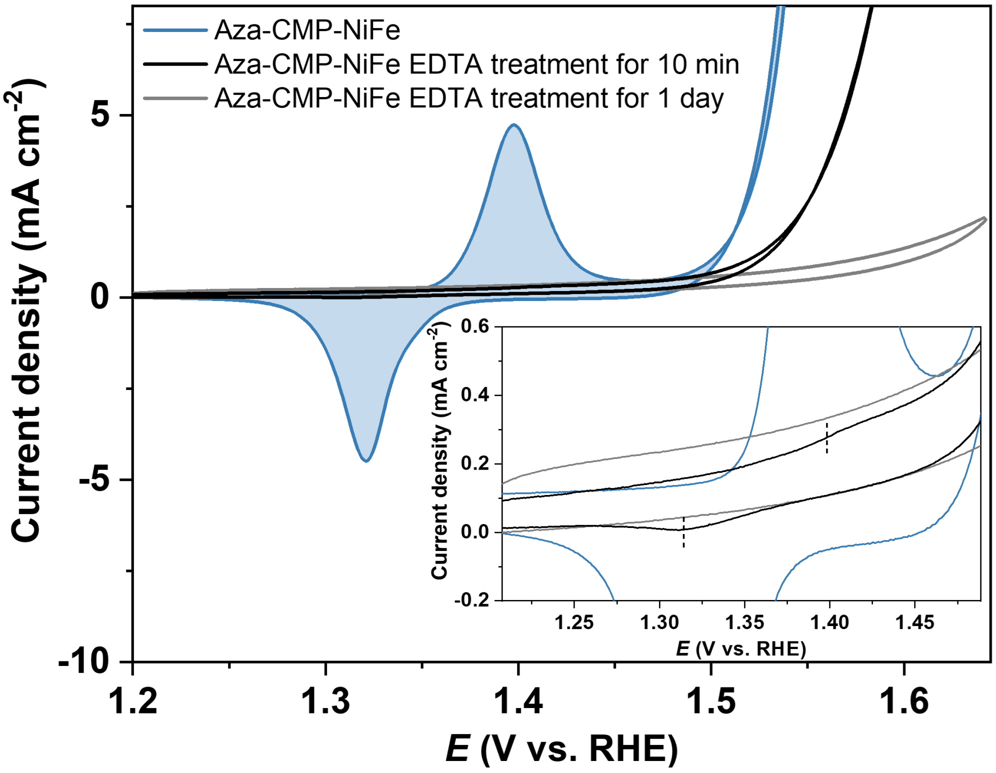

Supplementary Fig. 42 EDTA treatment. CV curves of Aza-CMP-NiFe/CP before and after being treated with 0.01 M Na-EDTA aqueous solution (1.0 M NaOH, 50 mV s^{-1} scan rate, without iR compensation). Inset: magnified figure of the redox region.

Supplementary Discussion 3

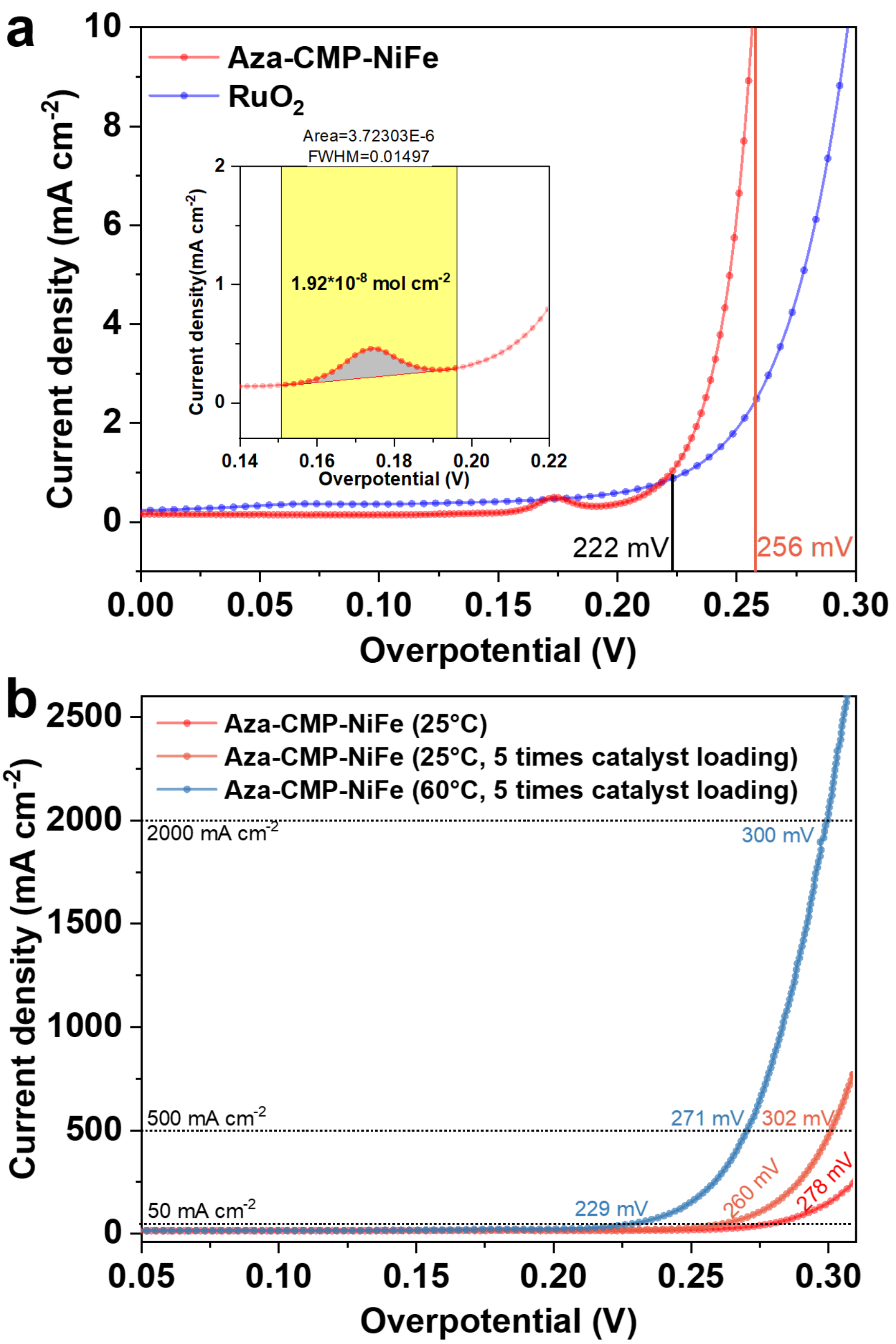

Supplementary Fig. 43 LSV curves of Aza-CMP-NiFe. (a) Zoomed LSV curves of Aza-CMP-NiFe and reference RuO_2 in 1.0 M KOH (scan rate: 1 mV s^{-1}). The surface concentration of redox-active Ni-Fe sites is calculated to be 1.92×10^{-8} mol cm^{-2} according to the $Ni^{2+/3+}$ oxidation peak charge. (b) LSV curves of Aza-CMP-NiFe with a fivefold increase in loading amount in 1.0 M KOH (scan rate: 1 mV s^{-1}).

Supplementary Discussion 3

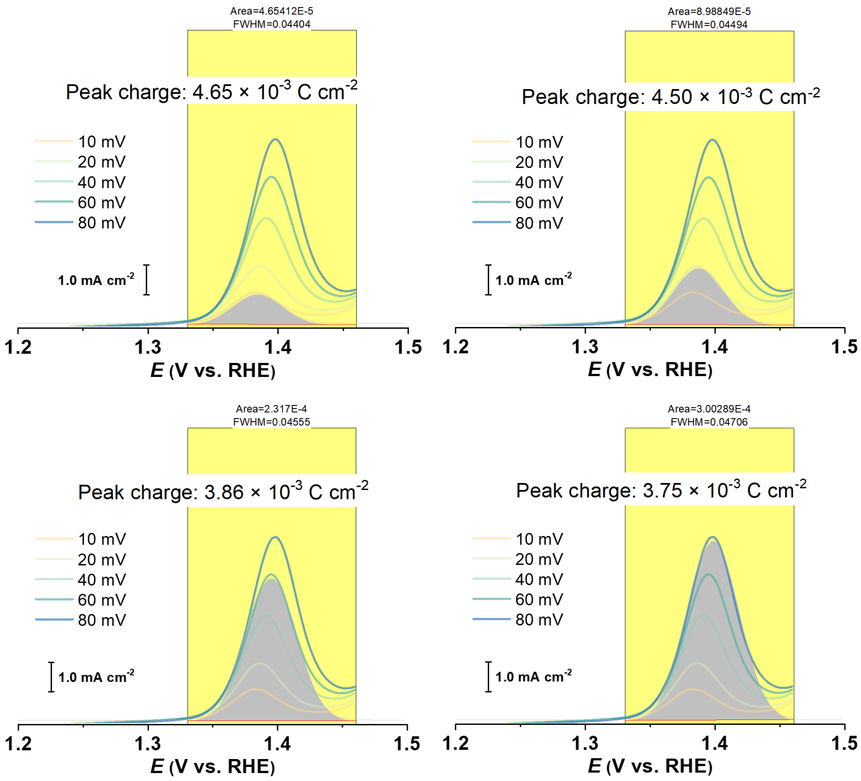

Charge transfer number **(total Ni content)**	0.44 – 0.55 e⁻ per Ni site
Charge transfer number **(total Fe content)**	1.74 – 2.13 e⁻ per Fe site
Charge transfer number **(total Ni-Fe content)**	0.87 – 1.07 e⁻ per metal site

Supplementary Fig. 44 The calculated loading of redox-active Ni-Fe sites via the charge of the oxidation peak at different scan rates in 1.0 M NaOH. The charge transfer can be calculated under different scenarios, depending on how the metal content is defined.

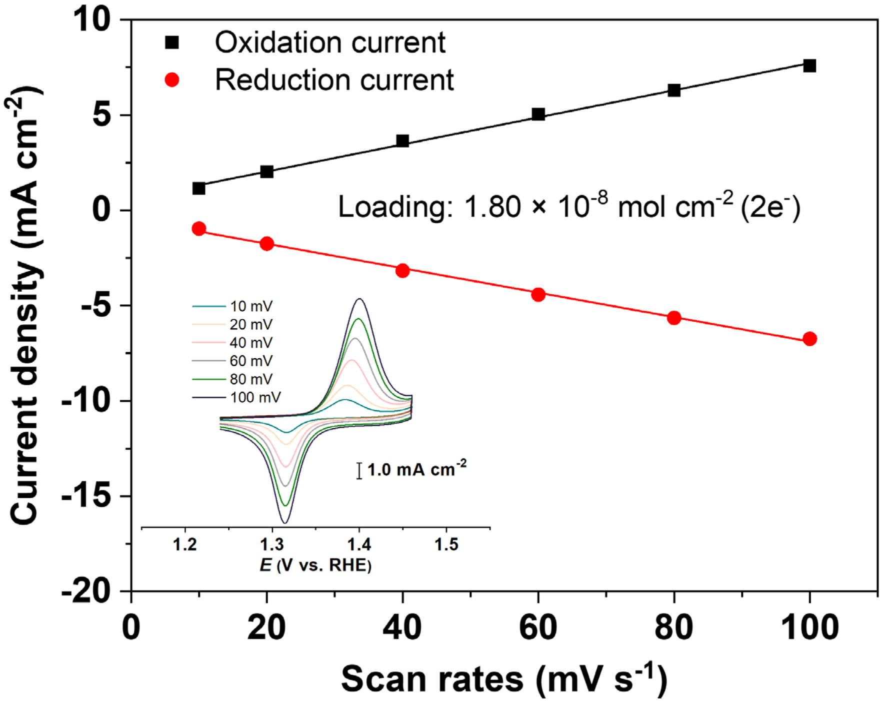

Supplementary Fig. 45 Dependence of peak current density on different scan rates. The calculated loading of redox-active Ni-Fe sites is 1.80 × 10^{-8} mol cm$^{-2}{}_{geo}$ according to the average slope values of the forward and backward scans (see ***Supplementary Eqns. 3*** and ***4*** for the calculation method). Inset: CV curves of Aza-CMP-NiFe/CP electrode at different scan rates in a 1.0 M NaOH solution.

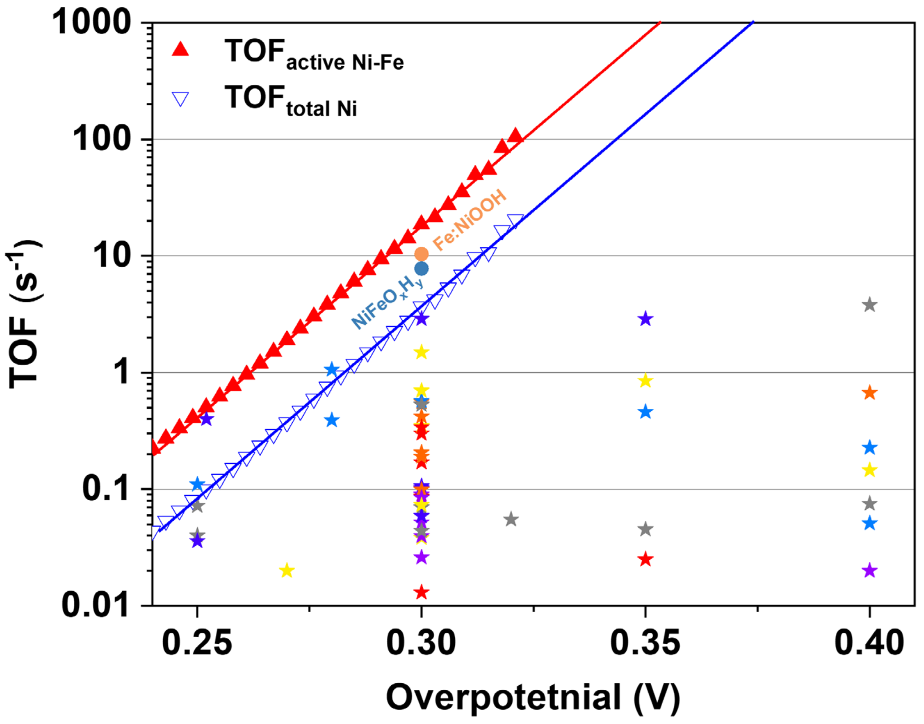

Supplementary Fig. 46 TOFs of Aza-CMP-NiFe based on the redox-active Ni-Fe sites, total Ni and Fe contents (1.0 M KOH) in comparison with selected state-of-the-art catalysts (represented by star-shaped markers). TOF values were calculated by ***Supplementary Eqn. 2***. Tabulated values of reference catalysts were retrieved from the literature.[23, 55, 56]

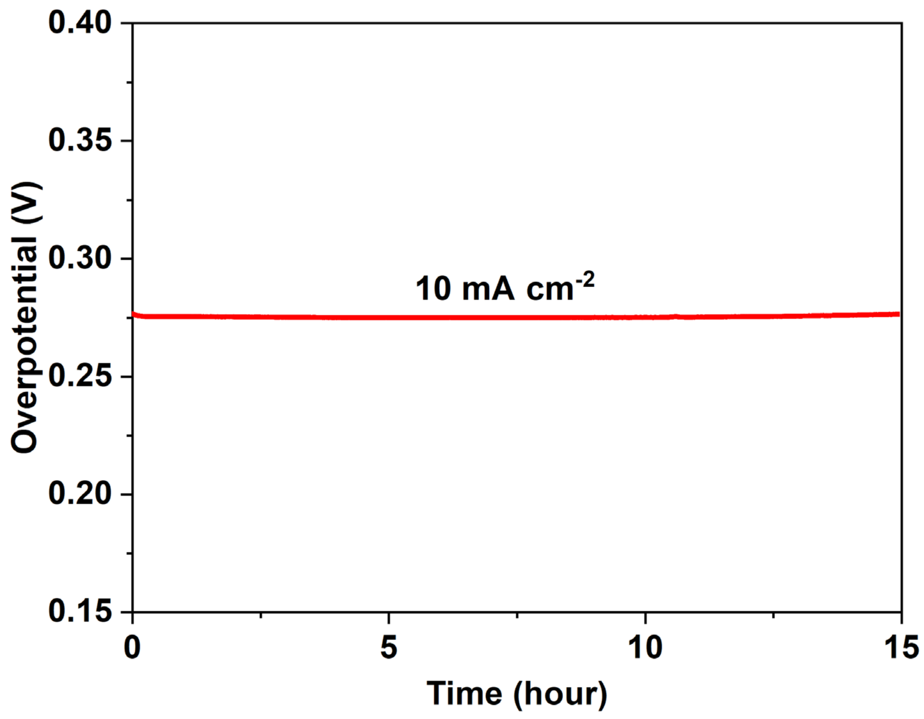

Supplementary Fig. 47 Catalytic stability test. Chronopotentiometry measurement of Aza-CMP-NiFe at a current density of 10 mA cm^{-2} for 15 hours in 1.0 M KOH.

Supplementary Discussion 3

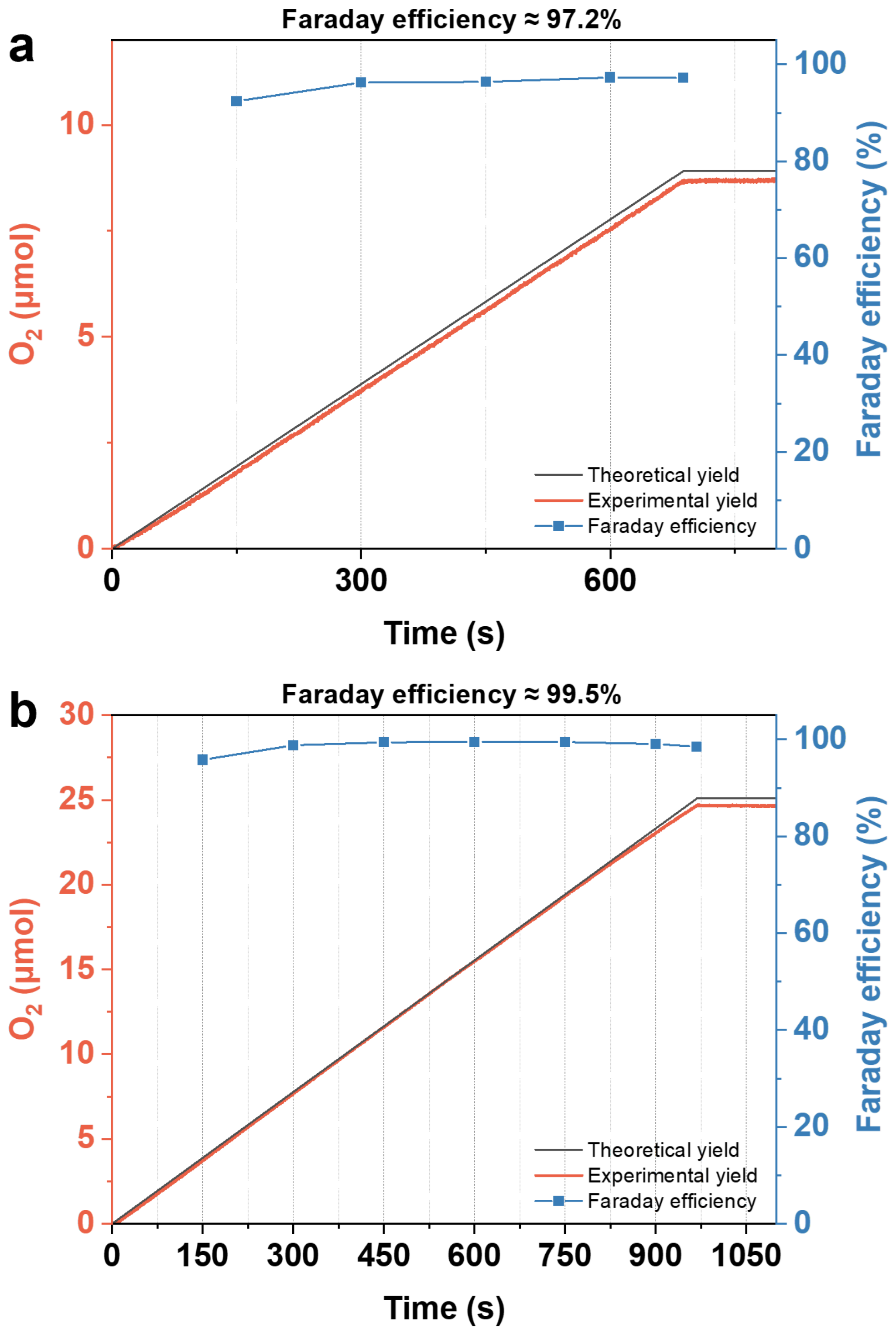

Supplementary Fig. 48 Faradaic efficiencies of Aza-CMP-NiFe/CP electrode for OER in 1.0 M KOH. Theoretically calculated and experimentally measured amounts of O_2 were shown as functions of electric charge for water oxidation at a current density of (a) 5 mA cm^{-2} and (b) 10 mA cm^{-2}.

Supplementary Discussion 4

Supplementary Discussion 4: Post-characterizations

The composition and electronic states of OER-tested electrodes were first analyzed using XPS. High-resolution spectra of the tested Aza-CMP-Ni/CP are consistent with those of the pristine sample, with no additional peaks observed in the Ni 2*p*, O 1*s*, and N 1*s* regions (***Supplementary Fig. 49***). Raman spectra for both pristine and tested Aza-CMP-Ni samples also indicate the absence of oxide phases after the OER since there is no clear change in characteristic peaks (***Supplementary Fig. 50***). The HAADF-STEM image in ***Supplementary Fig. 51*** suggests that most nickel atoms in the Aza-CMP maintain their atomic dispersion. However, the aggregation of the nickel component is also observed: nanoclusters with a diameter of less than 5 nm were generated in the selected area. The bright field TEM images of the thinner edge of the sample show the layered structure of Aza-CMP with a layered distance of 3.4 Å, and the existence of aggregated crystals is apparent (***Supplementary Fig. 52***). The aforementioned findings demonstrate that both XPS and Raman spectroscopy lack the sensitivity required to confirm minor structural alterations in catalysts. The golden standard to probe the single-site nature of the catalyst is to perform CVs after achieving relevant current density. As shown in ***Supplementary Fig. 53a***, the redox features and catalytic current density of Aza-CMP-Ni exhibit good reversibility following long-term CV operation (150 scans) in 1.0 M NaOH. However, the reduction peak becomes asymmetric after continuous electrolysis, signifying the evolution of redox-active species during operation. Given the sluggish kinetics requiring a relatively high operation voltage ($>$ 1.8 V vs. RHE) for Aza-CMP-Ni to achieve dominant current densities, the formation of nanosized oxide clusters is plausible and reasonable. Similar characterizations were applied to the tested Aza-CMP-NiFe/CP electrode. XPS spectra in the Ni 2*p*, Fe 2*p*, O 1*s*, and N 1*s* regions (***Supplementary Fig. 54***), along with the Raman spectrum (***Supplementary Fig. 55***) of the tested Aza-CMP-NiFe, are consistent with its pristine spectra. Concurrently, the redox features and catalytic current density of Aza-CMP-NiFe are preserved after 300 CV scans in 1.0 M NaOH electrolyte, demonstrating the robustness of Ni-Fe sites toward the OER under alkaline pH conditions (***Supplementary Fig. 53b***). Additionally, the HAADF-STEM image in ***Supplementary Fig. 56*** suggests maintaining an atomic-dispersed feature for metal sites in the Aza-CMP framework without aggregation; corresponding elemental mapping also confirmed the uniform distribution of Ni and Fe in Aza-CMP-NiFe after the OER (***Supplementary Fig. 57***).

The local structure evolution of catalysts during catalysis is further investigated using EXAFS. The EXAFS spectra of Aza-CMP-Ni/CP reveal no significant differences in the first shell after the sample was operated under catalytic conditions (***Supplementary Fig. 58a***). The minor shift observed in the second shell peak overlaps with the contribution from the CMP backbone and cannot be conclusively attributed to oxide formation, which supports the notion that EXAFS is insensitive to highly dispersed metal oxide species with nanosized particles.[57] XANES features, however, can provide enhanced sensitivity to the presence of minority species.[58] As demonstrated in ***Supplementary Fig. 59a***, the emergence of an additional peak indicates changes in electronic structures following catalysis. In fact, the amplitude of k-space EXAFS does exhibit a slight alteration for the tested Aza-CMP-Ni/CP sample (***Supplementary Fig. 59b***). WT of EXAFS data enables the separation of signal contributions into their k and R-space representations, thereby facilitating the identification of newly generated species during catalysis. As shown in ***Supplementary Fig. 58b***, WTs of pristine and tested samples are compared with reference materials $Ni(OH)_2$ and $LiNiO_2$. The intensity maxima in the second shell evolve as catalysis progresses, with the newly generated peaks at approximately 8 $Å^{-1}$ being consistent with the features of the M–O–M structure in $Ni^{2+}(OH)_2$ rather than those of $LiNi^{3+}O_2$. The WT results support the conclusion that atomic dispersion of Ni sites gradually aggregates to form

a (hydro)oxide phase during catalysis. For Aza-CMP-NiFe, the comparison of Fe and Ni K-edge EXAFS results is illustrated in ***Supplementary Fig. 60***. Neither the R space spectra of the Ni edge nor the Fe edge exhibit discernible changes in the first and second shells. Notably, the WTs of Ni and Fe sites before and after catalysis maintain nearly identical intensity contours, suggesting that the catalytic process has not significantly impacted the coordination environment. Meanwhile, the XANES of both the Ni edge (***Supplementary Fig. 61a***) and the Fe edge (***Supplementary Fig. 62a***) display no substantial alterations after the OER. The k-space EXAFS amplitudes of the tested samples retain all the features present in the pristine samples (***Supplementary Figs. 61b*** and ***62b***).

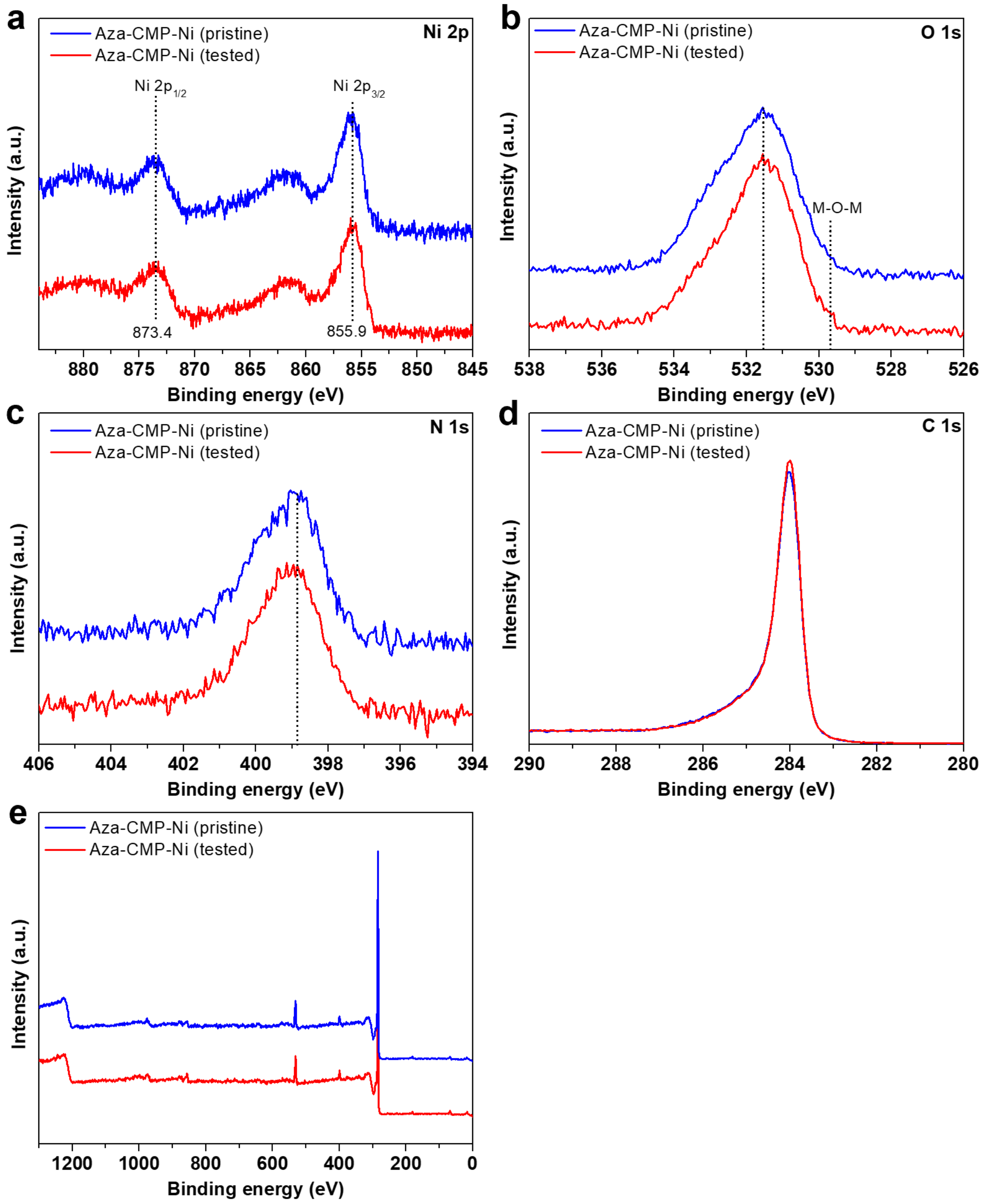

Supplementary Fig. 49 High-resolution XPS spectra of pristine and tested Aza-CMP-Ni/CP. (a) Ni 2*p*, (b) O 1*s*, (c) N 1*s*, (d) C 1*s*, and (e) survey regions.

Supplementary Discussion 4

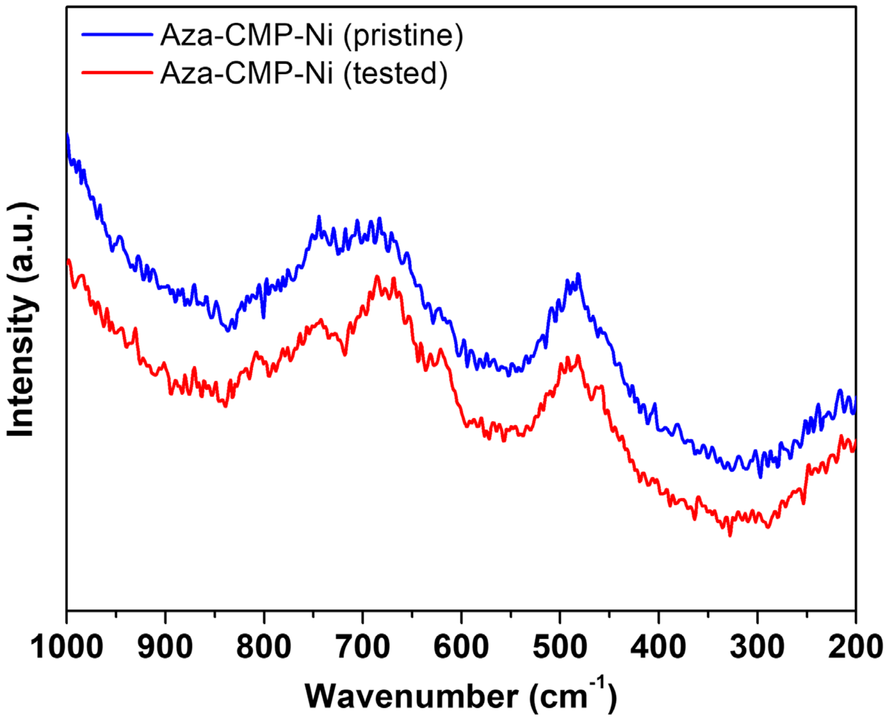

Supplementary Fig. 50 Raman spectra of pristine and tested Aza-CMP-Ni.

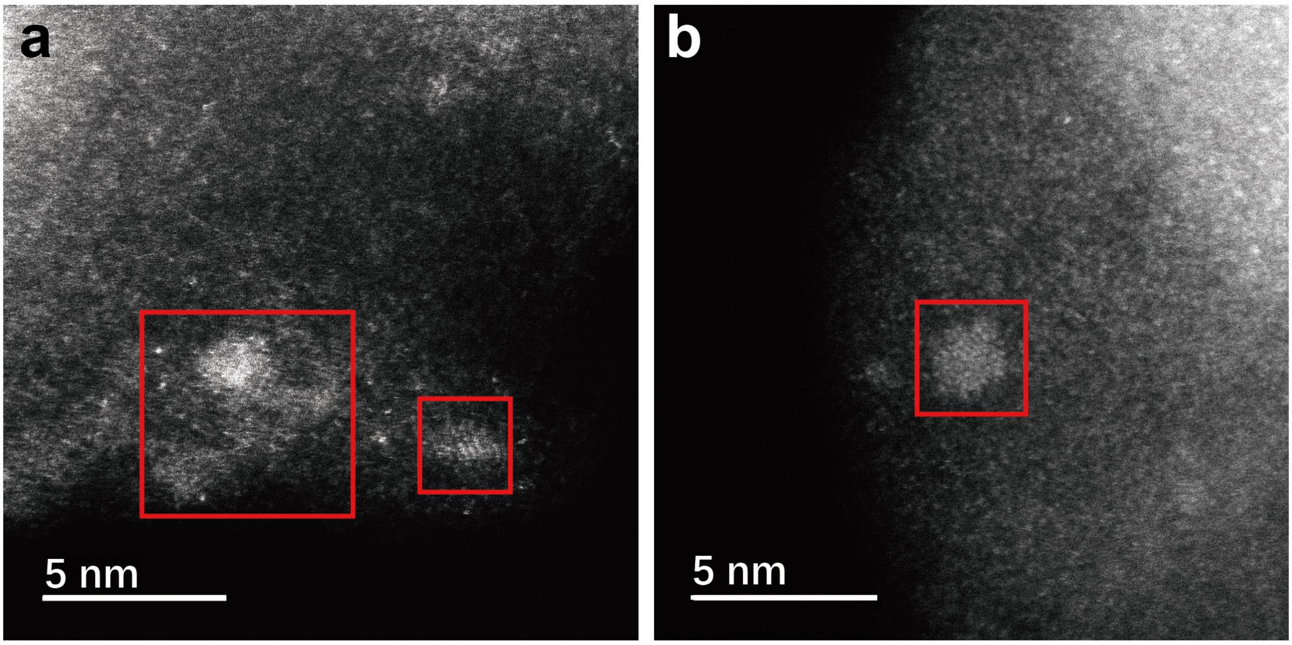

Supplementary Fig. 51 Atomic-resolution STEM images of tested Aza-CMP-Ni. Red rectangles indicate the aggregated clusters.

Supplementary Discussion 4

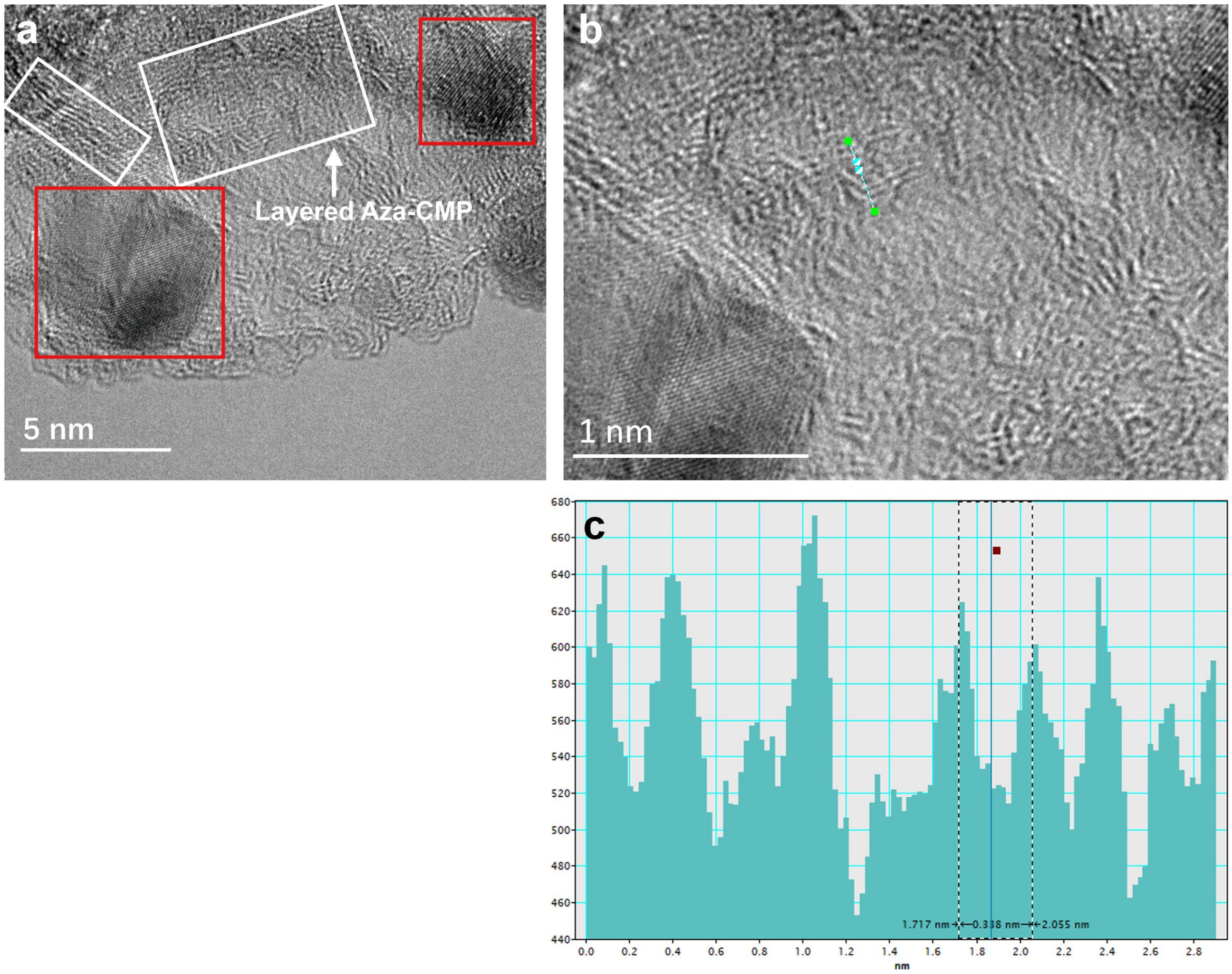

Supplementary Fig. 52 TEM characterization of tested Aza-CMP-Ni. (a-b) Bright-field TEM image of tested Aza-CMP-Ni (white rectangles indicate the layered Aza-CMP). (c) The corresponding intensity profile indicates the layered distance of Aza-CMP is around 3.4 Å.

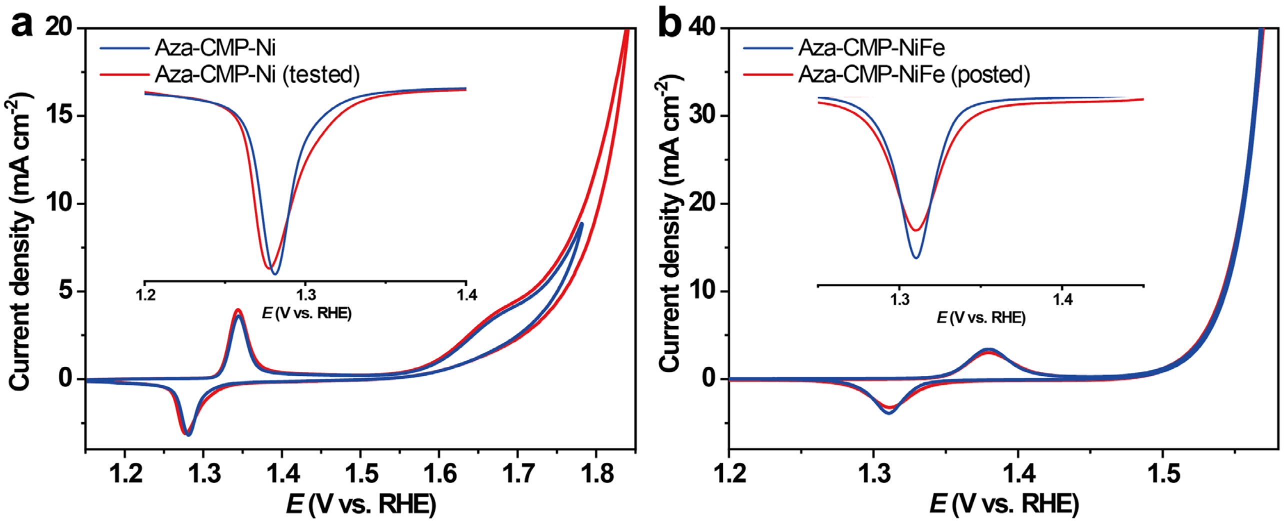

Supplementary Fig. 53 CV curves of pristine and tested samples. CV curves of (a) Aza-CMP-Ni and (b) Aza-CMP-NiFe were recorded in 1.0 M NaOH with a scan rate of 50 mV s^{-1}. Inset: zoomed figures in the reduction peak area.

Supplementary Discussion 4

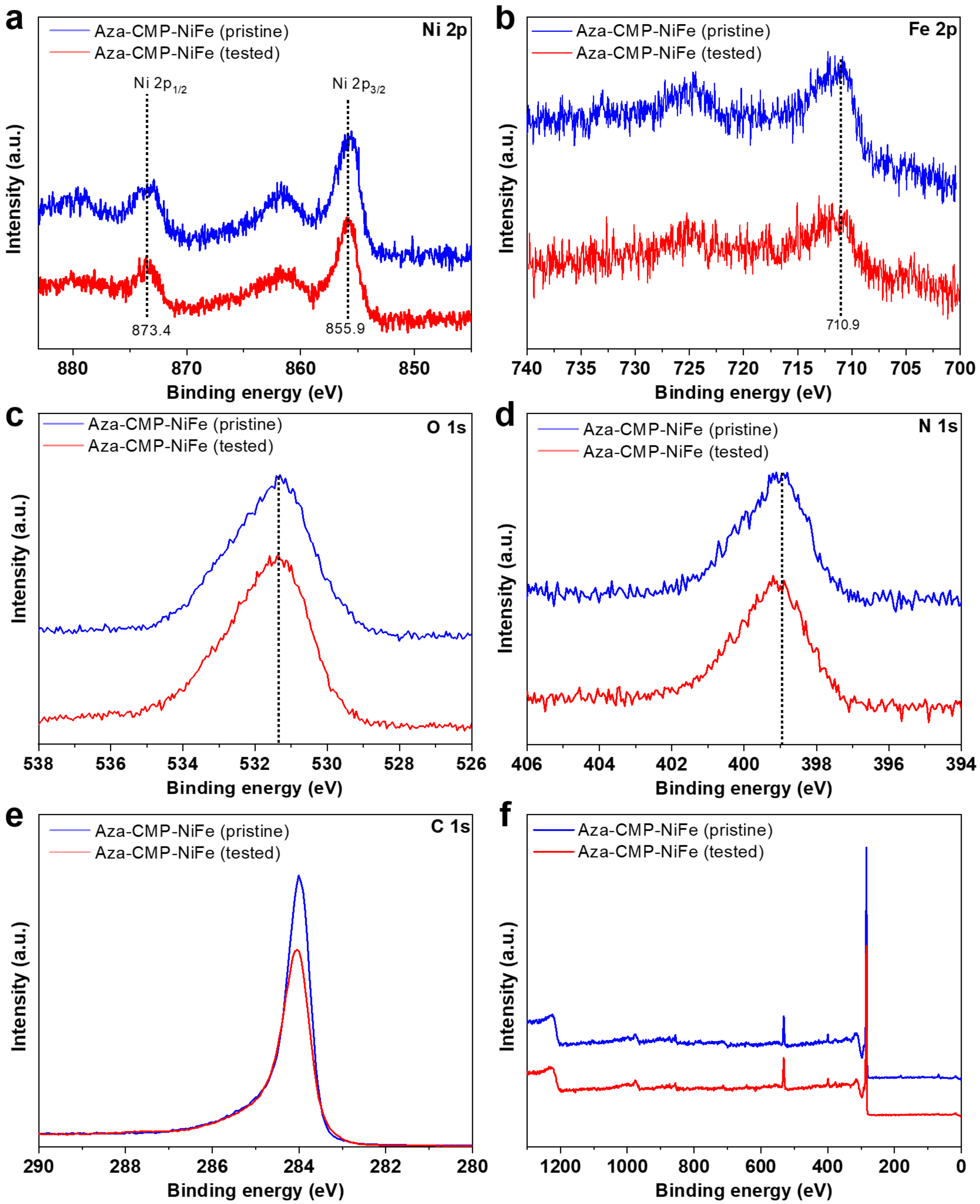

Supplementary Fig. 54 High-resolution XPS spectra of pristine and tested Aza-CMP-NiFe/CP. (a) Ni 2*p*, (b) Fe 2*p*, (c) O 1*s*, (d) N 1*s*, (e) C 1*s*, and (f) survey regions.

Supplementary Discussion 4

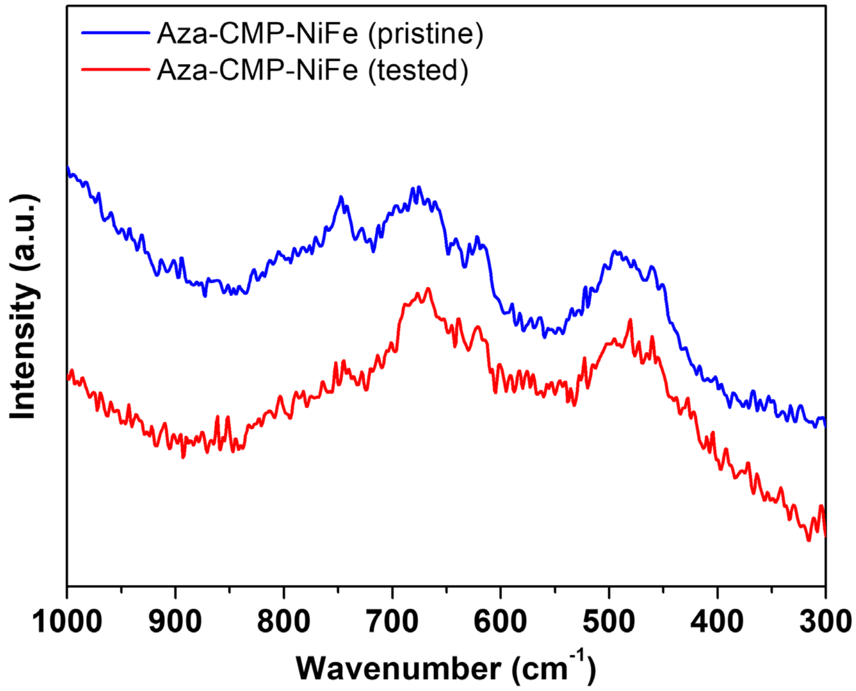

Supplementary Fig. 55 Raman spectra of pristine and tested Aza-CMP-NiFe.

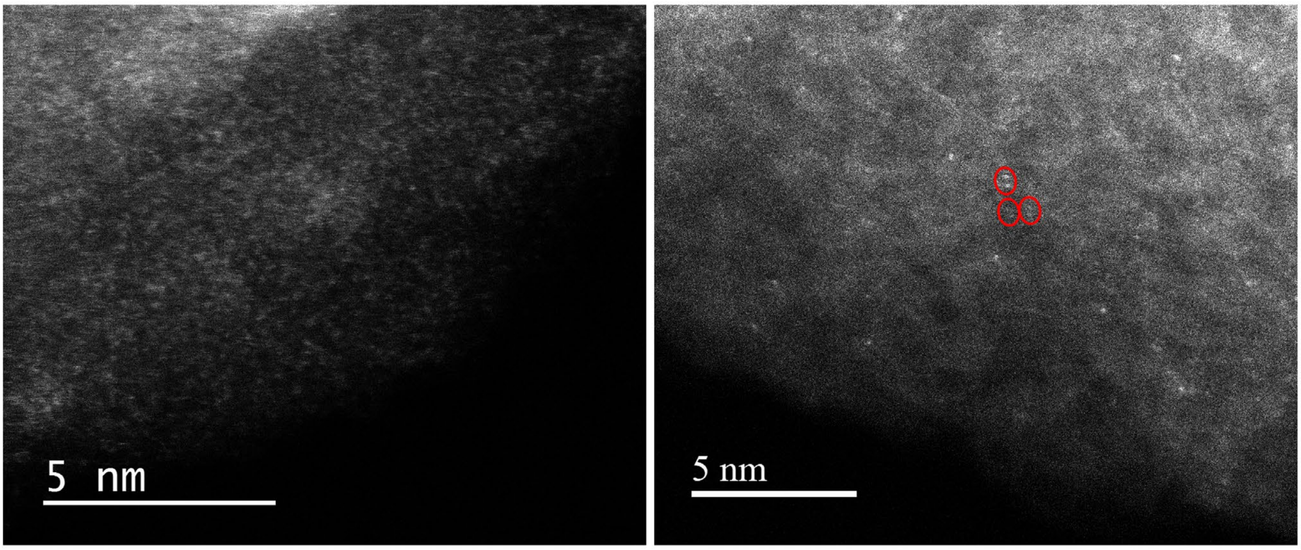

Supplementary Fig. 56 Atomic-resolution STEM image of tested Aza-CMP-NiFe. The red circles indicate the possible dual-metal sites. The result suggests that Ni-Fe sites in the Aza-CMP framework maintain atomic dispersion without aggregation.

Supplementary Discussion 4

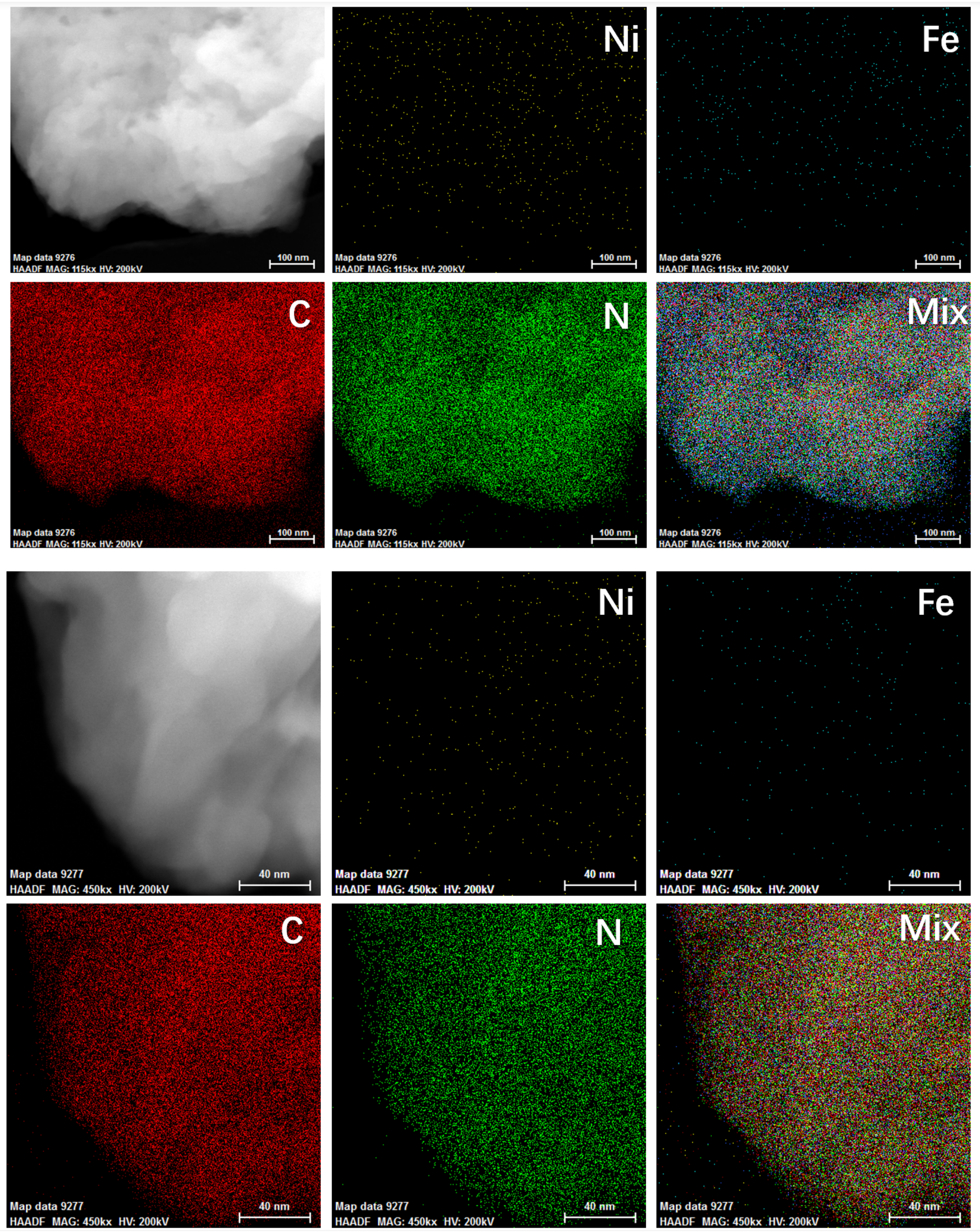

Supplementary Fig. 57 HAADF images and corresponding elemental mapping images of tested Aza-CMP-NiFe. The layer-by-layer Aza-CMP morphology is readily discernible, whereas the aggregated oxide morphology remains undetected.

Supplementary Discussion 4

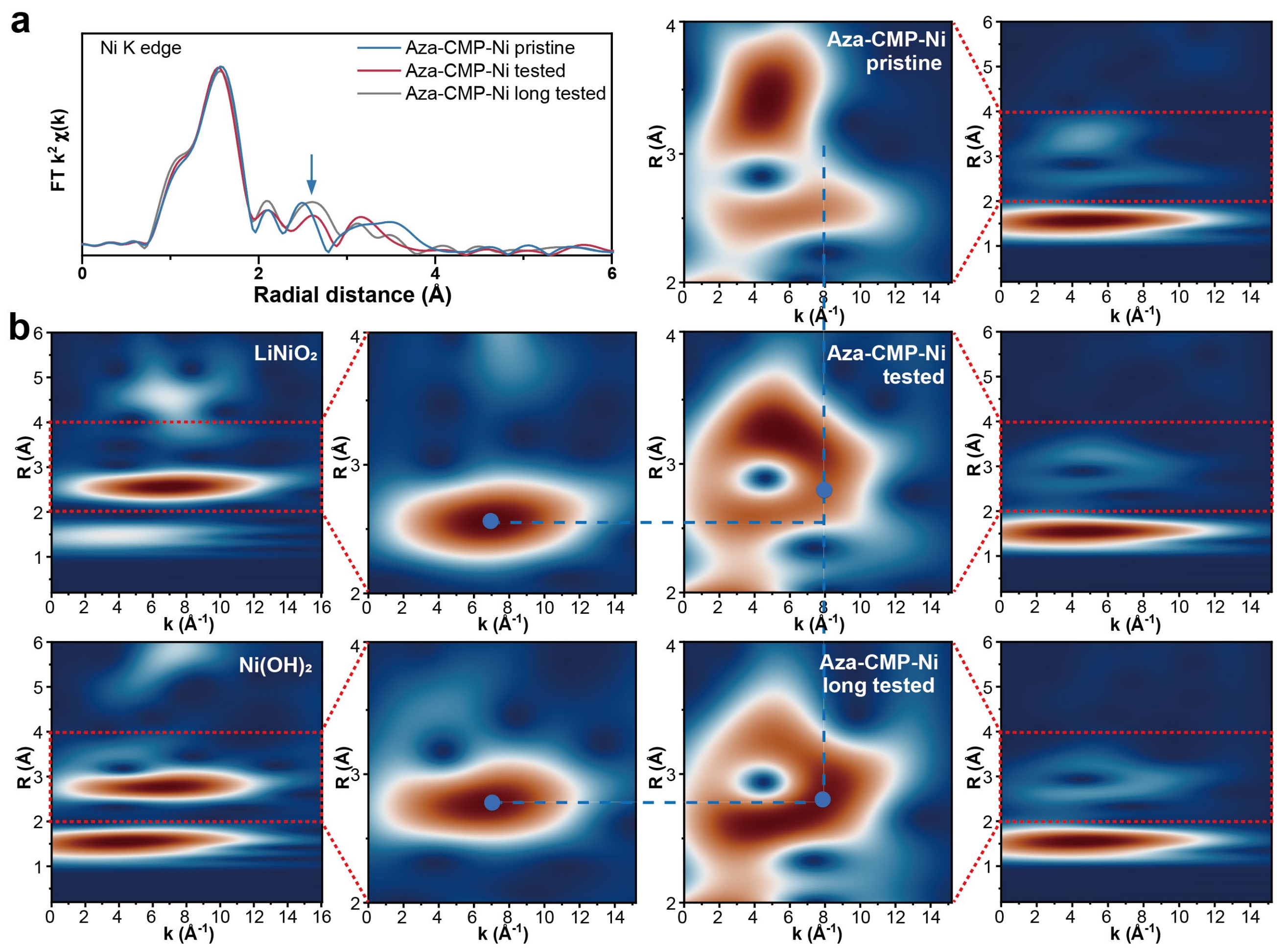

Supplementary Fig. 58 Ni K-edge Fourier transform EXAFS spectra of tested Aza-CMP-Ni samples. (a) Ni K edge EXAFS spectra and (b) the wavelet transforms of pristine, tested Aza-CMP-Ni, and reference materials. The 'tested' sample underwent 150 CV cycles, while the 'long tested' sample experienced 300 CV cycles in 1.0 M NaOH.

Supplementary Discussion 4

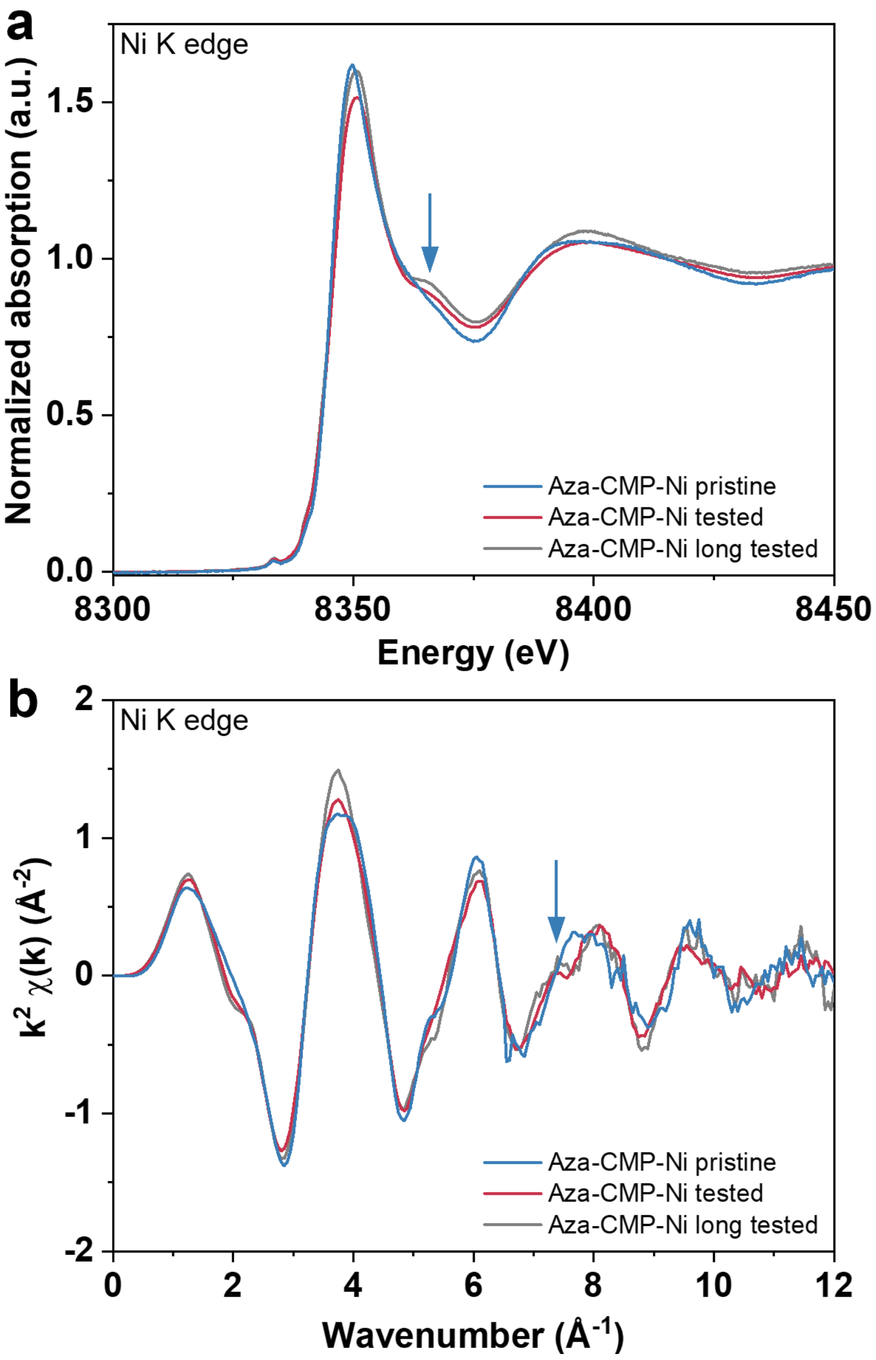

Supplementary Fig. 59 Ni K-edge XANES and EXAFS spectra of tested Aza-CMP-Ni samples. (a) Normalized Ni K-edge XANES spectra of pristine and tested Aza-CMP-Ni. (b) k^2-weighted Ni K-edge EXAFS spectra of Aza-CMP-Ni and tested Aza-CMP-Ni in k-space. Arrows signify alterations resulting from nanoparticle formation. The 'tested' sample underwent 150 CV cycles, while the 'long tested' sample experienced 300 CV cycles in 1.0 M NaOH.

Supplementary Discussion 4

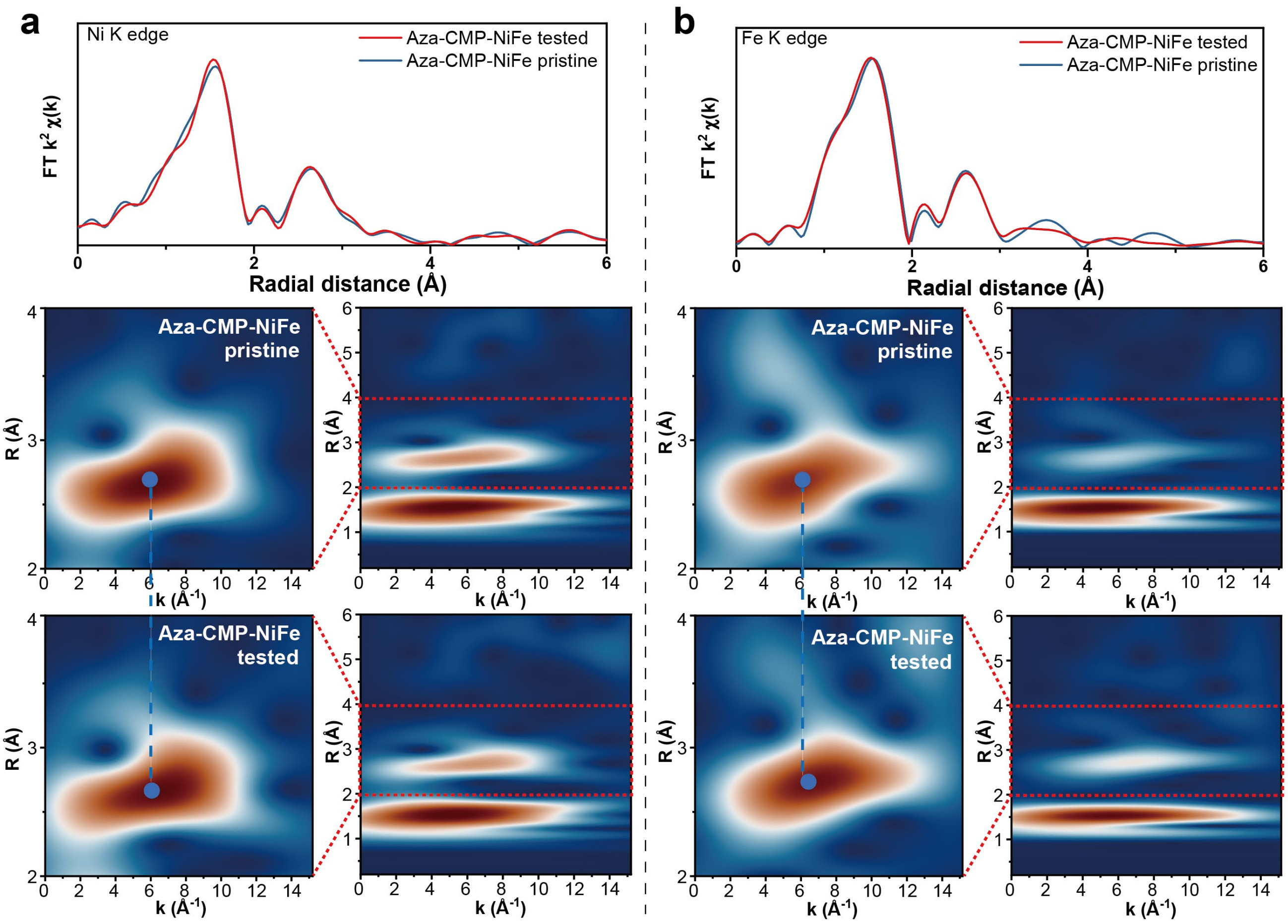

Supplementary Fig. 60 Fourier transform EXAFS spectra of tested Aza-CMP-NiFe samples. (a) Ni K edge EXAFS spectra and the wavelet transforms of pristine and tested Aza-CMP-NiFe (300 CV cycles). (b) Fe K edge EXAFS spectra and WTs of pristine and tested Aza-CMP-NiFe (300 CV cycles).

Supplementary Discussion 4

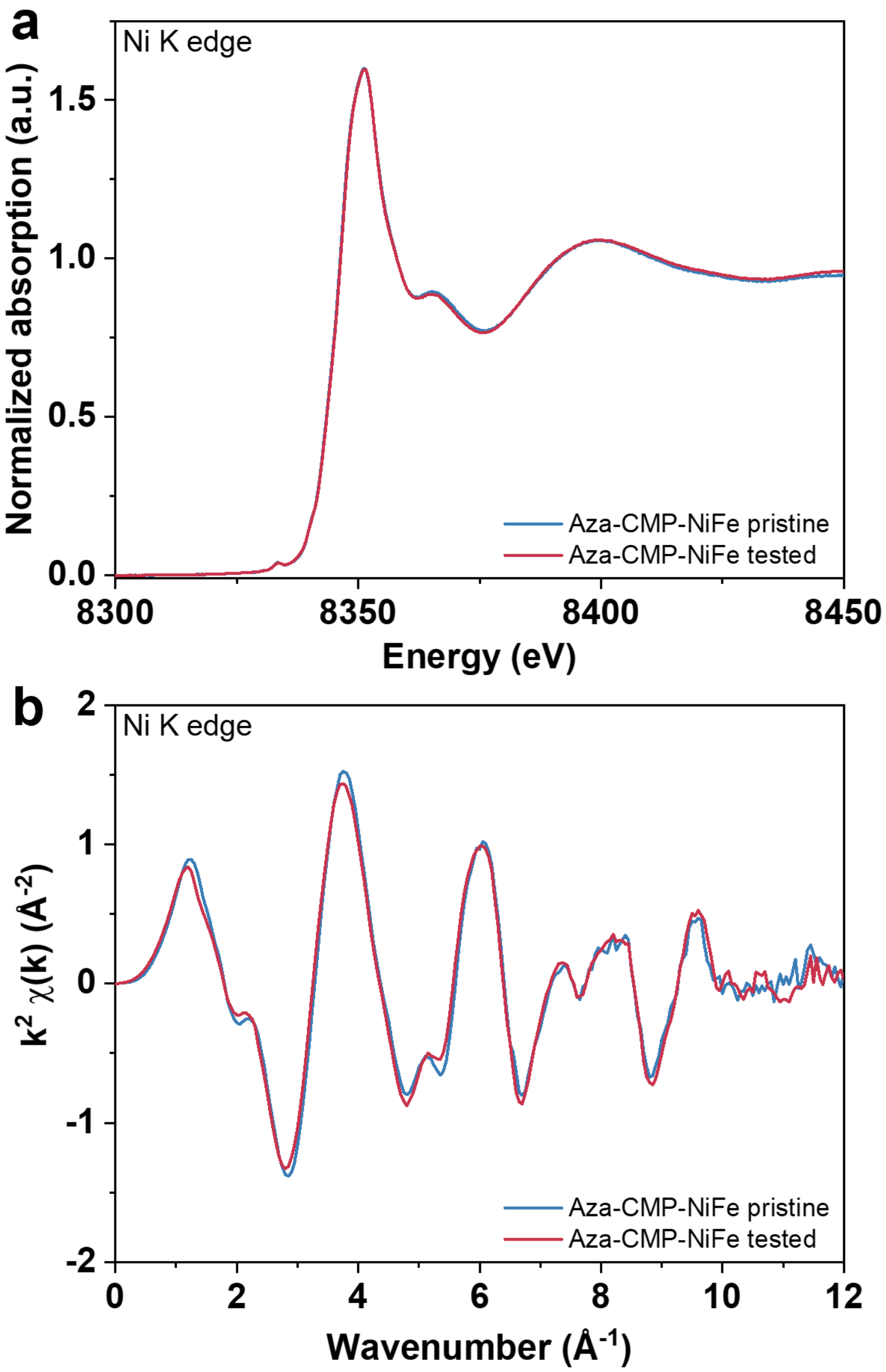

Supplementary Fig. 61 Ni K-edge XANES and EXAFS spectra of tested Aza-CMP-NiFe samples. (a) Normalized Ni K-edge XANES spectra of pristine and tested Aza-CMP-NiFe. (b) k^2-weighted Ni K-edge EXAFS spectra of pristine Aza-CMP-NiFe and tested Aza-CMP-NiFe in k-space. The tested sample underwent 300 CV cycles in 1.0 M NaOH.

Supplementary Discussion 4

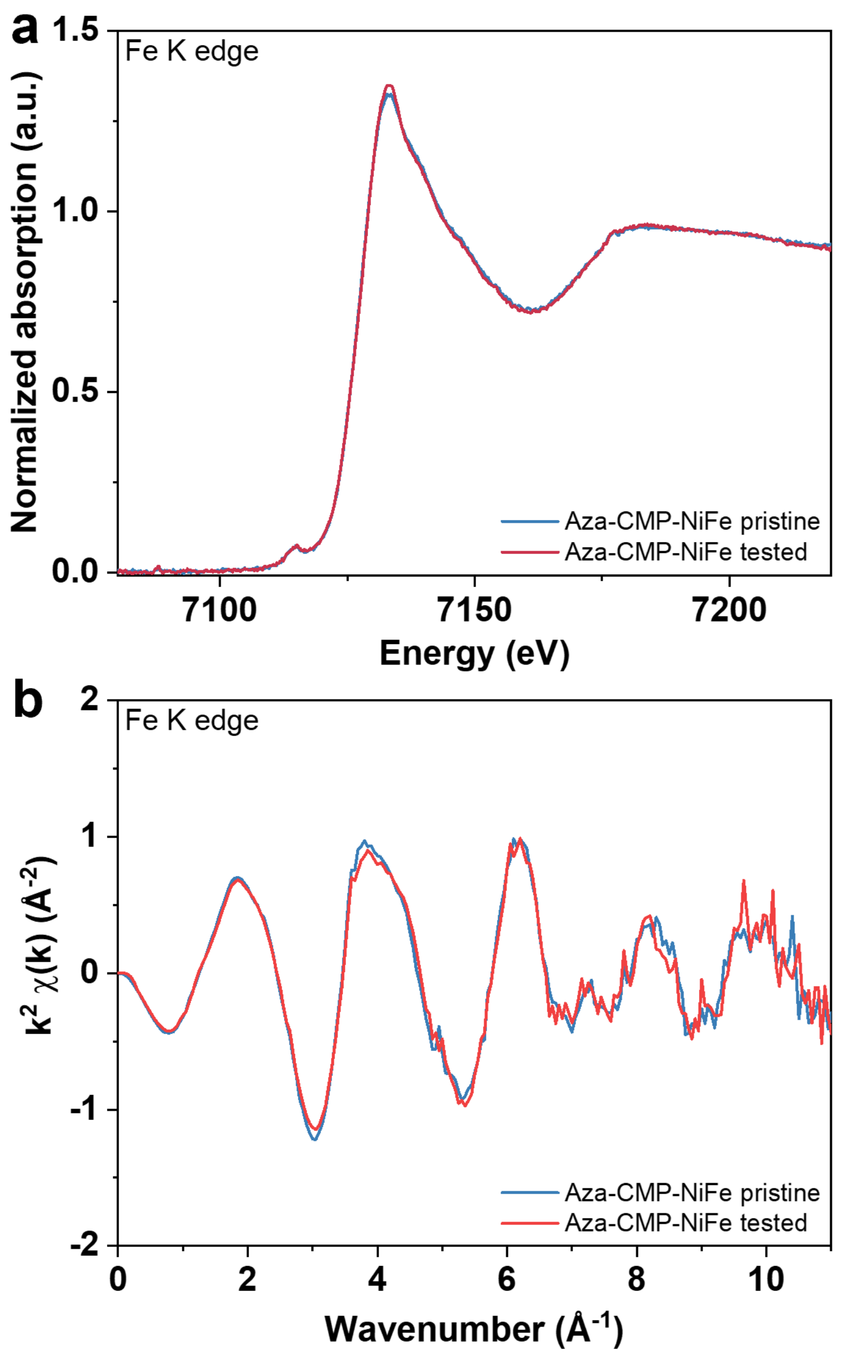

Supplementary Fig. 62 Fe K-edge XANES and EXAFS spectra of tested Aza-CMP-NiFe samples. (a) Normalized Fe K-edge XANES spectra of pristine and tested Aza-CMP-NiFe. (b) k^2-weighted Fe K-edge EXAFS spectra of pristine Aza-CMP-NiFe and tested Aza-CMP-NiFe in k-space. The tested sample underwent 300 CV cycles in 1.0 M NaOH.

Supplementary Discussion 5

Supplementary Discussion 5: Spectral detection of high-valent species.

5.1 XANES

The valence states of metal centers during electrochemical cycling were evaluated by *operando* XANES spectra. The oxidation states of metal elements were determined based on energy shifts in their K-edge absorption peaks. To establish a reference, a standard linear correlation between oxidation state and absorption edge energy was first constructed using reference samples with known oxidation states. The average oxidation state of the sample was then calculated from its absorption edge energy, defined as the energy at 0.5 normalized absorption. For the Ni edge, as shown in ***Supplementary Fig. 63a***, a clear linear relationship was observed between oxidation state and absorption edge energy, where a one-unit change in oxidation state corresponded to a 1.57 eV shift in edge position. This trend aligns well with the findings of Gonzalez-Flores and Dau et al., who reported a 1.6 eV shift per oxidation state unit.[59] In ***Supplementary Fig. 63b***, the Ni K-edge XANES spectra of Aza-CMP-Ni exhibit a notable shift of 1.6 eV during oxidation. Based on energy calibration with standard reference materials, this shift corresponds to a transition of the Ni center from +2.2 to +3.2 oxidation state (***Supplementary Table 2***). In contrast, the Ni K-edge XANES spectra of Aza-CMP-NiFe exhibit a smaller shift of 1.0 eV during oxidation, indicating a transition from +2.2 to +2.5 (***Supplementary Fig. 64a***). This suggests that Ni^{4+} species is not present in the Ni-Fe system. In addition, comparison with reference materials and the pre-edge shifts observed at different water oxidation potentials further indicate the formation of Ni^{3+} and the absence of Ni^{4+} species (***Supplementary Fig. 64b*** and ***64c***).

Supplementary Fig. 65a presents the Fe K-edge spectra of Aza-CMP-NiFe at different applied potentials. Due to the low Fe loading (~1 wt%), acquiring high-quality spectra under in situ conditions remains challenging. Under OER conditions (1.7 V vs. RHE), the Fe K-edge absorption edge shifts to higher energy compared to spectra recorded at non-catalytic potentials (open-circuit potential and 1.2 V vs. RHE). A comparison between the Fe K-edge spectra of powdered samples and those obtained at 1.2 V reveals good overlap in the pre-edge region, indicating that pre-edge peak identification remains feasible despite the lower signal quality under in situ conditions (***Supplementary Fig. 65b***). Under OER conditions, the Fe pre-edge peak shifts to higher energy, accompanied by increased peak width and intensity. These spectral changes suggest modifications in the electronic structure of outer Fe 3*d* orbitals. The pre-edge peak intensity suggests that the Fe site maintains an octahedral, six-coordinate environment. Additionally, the Fe pre-edge peak observed under OER conditions closely resembles the XANES spectra of molecular Fe^{4+} species in the literature.[60, 61, 62] The observed shifts in the pre-edge peak and white-line energy provide evidence supporting the potential formation of oxidized Fe species (Fe^{4+}–O) during OER.

In contrast to Ni compounds, the relationship between the oxidation state of Fe compounds and absorption edge energies is non-linear, as presented in ***Supplementary Fig. 66a***. This deviation would be attributed to the significant influence of the ligand environment, which introduces substantial variations in absorption energy even among Fe compounds with the same oxidation state. To further examine this non-linearity, an integration method was also applied to estimate the edge energy. The absorption spectrum was integrated up to the energy at which the normalized absorption first reaches 1, yielding the total integrated absorption. The edge position was then defined as the energy at which this integral reaches 90% of its maximum value.[63] Using this method, a non-linear relationship for the Fe K-edge was again observed (***Supplementary Fig. 66b***).

To determine the oxidation state of Fe in Aza-CMP-NiFe, the absorption edge energies of two Fe^{2+} and five Fe^{3+} reference compounds were averaged to construct a correlation curve. Assuming a linear relationship between oxidation states +2 and +3, the calculated Fe oxidation states at 1.2 V and

Supplementary Discussion 5

1.7 V vs. RHE are +3.14 and +3.43, respectively (***Supplementary Table 3*** and ***Supplementary Fig. 67a***). However, when a non-linear correlation (fitted with a Boltzmann function) spanning oxidation states +2 to +6 was applied, these values increased to +3.31 and +3.96, respectively. A similar trend was observed using the integration method: under a linear assumption, Fe oxidation states at 1.2 V and 1.7 V vs. RHE were calculated as +2.87 and +3.17, but increased to +2.90 and +3.65 when a non-linear relationship was applied (***Supplementary Table 4***, ***Supplementary Figs. 67b*** and ***67c***).

These findings indicate that using a linear correlation from Fe^{2+} to Fe^{3+} to estimate Fe^{4+} oxidation states is unreliable and likely results in underestimation. Furthermore, Fe^{4+} XANES data from previous publications suggest that the absorption edge's energy difference between Fe^{2+} and Fe^{4+} species (with similar ligand environments) is approximately 2-4 eV, significantly smaller than the 3.0-3.6 eV per oxidation state unit predicted by the linear model. The above discussions suggest that Fe oxidation states derived from linear extrapolation, both in this study and in previous reports, are likely underestimated. This underestimation may partially explain the ongoing challenges in unambiguously detecting Fe^{4+} species using in situ XANES techniques in electrocatalysis.

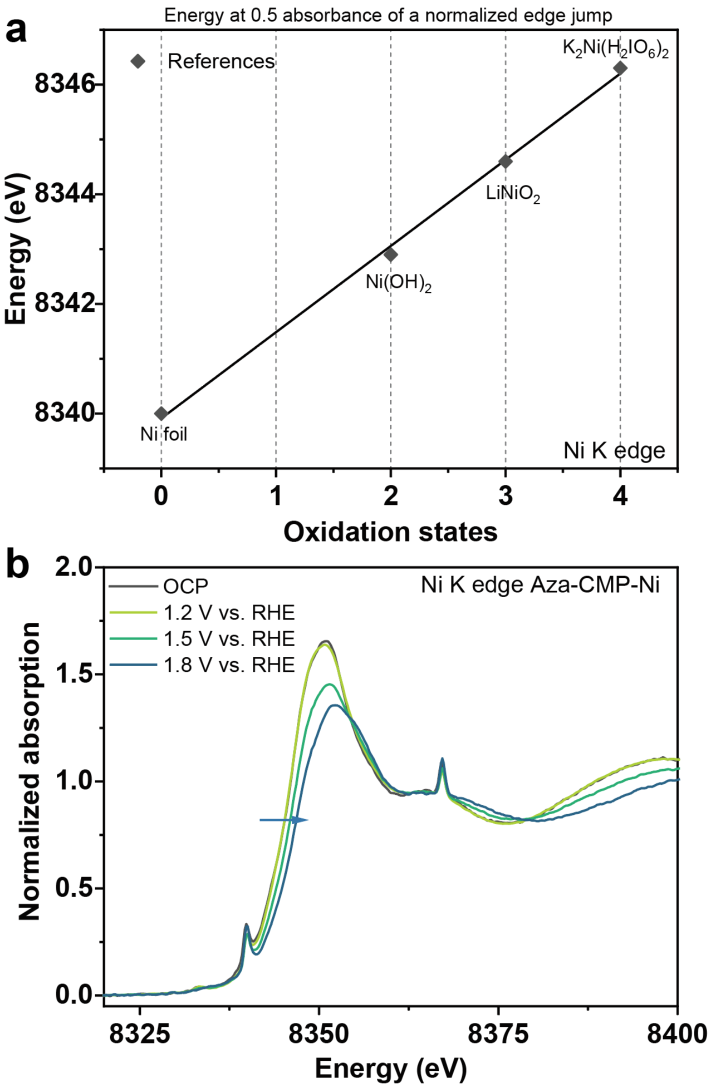

Supplementary Fig. 63 *Operando* Ni K-edge XANES spectra. (a) Edge-jump energies (at 0.5 absorbance) of Ni K-edge XANES spectra for reference materials with different oxidation states. (b) Aza-CMP-Ni under different potentials. As the potential further increases, the edge position shifts to higher energies, consistent with the oxidation of the nickel center to a higher valence. The glitch peaks at 8338 eV and 8366 eV were generated from background light due to a weak sampling signal.

Supplementary Discussion 5

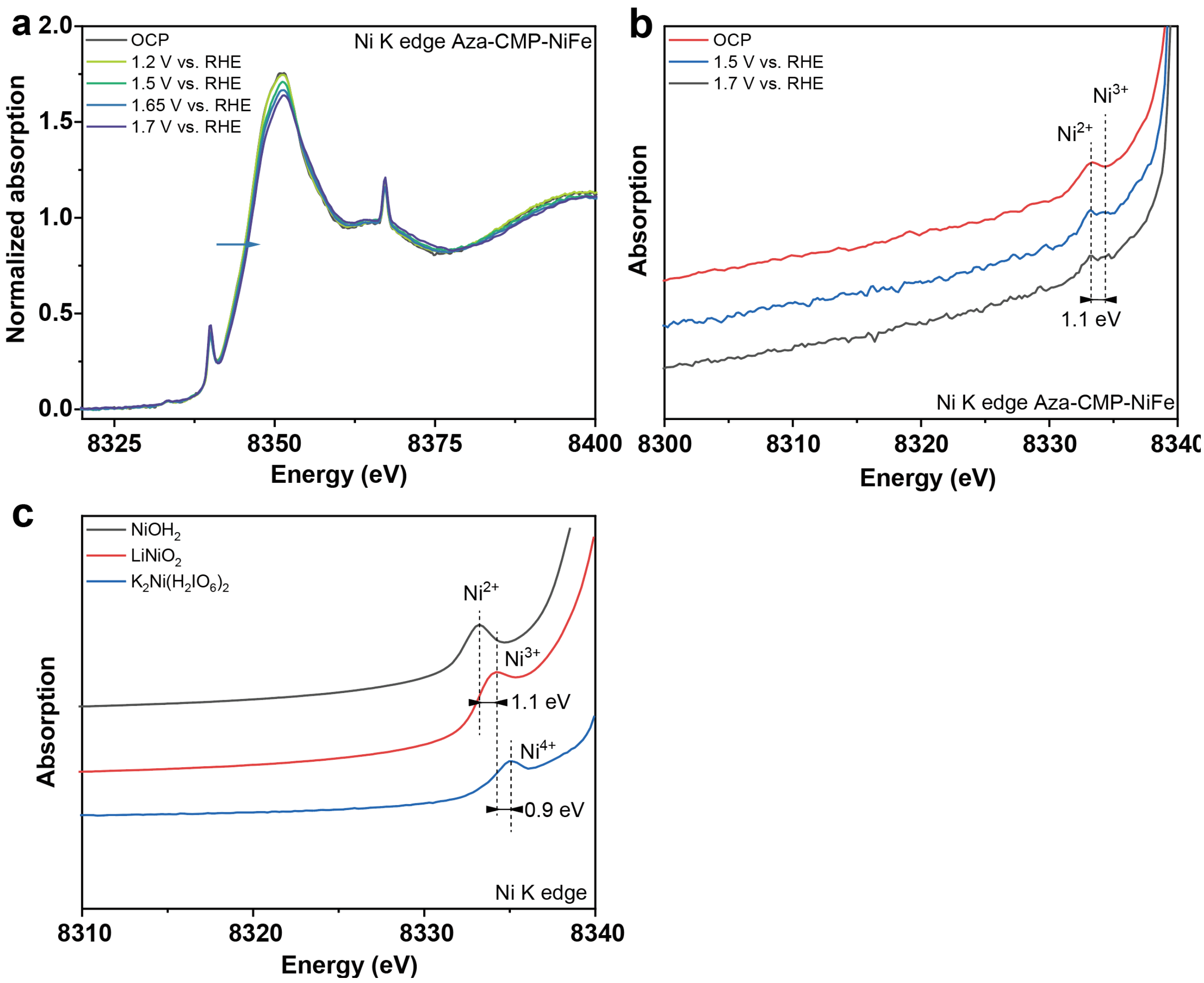

Supplementary Fig. 64 *Operando* Ni K-edge XANES spectra. (a) Aza-CMP-NiFe under different potentials. XANES spectra of (b) Aza-CMP-NiFe and (c) reference materials in pre-edge area.

Supplementary Table 2 Edge-jump energies (0.5 normalized absorbance) at Ni K edge.

**Sample**	**Edge-jump energy**	**Oxidation number**
Ni metal	8340	0
$Ni(OH)_2$	8342.9	2
$LiNiO_3$	8344.6	3
$K_2Ni(H_2IO_6)_2$	8346.3	4
Aza-CMP-Ni @ OCP	8343.27	2.14
Aza-CMP-Ni @ 1.2 V vs. RHE	8343.35	2.19
Aza-CMP-Ni @ 1.5 V vs. RHE	8344.02	2.61
Aza-CMP-Ni @ 1.8 V vs. RHE	8344.90	3.17
Aza-CMP-NiFe @ OCP	8343.21	2.10
Aza-CMP-NiFe @ 1.2 V vs. RHE	8343.24	2.12
Aza-CMP-NiFe @ 1.5 V vs. RHE	8343.55	2.32
Aza-CMP-NiFe @ 1.65 V vs. RHE	8343.64	2.37
Aza-CMP-NiFe @ 1.7 V vs. RHE	8343.83	2.49

Supplementary Discussion 5

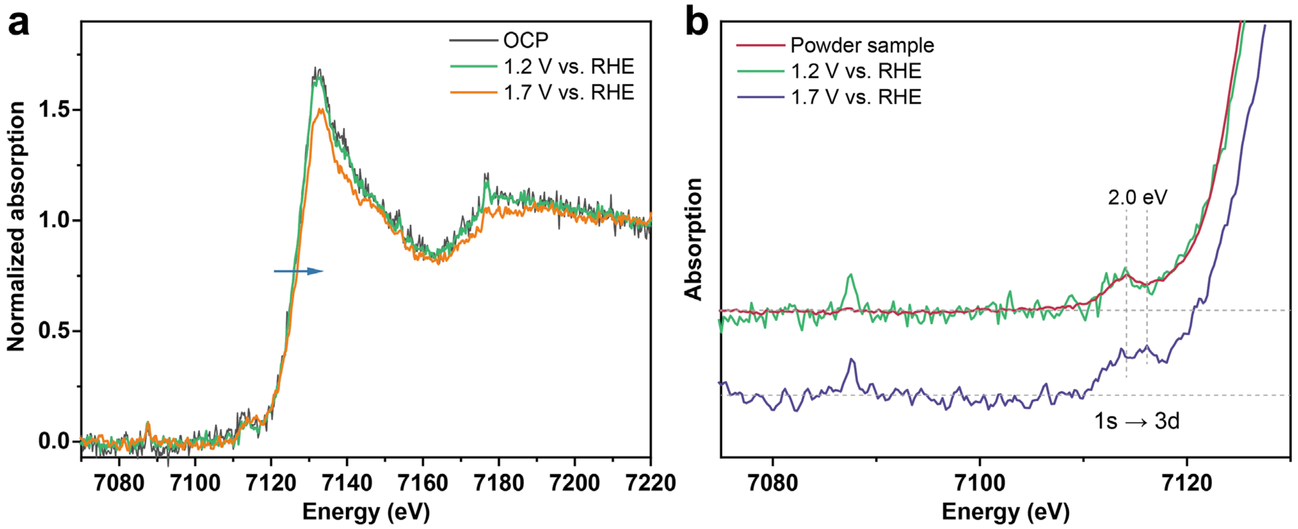

Supplementary Fig. 65 *Operando* Fe K-edge XANES spectra for Aza-CMP-NiFe (a) under different potentials and (b) in zoomed pre-edge and white line areas.

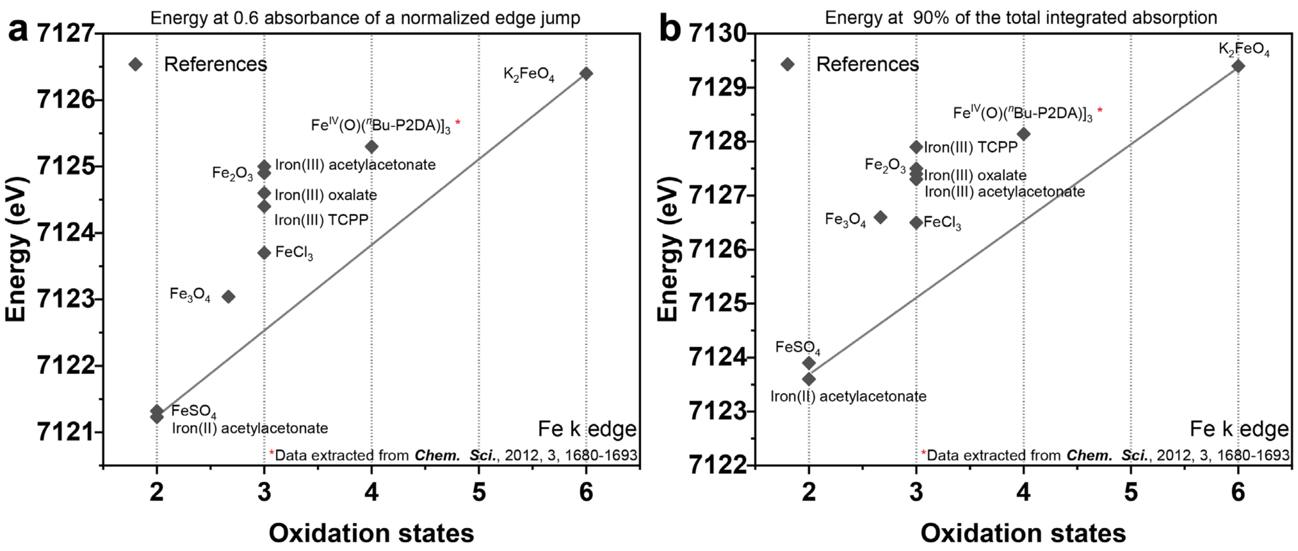

Supplementary Fig. 66 Absorption edge energy. (a) Edge-jump energies (at 0.6 absorbance) of Fe K-edge XANES spectra for reference materials with different oxidation states. (b) Edge energies (at 90% of total integrated absorption) of Fe K-edge XANES spectra for reference materials with different oxidation states.

Supplementary Discussion 5

Supplementary Table 3 Edge-jump energies (0.6 normalized absorbance) at Fe K edge.

Sample	Edge-jump energy	Oxidation number
Fe(II) acetylacetonate	7121.2	2
$FeSO_4$	7121.3	2
**Fe(II) average**	**7121.25**	**2**
Fe_3O_4	7123.0	2.67
Fe(III) acetylacetonate	7125.0	3
Fe_2O_3	7124.9	3
$FeCl_3$	7123.7	3
Fe(III) oxalate	7124.6	3
Fe(III) TCPP	7124.4	3
**Fe(III) average**	**7124.4**	**3**
Fe(IV)(O)(^{n}Bu-P2DA)]$_3$	7125.3*	4
K_2FeO_4	7126.4	6
Aza-CMP-NiFe @ OCP	7124.63	3.11 (Linear)
		3.28 (Curved)
Aza-CMP-NiFe @ 1.2 V vs. RHE	7124.68	3.14 (Linear)
		3.31 (Curved)
Aza-CMP-NiFe @ 1.7 V vs. RHE	7125.56	3.43 (Linear)
		3.96 (Curved)

TCPP = meso-Tetra(4-carboxyphenyl)porphine. ^{n}Bu-P2DA = *N*-(1′,1′-bis(2-pyridyl)pentyl)iminodiacetate)
* Data extracted from the literature[61].

Supplementary Table 4 Edge-jump energies (90% of total integrated absorption) at Fe K edge.

Sample	Edge-jump energy	Oxidation number
Fe(II) acetylacetonate	7123.9	2
$FeSO_4$	7123.6	2
**Fe(II) average**	**7123.75**	**2**
Fe_3O_4	7126.6	2.67
Fe(III) acetylacetonate	7127.3	3
Fe_2O_3	7127.5	3
$FeCl_3$	7126.5	3
Fe(III) oxalate	7127.4	3
Fe(III) TCPP	7127.9	3
**Fe(III) average**	**7127.3**	**3**
Fe(IV)(O)(^{n}Bu-P2DA)]$_3$	7128.1*	4
K_2FeO_4	7129.4	6
Aza-CMP-NiFe @ OCP	7127.04	2.87 (Linear)
		2.88 (Curved)
Aza-CMP-NiFe @ 1.2 V vs. RHE	7127.09	2.87 (Linear)
		2.90 (Curved)
Aza-CMP-NiFe @ 1.7 V vs. RHE	7128.13	3.17 (Linear)
		3.65 (Curved)

* Data extracted from the literature[61].

Supplementary Discussion 5

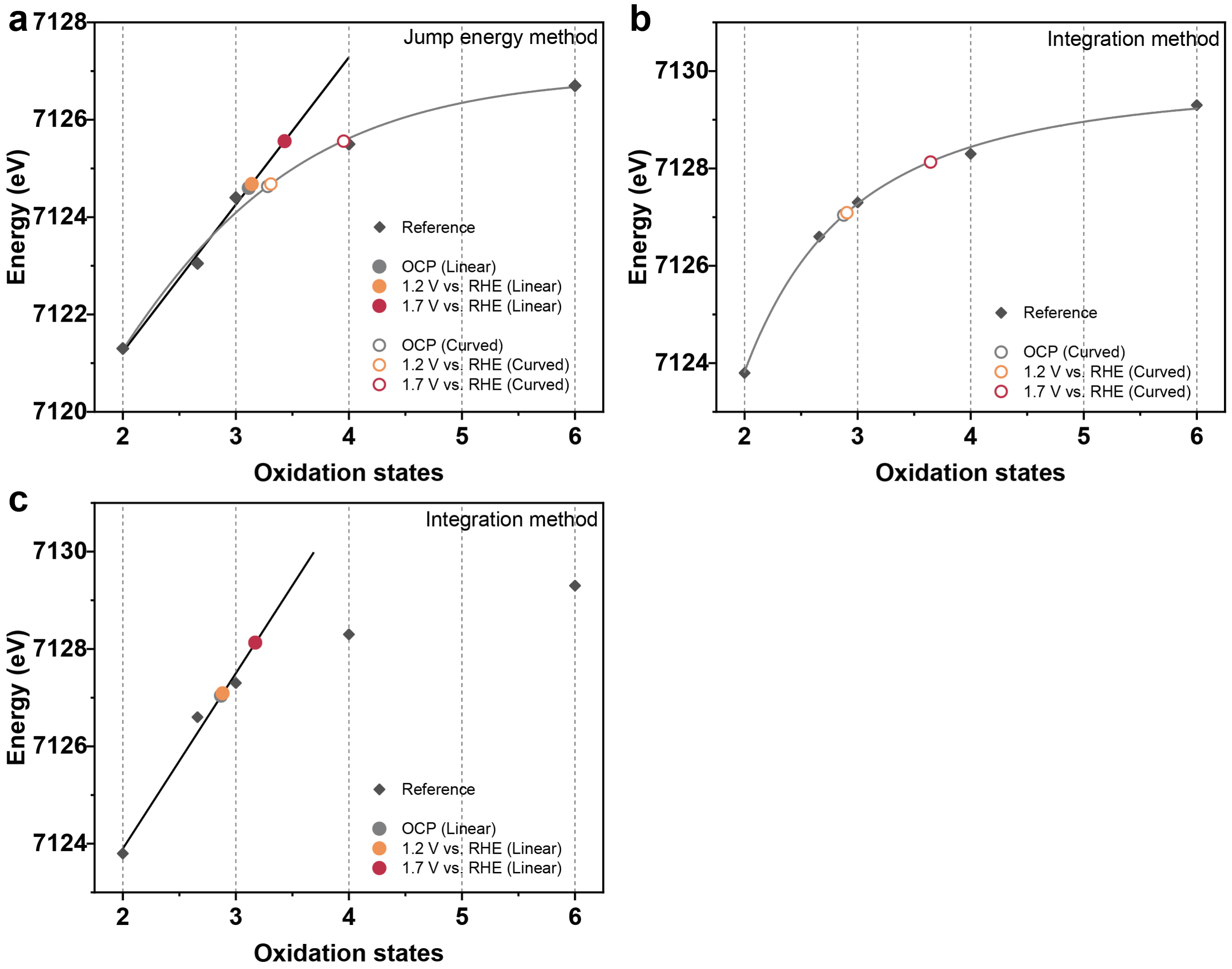

Supplementary Fig. 67 Calculation of oxidation states in Fe K-edge XANES spectra. (a) Edge-jump energies (at 0.6 absorbance) of Aza-CMP-NiFe at various potentials with oxidation states are estimated by linear and curved relationships. Edge energies (at 90% of total integrated absorption) of Aza-CMP-NiFe at various potentials with the oxidation states are estimated by (b) linear relationship and (c) curved relationship.

5.2 UV-Vis

Operando electrochemical UV-vis spectroscopy was employed to further corroborate the metal redox intermediates present in the catalysts during voltammetric cycling. The transmission UV-vis absorption spectra are shown in ***Supplementary Fig. 68***. As the electrode potential is increased from 0.9 to 1.7 V, a broad absorption band ranging from 330 to 600 nm is discernible in the Aza-CMP-Ni/indium tin oxide (ITO) electrode (***Supplementary Fig. 68a***). The potential of the occurrence of the optical transition coincides with the $Ni^{2+/3+}$ oxidation peak (***Supplementary Fig. 69a***). This spectral feature of the detected optical transition could be associated with the *d-d* inter-band transition of Ni^{3+} species.[64] The Aza-CMP-NiFe/ITO electrode displays a broad absorption band similar to that of Aza-CMP-Ni/ITO (***Supplementary Fig. 68b***), but with the additional presence of a shoulder peak between 500 and 650 nm (see comparison in ***Supplementary Fig. 69b***). This spectral change at 550 nm is fully reversible with potential, suggesting that the new absorption is caused by a change in the redox state of the surface-active species. Correspondingly, the absorption-potential relationship of the shoulder peak aligns well with the LSV oxidation waves, indicating that the new species is likely co-produced in the $Ni^{2+/3+}$ redox process (***Supplementary Fig. 69a***). To verify the absorption of active Fe^{4+} species,

Supplementary Discussion 5

UV-vis spectra of the reference Fe_2O_3 compound are presented in ***Supplementary Fig. 68c***. During the positive-going voltammetric sweep, the absorption centered around 580 nm is attributed to the Fe^{4+} formation, in agreement with literature.[65] To clearly show the absorption change between Ni and Ni-Fe sites, difference spectra are obtained by subtracting the spectrum of Aza-CMP-Ni measured at 1.7 V from the spectrum of Aza-CMP-NiFe measured at 1.5 V. As shown in ***Supplementary Fig. 69b***, the difference spectrum mirrors the absorption features of the Fe^{4+} species in Fe_2O_3 reference, affirming that the new absorption ranging from 500 to 650 nm is indicative of the generation of Fe^{4+} species (***Supplementary Fig. 68b***).

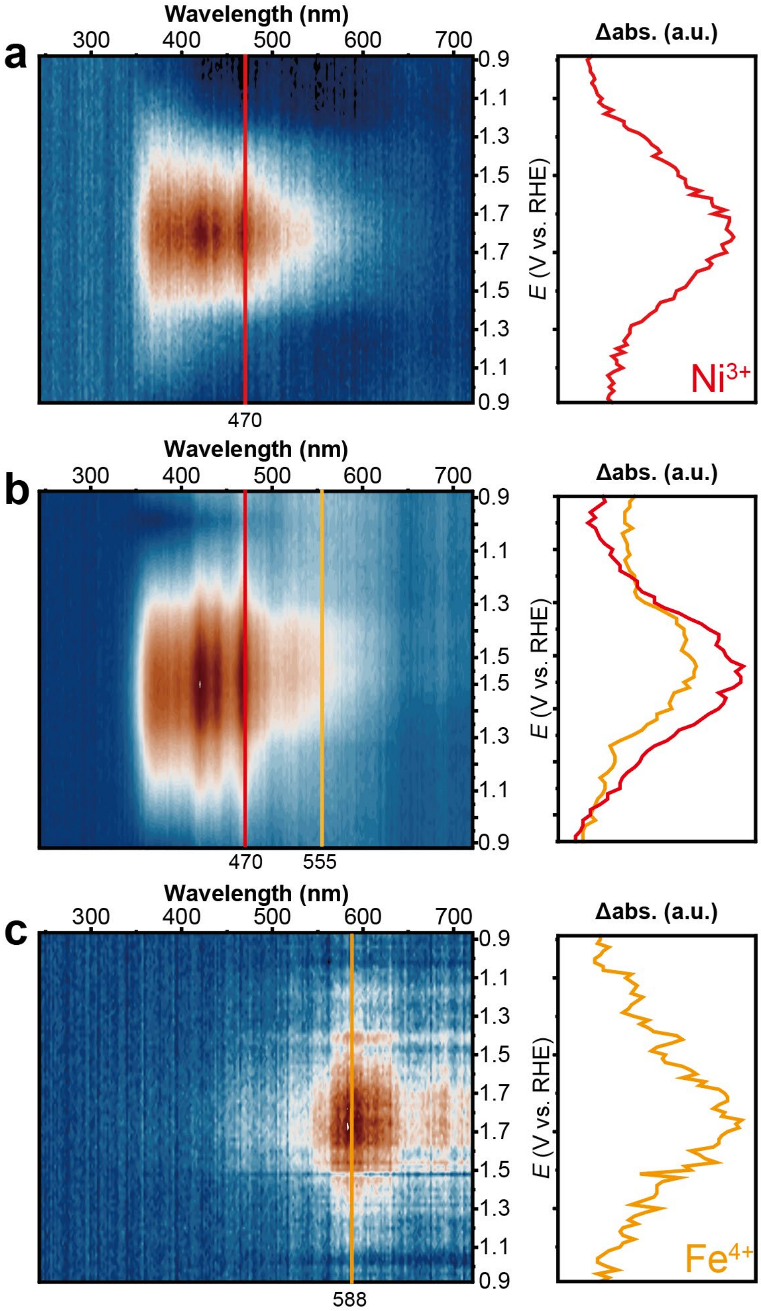

Supplementary Fig. 68 Contour plots of UV-vis absorption as a function of wavelength and potential. (a) Aza-CMP-Ni/ITO, (b) Aza-CMP-NiFe/ITO, and (c) reference Fe_2O_3/ITO. The electrolyte is 1.0 M NaOH.

Supplementary Discussion 5

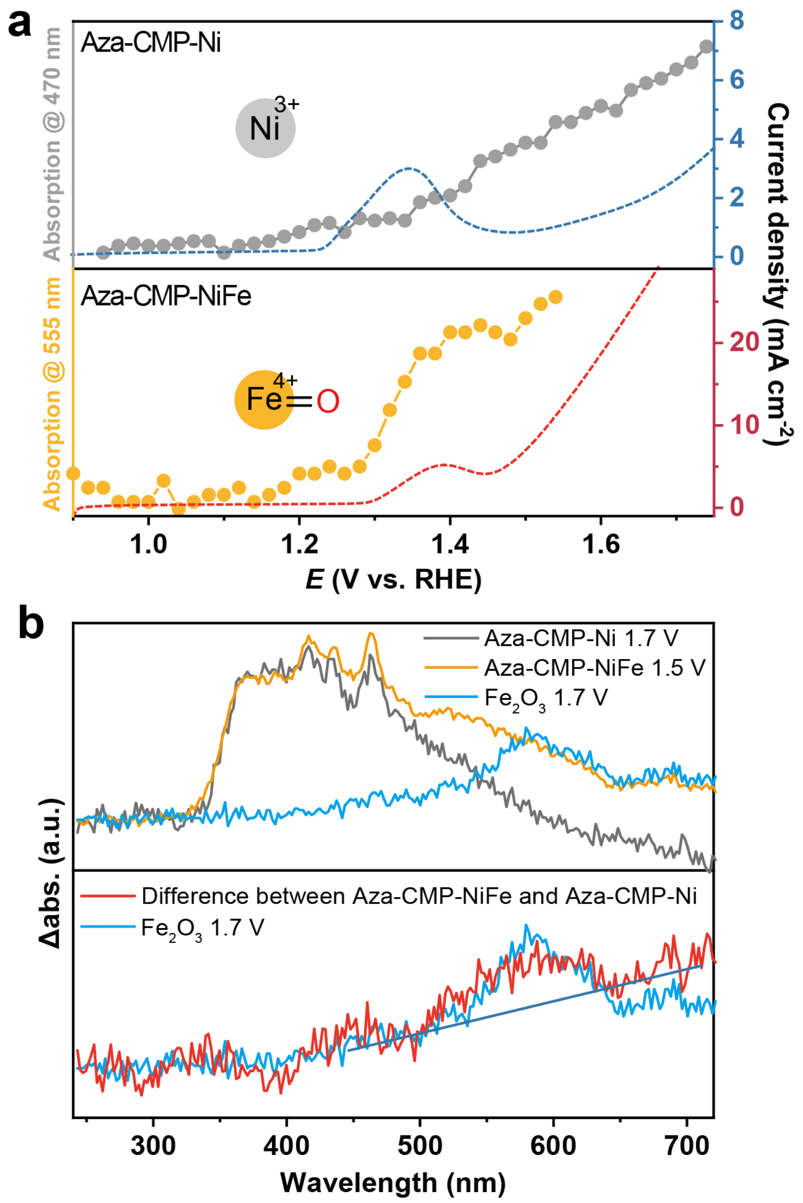

Supplementary Fig. 69 UV-vis absorption as a function of applied potential. (a) Potential dependence of the absorbance of absorption bands at 470 nm (Aza-CMP-Ni) and 555 nm (Aza-CMP-NiFe) in 1.0 M NaOH. The dashed lines indicate corresponding LSV curves. (b) UV-vis absorption spectra of Aza-CMP-Ni, Aza-CMP-NiFe, and the Fe_2O_3 reference at OER potential of 1.7 V vs. RHE. Data sourced from ***Supplementary Fig. 68***.

5.3 Mössbauer spectroscopy

Electrochemical *operando* ^{57}Fe Mössbauer spectroscopy was conducted to further investigate the evolution of the Fe centers in the catalyst during water oxidation cycling (***Supplementary Fig. 70*** and ***Supplementary Table 5***). ***Supplementary Fig. 70a*** and ***70b*** present the *operando* ^{57}Fe Mössbauer spectra of Aza-CMP-NiFe collected at open circuit potential (OCP) and 1.10 V vs. RHE. The two spectra are nearly identical, both showing a single doublet with an isomer shift (IS) of 0.33 mm s^{-1} and quadrupole splitting (QS) of 0.52 mm s^{-1}, consistent with high-spin Fe^{3+} species.[66] When the potential is increased to 1.38 V vs. RHE, coinciding with the Ni^{2+} to Ni^{3+} redox transition, significant spectral changes are observed (***Supplementary Fig. 70c***). A new doublet emerges at lower isomer shift values (IS = 0.02 mm s^{-1}, QS = 0.57 mm s^{-1}), with satisfactory goodness-of-fit. These features are consistent with the generation of Fe^{4+} species, and the spectral intensity indicates that approximately 69% of the

Supplementary Discussion 5

Fe sites are further oxidized.[16, 66, 67, 68] As the potential continues to increase and oxygen evolution proceeds, the fraction of Fe^{4+} species rises to 75% at 1.68 V vs. RHE, demonstrating that most Fe centers participate electrochemically in the water oxidation cycle via Fe^{4+} intermediates (***Supplementary Figs. 70d*** and ***70e***). Notably, significant Fe^{4+} accumulation precedes OER onset (***Supplementary Fig. 70f***), indicating that a pre-catalytic two-electron oxidation ($Ni^{2+}Fe^{3+}$ to $Ni^{3+}Fe^{4+}$) occurs. Combined with *operando* XAS and UV-Vis analyses, these results provide compelling evidence for the formation and catalytic relevance of Fe^{4+} species in the Ni-Fe active sites.

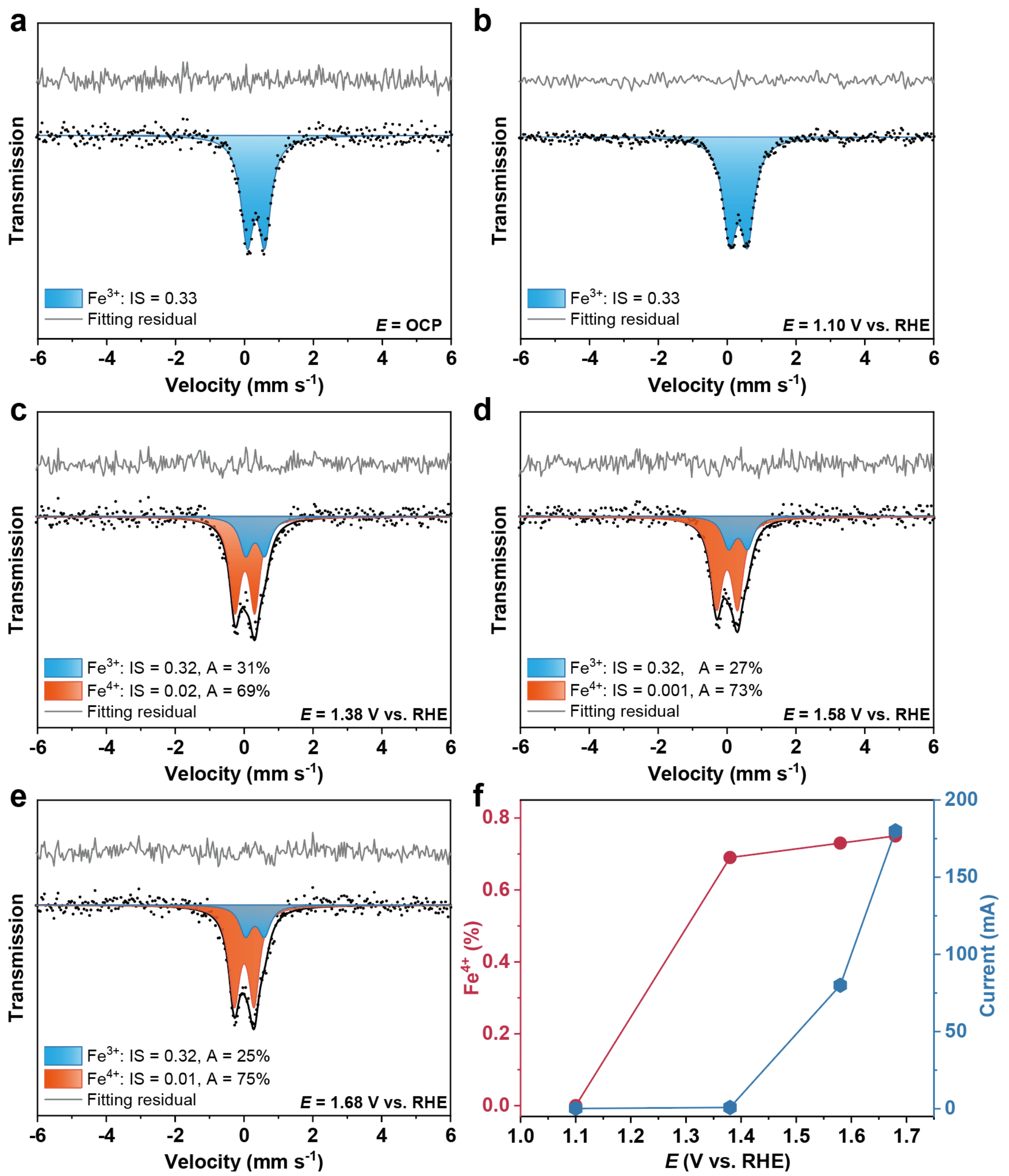

Supplementary Fig. 70 *Operando* ^{57}Fe Mössbauer spectra of Aza-CMP-NiFe collected at: (a) the open circuit potential, (b) 1.10 V vs. RHE, (c) 1.38 V vs. RHE, (d) 1.58 V vs. RHE and (e) 1.68 V vs. RHE. (f) The relative Fe^{4+} content and corresponding catalytic current determined at different applied potentials.

Supplementary Discussion 5

Supplementary Table 5 Mössbauer parameters for Aza-CMP-NiFe.

	**Site1 (Fe^{3+} doublet)**				**Site2 (Fe^{4+} doublet)**			
	**%Comp**	**IS**	**QS**	**Width**	**%Comp**	**IS**	**QS**	**Width**
**OCP**	100%	0.33	0.52	0.44	-	-	-	-
**1.10 V vs. RHE**	100%	0.33	0.52	0.46	-	-	-	-
**1.38 V vs. RHE**	31%	0.32	0.54	0.42	69%	0.02	0.57	0.38
**1.58 V vs. RHE**	27%	0.32	0.54	0.42	73%	0.001	0.59	0.40
**1.68 V vs. RHE**	25%	0.32	0.54	0.42	75%	0.01	0.57	0.39

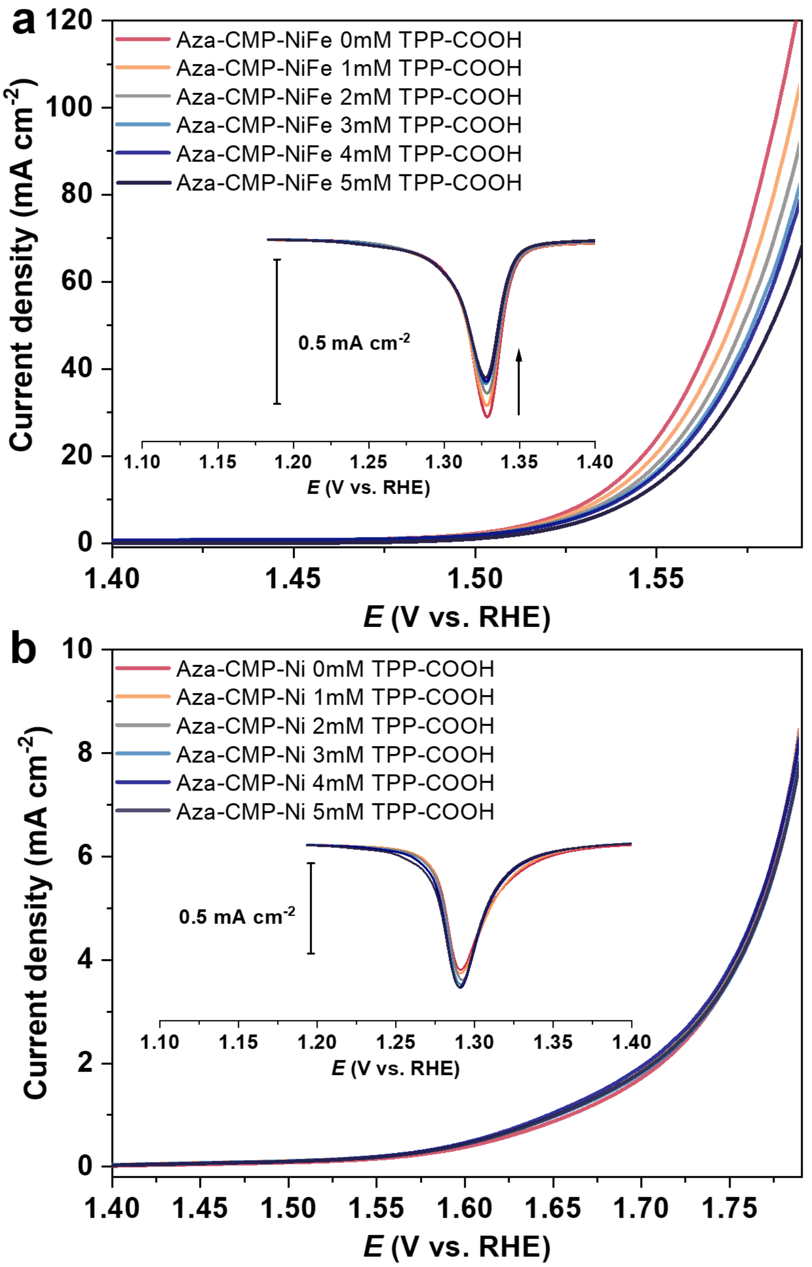

Supplementary Fig. 71 The detection of Fe–O species using oxygen atom transfer probes. CV curves of (a) Aza-CMP-NiFe and (b) Aza-CMP-Ni catalysts in 1 M NaOH with the titration of 0-5 mM 4-(diphenylphosphino) benzoic acid (TPP-COOH) probe (scan rate: 10 mV s^{-1}). Inset: changes in reduction peaks of $Ni^{2+/3+}$ redox.

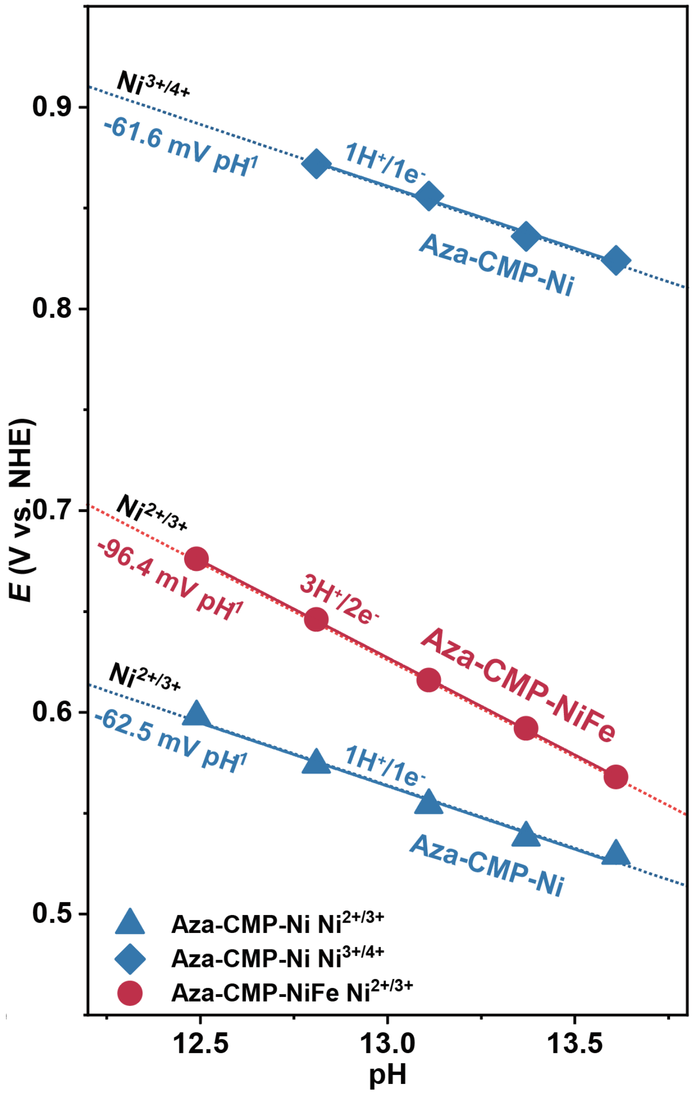

Supplementary Fig. 72 Pourbaix diagram of Aza-CMP-Ni and Aza-CMP-NiFe in NaOH solutions with varying concentrations. The redox potentials were extracted from the DPV curves presented in ***Figs. 3a*** and ***3b*** of the main text.

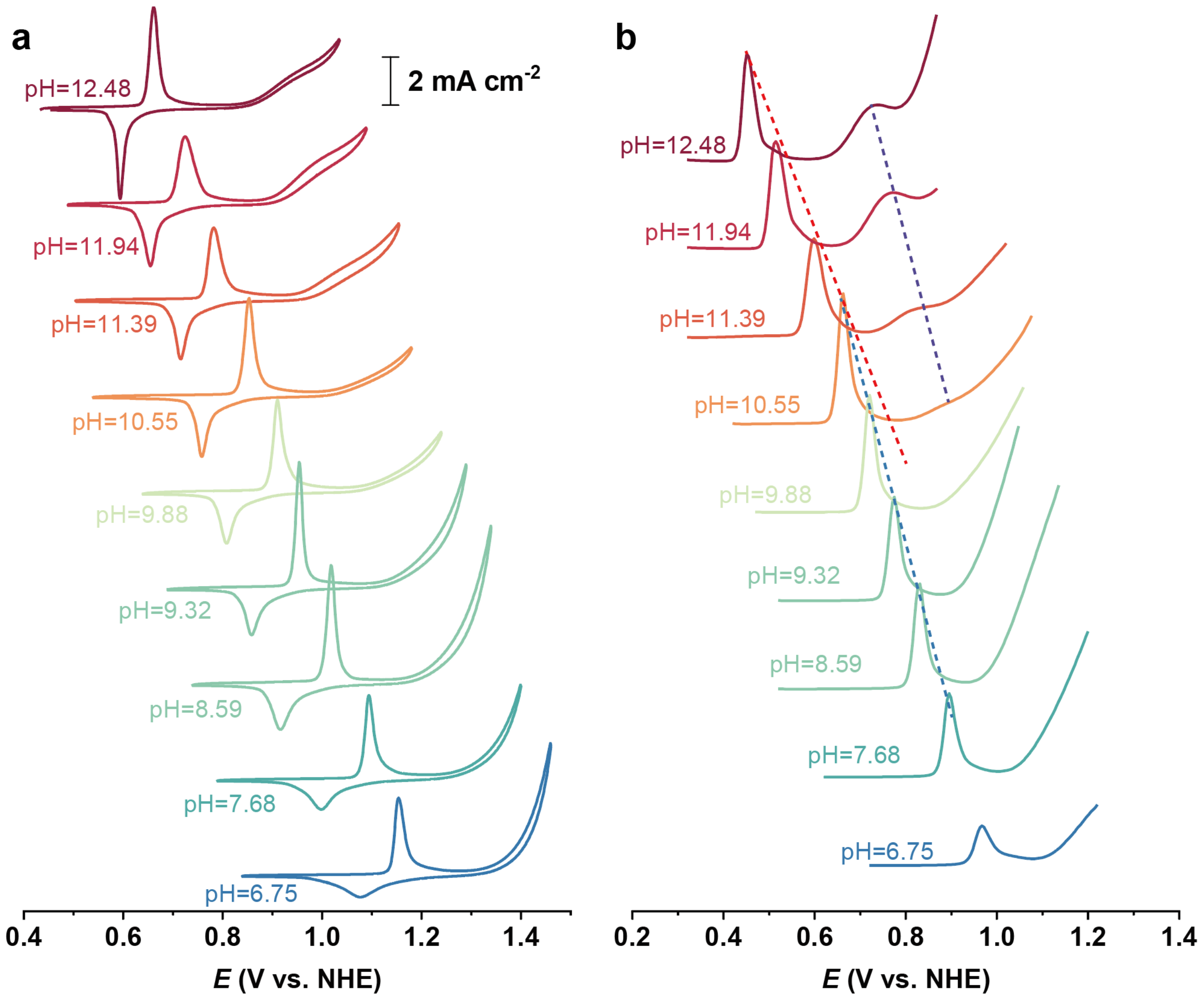

Supplementary Fig. 73 Redox features of Aza-CMP-Ni in different pH conditions. (a) CV and (b) DPV curves (0.5 M borate buffered solutions, CV scan rate: 50 mV s^{-1}, without iR compensation).

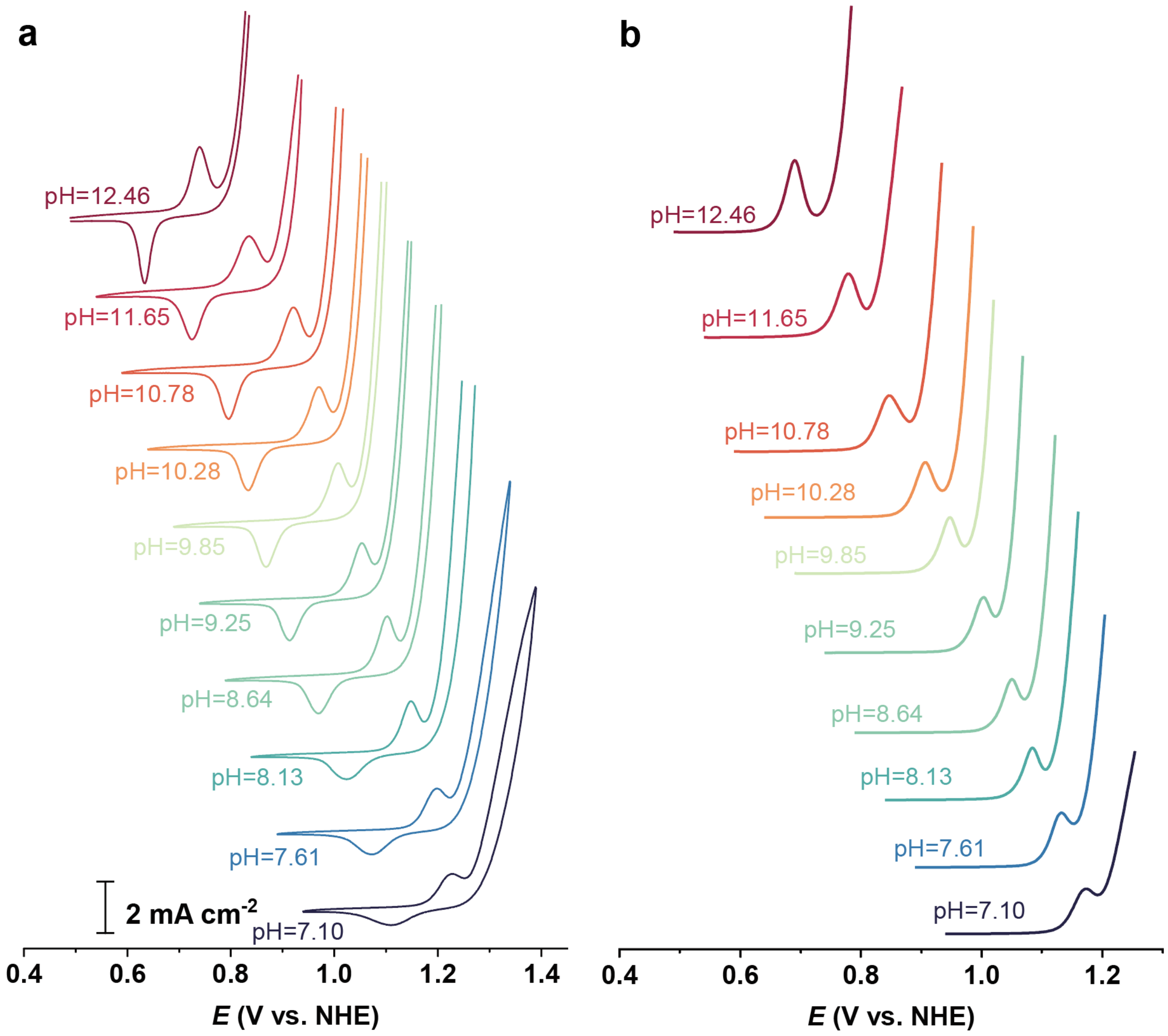

Supplementary Fig. 74 Redox features of Aza-CMP-NiFe in different pH conditions. (a) CV and (b) DPV curves (0.5 M borate buffered solutions, CV scan rate: 50 mV s^{-1}, without iR compensation).

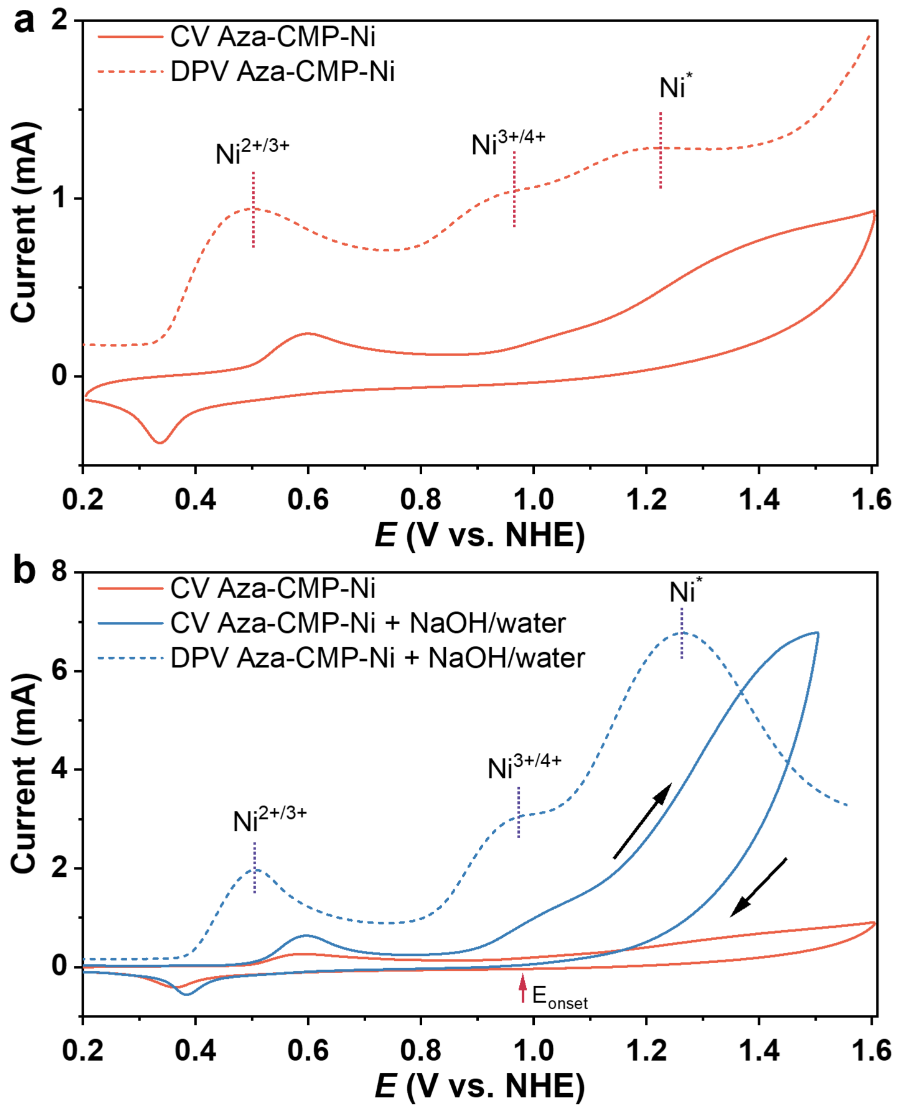

Supplementary Fig. 75 Redox features of Aza-CMP-Ni in nonaqueous conditions. CV and DPV curves of Aza-CMP-Ni/CP in (a) dry acetonitrile/$^{n}Bt_4NPF_6$ and (b) water/NaOH added acetonitrile electrolytes (scan rate: 50 mV s^{-1}). Oxidation peaks at 0.49 V and 0.96 V correspond to the sequential oxidations of $Ni^{2+/3+}$ and $Ni^{3+/4+}$, respectively, while the peak at 1.22 V is attributed to successive PCET processes, denoted as Ni*. When trace amounts of water/NaOH substrates were added to the electrolytes, both the redox peak current and the catalytic current increased significantly. The DPV peaks became more pronounced, although peak positions remained consistent with non-aqueous conditions. The onset potential (E_{onset}) of the catalytic current is measured at 0.99 V (defined as the potential at a backward scan current of 50 μA), which is more anodic than the $Ni^{3+/4+}$ redox potential (0.96 V). This finding suggests that OER on Ni sites is initiated by Ni* oxidation, which becomes immersed in the catalytic current during OER in aqueous solutions.

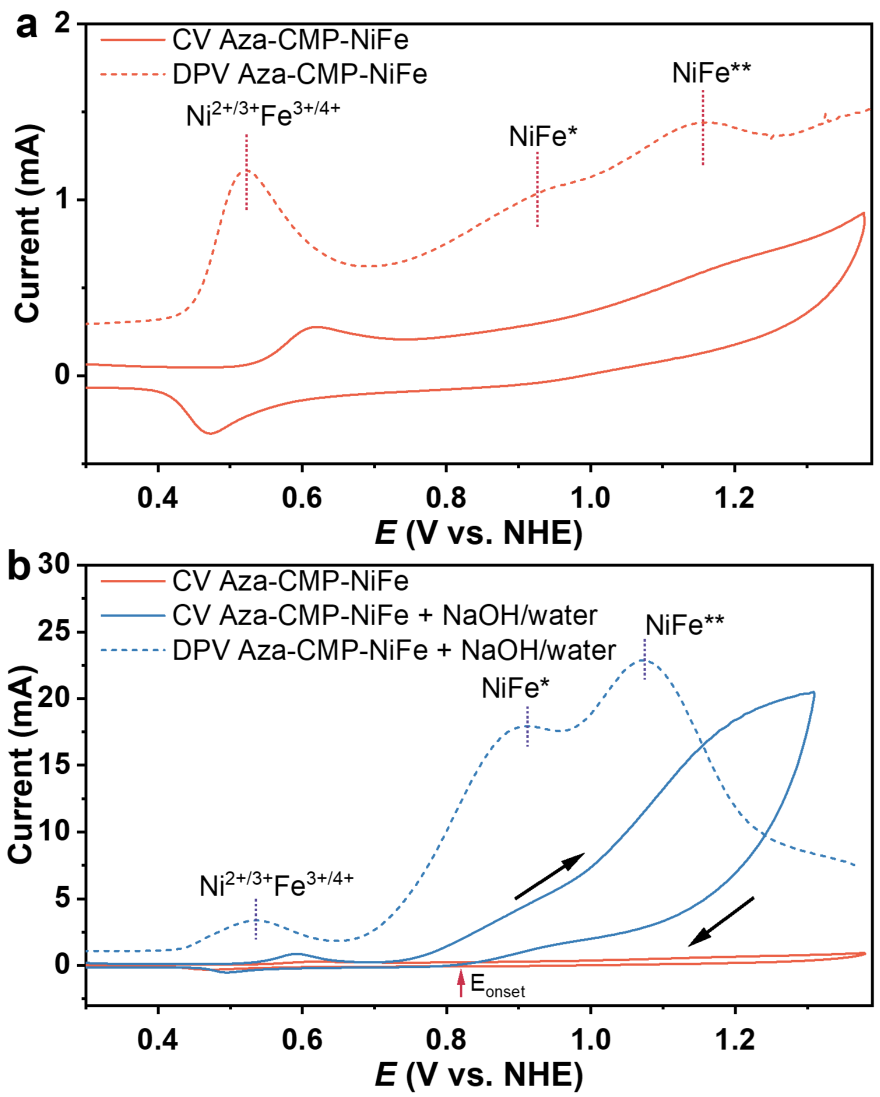

Supplementary Fig. 76 Redox features of Aza-CMP-NiFe in nonaqueous conditions. CV and DPV curves of Aza-CMP-NiFe/CP in (a) dry acetonitrile/$^{n}Bt_4NPF_6$ and (b) water/NaOH added acetonitrile electrolytes (scan rate: 50 mV s^{-1}). The nonaqueous CV and DPV display three oxidation peaks, with the first peak at 0.52 V attributed to the $Ni^{2+/3+}Fe^{3+/4+}$ couple. The two subsequent peaks at 0.92 V and 1.15 V, which are replaced by a strong catalytic current for water oxidation under aqueous conditions, could be ascribed to successive PCET processes (denoted as NiFe* and NiFe**). In the presence of water/NaOH substrates, the E_{onset} of the Ni-Fe sites catalyzing OER is measured at 0.82 V, which is more cathodic than the NiFe* oxidation (0.91 V), further suggesting that the OER current is synchronous with the onset of the NiFe* oxidation wave. The NiFe* oxidation may be attributed to the concerted proton-electron transfer with O–O bond formation; the subsequent NiFe** oxidation may represent further oxidation of the intermediate Fe–OO species, resulting in oxygen release.

Supplementary Discussion 6

Supplementary Discussion 6: Water oxidation mechanism clarification

6.1 Water oxidation kinetics under strong alkaline conditions

Assessing the pH dependence of OER activity can offer valuable insights into the proton-electron transfer characteristics of the RDS within specific reaction pathways (see ***Supplementary Notes 4*** for the explanation of pH-dependent OER activity). As illustrated in ***Supplementary Fig. 77a***, the potential shifts of Aza-CMP-Ni at catalytic current densities ranging from 1.0 mA cm^{-2} to 12 mA cm^{-2} display near-zero slopes at the NHE scale, suggesting that the RDS of Ni sites is primarily an electron transfer (ET) process under strong alkaline conditions. Conversely, for Aza-CMP-NiFe, the potentials of the OER catalytic waves shifted with pH, and slopes close to −59 mV pH^{-1} are obtained through linear fitting (***Supplementary Fig. 77b***), indicating that the RDS of Ni-Fe sites follows a concerted proton-electron transfer (CPET) process. The kinetic isotope effects ($KIEs_{H/D}$) were assessed to verify the proton transfer process during the catalytic RDS (***Supplementary Figs. 78a*** and ***78b***). The LSV of Aza-CMP-Ni in 1.0 M Fe-free NaOD/D_2O solutions exhibits comparable current densities as those in 1.0 M Fe-free NaOH/H_2O solutions, with the corresponding $KIEs_{H/D}$ values approximating unity throughout the catalytic overpotential range of 0.35-0.55 V. The secondary KIE in Aza-CMP-Ni further corroborates that, under alkaline conditions, (i) the O–H bond cleavage is not involved in the RDS, and (ii) the decoupled ET process may constitute the RDS of OER. In contrast, Aza-CMP-NiFe exhibits entirely distinct catalytic properties, with $KIEs_{H/D}$ values over 2 measured across the catalytic overpotential range of 0.28-0.36 V (***Supplementary Figs. 78c*** and ***78d***). This observation suggests that O–H bond cleavage is involved in the RDS under alkaline conditions, which is also consistent with the CPET feature determined by pH-dependent OER activity. Alkaline earth cations disrupt the hydrogen bond network in solution, which in turn alters the solvation strengths of electrolytes ($Li^+ > Na^+ > K^+$) and affects their interaction with surface intermediates.[15, 69] The cation effects in the oxygen evolution are associated with the interaction of the cation with active oxygenated species, enabling the differentiation between the diffusion-controlled solution APT process and the metal oxo (oxyl) coupling process (***Supplementary Fig. 79a***).[3] When the APT is involved in the water nucleophilic attack pathway, the kinetics associated with the nucleophilic attack of water on oxidized electrophilic species decreases as the solvation strength increases. However, in a rate-limiting O–O bond formation via the metal oxo (oxyl) coupling process or intramolecular hydroxyl transfer process, no such cation effects are observed, as the oxygen atom for O–O bond formation does not directly originate from H_2O/OH^- molecules in the inner Helmholtz plane. As shown in ***Supplementary Fig. 79b***, the OER activity of Aza-CMP-Ni decreases between alkaline cations K^+ and Li^+, suggesting that the RDS follows an electron transfer-controlled solution atom-proton transfer (ET-APT) process with the oxygen atom for O–O bond formation directly originating from the solution water molecule (illustrated schematically in ***Supplementary Fig. 80***). Additionally, Aza-CMP-NiFe demonstrates a similar behavior, where the OER activity also exhibits a strong cation dependence (***Supplementary Fig. 79c***), further indicating that the RDS of water oxidation at the Ni-Fe sites may follow a concerted APT (c-APT) process.

During the OER process, the formation of Fe^{4+}=O occurs concurrently with the absence of Ni^{4+} states in the molecular Ni-Fe sites. This suggests that the c-APT process (i.e., RDS of water oxidation at the Ni-Fe sites) should occur predominantly on the Fe component, involving a nucleophilic attack of water from the hydrogen-bonded network in the solvent. To conduct in-depth research on the intrinsic properties of the reaction pathway for a specified catalyst and understand the discrepancies in the RDS between Ni and Ni-Fe sites-catalyzed OER, the activation energy is assessed through temperature-controlled electrocatalysis according to previously reported methods.[3] As shown in

Supplementary Discussion 6

Supplementary Fig. 81, the OER activities of Aza-CMP-NiFe and Aza-CMP-Ni in OER catalysis exhibit a distinct temperature-dependent characteristic. The apparent activation energy, denoted as W, is derived from the corresponding Arrhenius plots of the current at various potentials against 1/T. As evident from the inverse proportionality between W and overpotential in ***Supplementary Fig. 82***, the activation energy value of Aza-CMP-NiFe is considerably less than that of Aza-CMP-Ni. Within the overpotential range of 0.2 to 0.6 V, the disparity in W at a specific overpotential linearly transitions from 7.9 to 3.8 kcal mol^{-1}, implying a TOF gap of 10^5 to 10^3 times at the reaction overpotential. This further points to different RDS intermediate states that could yield significant intrinsic performance variations.

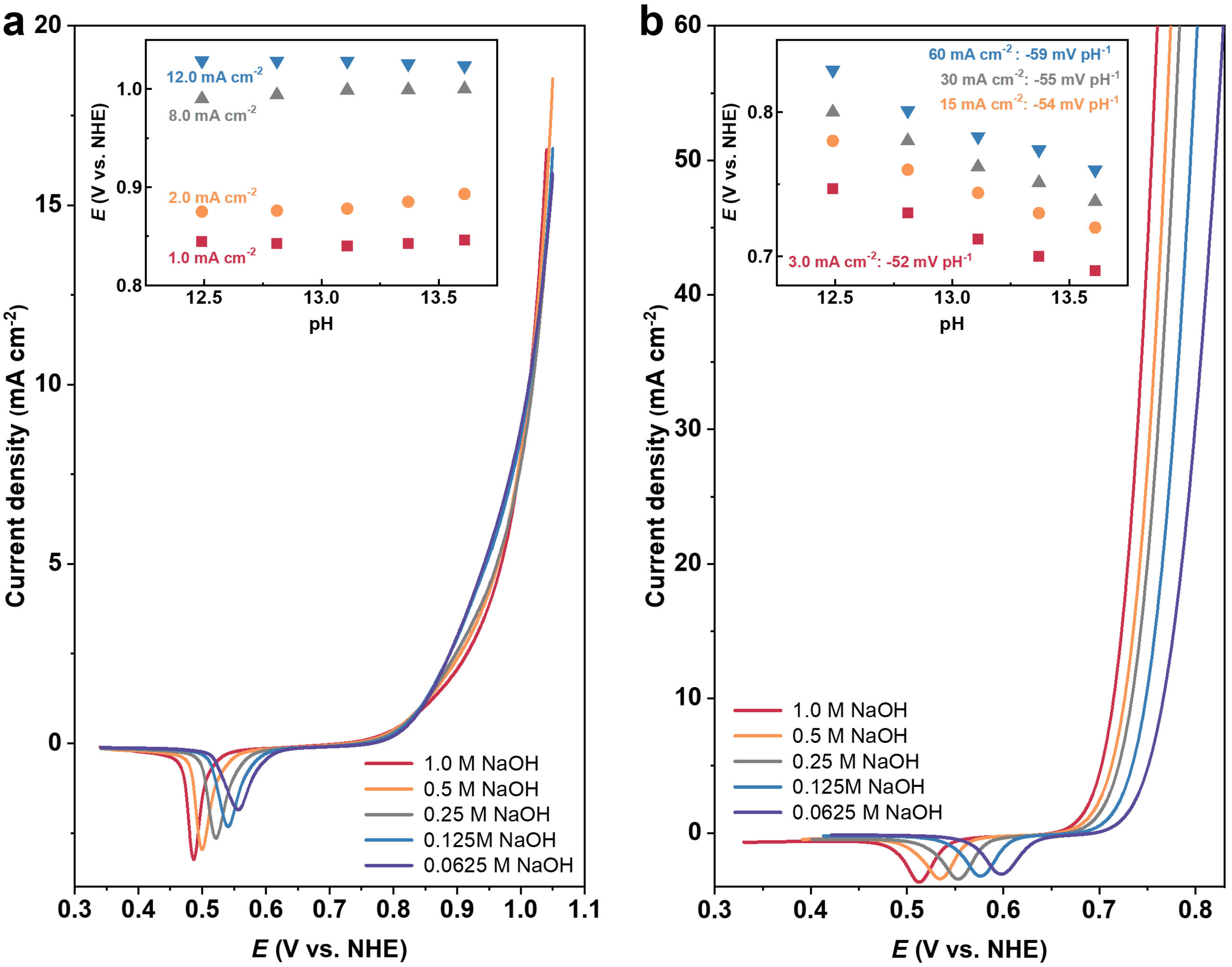

Supplementary Fig. 77 CV curves of Aza-CMP-Ni and Aza-CMP-NiFe in different pH conditions. (a) Aza-CMP-Ni and (b) Aza-CMP-NiFe. Inset: the corresponding pH-dependent OER activity plotted at different catalytic currents (unbuffered Fe-free NaOH solutions; scan rate: 50 mV s^{-1}, without iR compensation). The current variation observed in the Aza-CMP-Ni curves could be attributed to their occurrence within the $Ni^{3+/4+}$ redox region.

Supplementary Discussion 6

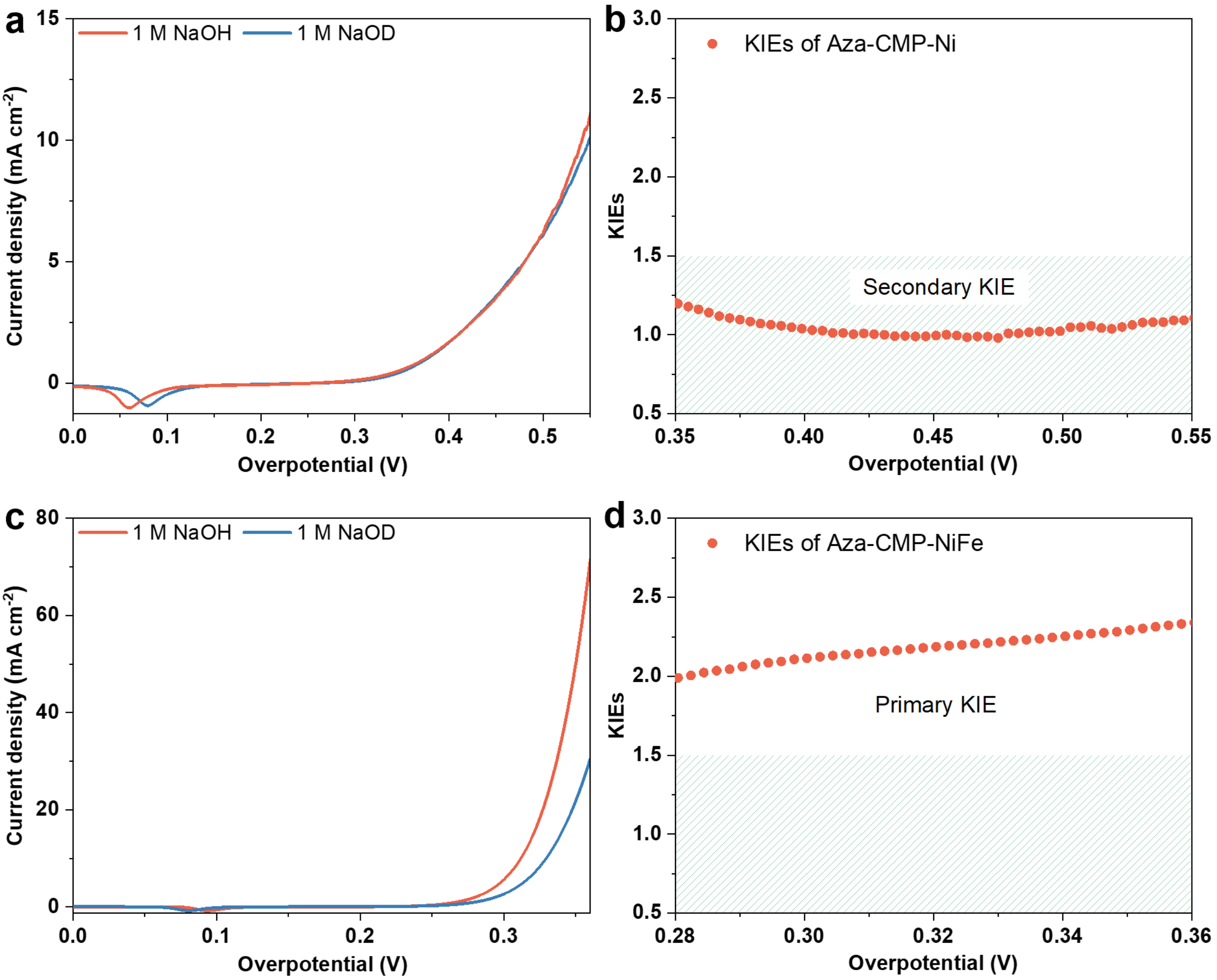

Supplementary Fig. 78 KIE measurements in alkaline pH conditions. (a) LSV curves of Aza-CMP-Ni in 1.0 M Fe-free NaOD/D_2O and NaOH/H_2O solutions (scan rate: 10 mV s^{-1}) and (b) corresponding KIEs against potential. (c) LSV curves of Aza-CMP-NiFe in 1.0 M Fe-free NaOD/D_2O and NaOH/H_2O solutions (scan rate: 10 mV s^{-1}) and (d) corresponding KIEs against potential. KIE values were determined by ***Supplementary Eqn. 6***.

Supplementary Discussion 6

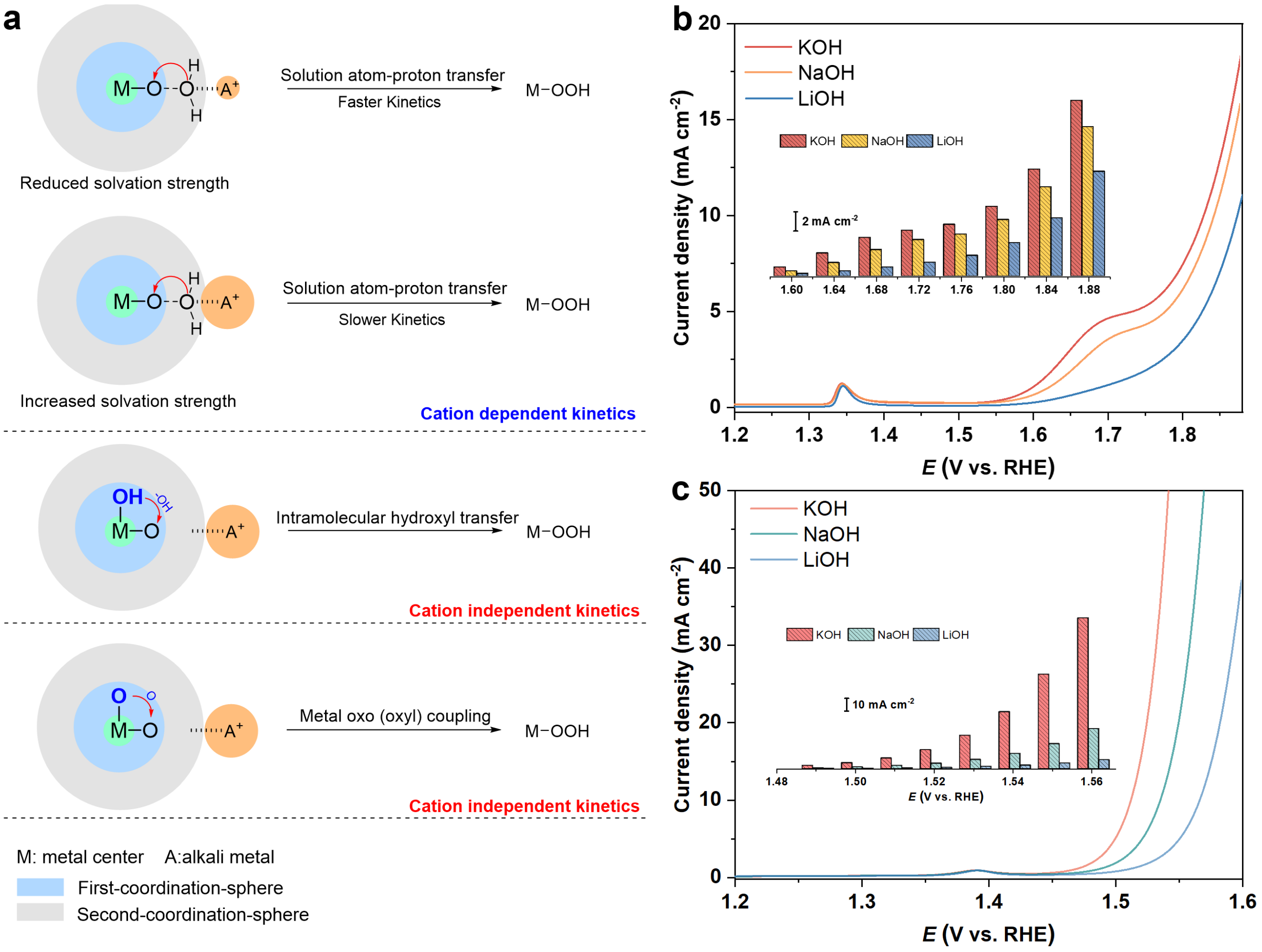

Supplementary Fig. 79 Cation-activity dependency in alkaline conditions. (a) Schematic diagram of cation-activity dependency for different O–O bond formation processes. (b) LSV curves of Aza-CMP-Ni in 1.0 M Fe-free KOH, NaOH, and LiOH solutions; inset: comparison of catalytic current at different potentials (scan rate: 10 mV s^{-1}). (c) LSV curves of Aza-CMP-NiFe in 1.0 M KOH, NaOH, and LiOH; inset: comparison of catalytic current at different potentials (scan rate: 5 mV s^{-1}).

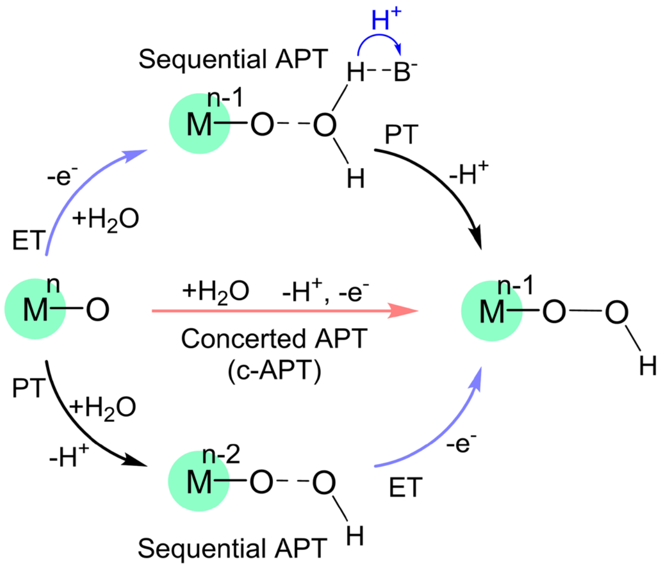

Supplementary Fig. 80 Schematic diagram of solution APT process for the O–O bond formation process.

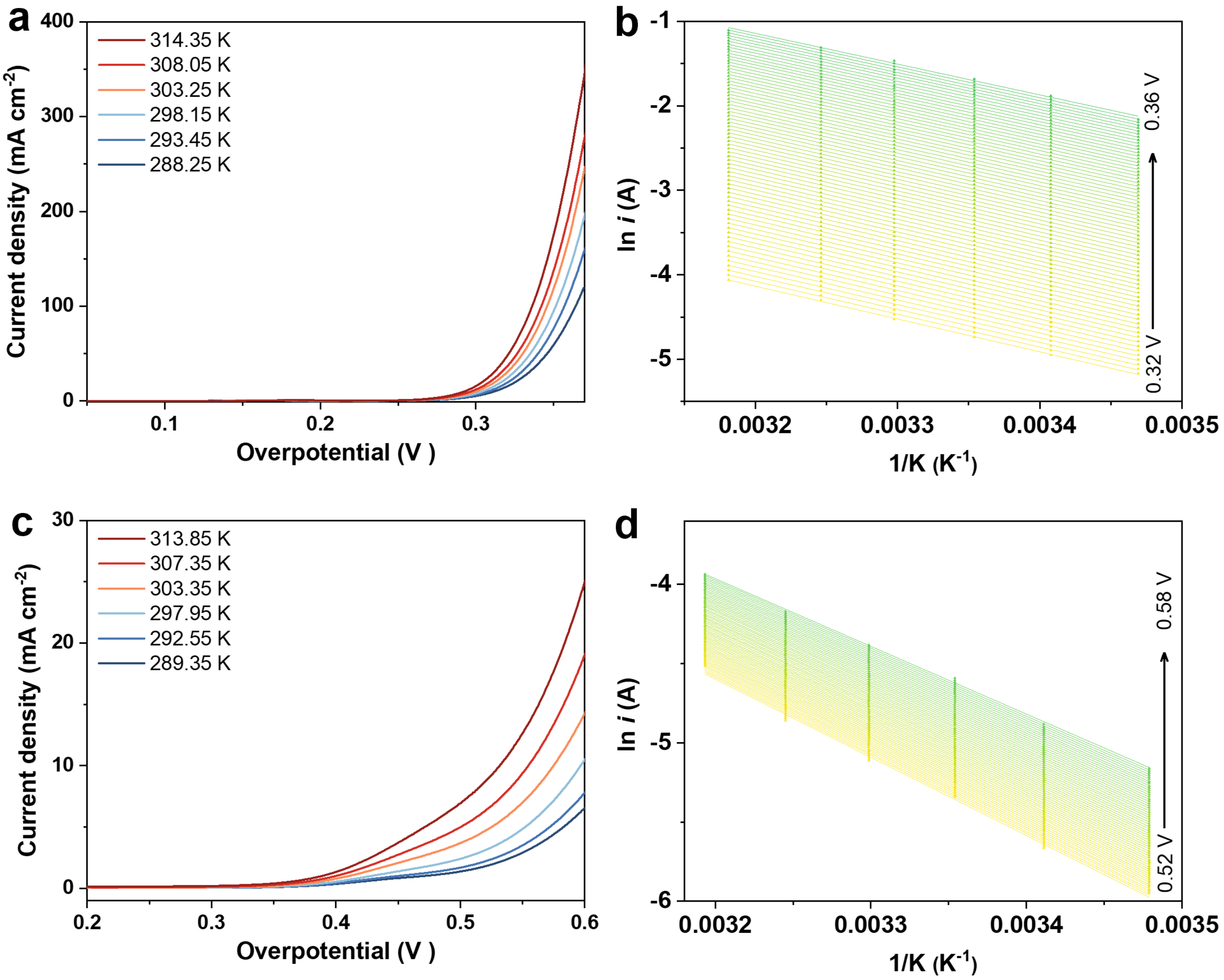

Supplementary Fig. 81 Temperature-dependent LSV curves. (a) Aza-CMP-NiFe and (c) Aza-CMP-Ni. Corresponding Arrhenius plots of (b) Aza-CMP-NiFe and (d) Aza-CMP-Ni at different overpotentials (1.0 M NaOH, scan rate: 5 mV s^{-1}). Please refer to our previous report on experimental methods.[3]

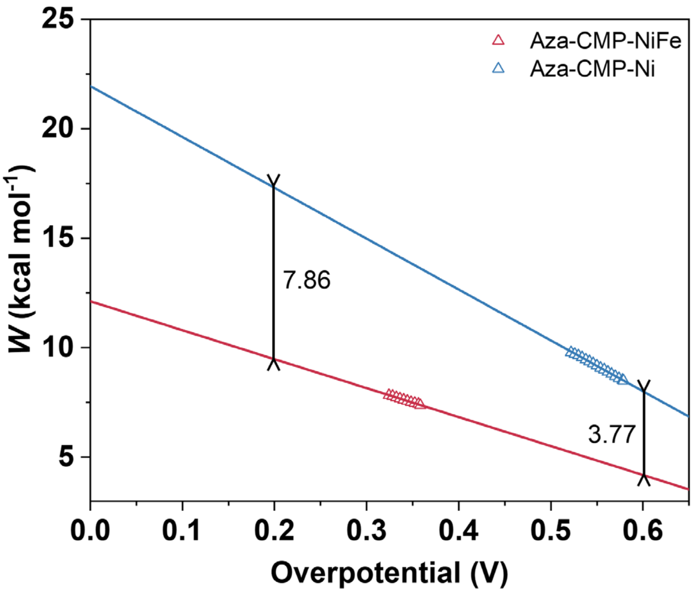

Supplementary Fig. 82 Plots of apparent activation energy (*W*) against reaction overpotential. The data are sourced from ***Supplementary Figs. 80b*** and ***80d***.

Supplementary Discussion 6

6.2 Water oxidation kinetics under weak alkaline conditions

The pH-dependent catalytic activity, spanning from near-neutral to strongly alkaline conditions, was further examined to shed light on the proton-electron transfer properties of RDS. In this work, sodium borate buffer served as the electrolyte within the pH range of 7 to 12. To demonstrate that the possible inflection points observed in the Pourbaix diagrams and activity tests are not attributable to the presence of buffer ions, Aza-CMP-Ni was tested in a diluted Fe-free NaOH solution with a pH adjusted to approximately 12.0 to assess its Pourbaix behavior. As depicted in ***Supplementary Fig. 83***, the same inflection point for the $Ni^{2+/3+}$ oxidation is observed near pH 12.5, aligning with the findings presented in ***Fig. 3c***. This indicates that the presence of an inflection point (i.e., a pK_a point) is intrinsic to the catalyst's redox properties and independent of the electrolyte's composition.

As previously stated, Ni sites follow an ET-controlled RDS in strongly alkaline conditions. Interestingly, as shown in ***Supplementary Fig. 84b***, clear pH-dependent activities are observed with a slope of −123 mV pH^{-1} when the pH is below 12.5, indicating a PT-controlled RDS, as discussed in ***Supplementary Notes 4***. As the pH further decreases to 11.5, the slope of the pH-dependent activities shifts to approximately −69 mV pH^{-1}, suggesting a c-APT process. This observation can potentially be attributed to the structural transformation of OER-active Ni^{4+} species across the pK_a point of Ni^{3+} species (***Supplementary Fig. 84c***). Simultaneously, the Tafel slopes also exhibit a significant change in correspondence with the pH-dependent activity (***Supplementary Fig. 84a***). Within the pH range of 12.5 to 14, the Tafel slope remains in the range of 120 to 130 mV dec^{-1}. However, when the catalytic current displays a pH dependence, the Tafel slope notably increases over 200 mV dec^{-1}, further implying a substantial shift in the catalytic kinetics. As the pH further decreases to below 10, the Tafel slope gradually recovers to approximately 110 mV dec^{-1}, indicating an additional change in the catalytic mechanism. This change coincides with the disappearance of the $Ni^{3+/4+}$ redox peaks in DPV/LSV of Aza-CMP-Ni (***Supplementary Fig. 73***). Since this work mainly focuses on alkaline catalysis, the catalytic mechanism under near-neutral conditions will not be detailed here. The $KIEs_{H/D}$ is found to be over 1.5 within an overpotential range of 0.50 V to 0.65 V, providing further evidence for a PT-controlled RDS under a pH of 12.0 (***Supplementary Fig. 86***). The cation effects were also assessed in a borate-buffered solution (pH 12). As shown in ***Supplementary Fig. 87a***, the evident cation-dependent current indicates that RDS of Ni sites at pH 12 should involve a proton transfer-controlled solution atom-proton transfer (PT-APT) process. Interestingly, when the RDS is controlled by the proton transfer process, an opposite trend in the cation effects is observed, where the catalyst exhibits greater activity in the lithium-ion electrolyte than in the potassium-ion electrolyte. This phenomenon suggests a strong link between the electrolyte cations and the proton transfer rate, aligning with the cation-dependent behavior of the hydrogen evolution reaction (HER) in alkaline environments.[70, 71, 72] Furthermore, the variation in the cation effects trend, noted before and beyond pH 12.5, lends additional support to the alteration in RDS. The transition in the catalytic RDS from ET-APT to PT-APT is likely influenced by the pK_a nature of specific reaction intermediates. For a detailed explanation and theoretical derivation, please refer to ***Supplementary Notes 4***.

For Ni-Fe sites, an inflection point appears at a pH of around 12.5 in the current-pH curve, followed by an increased slope of −88 mV pH^{-1} upon further pH reduction (***Supplementary Fig. 88b***). In the classical interpretation, this process should be attributed to the $3H^+/2e^-$ transfer process. Consequently, a total of six protons and four electrons would be transferred from the initial state to trigger water oxidation, which seems implausible. A more reasonable explanation suggests that the pH dependence of the water oxidation catalytic current (at the foot of the wave) also relies on the pH dependence of the pre-rate-determining step (pre-RDS) (i.e., $Ni^{2+/3+}$ couple). The super-Nernst shifted catalytic current is constrained by the super-Nernst shifted pre-RDS redox, as an external driving force

(100 mV in this case) is still required to drive the pre-RDS state toward OER (***Supplementary Fig. 118*** in ***Supplementary Notes 4***). As the pH decreases, the Tafel slope gradually increases from around 30 to 60 mV dec^{-1} (***Supplementary Fig. 88a***). Given that no pK_a point for $Ni^{2+/3+}Fe^{3+/4+}$ redox is identified between pH 14 to 7, as illustrated in ***Supplementary Fig. 88c***, these shifts in Tafel slopes suggest a change in the O–O bond formation mechanism related to the solvation environment across different pH conditions. Meanwhile, Ni-Fe sites exhibit $KIEs_{H/D}$ values of over 2 at pH 9.3, precluding the possibility that RDS is governed by an ET process (***Supplementary Fig. 90***). Therefore, the RDS for Ni-Fe sites should maintain the $1H^+/1e^-$ characteristics from pH 14 to 7. Additionally, the pronounced cation effects under pH 9.3 support that O–O bond formation, triggered by Ni-Fe sites in near-neutral conditions, follows the similar solution-APT process as in strongly alkaline conditions yet with distinct deprotonation features (***Supplementary Fig. 87b***). In summary, this section has systematically analyzed and provided a detailed comparison of the proton-electron transfer processes in Aza-CMP-Ni and Aza-CMP-NiFe during the OER, with the key results concisely summarized in ***Supplementary Fig. 91***.

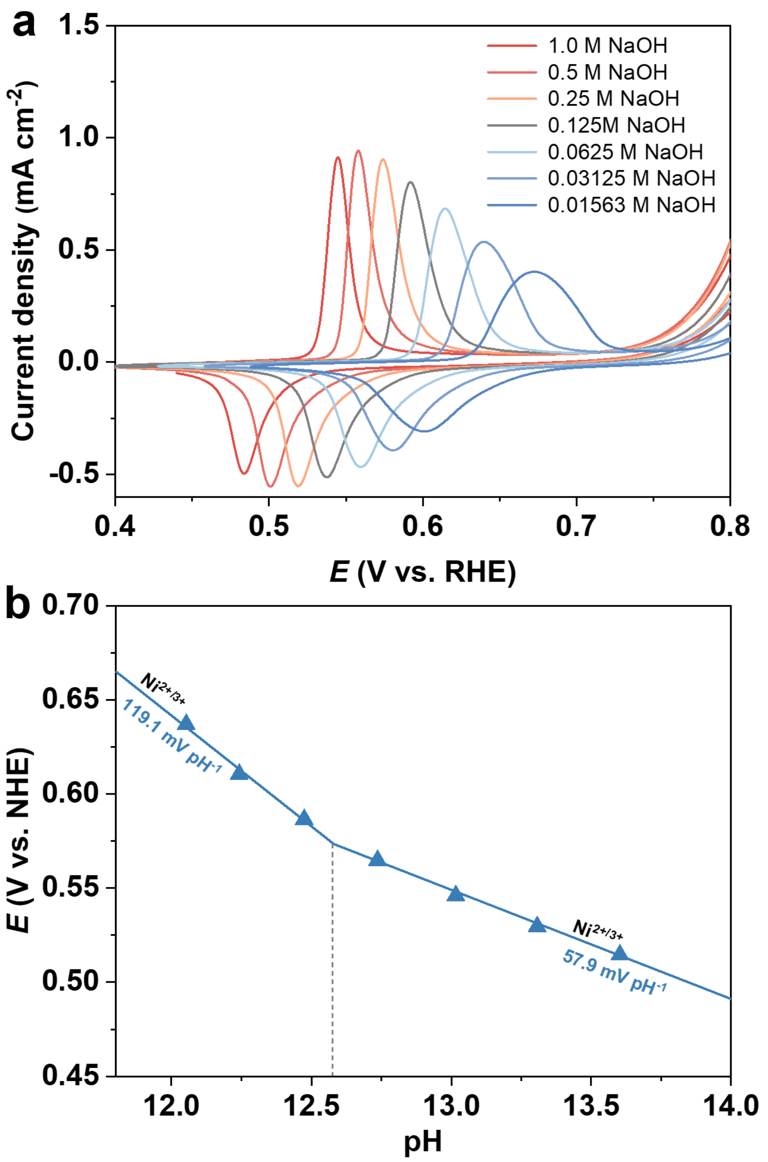

Supplementary Fig. 83 Redox features of Aza-CMP-Ni in alkaline conditions. (a) CV curves and (b) corresponding Pourbaix diagram (unbuffered Fe-free NaOH solutions, CV scan rate: 10 mV s^{-1}, without iR compensation).

Supplementary Discussion 6

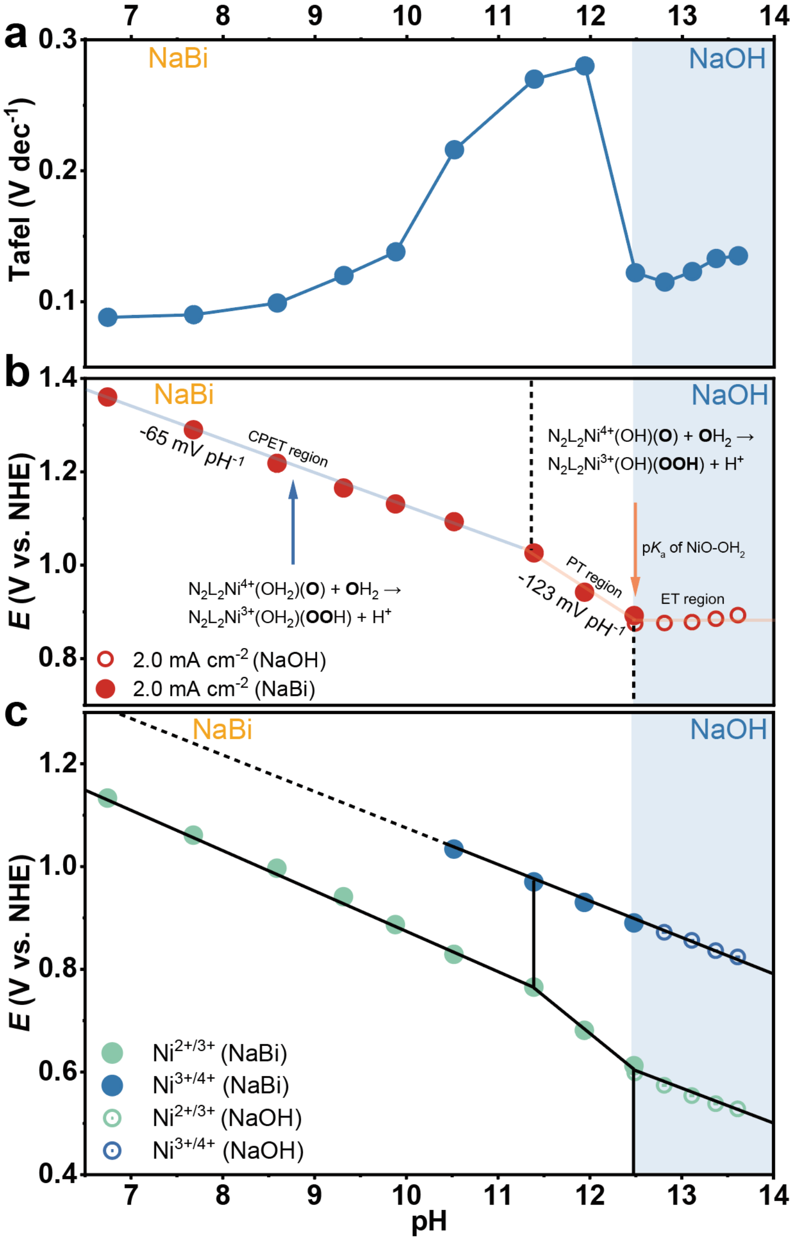

Supplementary Fig. 84 Water oxidation kinetics of Aza-CMP-Ni in different pH conditions. (a) The relationship between Tafel slope and electrolyte pH, the Tafel slope calculation is presented in ***Supplementary Fig. 85***. (b) pH-dependent OER activity under extended pH range. The data are sourced from ***Supplementary Figs. 73*** and ***77***. (c) Pourbaix diagram of Aza-CMP-Ni under the same pH range for direct comparison. The data are sourced from ***Supplementary Figs. 72*** and ***73***.

Supplementary Discussion 6

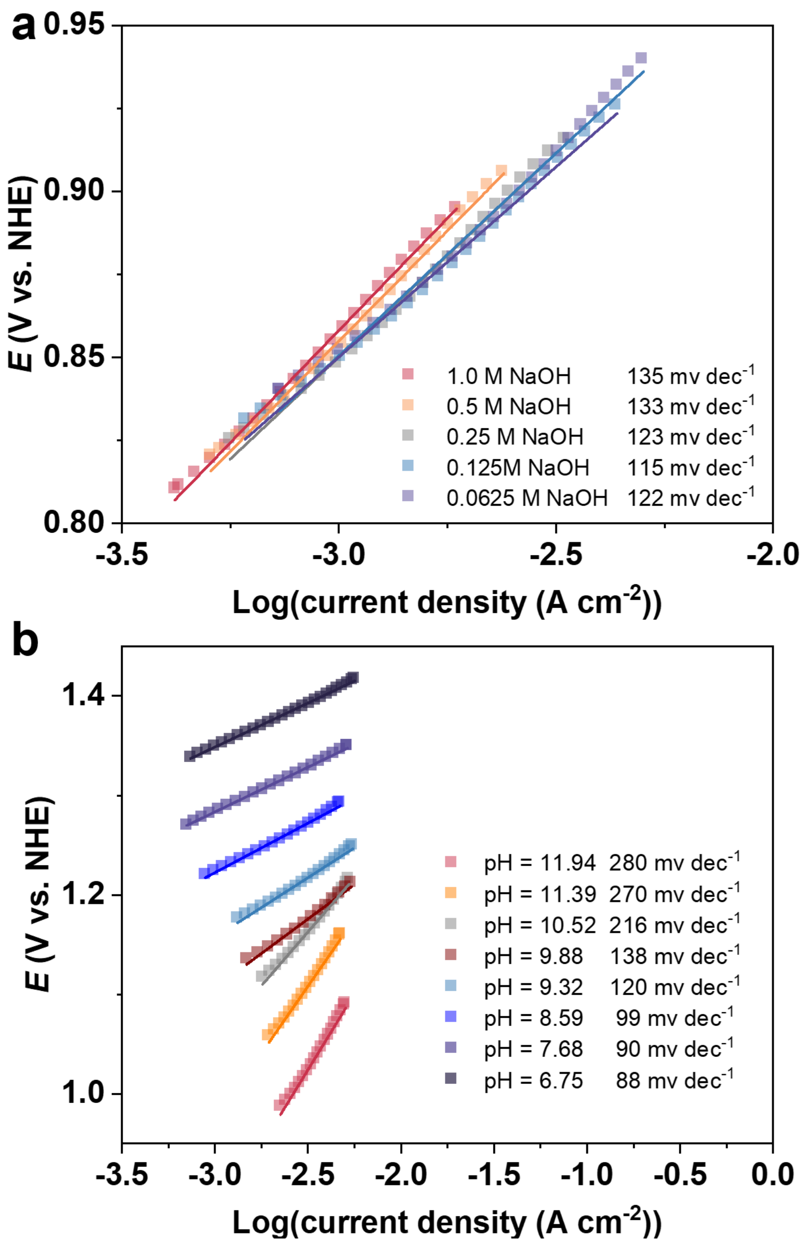

Supplementary Fig. 85 Tafel slopes of Aza-CMP-Ni in different pH conditions. Tafel slopes extracted from the corresponding LSV curves of Aza-CMP-Ni. (a) Fe-free NaOH solutions. (b) Borate buffered solutions. In the pH range of 12 to 10, the catalytic current displays nonlinear behavior, likely resulting from the overlap between the OER current and the $Ni^{3+/4+}$ oxidation peak. While this overlap may compromise the accuracy of the Tafel slope calculation, the overall trend of the Tafel slope with pH remains reliable.

Supplementary Discussion 6

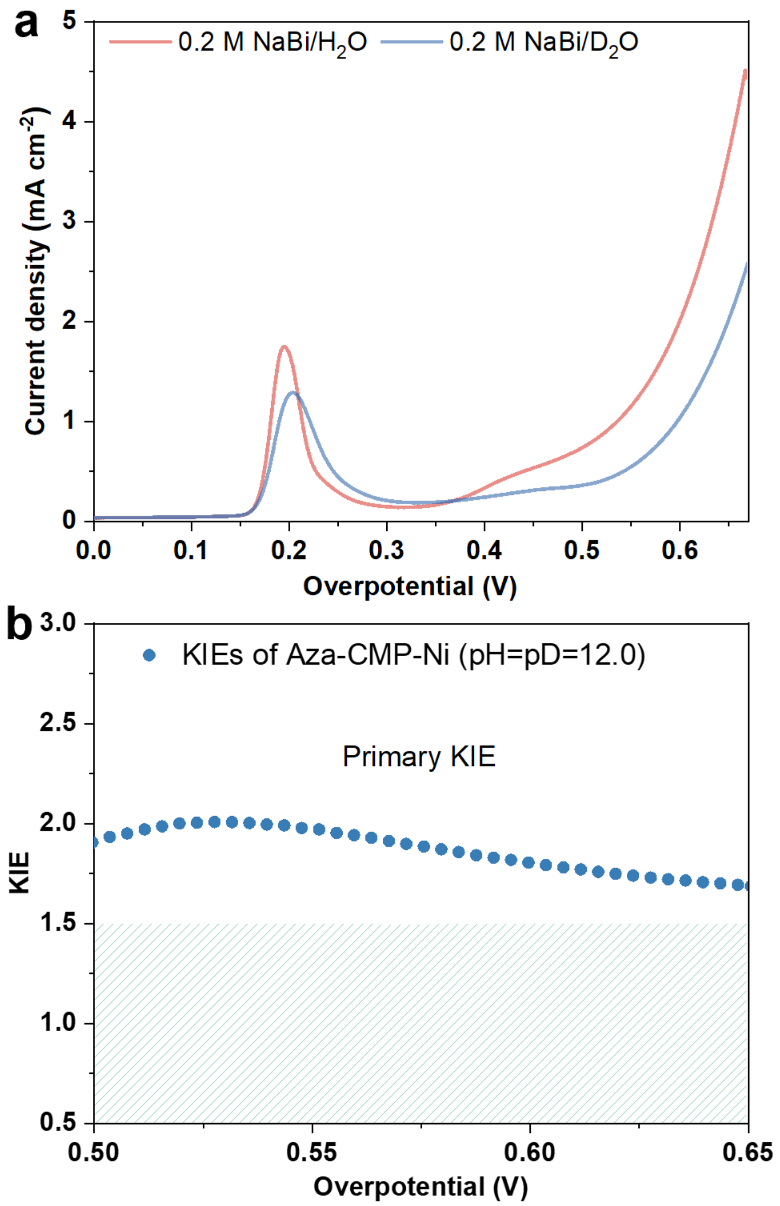

Supplementary Fig. 86 KIE measurements in weak alkaline conditions. (a) LSV curves of Aza-CMP-Ni in 0.2 M NaBi/D_2O and NaBi/H_2O solutions (scan rate: 20 mV s^{-1}) and (b) corresponding KIEs against potential. pH controlled using concentrated Fe-free NaOH or NaOD. KIE values were determined by ***Supplementary Eqn. 6***.

Supplementary Discussion 6

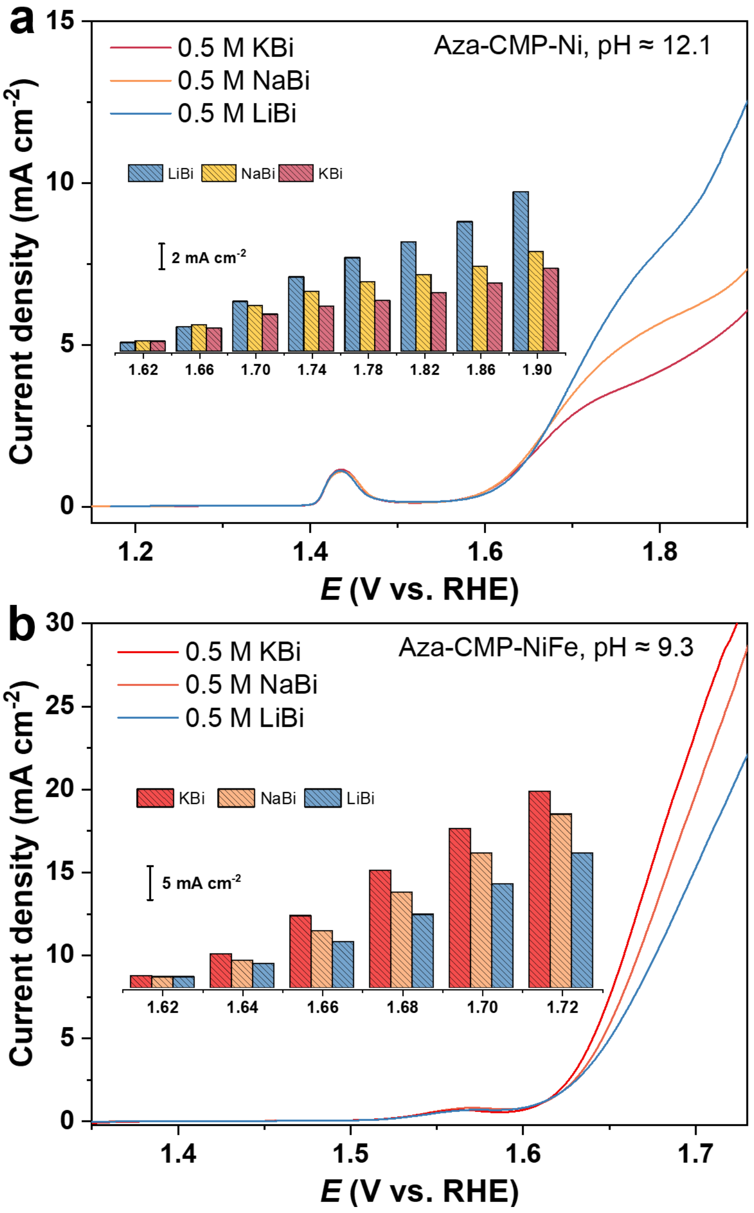

Supplementary Fig. 87 Cation-activity dependency in weak alkaline conditions. (a) LSV curves of Aza-CMP-Ni in 0.5 M KBi, NaBi, and LiBi with a scan rate of 10 mV s^{-1}. (b) LSV curves of Aza-CMP-NiFe in 0.5 M KBi, NaBi, and LiBi with a scan rate of 10 mV s^{-1}. Electrolyte pH was controlled by using concentrated Fe-free KOH, NaOH, or LiOH. Inset: comparison of catalytic current at different potentials.

Supplementary Discussion 6

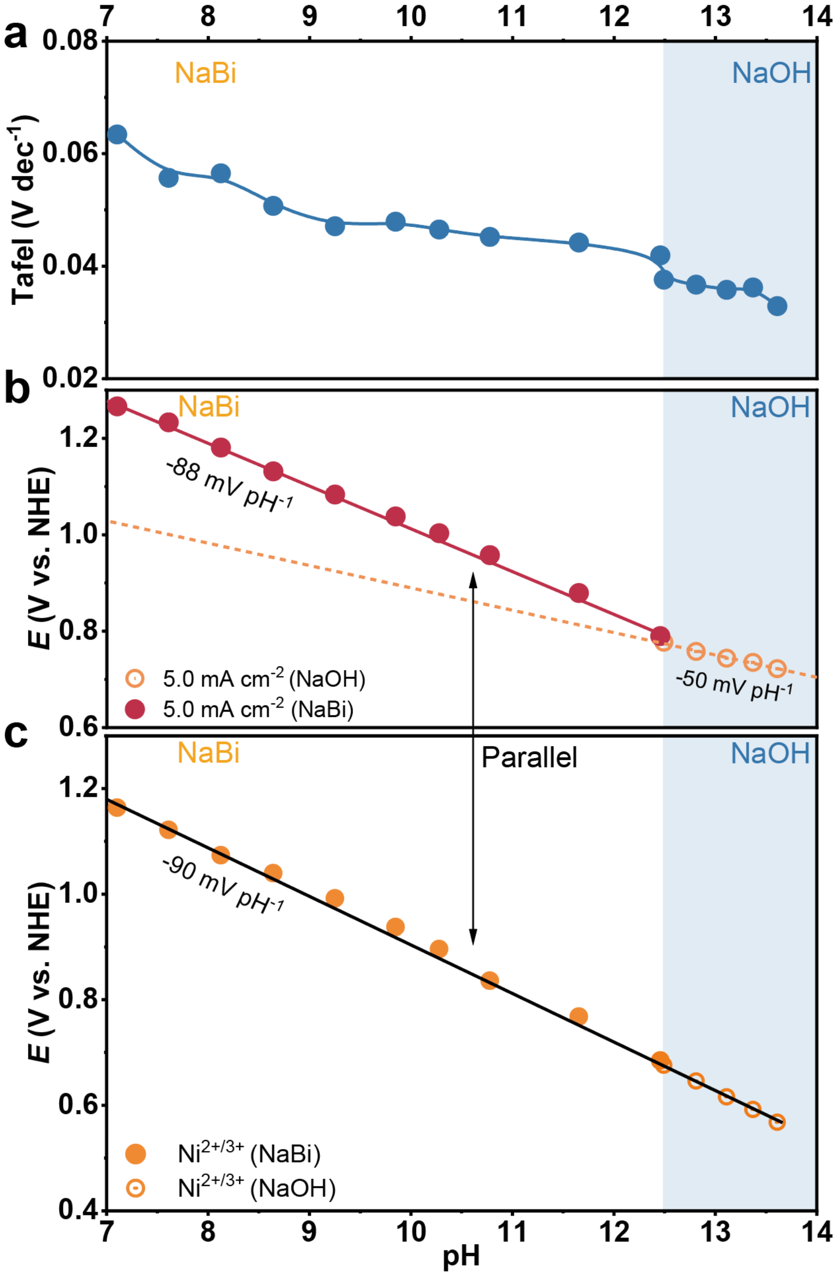

Supplementary Fig. 88 Water oxidation kinetics of Aza-CMP-NiFe in different pH conditions. (a) The relationship between Tafel slope and electrolyte pH, the Tafel slope calculation is presented in ***Supplementary Fig. 89***. (b) pH-dependent OER activity under extended pH range. The data are sourced from ***Supplementary Figs.*** *74* and *77*. (c) Pourbaix diagram of Aza-CMP-NiFe under the same pH range for direct comparison. The data are sourced from ***Supplementary Figs.*** *72* and *74*.

Supplementary Discussion 6

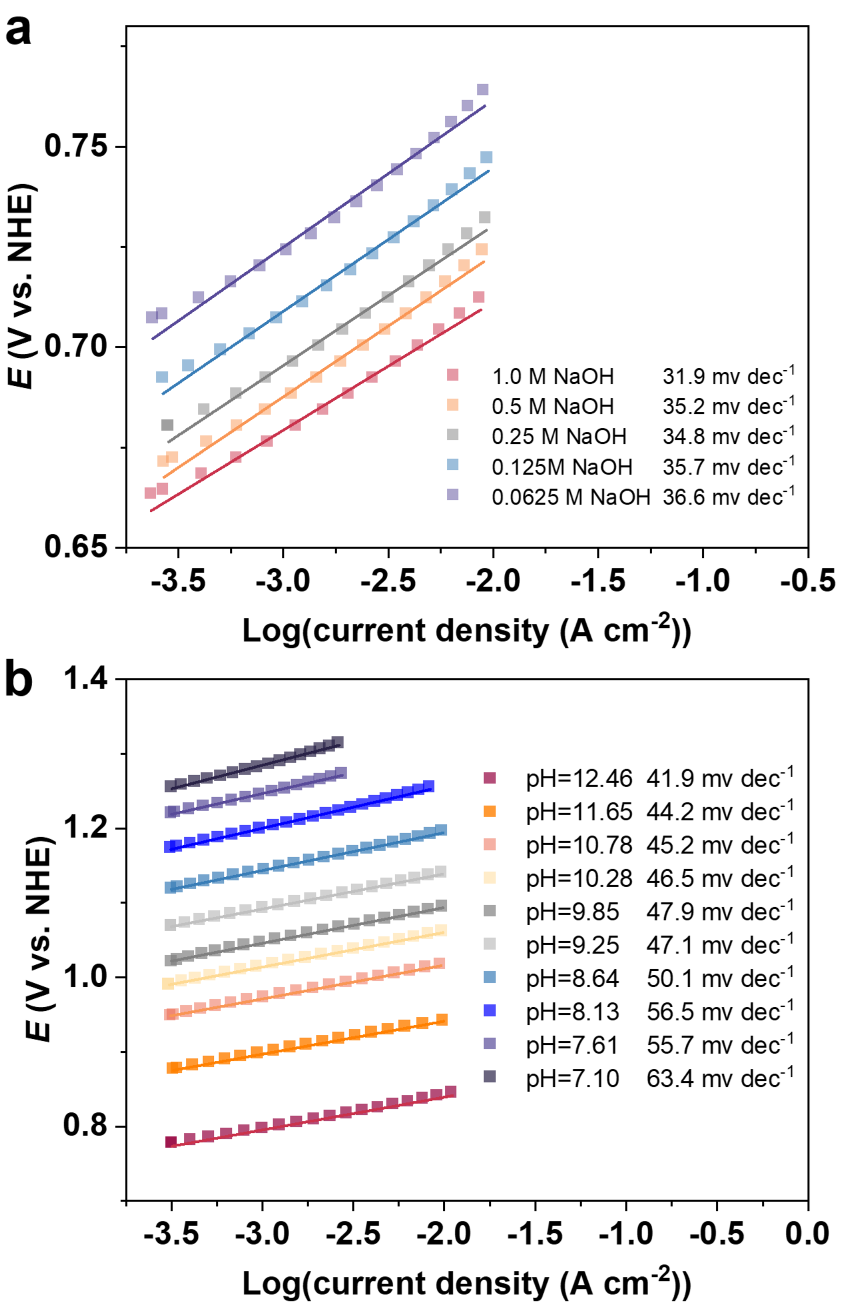

Supplementary Fig. 89 Tafel slopes of Aza-CMP-NiFe in different pH conditions. Tafel slopes extracted from corresponding LSV curves of Aza-CMP-NiFe. (a) NaOH solutions. (b) Borate buffered solutions.

Supplementary Discussion 6

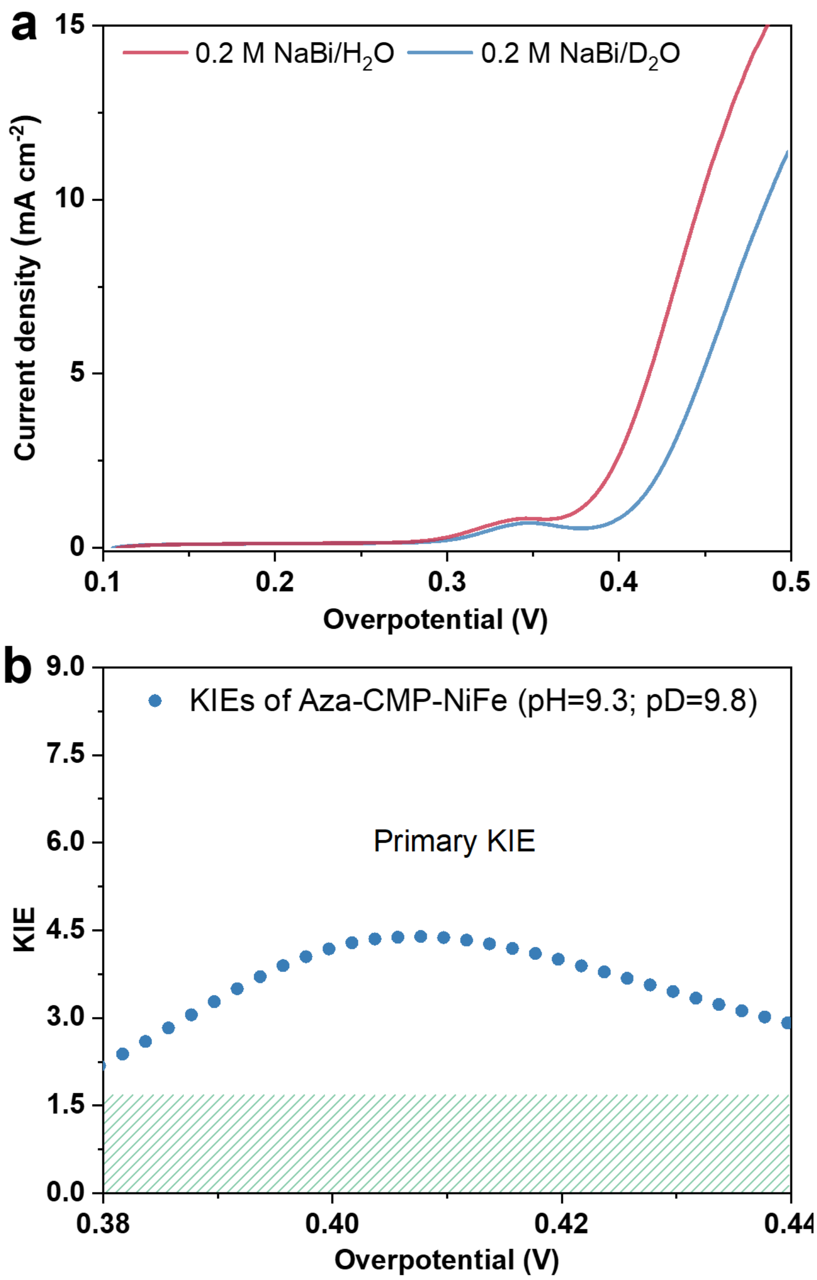

Supplementary Fig. 90 KIE measurements in weak alkaline conditions. (a) LSV curves of Aza-CMP-NiFe in 0.2 M NaBi/D_2O and NaBi/H_2O solutions (scan rate: 10 mV s^{-1}) and (b) corresponding KIEs against potential. KIE values were determined by ***Supplementary Eqn. 6***.

Supplementary Discussion 6

a

	Aza-CMP-Ni (pH>12.5)	Aza-CMP-Ni (pH<12.5)
$Ni^{2+/3+}$	**$1H^+/1e^-$**	**$2H^+/1e^-$**
$Ni^{3+/4+}$	**$1H^+/1e^-$**	**$1H^+/1e^-$**
OER-pH dependency	**-123 mV pH^{-1}**	**0 mV pH^{-1}**
KIEs	**Secondary**	**Primary**
Anion effect	✓	✓
RDS	**WNA-ET**	**WNA-PT**

b

	Aza-CMP-NiFe (Strong alkaline)	Aza-CMP-NiFe (Weak alkaline)
$Ni^{2+/3+}$	**$3H^+/2e^-$**	**$3H^+/2e^-$**
$Ni^{3+/4+}$	**N/A**	**N/A**
OER-pH dependency	**-52 mV pH^{-1}**	**-88 mV pH^{-1}**
KIEs	**Primary**	**Primary**
Anion effect	✓	✓
RDS	**WNA**	**WNA**

Supplementary Fig. 91 Comparison of kinetic phenomena and effects under different pH conditions. (a) Aza-CMP-Ni and (b) Aza-CMP-NiFe.

Supplementary Discussion 7

Supplementary Discussion 7: Proton inventory, anion concentration-activity relationship, and Tafel slope

7.1 Overpotential calculation for proton inventory measurement

The thermodynamic driving force of a given reaction varies with electrolyte acidity when pH-independent reference electrodes such as Ag/AgCl and Hg/HgO are used. The slight difference between pOH and pOD (due to the volume error introduced during electrolyte preparation) may lead to errors in the calculated overpotentials. To avoid potential errors caused by concentration differences when using the Hg/HgO reference electrode, the reference electrode was directly calibrated with the reversible hydrogen electrode (RHE)/reversible deuterium electrode (RDE) in as-prepared 1.0 M NaOH and NaOD, respectively. As shown in ***Supplementary Fig. 92***, the difference between Hg/HgO and RHE and RDE 1.0 M NaOH and NaOD was measured to be 0.905 V and 0.937 V, respectively. The overpotentials could be calculated using the following equations:

$$\eta^{D_2O} = E^{read}_{Hg/HgO} + E^{Hg/HgO}_{RDE} - 1.262\ V = E^{read}_{Hg/HgO} + 0.937\ V - 1.262\ V = E^{read}_{Hg/HgO} - 0.325\ V \quad (11)$$

$$\eta^{H_2O} = E^{read}_{Hg/HgO} + E^{Hg/HgO}_{RHE} - 1.229\ V = E^{read}_{Hg/HgO} + 0.905\ V - 1.229\ V = E^{read}_{Hg/HgO} - 0.324\ V \quad (12)$$

The difference of overpotentials in 1.0 M NaOD/D_2O and 1.0 M NaOH/H_2O is approximately 0.001 V, which can be neglected; therefore, it is reasonable to compare the current densities j_{H2O} and j_{D2O} at the same potential by using a Hg/HgO reference electrode (vs. Hg/HgO) for the calculation of kinetic isotopic effects with OH/OD mixed solutions.

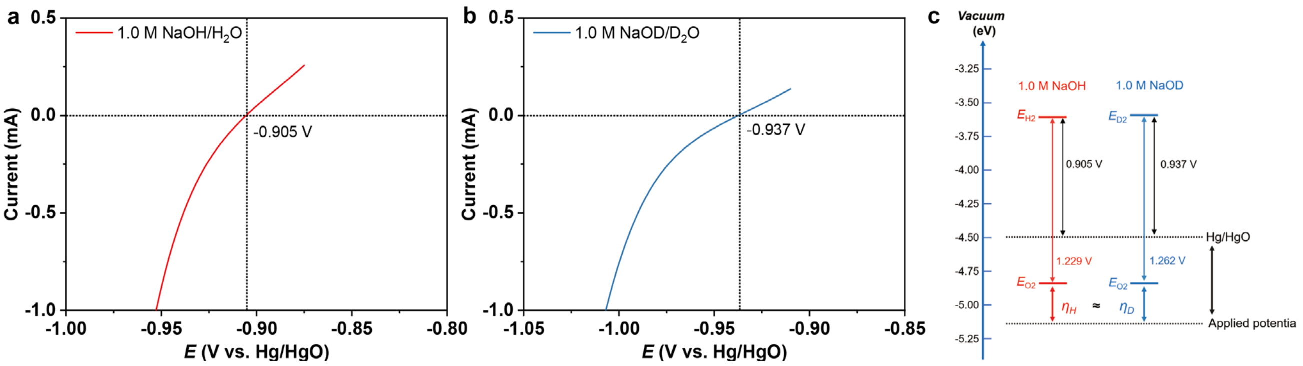

Supplementary Fig. 92 Overpotential calculation in NaOH/NaOD solutions. Current-potential curves of Pt mesh in H_2-saturated (a) 1.0 M NaOH and (b) 1.0 M NaOD solutions, used for calibration of the Hg/HgO electrode with respect to RHE/RDE. Scan rate: 10 mV s^{-1}. (c) Overpotential calculation for water oxidation in 1.0 M NaOH/NaOD solutions, the difference is negligible when using Hg/HgO as a reference electrode.

For the proton inventory measurements employing NaBi buffer, the pH of 0.5 M NaBi/H_2O solutions was found to be 9.31, while the pD of 0.5 M NaBi/D_2O solutions was recorded as 9.86. The overpotentials were calculated using the equations provided:

$$\eta^{D_2O} = E^{read}_{Ag/AgCl} + E^{Ag/AgCl}_{RDE} - 1.262\ V = E^{read}_{Ag/AgCl} + 0.197 + 0.059 \times 9.86 - 1.262 = E^{read}_{Ag/AgCl} - 0.4832\ V \quad (13)$$

$$\eta^{H_2O} = E^{read}_{Ag/AgCl} + E^{Ag/AgCl}_{RHE} - 1.229\ V = E^{read}_{Ag/AgCl} + 0.197 + 0.059 \times 9.31 - 1.229 = E^{read}_{Ag/AgCl} - 0.4827\ V \quad (14)$$

The difference in overpotentials between 0.5 M NaBi/D_2O and 0.5 M NaBi/H_2O solutions is negligible. Thus, it is valid to compare the current densities, j_{H2O} and j_{D2O}, at identical potentials when

utilizing an Ag/AgCl reference electrode (vs. Ag/AgCl) for determining kinetic isotope effects in H_2O/D_2O mixed solutions.

Similarly, for the proton inventory measurements employing NaPi buffer, the pH of 0.5 M NaPi/H_2O solutions was found to be 12.54, and the pD of 0.5 M NaPi/D_2O solutions was adjusted to 13.09. Calculations of overpotentials were conducted as follows:

$$\eta^{D_2O} = E^{read}_{Ag/AgCl} + E^{Ag/AgCl}_{RDE} - 1.262\ V = E^{read}_{Ag/AgCl} + 0.197 + 0.059 \times 13.09 - 1.229 = E^{read}_{Ag/AgCl} - 0.292\ V \quad (15)$$

$$\eta^{H_2O} = E^{read}_{Ag/AgCl} + E^{Ag/AgCl}_{RHE} - 1.229\ V = E^{read}_{Ag/AgCl} + 0.197 + 0.059 \times 12.54 - 1.229 = E^{read}_{Ag/AgCl} - 0.292\ V \quad (16)$$

A negligible difference in overpotentials between 0.5 M NaPi/D_2O and 0.5 M NaPi/H_2O allows for a direct comparison of current densities at the same potential using an Ag/AgCl reference electrode.

7.2 Proton inventory data for Aza-CMP-NiFe

Electrochemical proton inventory studies were carried out using the above-mentioned solutions to investigate the involvement of proton-containing intermediates and provide further support for the identification of the RDS. The findings are presented in ***Supplementary Figs. 94-96***, with results in the main text shown as normalized curves by normalizing the decay of the k_n/k_0 value.

The participation of proton relays (e.g., nearby uncoordinated N sites) in basic electrolytes is excluded since the pyridine-like Z-sites mainly contributed to an inverse isotopic effect, leading to the expectation of a dome-shaped curve under dominant medium effects.[73] For the variations from a deeply bowl-shaped curve to a semi-straight-shaped curve in this case of water oxidation, the most probable complex model, involving more than one reaction process, is the "two steps in succession" model wherein both steps significantly impact the observed rate (***Supplementary Fig. 93***). The formation of MOOH from M=O and solvation water is a key contributor to the pronounced cation effects, with the proton inventory curve demonstrates a semi-linear trend. Further deprotonation of MOOH at intramolecular relay sites to form MOO species also plays a critical role in the RDS, resulting in enhanced curvature in the bowl-shaped proton inventory plots. Meanwhile, the effect of the external Brønsted base (anion) on deprotonation, facilitated by the solvation environment, is modulated by the values of k_B and k_R. Consider such a case (analogous to the form of the Ping Pong Bi Bi mechanism) where more than one transition state or reactant state may be significant for a particular kinetic parameter,[74] it's posited that k_B and k_R steps will generally contribute to isotope effects. The resultant isotope effects represent a weighted average, defined by weighting factors (w), according to the virtual transition state approach.[74, 75] Consequently, the apparent structure of a transition state derived from the measured isotopic effects is the structure of an imaginary species (TSv), which is, in fact, the weighted average of one or more real transition states (TS1 and TS2). The configuration of the theoretical curve is elaborated upon in ***Supplementary Notes 5***.

Upon the WNA mechanism, the acid dissociation constant of buffer ions indicates base strength, which is closely related to the value of k_B in solution-mediated APT. Under acidic conditions, water directly acts as a poor proton acceptor in the reaction (***Supplementary Eqn. 17***), even if $pK_a(H_3O^+) = -1.7$, indicating a small k_B and sluggish kinetics. In a buffered solution, k_B demonstrates a dependency on the pK_a value of buffer ions, as evidenced by k_B values of 3.8, 10.3, and 48 $M^{-1}\ s^{-1}$ for H_2PO^{4-} (pK_a = 2.2), acetate (pK_a = 4.8), and HPO_4^{2-} (pK_a = 7.2), respectively, with $[Ru^V(tpy)(Mebim\text{-}py)(O)]^{3+}$ WOC.[76] At higher pH, despite the kinetically significant concentration of OH^-, it should not be treated as an independent molecule in the solution reaction since OH^- in solution exists within a hydrogen-bonded network, solvated by water molecules, and potentially coordinating with cations. In the context of alkaline OER, H_2O and OH^- are functionally equivalent reactants due to the "shared" proton,[77, 78]

as illustrated in ***Supplementary Eqn. 19***. Given that the transferred proton can be conceptualized as being equally distributed between two molecules,[79] OH^- emerges as an excellent Brønsted-base with a high pK_a of 15.7 (***Supplementary Eqn. 20***), analogous to the scenario presented in ***Supplementary Eqn. 18*** for buffered conditions. It is worth mentioning that the microkinetic model for pH- and potential-dependent oxygen evolution also supports that the OER occurs primarily via water oxidation rather than hydroxide oxidation, even under alkaline pH conditions.[80] Based on the above analysis, the changes in proton inventory curves in different buffers are caused by varying k_B values.

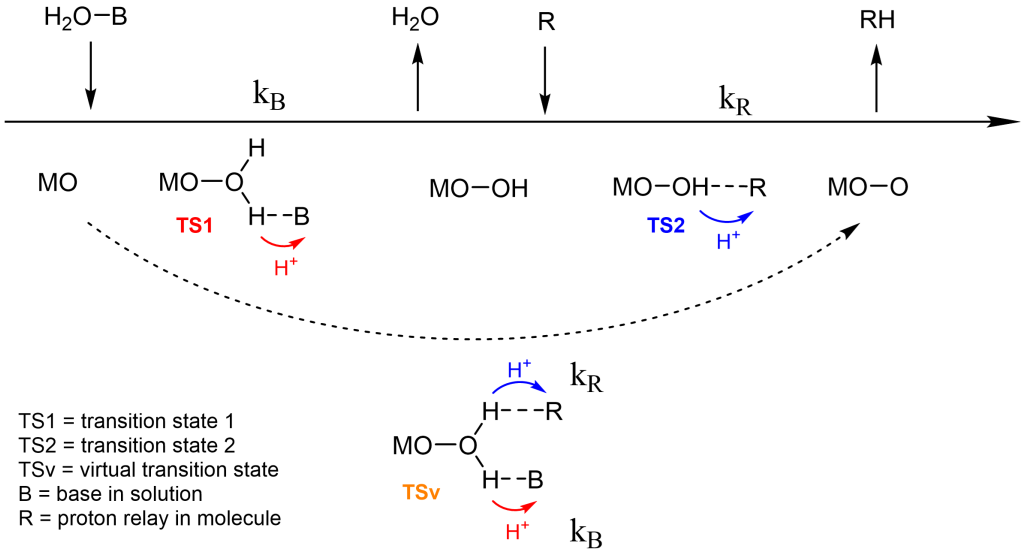

Supplementary Fig. 93 Linear reactions with two succession steps to form M–OO structure via WNA.

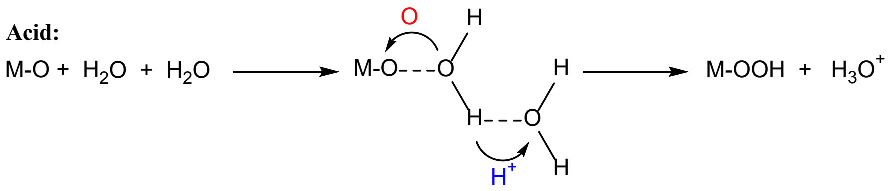

(17)

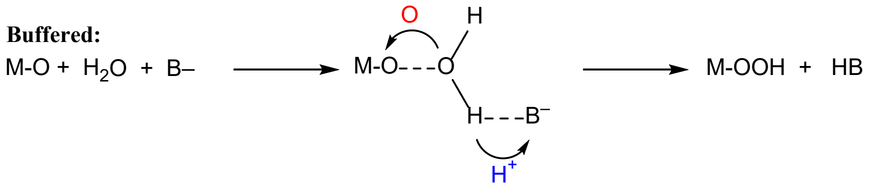

(18)

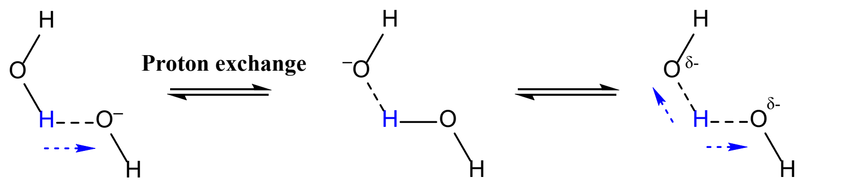

(19)

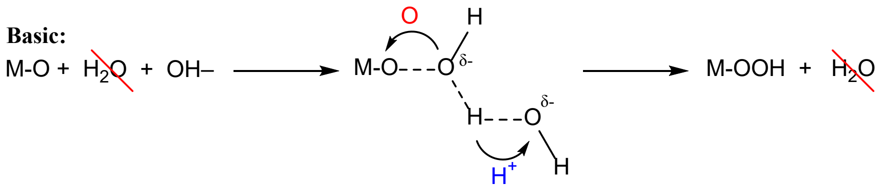

(20)

Supplementary Discussion 7

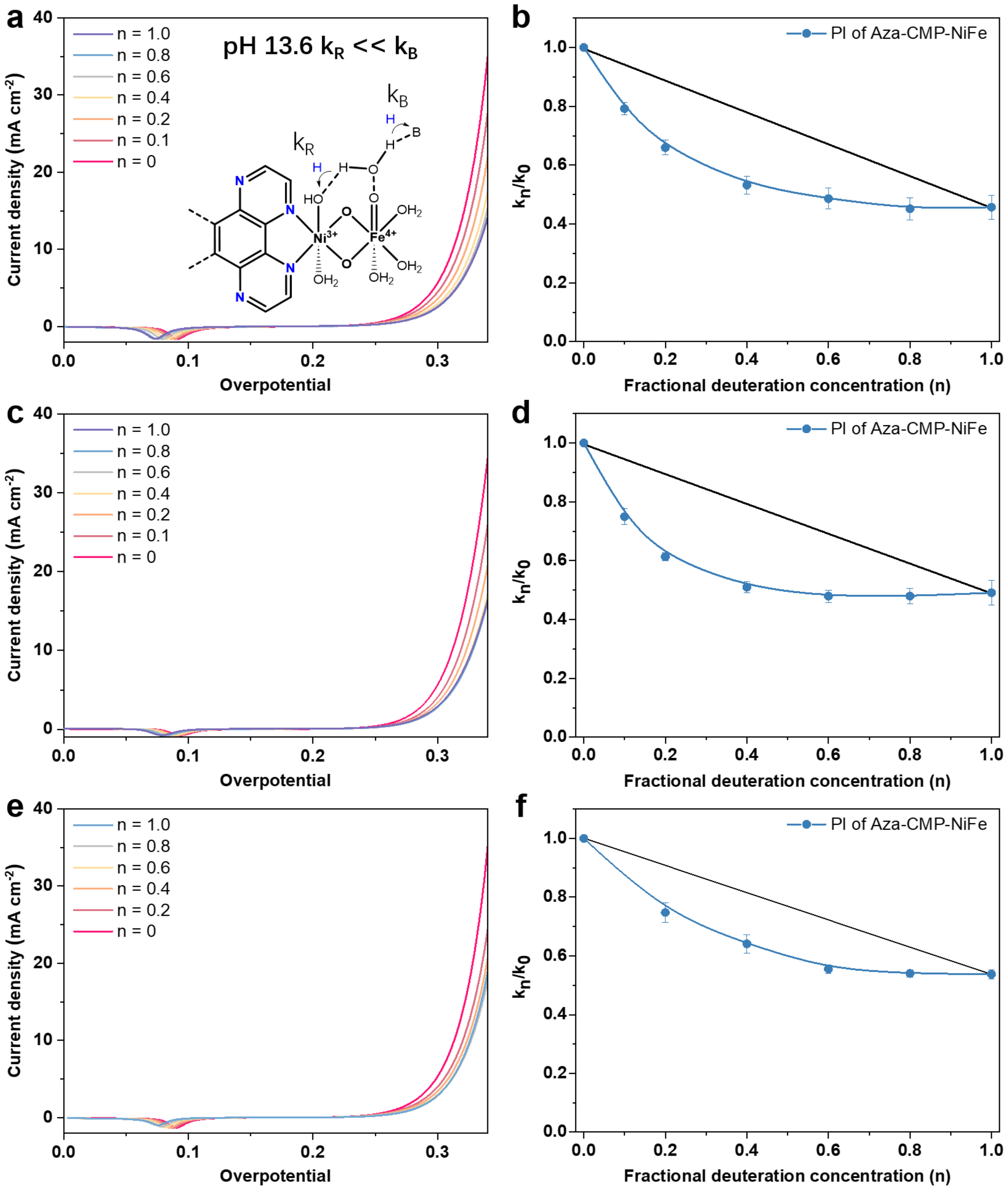

Supplementary Fig. 94 Proton inventory in NaOH/NaOD solutions. (a, c, e) LSV curves of Aza-CMP-NiFe in mixed solutions of 1.0 M NaOH in H_2O and 1.0 M NaOD in D_2O with different fractional deuteration concentrations (n) (scan rate: 10 mV s^{-1}). (b, d, f) The plots of k_n/k_0 as a function of n, where n = [D]/([D] + [H]) and at a certain potential were abbreviated as k_n, $k_0 = k_{H2O}$. The experiments were repeated three times to ensure the reliability of the data. Inset: schematic RDS. The centers of the error bars represent the average KIE values measured under different applied potentials (obtained from the LSV curves, comprising 100 data points across an OER window from 0.26 to 0.36 V). The error bars indicate the standard deviation calculated from these 100 potentials, reflecting the variability in the KIE values.

Supplementary Discussion 7

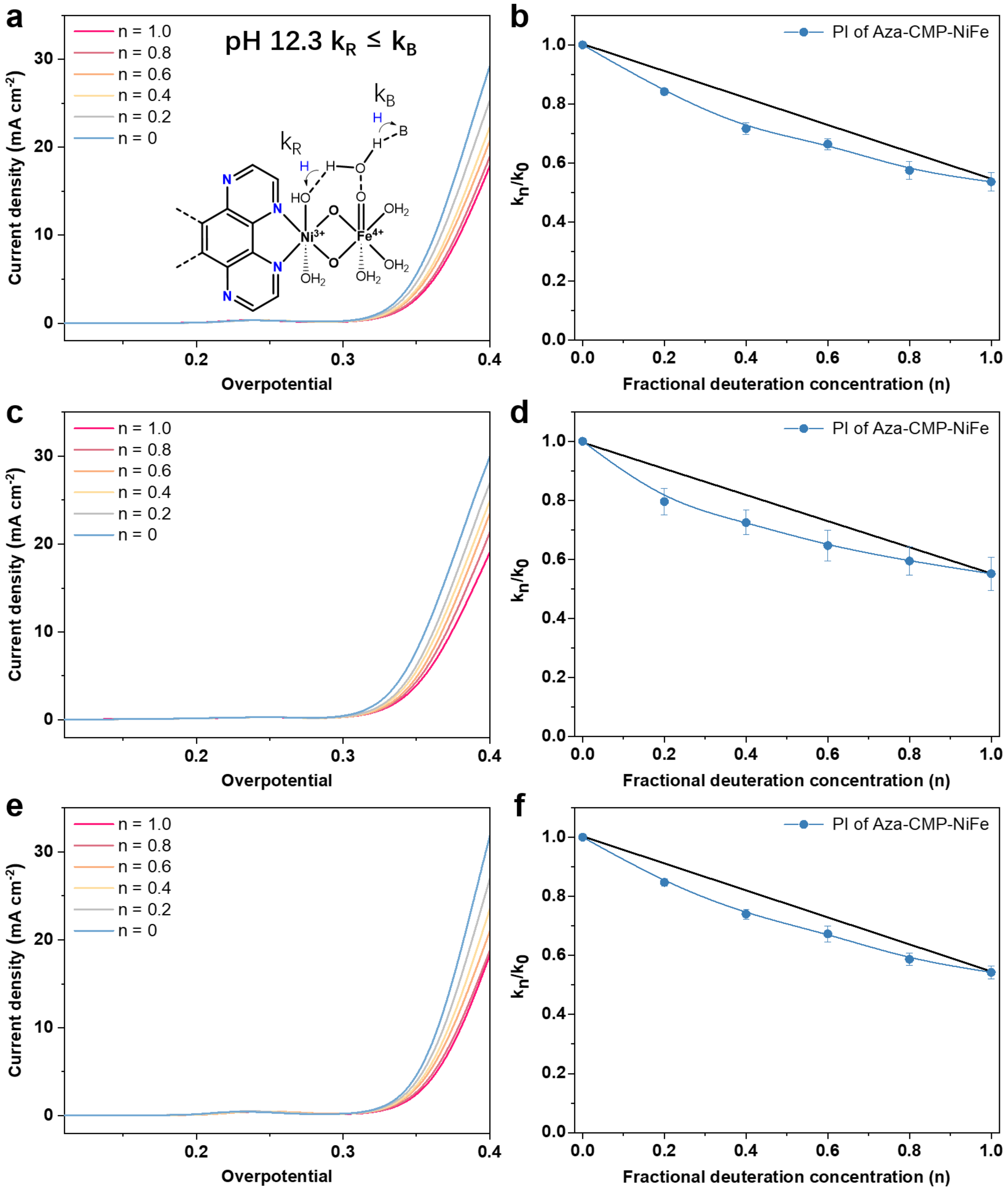

Supplementary Fig. 95 Proton inventory in NaPi solutions. (a, c, e) LSV curves of Aza-CMP-NiFe in mixed solutions of 0.5 M NaPi in H_2O/D_2O with different fractional deuteration concentrations (n) (scan rate: 10 mV s^{-1}). (b, d, f) The plots of k_n/k_0 as a function of n, where n = [D]/([D] + [H]) and at a certain potential were abbreviated as k_n, $k_0 = k_{H2O}$. The experiments were repeated three times to ensure the reliability of the data. Inset: schematic RDS. The centers of the error bars represent the average KIE values measured under different applied potentials (obtained from the LSV curves, comprising 100 data points across an OER window from 0.30 to 0.40 V). The error bars indicate the standard deviation calculated from these 100 potentials, reflecting the variability in the KIE values.

Supplementary Discussion 7

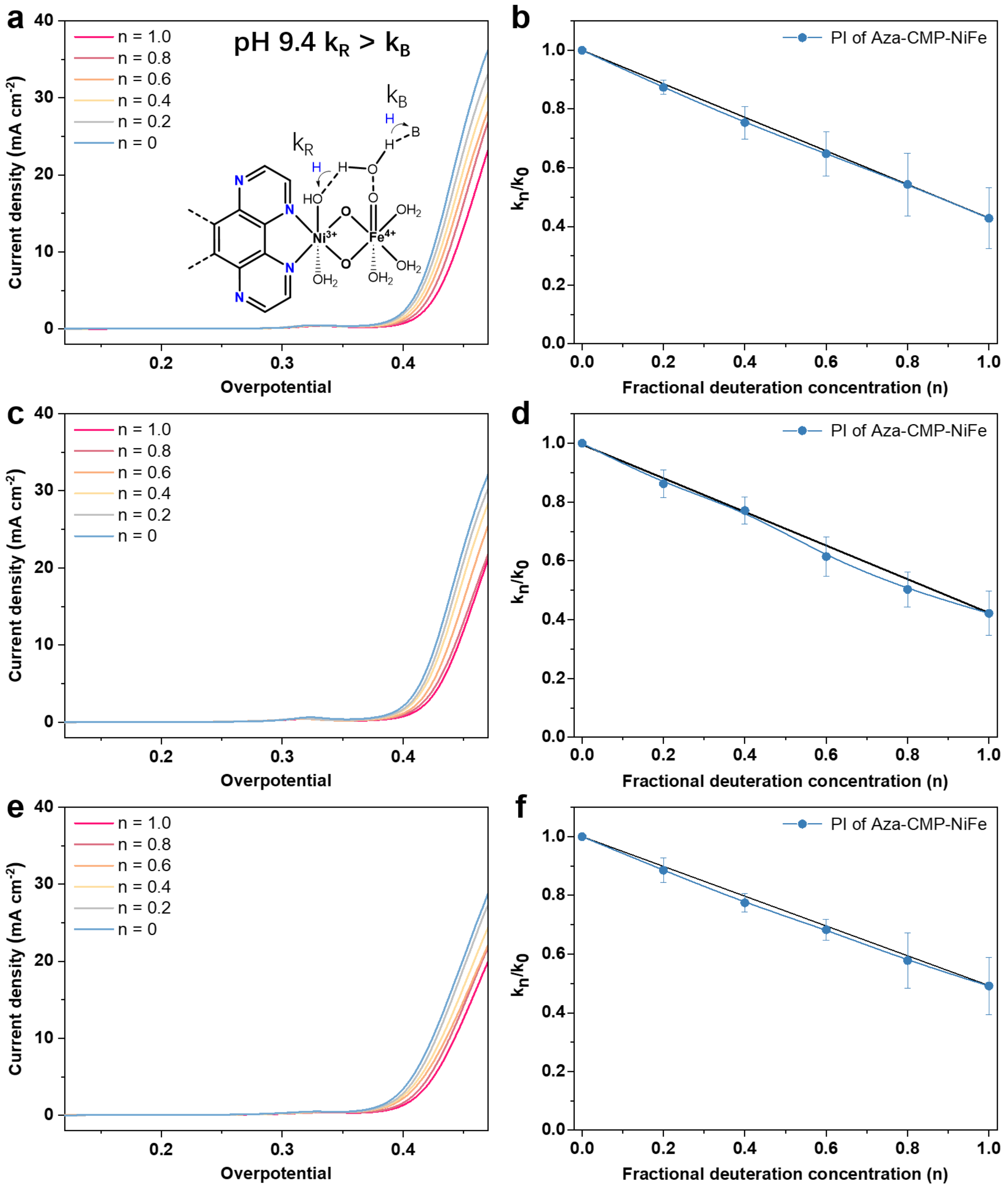

Supplementary Fig. 96 Proton inventory in NaBi solutions. (a, c, e) LSV curves of Aza-CMP-NiFe in mixed solutions of 0.5 M NaBi in H_2O/D_2O with different fractional deuteration concentrations (n) (scan rate: 10 mV s^{-1}). (b, d, f) The plots of k_n/k_0 as a function of n, where n = [D]/([D] + [H]) and at a certain potential were abbreviated as k_n, $k_0 = k_{H2O}$. The experiments were repeated three times to ensure the reliability of the data. Inset: schematic RDS. The centers of the error bars represent the average KIE values measured under different applied potentials (obtained from the LSV curves, comprising 100 data points across an OER window from 0.37 to 0.47 V). The error bars indicate the standard deviation calculated from these 100 potentials, reflecting the variability in the KIE values.

Supplementary Discussion 7

7.3 Proton inventory measurements under CO poisoning conditions

To further elucidate the synergistic effect between Ni and Fe centers, carbon monoxide (CO) was selected to inhibit the adsorption of H_2O/OH^- ligands on NiFe sites. The π acceptor CO ligand is capable of forming a bond with Ni^{2+} sites through π back-bonding, whereas the interaction between CO and Fe^{3+} is hindered by reduced electron density.[81] In the [NiFe]hydrogenase system, exogenously introduced CO has been observed to bind to the Ni atom at the Ni-Fe active site.[82] Hence, it is rational that CO could selectively substitute the axial H_2O/OH^- ligands on the Ni portion of the NiFe sites. Even if CO manages to bind to the Fe site, it would obstruct the O–O bond formation process at the Fe site and thus does not influence the property of ultimate statistical currents. The CV results of the CO-poisoned Aza-CMP-NiFe are presented in ***Supplementary Fig. 97a***. Following a 30-minute purging of the electrode and electrolytes with CO, the OER currents on the resultant NiFe sites exhibit noticeable degradation, suggesting the bonding between CO ligands and the metal redox center. The anodic shift of the $Ni^{2+/3+}$ redox potential indicates that the ligand exchange occurs on the Ni^{2+} metal center. The PCET feature of the CO-treated sample is investigated by examining the pH dependency of the $Ni^{2+/3+}$ redox couple (***Supplementary Fig. 97b***). A pH-dependent slope of -74.9 mV pH^{-1} demonstrates that the presence of the axial CO ligand significantly diminishes the number of transferred protons in the process (***Supplementary Fig. 97c***). As schematically depicted in ***Supplementary Fig. 97d***, the replacement of axial OH_2 ligands with CO ligands results in a possible $2H^+/2e^-$ process during the formation of Ni^{3+} species, further validating the successful bonding between the Ni center and CO ligands.

With the Ni center blocked by CO ligands, CO-poisoned electrochemical proton inventory studies were further conducted under alkaline conditions (1.0 M NaOH/NaOD) to investigate the participation of the adjacent Ni center in NiFe sites. As shown in ***Supplementary Fig. 98***, the downward arc of the PI curves is notably diminished, moving closer to a linear relationship. This suggests that Ni sites play a pivotal role in the proton transfer of the RDS. As OH sites are replaced by CO, protons involved in the O–O formation process cannot be transferred to the adjacent Ni sites (***Supplementary Fig. 98a***). This results in NiFe sites effectively following a single-site water oxidation catalytic process, wherein water nucleophilically attacks Fe^{4+}=O to form the Fe^{3+}OOH structure, considered as the RDS. In other words, CO poisoning results in a transformation of the RDS from a "two steps in succession" model to the "$MOOH_2$" step, yielding a more linear PI curve. The aforementioned experimental results further support our proposed water oxidation mechanism at Ni-Fe sites; that is, the Fe center is the active site for O–O bond formation, the Ni centers act as intramolecular proton acceptors, and the actual RDS of water oxidation is the reaction from Fe^{4+}=O to Fe^{4+}OO, involving an intermolecular transfer of $1H^+/1e^-$ and an intramolecular transfer of $1H^+/1e^-$ from the Fe site to the Ni site.

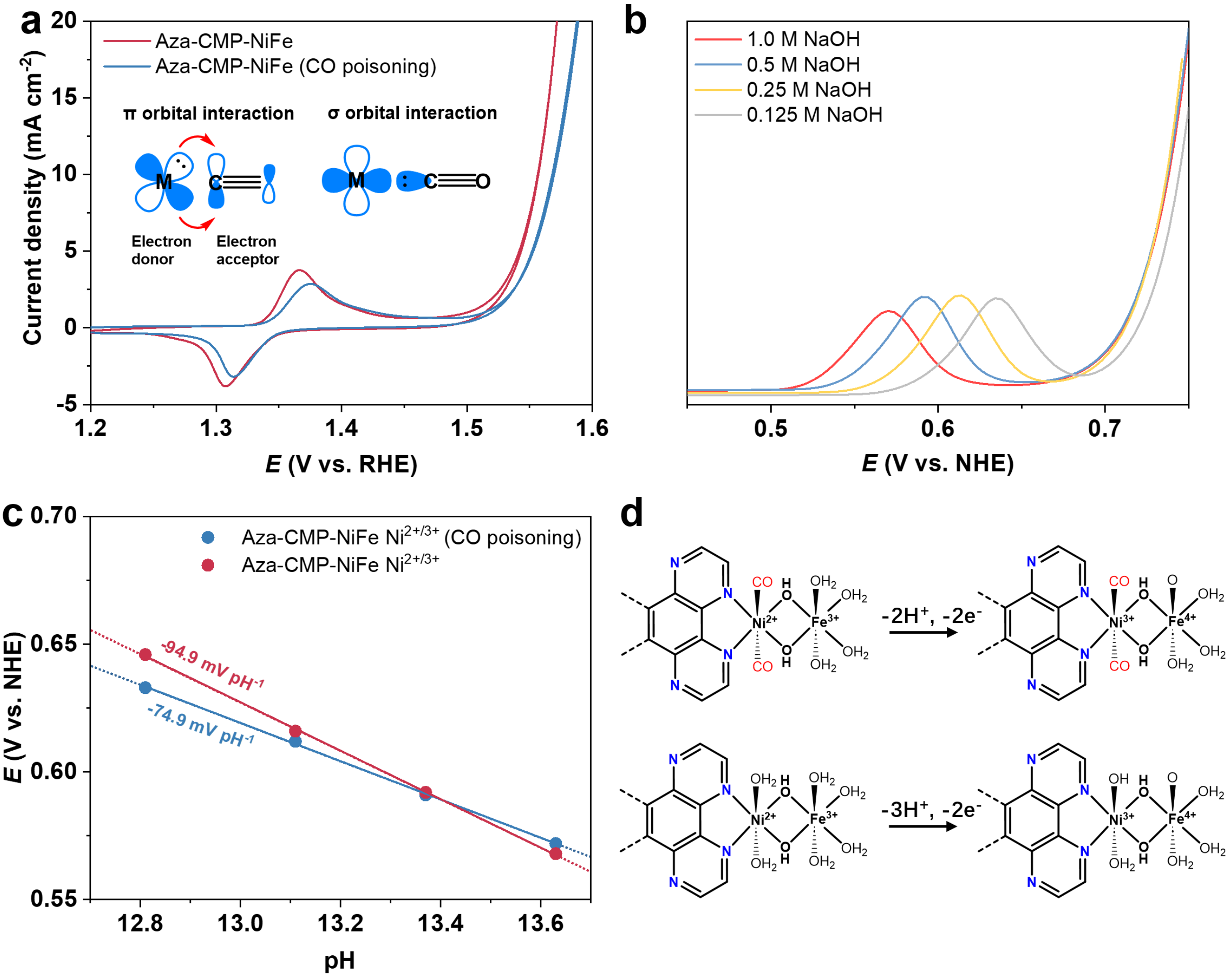

Supplementary Fig. 97 Redox features under CO poisoning conditions. (a) CV curves of CO-poisoned Aza-CMP-NiFe (scan rate: 50 mV s^{-1}). (b) DPV curves of CO-poisoned Aza-CMP-NiFe at different pH of electrolytes and (c) corresponding Pourbaix diagram. (d) Schematic PCET processes of $Ni^{2+/3+}$ redox couple after CO treatment.

Supplementary Discussion 7

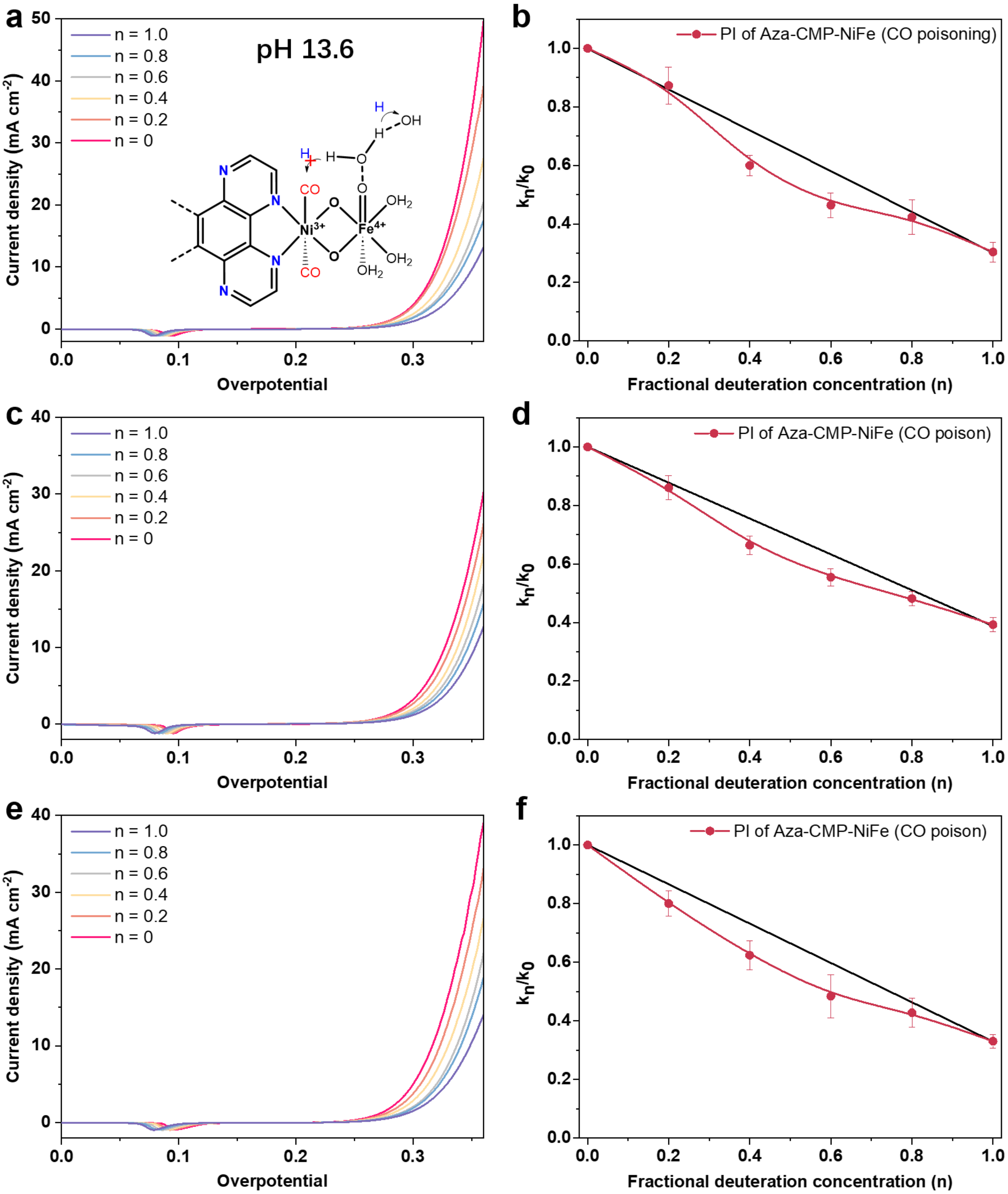

Supplementary Fig. 98 Proton inventory under CO poisoning conditions. (a, c, e) LSV curves of CO-poisoned Aza-CMP-NiFe in mixed solutions of 1.0 M NaOH in H_2O and 1.0 M NaOD in D_2O with different fractional deuteration concentrations (n) (scan rate: 10 mV s^{-1}). (b, d, f) The plots of k_n/k_0 as a function of n, where n = [D]/([D] + [H]) and at a certain potential were abbreviated as k_n, $k_0 = k_{H2O}$. The experiments were repeated three times to ensure the reliability of the data. Inset: schematic RDS. The centers of the error bars represent the average KIE values measured under different applied potentials (obtained from the LSV curves, comprising 100 data points across an OER window from 0.26 to 0.36 V). The error bars indicate the standard deviation calculated from these 100 potentials, reflecting the variability in the KIE values.

Supplementary Discussion 7

7.4 Anion (base) effects for Aza-CMP-NiFe

To gain deeper insight into the intermediate states of the RDS, the role(s) of mediated APT in Ni-Fe sites catalyzed water oxidation was investigated by adding different concentrations of anion ions (Bi, Pi, and OH^-) at varying pH. The property of the proton acceptor significantly affects the catalytic behavior in APT-controlled O–O bond formation. The diffusion-controlled solution APT process is influenced by the concentration of the external proton acceptor in the electrolyte, and first-order reactions on anion concentrations are widely observed in catalyst-modified electrodes when the RDS for the OER proceeds through the APT pathway.[12, 83, 84, 85, 86] In the context of the virtual transition state, the presence of external proton acceptor-related TS1 and the unrelated TS2 renders the overall reaction order with respect to anion concentration more complex than a mere first-order reaction. The acid dissociation constant of the buffer ions, which plays a pivotal role in affecting the reaction rate (k_B) in solution-mediated APT mechanisms, essentially determines the order of reactions based on the dynamic between k_B and k_R values, that is, determined by the specific anion species in the solution.

Considering the forward direction of the two-step illustrated previously (***Supplementary Fig. 93***), the forward reaction rates, denoted by k_v, are as follows:[87]

$$k_v = \frac{k_R k_B[R][B] - k_{-R}k_{-B}[MOOH][MOO]}{k_R[R] + k_B[B] + k_{-R}[MOO] + k_{-B}[MOOH]} \qquad (21)$$

When assumed that the reverse reaction rate for each step is sufficiently negligible and the concentration of R sites remains constant, consequently, the term $k_R[R]$ can be considered independent of concentration, allowing for the reformulation of the preceding equation as follows:

$$k_v = \frac{k_R k_B[B]}{k_R + k_B[B]} \qquad (22)$$

Therefore, the correlation between the observed reaction rate (k_{obs}) and the concentration of anions can be outlined as follows:

$$k_{obs} = \left[\frac{jA}{4F\Gamma}\right]_\eta = k_{H_2O} + k_v \qquad (23)$$

$$\left[\frac{jA}{4F\Gamma}\right]_\eta = k_{H_2O} + \frac{k_R k_B[B]}{k_R + k_B[B]} \qquad (24)$$

Where A is the surface area (cm^2) of the electrode, F is the Faraday constant, Γ is the number of electroactive species (mol cm^{-2}), j is the current density (mA cm^{-2}), k_{H2O} is the fundamental rate with water molecules as proton relay, and [B] is the concentration of additional anions (mol L^{-1}). The reaction order for additional anions (external bases) in solutions, denoted as ρ[B], can be determined using the following equation:

$$\rho_{[B]} = \left[\frac{\partial \log j}{\partial \log[B]}\right]_\eta \qquad (25)$$

The simulation in ***Supplementary Fig. 99*** demonstrates that variations in the anion concentration affect the shape of the activity-concentration curve based on the k_B value. A smaller k_B results in a curve that more closely approximates linearity, aligning the reaction order approaching 1. Conversely, a larger k_B tends to shift the reaction order toward 0.

Experimental studies on the effects of anions (base) were conducted using various solutions with differing k_B values, while the ionic strength was standardized using $NaSO_4$ (given that the pK_a of sulfate is approximately −3, it is considered an innocent species in proton shuttling). In the case of borate buffer with a pK_a value of 9.2, the anion effects are significant. The ρ[Bi] value for Ni-Fe sites ranges between approximately 0.3-0.6 across the catalytic potential range under pH 9.2 electrolyte (***Supplementary Fig. 100a***). Conversely, under pH 12.3 conditions, ρ[Pi] for Ni-Fe sites significantly decreases to about 0.1-0.3, as shown in ***Supplementary Fig. 100b***. Notably, under strong alkaline

conditions, the $\rho[OH^-]$ value approaches 0 (***Supplementary Fig. 100c***), and two orders of magnitude improve the TOF values compared to the borate environment (***Supplementary Fig. 100d***). The observed variation in reaction order depending on the buffer type aligns perfectly with the theoretical predictions, thereby reinforcing the hypothesis of a two-step competition in the RDS, in accordance with the virtual transition states concept.

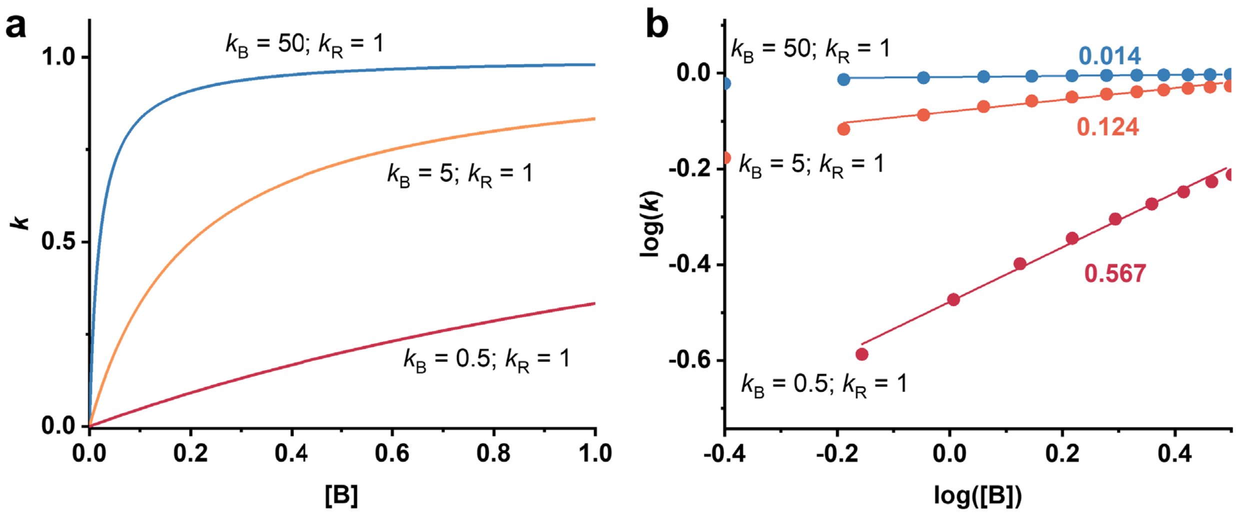

Supplementary Fig. 99 Theoretical anion dependency. (a) Anion concentration-activity relationship derived from ***Supplementary Eqn. 24*** and (b) corresponding $\rho[B]$ with different rate constants calculated using ***Supplementary Eqn. 25***. The parameter k_{H2O} was set to 0.

Supplementary Discussion 7

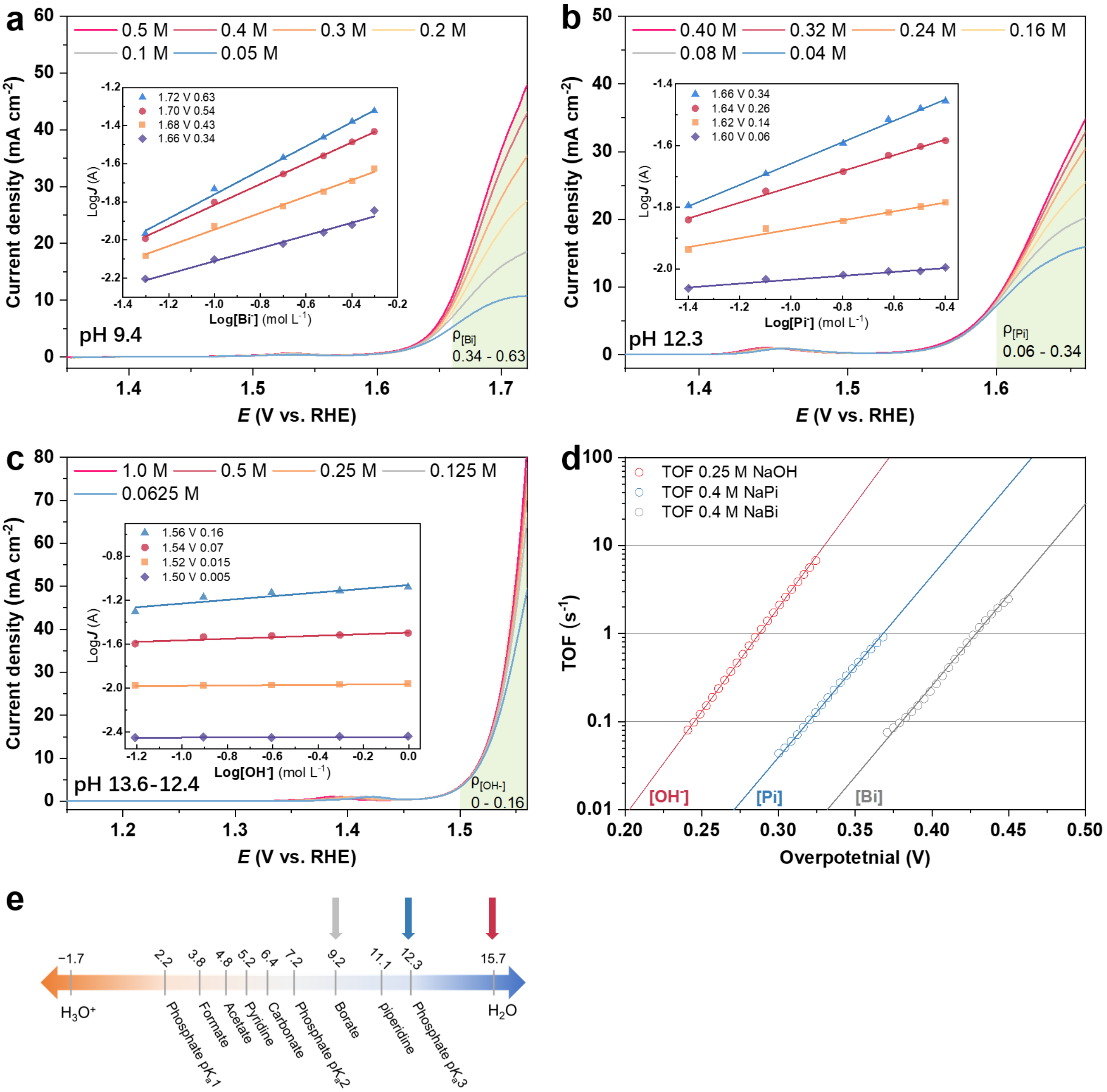

Supplementary Fig. 100 Anion effects for Aza-CMP-NiFe. LSV curves and corresponding reaction orders of Aza-CMP-NiFe as a function of (a) [Bi], (b) [Pi], and (c) [OH⁻]. The inset figures illustrate the reaction order for additional anions at different potentials. (d) TOF values comparison with different anion species. (e) Acid dissociation constant of water and typical buffer ions.

Supplementary Discussion 7

7.5 Tafel slope

In the kinetic model of the PCET reaction established by Marcus theory, the pre-factor is influenced by the vibronic coupling terms between the reactant and product states. Additionally, the total reaction free energy depends on the distance *R* from the electrode, the applied potential *E*, and the electronic energy level ε in the electrode relative to the Fermi level.[88] To provide a clearer numerical illustration, we calculated all possible Tafel slopes using a standardized formula for multi-electron reactions. This formula links the Tafel slope to the transfer coefficient and the number of electrons involved:[89]

$$\frac{\partial\eta}{\partial logj} = \frac{2.303\mathrm{R}T}{\mathrm{F}} \times \frac{1}{n_\mathrm{b} + \beta_f n_\mathrm{d}} \qquad (26)$$

$$\beta_f = \frac{1}{2}\left(1 + \frac{\mathrm{F}\eta}{2\lambda}\right) \qquad (27)$$

Where β_f is the symmetry coefficient for the forward reaction, F is the Faradaic constant, R is the ideal gas constant, *T* is temperature, n_b is the electrons transferred before the RDS, n_d is the electrons transferred during the RDS, λ is the reorganization energy of the acceptor species in solution, and η is the reaction overpotential. Under room temperature (298.15 K), the Tafel slope could be written as

$$\frac{\partial\eta}{\partial logj} = \frac{59\ \mathrm{mV\ dec^{-1}}}{n_\mathrm{b} + \frac{1}{2}\left(1 + \frac{\mathrm{F}\eta}{2\lambda}\right) n_\mathrm{d}} \qquad (28)$$

Reorganization energy can be divided into two parts:[90]

$$\lambda = \lambda_\mathrm{i} + \lambda_\mathrm{o} \qquad (29)$$

Where λ_i is inner shell reorganization energy and λ_o is inner shell reorganization energy. λ_i reflects the energy changes associated with the self-reorganization of the donor-acceptor (A-D) molecules. λ_o accounts for the energy changes due to the environmental reorganization, such as the orientation of solvent molecules. From a molecular perspective, since electron transfer typically occurs between delocalized molecules, λ_i is generally much smaller than λ_o. For single electron transfers, the outer shell reorganization energy can be calculated using the following equation:[90]

$$\lambda = \left[\frac{N_\mathrm{A}e^2}{8\pi}\left(\frac{1}{\varepsilon_{op}} - \frac{1}{\varepsilon_s}\right)\right]\left(\frac{1}{a_\mathrm{A}} + \frac{1}{a_\mathrm{D}} - \frac{2}{r}\right) \qquad (30)$$

Where ε_{op} is the optical dielectric constant of the solvent, ε_s is the static dielectric constant of the solvent, a_A is the diameter of the acceptor, a_D is the diameter of the donor, and *r* is the distance between the centers of the acceptor and donor, $2r = a_\mathrm{A} + a_\mathrm{D}$. For intramolecular proton-coupled electron transfer (PCET) reactions, the reorganization energy tends to be lower because these reactions do not directly involve the solvent environment, unlike intermolecular PCET reactions. Additionally, considering solely the proton transfer distance *R*, intramolecular transfers typically involve shorter distances compared to intermolecular reactions. Consequently, the lower reorganization energy in intramolecular PCET reactions contributes to an increased symmetry coefficient β_f and a reduction in the Tafel slope. In the experiment, the Tafel slope is influenced by multiple factors, including non-kinetic effects and changes in surface coverage due to pre-reactions.[91, 92] Therefore, using the Tafel slope alone to determine the reaction mechanism is generally unreliable. In our case, theoretically, an RDS controlled by TS1 is expected to exhibit a larger Tafel slope compared to an RDS governed by TS2 as discussed above. This theoretical prediction aligns with the experimental observations in ***Supplementary Fig. 88***, implying that the reaction mechanism varies with pH according to the virtual transition state hypothesis.

Supplementary Discussion 7

7.6 O–O bond formation on Ni-Fe sites

Integrating insights from proton inventory, cation effects, and anion effects experiments, it is deduced that the RDS at the Ni-Fe sites is predominantly the O–O bond formation process, which entails two succession proton transfer reactions, as depicted in ***Supplementary Fig. 101***. The Fe–OOH fragment is formed through a concerted PCET process, exhibiting a pronounced cation effect. Concurrently, or within the same reaction timescale, the subsequent deprotonation of Fe–OOH is facilitated by the adjacent nickel Brønsted-base site, notably without the influence of solvation anion effects, leading to the formation of the M–OO species prior to the oxygen release step. Given that the nickel site transitions to a higher oxidation state during the $Ni^{2+/3+}$ oxidation process, it can serve both as a proton and electron acceptor. Consequently, in the secondary state of RDS, the intra-system proton transfer is effectively "coupled" with electron transfer. Proton inventory analysis reveals that the two stages (TS1, TS2) of the RDS can be conceptualized as a singular virtual transition state (TSv) in which the catalytic site loses a total of one electron and one proton. Combining findings from the proton inventory and anion effects experiments suggests that the equivalent pK_a value of the intramolecular Ni^{3+} relay site falls between 9 and 12, closely aligning with the pK_a value (11.5) for the Ni^{3+} species in the single Ni site of Aza-CMP-Ni (***Supplementary Fig. 84***). The intramolecular proton and electron shuttle directly accelerates the kinetics on the iron site, resulting in excellent intrinsic OER activities, particularly in neutral to mildly alkaline environments. Moreover, given that the intramolecular transfer process is essentially not controlled by the solvent, further increasing the concentration of the alkaline solution under strongly alkaline conditions fails to yield notable performance improvements and might instead introduce adverse effects. Consequently, selecting an appropriate pH range is crucial. Finally, we briefly address how multinuclear metal sites might affect the mechanistic conclusions regarding O–O bond formation on Ni-Fe sites. Based on the preceding discussion, we conclude that the probability of nanoscale NiFe clusters existing in the synthesized pristine Aza-CMP-NiFe is very low, with Ni-Fe molecular sites likely dominating the system. Quantitative electrochemical analysis, as detailed in ***Supplementary Notes 3***, shows that the electrochemically active species in Aza-CMP-NiFe closely align with theoretical models. This strongly supports the conclusion that only one predominant redox species exists on the electrode surface. Nevertheless, we still hope to point out that even if low-activity aggregates are present in the system, they would not significantly impact the analysis of the catalytic mechanism discussed above. The characterization results for high-valent species remain reliable regardless of the presence of multi-nuclear metal sites, as these results are independent of the form of the active species. For instance, even if the active Ni species exist as clusters, the high-valent Ni^{4+} species involved in catalysis would still be observed. Thus, the potential existence of multinuclear metal sites does not alter these conclusions. More importantly, the mechanistic studies in this work, including proton inventory, pH-dependent effects, solution anion/cation effects, kinetic isotope effects, APT studies, quantitative cyclic voltammetry analysis, and electrochemical activation energy measurements, are entirely based on electrochemical methods. As a result, the data and conclusions derived from these studies are not influenced by the presence of non-electrochemically active sites, such as clusters, even if they exist.

The reversible dissociation and recovery of Fe sites have been reported in NiFe material-based systems, wherein Fe ions can detach from Ni-Fe sites under catalytic conditions but tend to rebind to Ni sites during relaxation or at lower potentials.[93, 94] A similar reversible process may also be present in molecular site-based systems. The formation of Ni-Fe sites is driven by the selective adsorption of Fe ions onto Ni sites, indicating that a reversible desorption process could potentially occur. However, this dynamic equilibrium is unlikely to be detectable by the current structural characterization of the catalyst under operating conditions due to the difference in timescale.

Supplementary Discussion 7

Once Ni-Fe sites undergo decomposition during catalysis, they are no longer catalytically active. Importantly, the mechanistic studies conducted in this work are all based on electrochemical methods. Given the timescale of these measurements (e.g., a CV scan from 1.2 to 1.6 V at 10 mV s-1 takes 80 s), significant decomposition of Ni-Fe sites is unlikely to occur during the experiments. This is further supported by the long-term stability test, which shows that the catalytic current of Aza-CMP-NiFe remains stable over a 15-hour operation period. Moreover, any dissociation of Fe during OER is likely counteracted by the rapid re-adsorption of Fe ions from trace impurities in the electrolyte, leading to a dynamic equilibrium between site degradation and repair. On the macroscopic scale, this is reflected in the observation that pre- and post-catalytic structures show no significant differences. Crucially, the possible presence of such a dynamic equilibrium should not be interpreted as a sign of catalyst instability. Nor does it undermine the reliability of structural characterization or the mechanistic insights derived from our analysis. A more precise characterization of this dynamic process would likely necessitate time-resolved *operando* synchrotron techniques. However, such measurements remain highly challenging for heterogeneous atomic-dispersed systems with ultra-low metal loadings (< 3 wt%), underscoring a critical direction for future research.

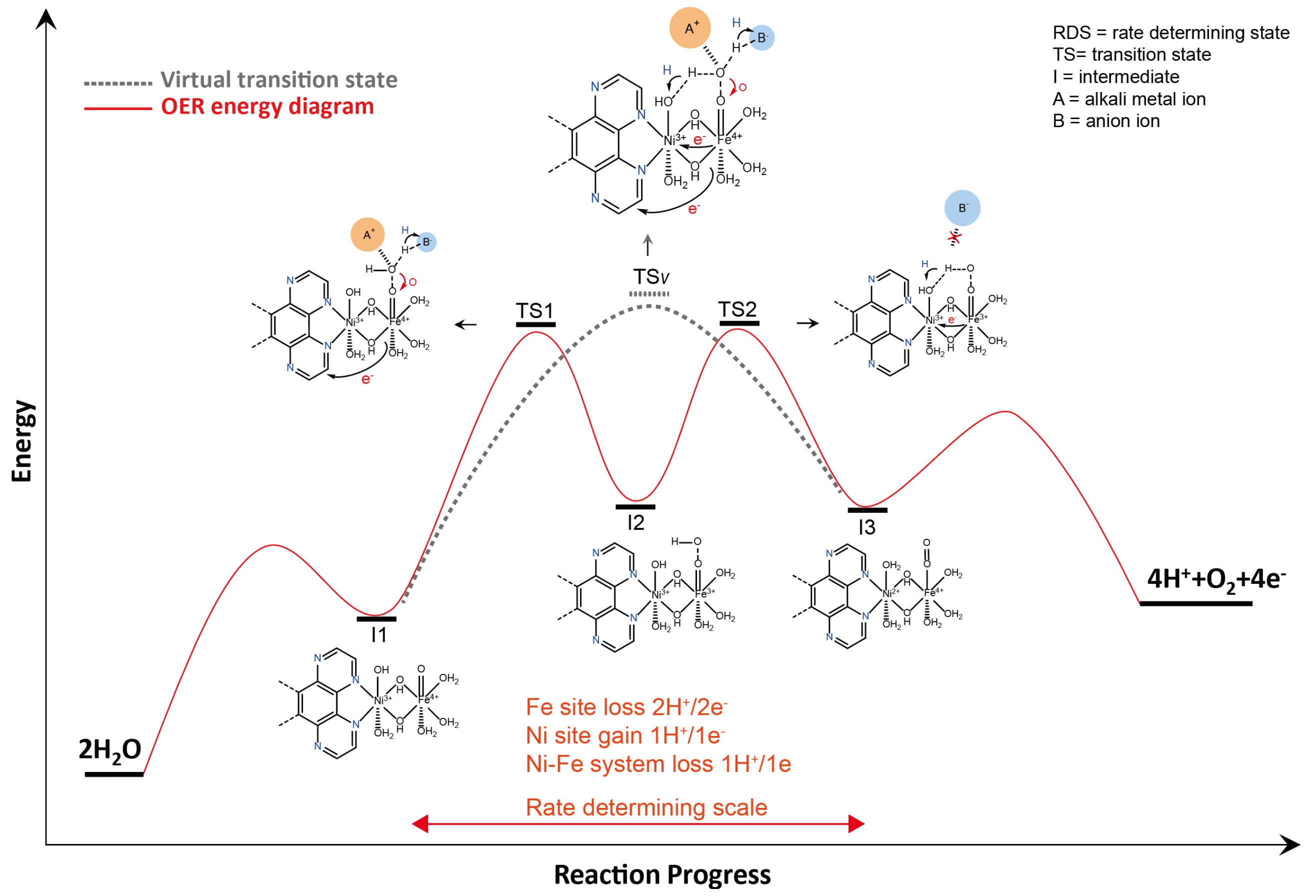

Supplementary Fig. 101 Schematic reaction diagram and related reaction intermediates and transition states.

Supplementary Notes 1

Supplementary Notes 1: EXAFS simulation

1.1 Aza-CMP-Ni simulation

The ideal Ni K-edge EXAFS patterns of the calculated DFT model were simulated using the following protocol. The FEFF file for the Aza-CMP-Ni DFT structure depicted in ***Supplementary Fig. 102*** was generated using Demeter 0.9.26, with some hydrogen atoms omitted to simplify subsequent simulations (***Supplementary Fig. 104a***). Possible scattering paths were generated using the FEFF calculation in the Artemis program.[95] A total of 120 paths were identified within 5.000 Å. The single scattering paths contributed by the first coordination sphere are listed in ***Supplementary Table 6***.

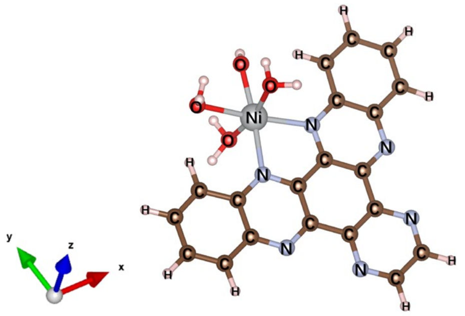

Supplementary Fig. 102 Calculated DFT model of Aza-CMP-Ni.

Supplementary Table 6 Example of single scattering paths obtained by the FEFF calculation.

#	degen	Reff	scattering path	rank	type
1	1	2.003	O1.1	100	single scattering
2	2	2.138	O2.1	100	single scattering
3	2	2.143	N1.1	93.84	single scattering
4	1	2.193	O4.1	47.13	single scattering
5	2	2.928	C7.1	41.53	single scattering
6	2	2.952	H2.1	5.88	single scattering
7	2	3.185	C11.1	34.06	single scattering

The simulated EXAFS spectra were obtained by summing up all the dominant waves from the FEFF calculation. As illustrated in ***Supplementary Fig. 103a***, the FEFF calculation is displayed as χ(k) with S_0^2 set to 1 and each of E_0, ΔR, and σ^2 set to 0. The comparison between simulated and experimental plots is shown in ***Supplementary Fig. 103b***, with results suggesting a good correspondence between the simulated and experimental results in the 0-12 $Å^{-1}$ range. The slight amplitude and phase mismatch is attributed to the non-negligible values of E_0, ΔR, and σ^2. Furthermore, ***Supplementary Fig. 103c*** presents the χ(R) plots for the FEFF calculation, illustrating the Fourier transform of χ(k) parameterized with the same values (note that the path radius larger than 4.5 Å was omitted when plotting the χ(R) pattern). The peaks contributed by the 1st and outer coordination spheres aligned well with the experimental results (***Supplementary Fig. 103d***). The wavelet transform (WT) was performed using the HAMA-Fortran program. The signal was processed with the Morlet wavelet function employing κ = 10 and σ = 1, and setting the processing range from 0 to 6 Å. The corresponding simulated WT of the EXAFS data from the DFT model detected only one intensity maximum at approximately 5 $Å^{-1}$, which was assigned to the Ni–O and Ni–N contributions, further demonstrating the configuration similarity between the proposed structure and the as-fabricated Aza-CMP-Ni (***Supplementary Figs. 103e*** and ***103f***). The most significant advantage of simulation analysis

is the clear distinction of contributions from each atom in the proposed structure. As illustrated in ***Supplementary Fig. 104b***, the peak in the 1st coordination sphere (1-2 Å) is contributed by the 2 surrounding N atoms in the CMP backbone and 4 coordinated O atoms from the solvent. Due to the close bonding distance, it would be difficult to distinguish the Ni–O and Ni–N paths in the experiments. The carbon atoms in the Aza-CMP framework led to the two main peaks in the outer coordination sphere (2-4 Å), which are also clearly observed in the experimental spectrum (***Supplementary Figs. 104c*** and ***104d***). The aforementioned simulation further corroborates the reliability of the proposed molecular nature of the as-fabricated Aza-CMP-Ni, facilitating subsequent OER kinetic studies.

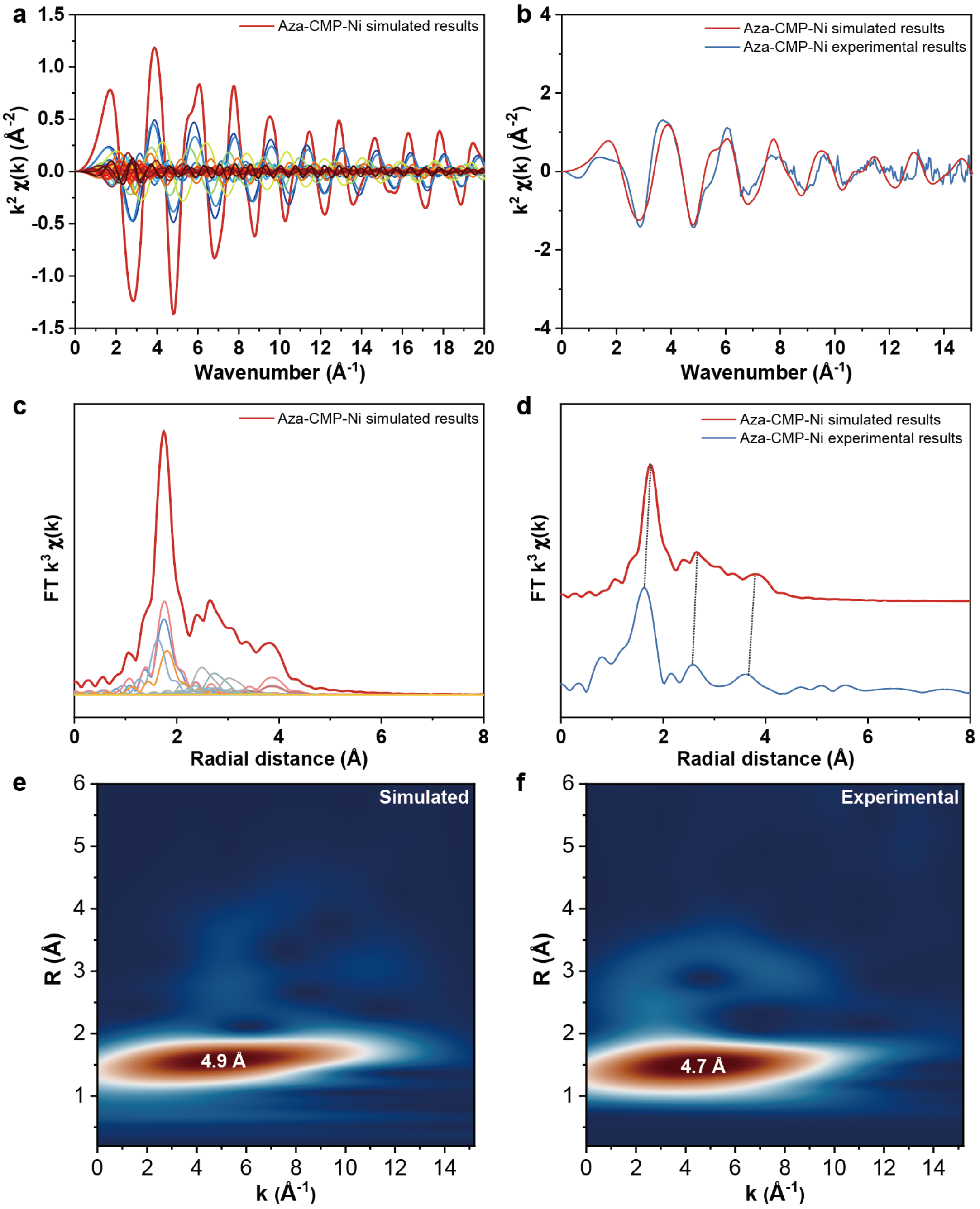

Supplementary Fig. 103 EXAFS simulation of Aza-CMP-Ni. (a) Simulated k^2-weighted Ni K-edge EXAFS curves of Aza-CMP-Ni in k-space. (b) The comparison between the simulated and experimental EXAFS curves in k-space. (c) Simulated k^3-weighted Ni K-edge EXAFS curves of Aza-CMP-Ni in R space. (d) The comparison between the simulated and experimental EXAFS curves in R space. The wavelet transforms of (e) simulated and (f) experimental Ni K-edge EXAFS spectra.

Supplementary Notes 1

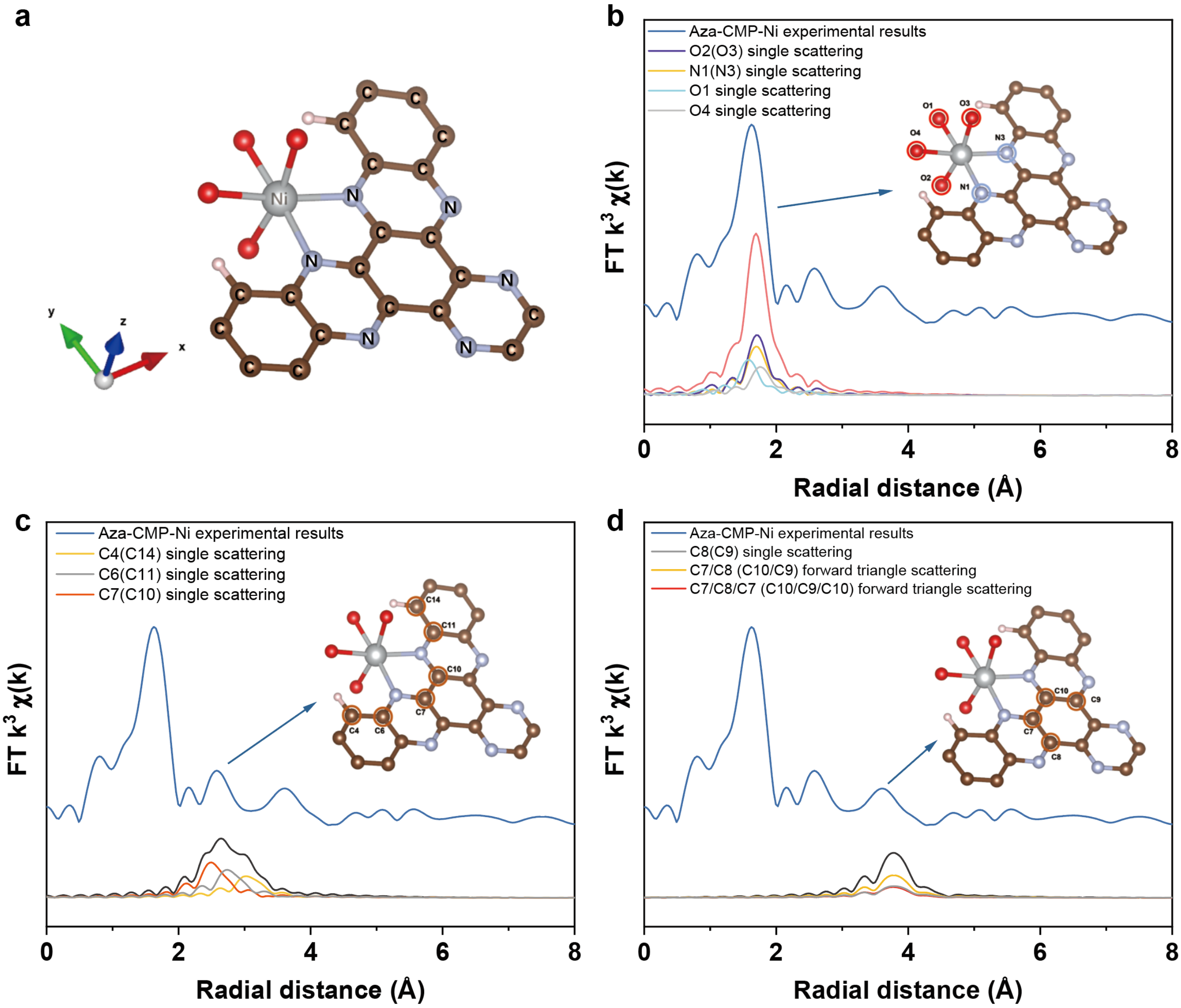

Supplementary Fig. 104 EXAFS signal contribution analysis. (a) Aza-CMP-Ni Model for EXAFS simulation, part of hydrogen atoms was omitted to simplify further simulations. (b) EXAFS contribution analysis of the 1st coordination sphere (1-2 Å). (c) EXAFS contribution analysis of the 2nd coordination sphere (2-3 Å). (c) EXAFS contribution analysis of the outer coordination sphere (>3 Å).

Supplementary Notes 1

1.2 Aza-CMP-NiFe simulation

The Ni K-edge and Fe K-edge EXAFS curves of the proposed Aza-CMP-NiFe structure in ***Supplementary Fig. 105*** were also simulated using similar protocols. As shown in ***Supplementary Figs. 106a*** and ***106c***, the simulated EXAFS spectra were obtained by summing up all the dominant waves from the FEFF calculation in both k and R space, respectively. The comparison between simulated and experimental plots is displayed in ***Supplementary Figs. 106b*** and ***106d***. In the k-space, the simulated curve corresponds well with the experimental results in the range of 0-14 $Å^{-1}$; the amplitude and phase mismatch occurring when the wavenumber is >10 $Å^{-1}$ might be caused by poor signal quality during XAS measurements of low metal content samples (***Supplementary Fig. 106b***). In the R space, both simulated and experimental curves exhibit one intensity maximum at the 2nd coordination sphere (2-3 Å) (***Supplementary Fig. 106d***). The expected intensity ratio of the second-shell peak to the primary peak in the first shell (1-2 Å) is approximately 0.6:1. However, the experimental ratio is 0.4:1, indicating that not all Ni sites in the framework form Ni-Fe bonds; rather, only electrochemically active Ni sites interact with Fe to form Ni-Fe sites. This observation aligns with the results of our elemental analysis, which show that Fe content is lower than Ni content in Aza-CMP-NiFe. The corresponding simulated k^2-weighted EXAFS WT shows two intensity maximums at approximately 4.6 $Å^{-1}$ and 6.3 $Å^{-1}$, which are assigned to the Ni–O and Ni–Fe contributions (***Supplementary Fig. 106f***); the position of the second shell peak is clearly lower than the metallic Ni–Ni path (nickel foil, k = 7.5 $Å^{-1}$) and oxide Ni–Ni path ($Ni(OH)_2$ and $LiNiO_2$, k = 7.3 $Å^{-1}$) (***Supplementary Fig. 36***). The WT results confirm a similar configuration is shared between the proposed structure and the as-fabricated Aza-CMP-NiFe (***Supplementary Fig. 106e***). The EXAFS contribution analysis of the 1st shell peak displayed in ***Supplementary Fig. 107a*** reveals that the intensity maximum is attributed to the surrounding N atoms in the Aza-CMP backbone and coordinated O atoms. Additionally, as shown in ***Supplementary Fig. 107b***, the 2nd shell peak (2-3 Å) is primarily contributed by neighboring metal atoms, which is the iron atom in this case. It is worth mentioning that the shoulder peak observed in the 2nd shell of the experimental curve is attributed to the backbone carbon atoms in the CMP framework, further explaining that due to the existence of multiple paths, including Ni–O, Ni–N, Ni–C, and Ni–Fe paths, there are too many variables, and it is not feasible to directly fit the data in the dual-metal system.

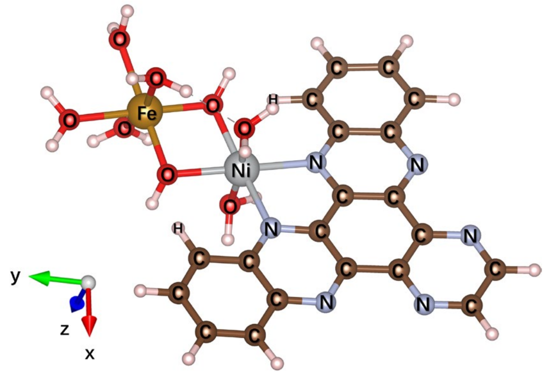

Supplementary Fig. 105 Calculated DFT model of Aza-CMP-NiFe.

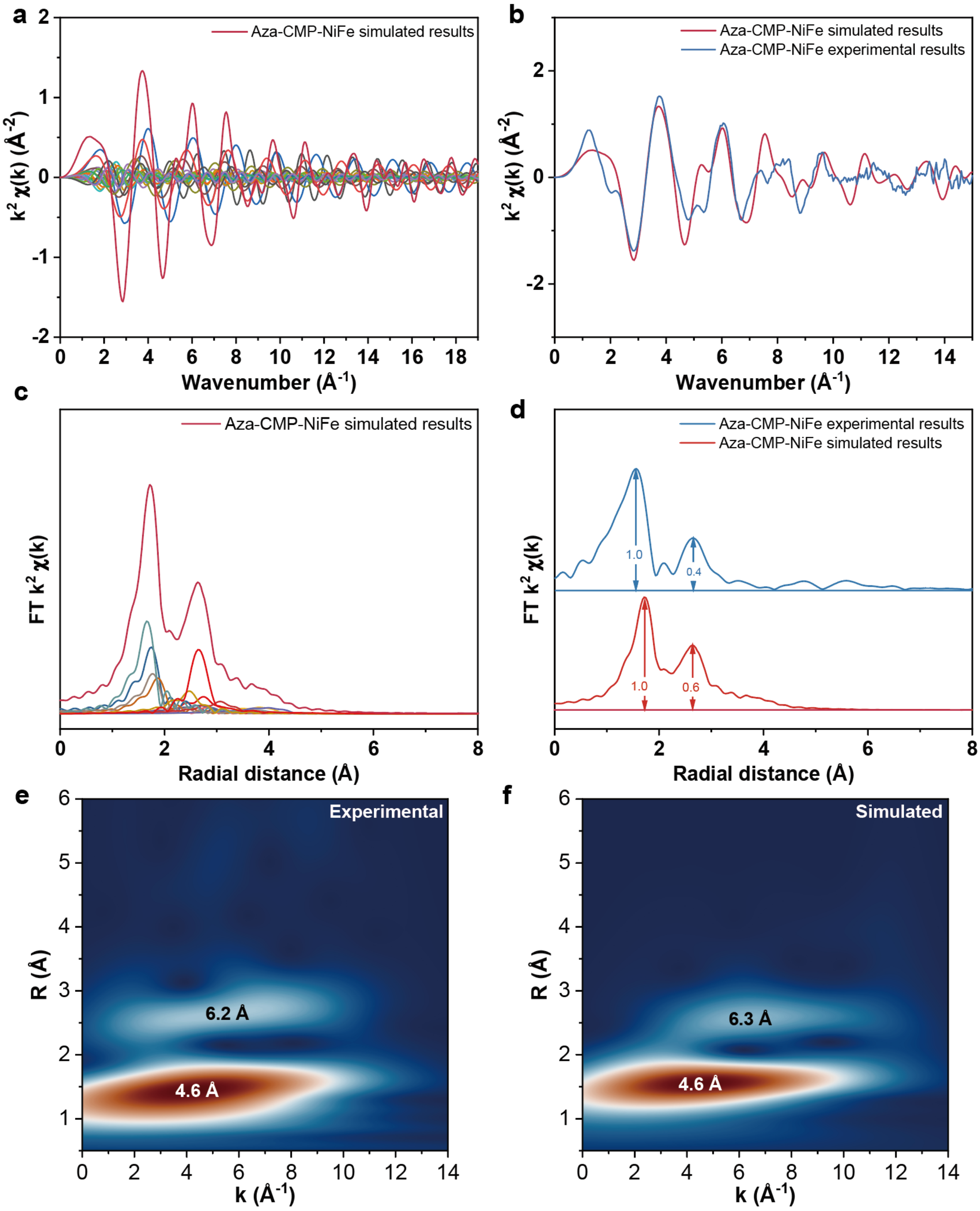

Supplementary Fig. 106 EXAFS simulation of Aza-CMP-NiFe at Ni K edge. (a) Simulated k^2-weighted Ni K-edge EXAFS curves of Aza-CMP-NiFe in k-space. (b) The comparison between the simulated and experimental EXAFS curves in k-space. (c) Simulated k^2-weighted Ni K-edge EXAFS curves of Aza-CMP-NiFe in R space. (d) The comparison between the simulated and experimental EXAFS curves in R space. The wavelet transforms of (e) experimental and (f) simulated Ni K-edge EXAFS spectra.

Supplementary Notes 1

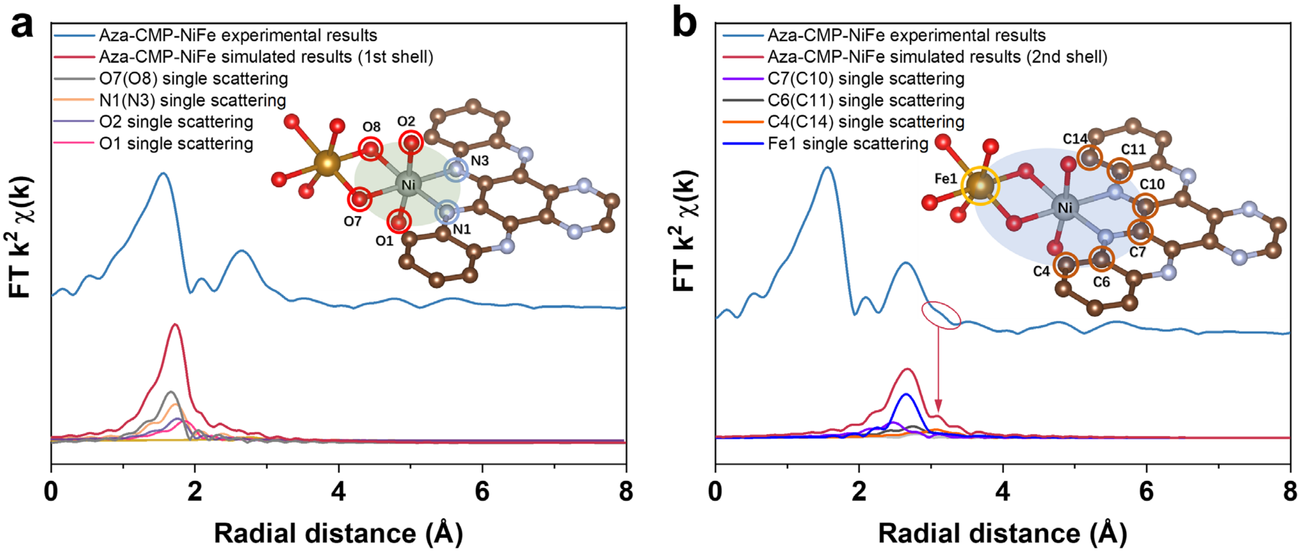

Supplementary Fig. 107 EXAFS signal contribution analysis. (a) EXAFS contribution analysis of the 1st coordination sphere (1-2 Å). (b) EXAFS contribution analysis of the 2nd coordination sphere (2-3 Å), the red circle indicates the intensity contributions from backbone carbon atoms.

For the Fe K-edge EXAFS, the simulated curves in the k-space align with the experimental results in the range of 0-12 $Å^{-1}$ (***Supplementary Fig. 108b***). Both simulated and experimental curves exhibit two intensity maxima in the R range of 1-3 Å (***Supplementary Fig. 108d***); the 1st shell peak corresponds to Fe–O/N scattering paths, while the 2nd shell peak is primarily attributed to the Fe–Ni scattering path. The similar 1st/2nd peak intensity ratio suggests that the coordination number of Ni is close to 1 for the as-fabricated Aza-CMP-NiFe. The corresponding EXAFS WT displays two intensity maxima at approximately 4.9 $Å^{-1}$ and 6.2 $Å^{-1}$, assigned to the Fe–O and Ni–Fe contributions, respectively (***Supplementary Fig. 108f***). The experimental position of the second shell peak (5.6 $Å^{-1}$) is lower than the metallic Fe–Fe path (iron foil, k = 7.95 $Å^{-1}$), oxide Fe–M path (NiFe hydroxide, k = 7.2 $Å^{-1}$), and oxide Fe–Fe path (Fe_2O_3, k = 7.1 $Å^{-1}$) (***Supplementary Fig. 37***), further confirming the unique Fe–Ni dual-metal atom feature in the experimental structure (***Supplementary Fig. 108e***). As demonstrated in the EXAFS contribution analysis of the 1st shell peak, the intensity maximum is attributed to the four coordinated terminal O atoms and two bridging O atoms (***Supplementary Fig. 109a***). The 2nd shell peak (2-3 Å) is contributed by the neighboring Fe center and two axial O atoms coordinated with the Ni center (***Supplementary Fig. 109b***). In summary, all simulation results support the molecular Ni-Fe site nature of the as-fabricated Aza-CMP-NiFe, enabling further OER kinetic studies.

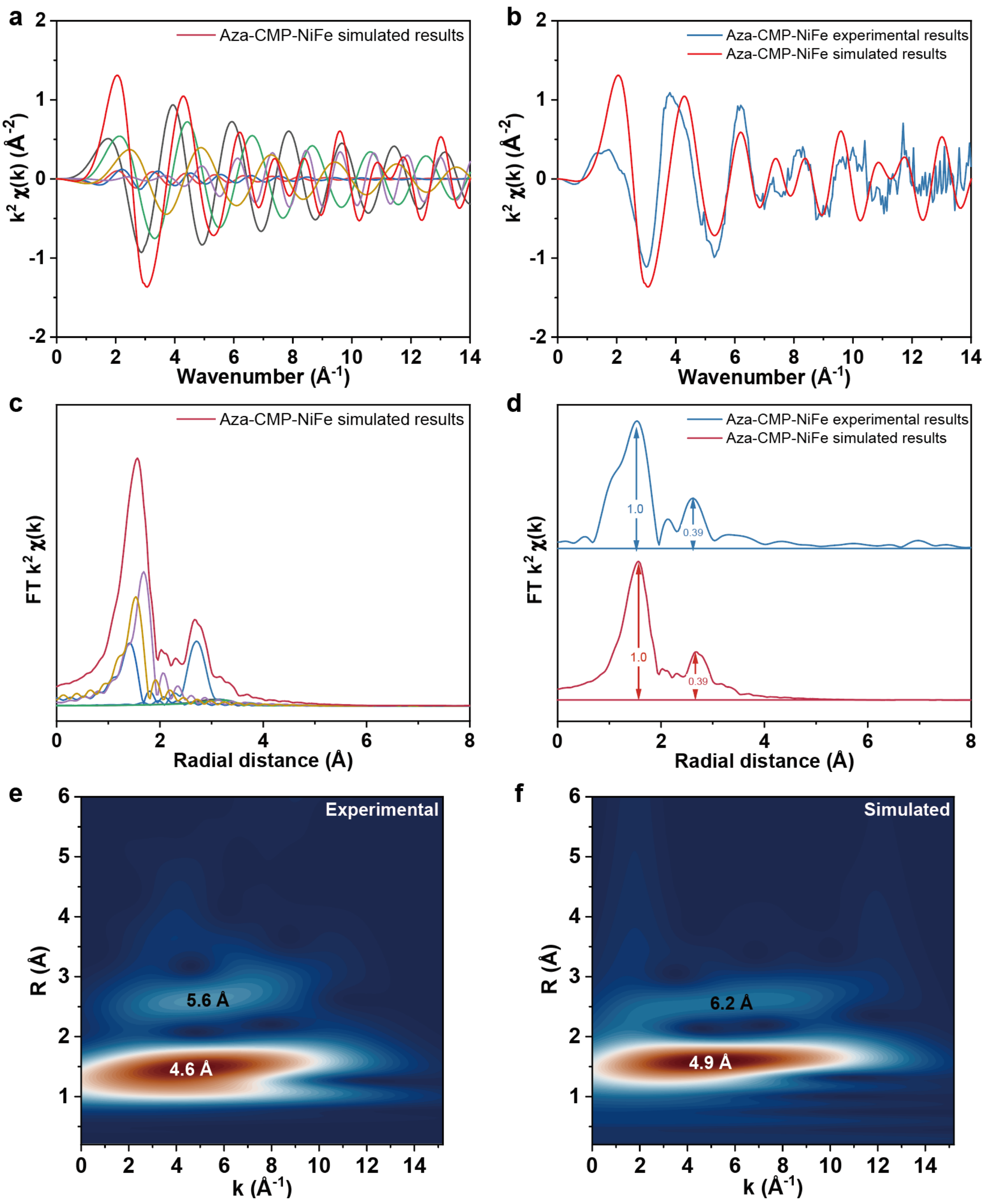

Supplementary Fig. 108 EXAFS simulation of Aza-CMP-NiFe at the Fe K edge. (a) Simulated k^2-weighted Fe K-edge EXAFS curves of Aza-CMP-NiFe in k-space. (b) The comparison between the simulated and experimental EXAFS curves in k-space. (c) Simulated k^2-weighted Fe K-edge EXAFS curves of Aza-CMP-NiFe in R space. (d) The comparison between the simulated and experimental EXAFS curves in R space. The wavelet transforms of (e) experimental and (f) simulated Fe K-edge EXAFS spectra.

Supplementary Notes 1

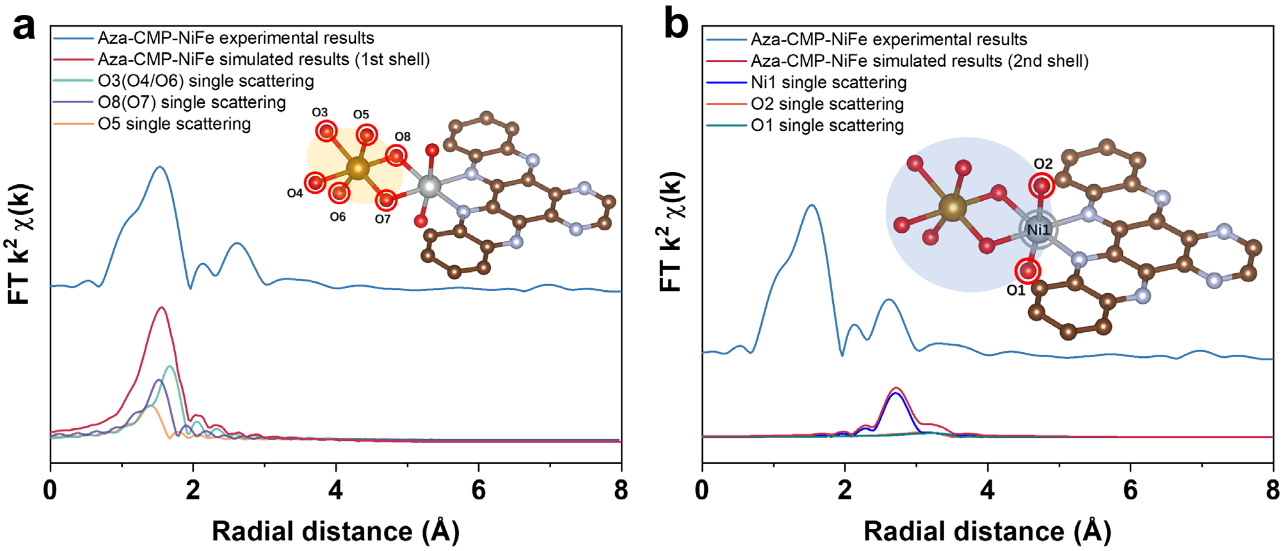

Supplementary Fig. 109 EXAFS signal contribution analysis. (a) EXAFS contribution analysis of the 1st coordination sphere (1-2 Å). (b) EXAFS contribution analysis of the 2nd coordination sphere (2-3 Å).

Supplementary Notes 2

Supplementary Notes 2: Chemical probing of Ni^{4+}=O by Et_3N

The types of active intermediates produced during the OER significantly impact the water oxidation mechanism and catalytic performance. Therefore, investigating these active intermediates is crucial for a comprehensive understanding of potential reaction pathways. Recently, advanced *operando* chemical probes have been developed and utilized to identify the key intermediates in the reaction cycles. Probes used in this context can be categorized into two types. The first type can selectively bind to reaction intermediates, thereby inhibiting the conventional water oxidation pathway and resulting in decreased reaction kinetics. For instance, tetramethylammonium (TMA^+) can interact with deprotonated active oxygen intermediates (O_2^{2-}) and occupy active sites.[96] Similarly, 4-(diphenylphosphino) benzoic acid (TPP-COOH) can effectively engage with oxygen intermediates Fe^{4+}=O, altering the OER kinetics by reducing the OER current.[97] The second type of probes can be selectively oxidized by specific intermediates, which exhibit an enhanced catalytic current due to the facile probe oxidation reaction compared to water oxidation. For example, alcohol molecules can probe reactive OH*(Ni^{3+}) generated in OER on oxides and (oxy)hydroxides,[98] causing the onset potentials of catalytic currents to shift negatively.[99] These chemical probes provide powerful tools to directly detect oxygen intermediates at operating conditions of OER. For the second type of chemical probe, target intermediate identification depends on comparing oxidation potentials. The effectiveness of these probes is determined by whether the intermediate's oxidation potential is sufficient to oxidize the probe. Typically, the ground state for Ni-Fe-based catalysts is $Ni^{2+}Fe^{3+}$.[2, 100, 101] To drive oxygen evolution, higher valent states such as Ni^{4+} or Fe^{4+} are required to facilitate the corresponding proton-electron transfer processes. As shown in ***Supplementary Table 7***, detecting the presence of Ni^{4+} remains a significant challenge to date. Ni^{4+} species is considered a critical intermediate for Ni-based water oxidation catalysis; the formation of Ni^{4+} is pivotal to understanding the OER catalytic mechanism. In our system, Ni^{4+} is expected to have a higher oxidation potential than Fe^{4+} (as its formation occurs at a higher oxidation potential). Therefore, selecting a water-soluble organic molecule with an oxidation potential between Ni^{4+} and Fe^{4+} is crucial for distinguishing them. In this section, we demonstrate that triethylamine (Et_3N) can be selectively oxidized by high valent Ni^{4+} species, providing a useful probe to identify the presence of high valent Ni during the OER catalysis by Ni-based catalysts.

Supplementary Table 7 Survey of chemical probes for intermediate identification in NiFe systems.

**Intermediates**	**Chemical probes**	**Reference**
Ni^{3+}(OH)	CH_3OH	Joule **2019**, 3 (6), 1498-1509
Fe^{4+}=O	TPP-COOH	J. Am. Chem. Soc. **2021**, 143 (3), 1493-1502.
Ni^{4+}=O	**Et_3N**	**This work**
M–OO^{2-}	TMA^+	Nat. Energy **2019**, 4 (4), 329-338.

As shown in ***Supplementary Fig. 110a***, the titration of triethylamine progressively enhanced the catalytic current of Aza-CMP-Ni in a concentration-dependent manner. Concurrently, a discernible redox wave emerged before 1.7 V vs. RHE, attributable to the electrooxidation of Et_3N. The corresponding concentration-current relationship is presented in ***Supplementary Fig. 110b***, revealing a gradual increase in the slope to 0.52 with the rising applied potential. Conversely, the titration of Aza-CMP-NiFe resulted in substantially smaller changes in the OER current (***Supplementary Fig. 110c***). The slopes are calculated close to 0 from 1.5 V to 1.6 V vs. RHE, where significant OER

catalytic activity could be observed (***Supplementary Fig. 110d***). These contrasting characteristics suggest that Ni and Ni-Fe sites generate disparate intermediates in aqueous conditions, where Ni site intermediates of Aza-CMP-Ni exhibit the ability to catalyze the oxidation of Et_3N molecules. High-valent metal oxo species were identified as capable of oxidizing Et_3N. For instance, $Ru^{5+}{=}O(EDTA)^{-}$ can efficiently react with the *N*-alkyl group in tertiary and secondary amines to produce secondary and primary amines, respectively, with the *N*-alkyl group undergoing oxidation to the corresponding aldehyde.[102, 103] Given the proximity of the onset potential of Et_3N oxidation in the LSV curve of Aza-CMP-Ni to $Ni^{3+/4+}$ redox areas, we propose that $Ni^{4+}{=}O$ species generated in Aza-CMP-Ni are active toward oxidative *N*-dealkylation, with kinetics significantly faster than the sluggish OER. This observation further demonstrates that high-valence Ni species with robust oxidative ability may not exist in the Ni-Fe system.

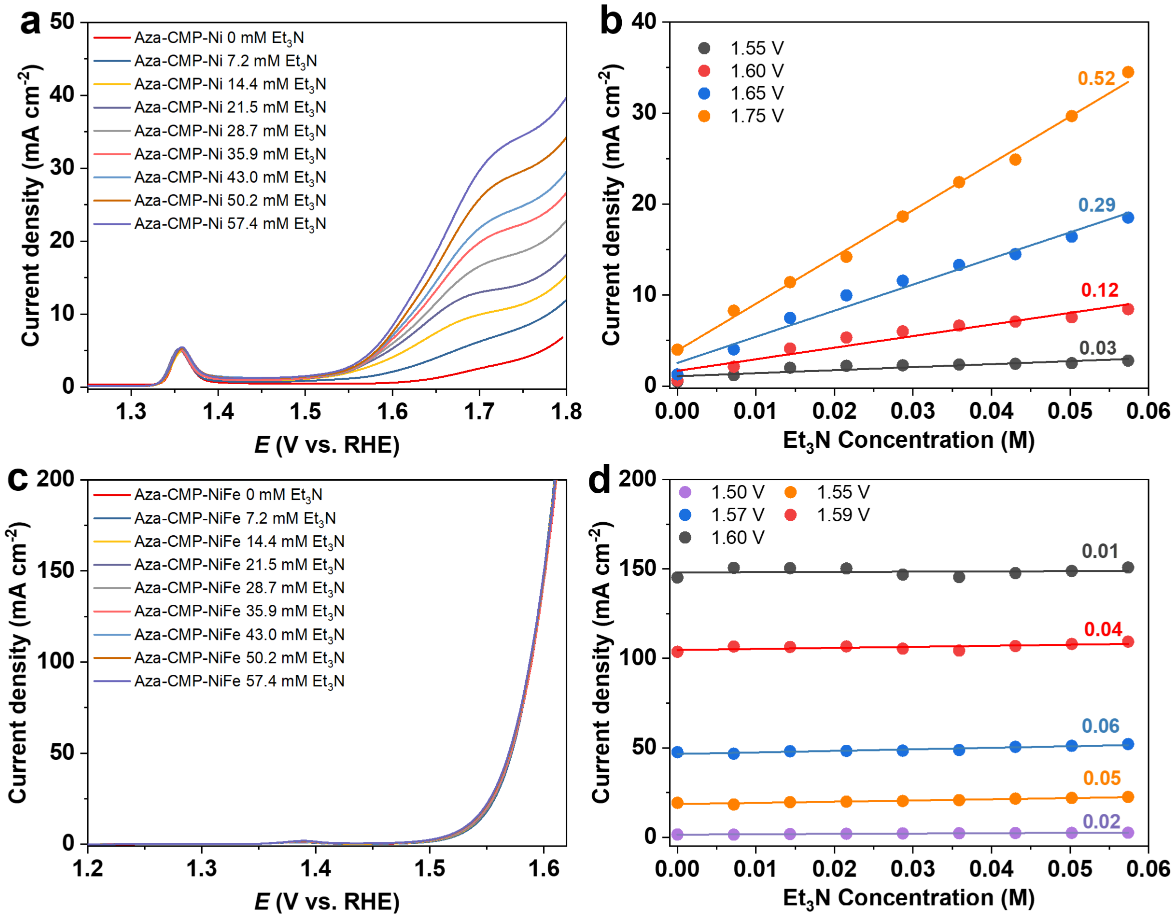

Supplementary Fig. 110 Triethylamine titration analysis. (a) LSV curves of Aza-CMP-Ni/CP in 1.0 M NaOH with different concentrations of Et_3N and (b) the corresponding Et_3N concentration-current relationship (scan rate: 50 mV s^{-1}). (c) LSV curves of Aza-CMP-NiFe/CP in 1.0 M NaOH with different concentrations of Et_3N and (d) the corresponding Et_3N concentration-current relationship (scan rate: 50 mV s^{-1}).

We also investigated the electrochemical properties of the blank CP substrate with various concentrations of Et_3N. As illustrated in ***Supplementary Fig. 111a***, the catalytic current also exhibits a degree of concentration dependence, suggesting that at a higher potential, Et_3N can also be oxidized on carbon materials. However, when juxtaposed with the Aza-CMP-Ni/CP electrode, the CP electrode displays a much higher current onset potential and lower current under the same concentration of Et_3N, indicating differing active intermediates (***Supplementary Fig. 111b***). DPV curves provide a more

intuitive representation of the redox events during catalysis (***Supplementary Fig. 111c***). As mentioned in the main text, two redox peaks corresponding to the reduction-oxidation of the $Ni^{2+/3+}$ couple at 1.31 V vs. RHE and the $Ni^{3+/4+}$ redox couple at 1.64 V vs. RHE were observed for Aza-CMP-Ni/CP in pure NaOH electrolytes. With the addition of Et_3N, the $Ni^{2+/3+}$ couple exhibited almost no changes, indicating that Ni^{3+} cannot react with Et_3N. However, the intensity of the $Ni^{3+/4+}$ peak significantly increased compared to $Ni^{2+/3+}$, suggesting the oxidation of Ni^{4+} is facilitated in the presence of the more readily oxidized Et_3N. Furthermore, Et_3N oxidation on the CP electrode also revealed an oxidation peak at a higher potential of 1.71 V, reinforcing that the presence of high-valence Ni expedites Et_3N oxidation. The catalytic currents of Aza-CMP-Ni/CP, Aza-CMP-NiFe/CP, and CP electrodes were compared at a potential of 1.6 V vs. RHE (***Supplementary Fig. 111d***). The CP electrode is virtually inactive at low potential and demonstrates no catalytic current. However, upon the formation of active Ni^{4+}=O, the Aza-CMP-Ni/CP demonstrated a pronounced concentration dependency toward Et_3N oxidation. Importantly, the concentration of Et_3N probes does not impact the OER kinetics of the Aza-CMP-NiFe catalyst. This observation implies that the crucial intermediates on Ni-Fe sites of Aza-CMP-NiFe substantially deviate from those on Ni sites of Aza-CMP-Ni, following a unique OER pathway. This discrepancy may be attributed to the absence of Ni^{4+} generation during water oxidation at Ni-Fe sites, further corroborating that the oxygen intermediates on Ni^{4+} sites are the principal targets for probing.

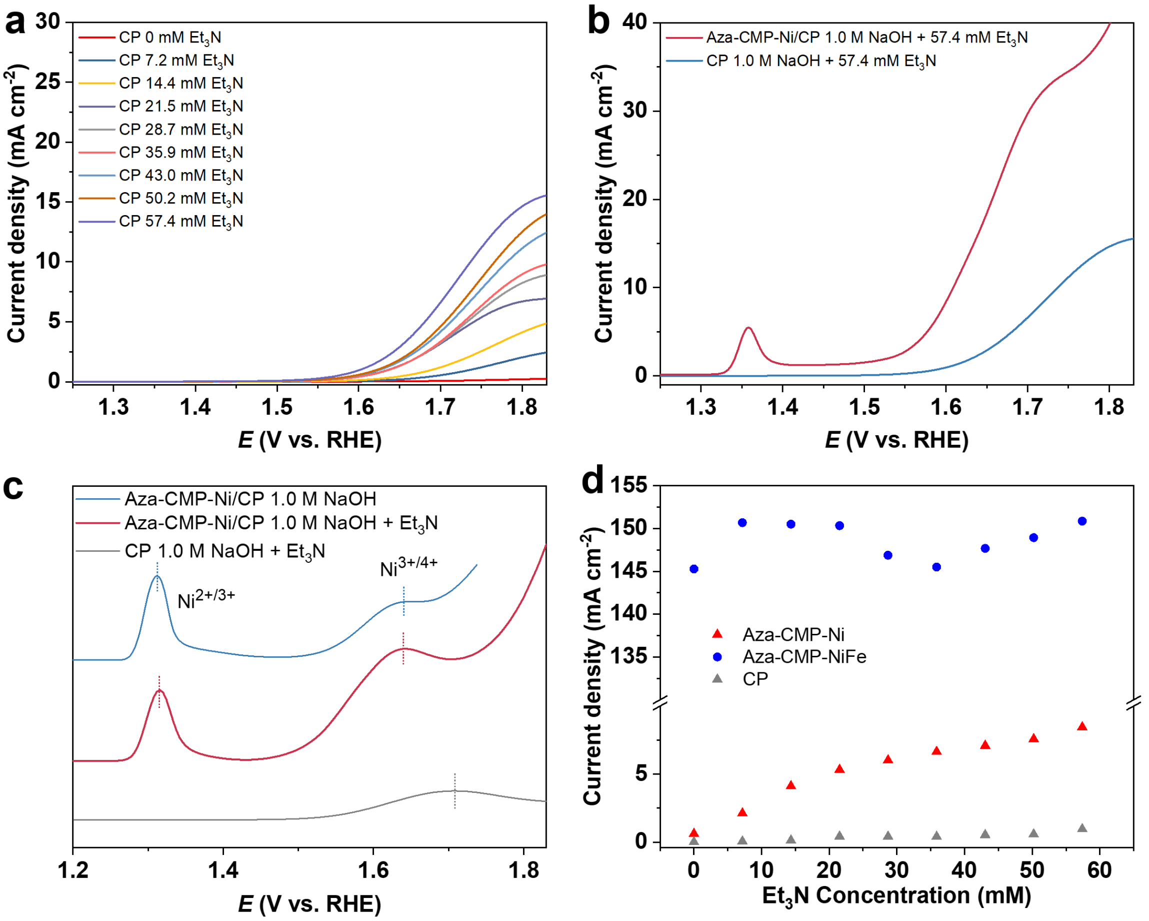

Supplementary Fig. 111 Triethylamine titration comparison. (a) LSV curves of blank CP in 1.0 M NaOH with different concentrations of Et_3N (scan rate: 50 mV s^{-1}). (b) The comparison of LSV curves of Aza-CMP-Ni/CP and CP electrodes under 1.0 M NaOH with the addition of 57.4 mM Et_3N. (c) The comparison of DPV curves of Aza-CMP-Ni/CP and CP electrodes. (d) The comparison of current densities was obtained from Aza-CMP-Ni/CP, Aza-CMP-NiFe/CP, and CP electrodes at 1.6 V vs. RHE.

Supplementary Notes 2

The product generated during Et_3N oxidation was analyzed using ^1H NMR spectroscopy. As shown in ***Supplementary Fig. 112***, in the fresh electrolyte, the triplet peaks at 0.8 ppm and the double-double peaks at 2.3 ppm are attributed to the CH_3 and CH_2 components of ethyl groups, respectively. Upon continued electrolysis by CV operation from 1.5 to 1.7 V vs. RHE, new triplet peaks and double-double peaks emerging at a relatively higher chemical shift are ascribed to the *N*-ethyl group in Et_2NH; the intensity of these new peaks also increases with extended electrolysis time. Meanwhile, a new peak at 8.25 ppm is observed in the high chemical shift region, which could be attributed to the corresponding aldehyde (***Supplementary Fig. 113***).

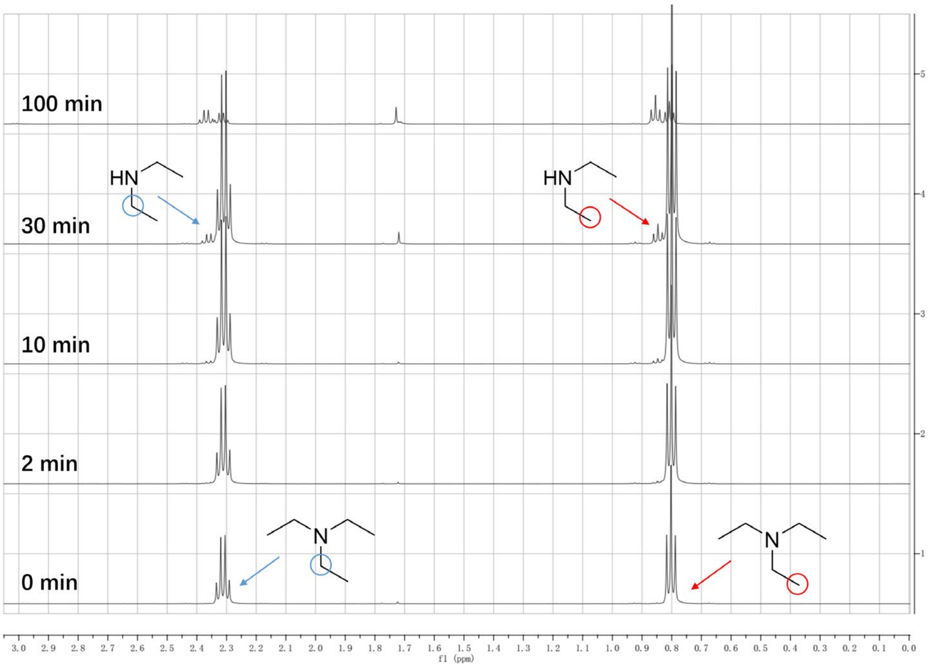

Supplementary Fig. 112 ^1H NMR spectra of the electrolytes at different operation times using the Aza-CMP-Ni/CP electrode. At low chemical shift region.

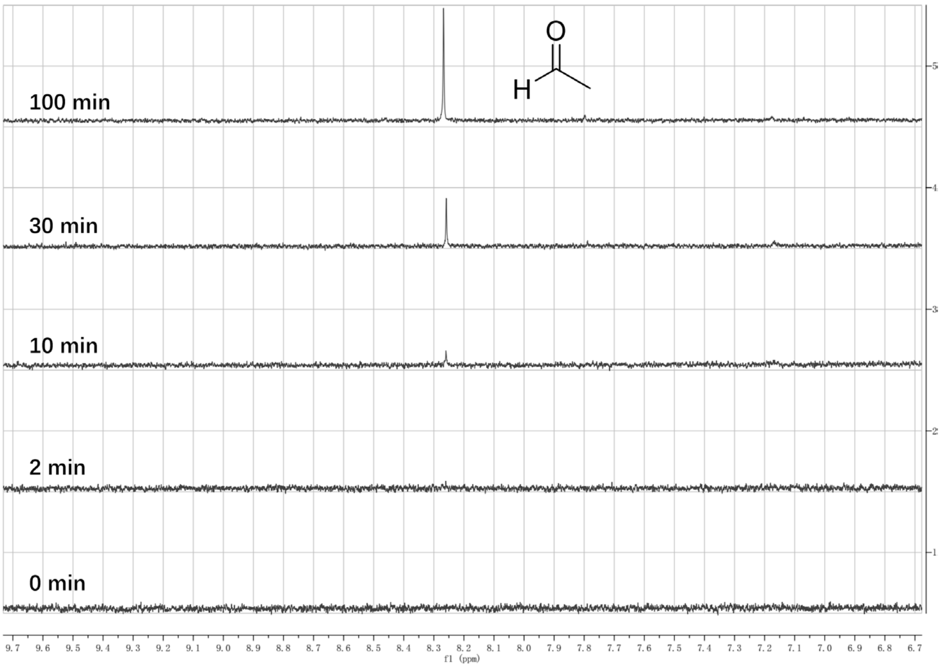

Supplementary Fig. 113 ^1H NMR spectra of the electrolytes at different operation times using the Aza-CMP-Ni/CP electrode. At high chemical shift region.

Supplementary Notes 2

It is important to clarify that Et_3N can only indicate the presence of a high-valent species capable of oxidizing Et_3N (e.g., Ni^{4+}), but it cannot specifically identify the structural form of the Ni^{4+} species, such as whether it exists as a Ni^{4+}=O moiety. The presence of a high-valent species does not necessarily imply the formation of a terminal M=O structure. Therefore, this work does not claim that Et_3N specifically identifies the existence of Ni^{4+}=O. Building on previous studies indicating that the oxidation of Et_3N is primarily facilitated by high-valent metal-oxo species (e.g., Ru^{5+}=O),[102, 103] the surface species responsible for catalyzing Et_3N oxidation here is speculated to be metal oxo species. Meanwhile, together with other *operando* spectroscopic characterizations and the Pourbaix diagram (Fig. 3c) in the main text, we proposed that the Ni^{4+} species is most likely present in the form of Ni^{4+}=O. Therefore, akin to Ru^{5+}, the mechanism of Ni^{4+}-catalyzed Et_3N oxidation is depicted in ***Supplementary Fig. 114***. The insertion of the Ni=O group into the α–C–H bond of the amine occurs when Ni^{4+} is generated. The kinetics of this process are faster than those of O–O bond formation during water oxidation at Ni sites, resulting in the immediate onset of the overall catalytic current upon the formation of Ni^{4+} and exhibiting a strong concentration dependence on the Et_3N substrate. In summary, the Et_3N molecule can effectively interact with Ni^{4+} oxygen intermediates, subsequently enhancing the current through a facile Et_3N oxidation reaction. This interaction serves as a reliable chemical probe for identifying the presence of high-valence Ni during water oxidation.

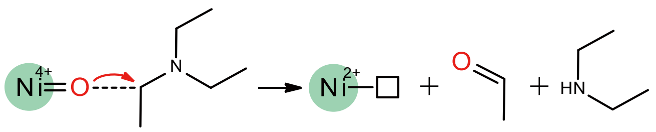

Supplementary Fig. 114 The proposed reaction mechanism of Ni^{4+} catalyzes Et_3N oxidation.

The probe molecules could undergo single-electron electrochemical oxidation, which might interfere with their intended detection. To clarify this concern, the electrochemical behavior of Et_3N, and TPP-COOH was assessed using a blank carbon paper electrode in 1 M NaOH electrolyte. As shown in ***Supplementary Fig. 115***, both probes exhibited oxidation potentials around 1.8 V vs. RHE, which are notably higher than the potentials for Ni^{4+} formation in Aza-CMP-Ni and Fe^{4+} formation in Aza-CMP-NiFe. This suggests that direct single-electron electrochemical oxidation of these probes is unlikely to affect the detection of key high-valent metal species in our system. Before reaching the OER onset potential, both TPP-COOH and Et_3N remain stable. Under OER conditions, their oxidation is more plausibly driven by electrocatalytic interactions with surface high-valent species rather than direct single-electron electrochemical oxidation. In summary, the Et_3N molecule can effectively interact with Ni^{4+} oxygen intermediates, subsequently enhancing the current through a facile Et_3N oxidation reaction. This interaction serves as a reliable chemical probe for identifying the presence of high-valence Ni during water oxidation.

Supplementary Notes 2

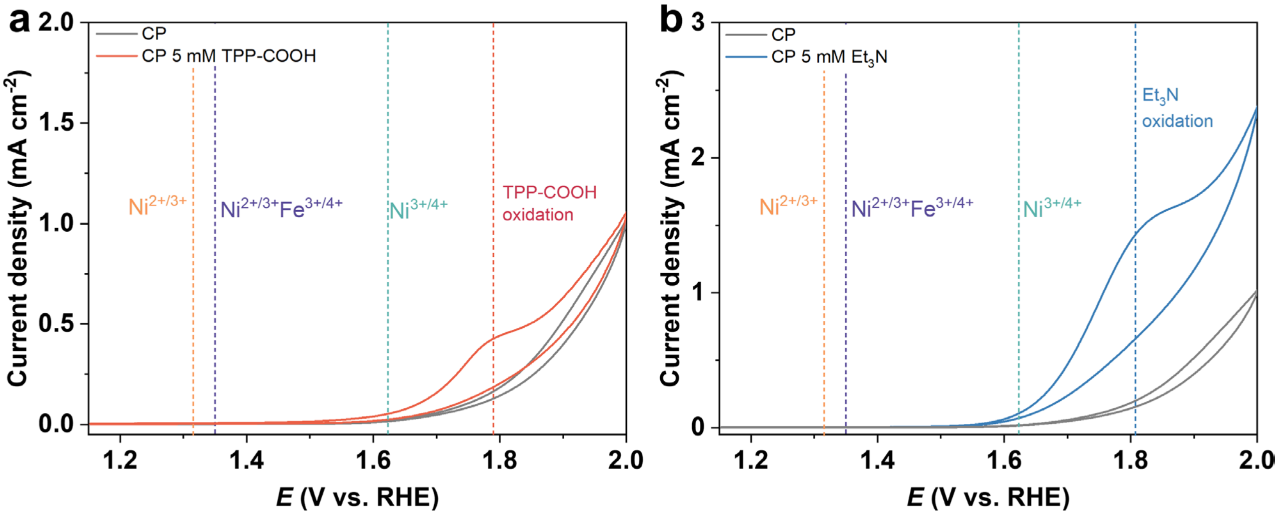

Supplementary Fig. 115 CV curves of blank carbon paper in 1.0 M NaOH with (a) TPP-COOH and (b) Et_3N (scan rate: 50 mV s^{-1}).

Supplementary Notes 3

Supplementary Notes 3: Quantitative CV analysis

3.1 $Ni^{2+/3+}$ redox

The electrochemical response of adsorbed monolayers for the electrode reaction $O + ne \leftrightarrow R$ can be significantly influenced by the adsorption of species O or R. For an ideal Nernstian reaction under Langmuir isotherm conditions, where $E_{p,O} = E_{p,R}$, the expected current-potential behavior in cyclic voltammetry experiments is described in ***Supplementary Eqn. 31***.[11] The anodic wave upon scan reversal appears as the mirror image of the cathodic wave, reflected across the potential axis. Additionally, the total width at half-height of either the cathodic or anodic wave is provided by ***Supplementary Eqn. 32***.

$$i_{23} = \frac{n_e^2 F^2 v A \Gamma_T}{RT} * \frac{\exp\left[\left(\frac{n_e F}{RT}\right)\left(E - E^{0'}\right)\right]}{\left\{1 + \exp\left[\left(\frac{n_e F}{RT}\right)\left(E - E^{0'}\right)\right]\right\}^2} \quad (31)$$

$$\Delta E_{p,1/2} = 3.53\frac{RT}{n_e F} = \frac{90.6}{n_e}\ mV\ (25\ ℃) \quad (32)$$

However, in our study of $Ni^{2+/3+}$ redox in Aza-CMP-Ni and Aza-CMP-NiFe, the $E_{p,O}$ did not match with $E_{p,R}$, and the potential difference suggests that the electrochemical process is more irreversible, with strong interactions between the adsorbed molecules. The non-ideal current-potential behavior can be established using ***Supplementary Eqns. 33-38***.[104, 105]

$$n_e\left(E - E^{0'}\right) = \left(\frac{RT}{F}\right)\{ln[f/(1-f)] + wG\theta_T(1-2f)\} \quad (33)$$

$$i_{23} = \frac{n_e^2 F^2 v A \Gamma_T}{RT} * \frac{f(1-f)}{\left[1 - 2wG\theta_T f(1-f)\right]} \quad (34)$$

$$G = a_O + a_R - 2a_{OR} \quad (35)$$

$$w = n_O = n_R \quad (36)$$

$$\theta_T = \theta_R + \theta_O \quad (37)$$

$$f = \theta_O/\theta_T \quad (38)$$

Where a_O, a_R and a_{OR} are the constants of interaction between molecules of O, molecules of R, and molecules of O and R, respectively. n_O and n_R are the number of molecules of water displaced by one molecule of O or R. θ_O is the coverage of the oxidized molecules on the surface; θ_R is the coverage of the reduced molecules on the surface; f is the fraction of the oxidized molecules on the surface. The non-ideality of the voltage-current relationship is mainly reflected by the parameter $wG\theta_T$. If $wG\theta_T = 0$, then the results from ***Supplementary Eqns. 33-38*** is equivalent to the results from ***Supplementary Eqn. 31***.

For Aza-CMP-NiFe, the current-potential data calculated using the values of $A\Gamma_T = 1.8 \times 10^{-8}$ mol, $v = 0.05$ V s^{-1}, $E_O^{0'} = 1.395$ V, $E_R^{0'} = 1.315$ V, and $wG\theta_T$ ranging from 0 to 0.75 are represented by the points plotted in ***Supplementary Fig. 116***. With $wG\theta_T = 0.25$, the agreement with the experimental curve is excellent. Similar calculations were also performed for Aza-CMP-Ni. As shown in ***Supplementary Fig. 117***, with $A\Gamma_T = 2.3 \times 10^{-8}$ mol, $v = 0.05$ V s^{-1}, $E_O^{0'} = 1.352$ V, $E_R^{0'} = 1.287$ V, the fitting result when $wG\theta_T = 1.35$ best matches the experimental data. The presence of interaction forces between the adsorbed molecules leads to a change in shape; the higher $wG\theta_T$ value (i.e., the narrower and sharper peak) for Aza-CMP-Ni indicates that attractive forces predominate in Ni-sites. Although it is challenging to quantitatively compare the differences in interactions, we still qualitatively found that incorporating Fe altered the interaction between surface species at the

molecular level, suggesting that the binding of Fe sites occurred at the level of molecular adsorption. In summary, the analysis of $Ni^{2+/3}$ redox indicated that Aza-CMP-Ni and Aza-CMP-NiFe could be treated as adsorbed Ni/Ni-Fe monolayers (loading of 1.8×10^{-8} mol cm^{-2} and 2.3×10^{-8} mol cm^{-2}, respectively), which further supports the molecular nature of Ni sites and Ni-Fe sites.

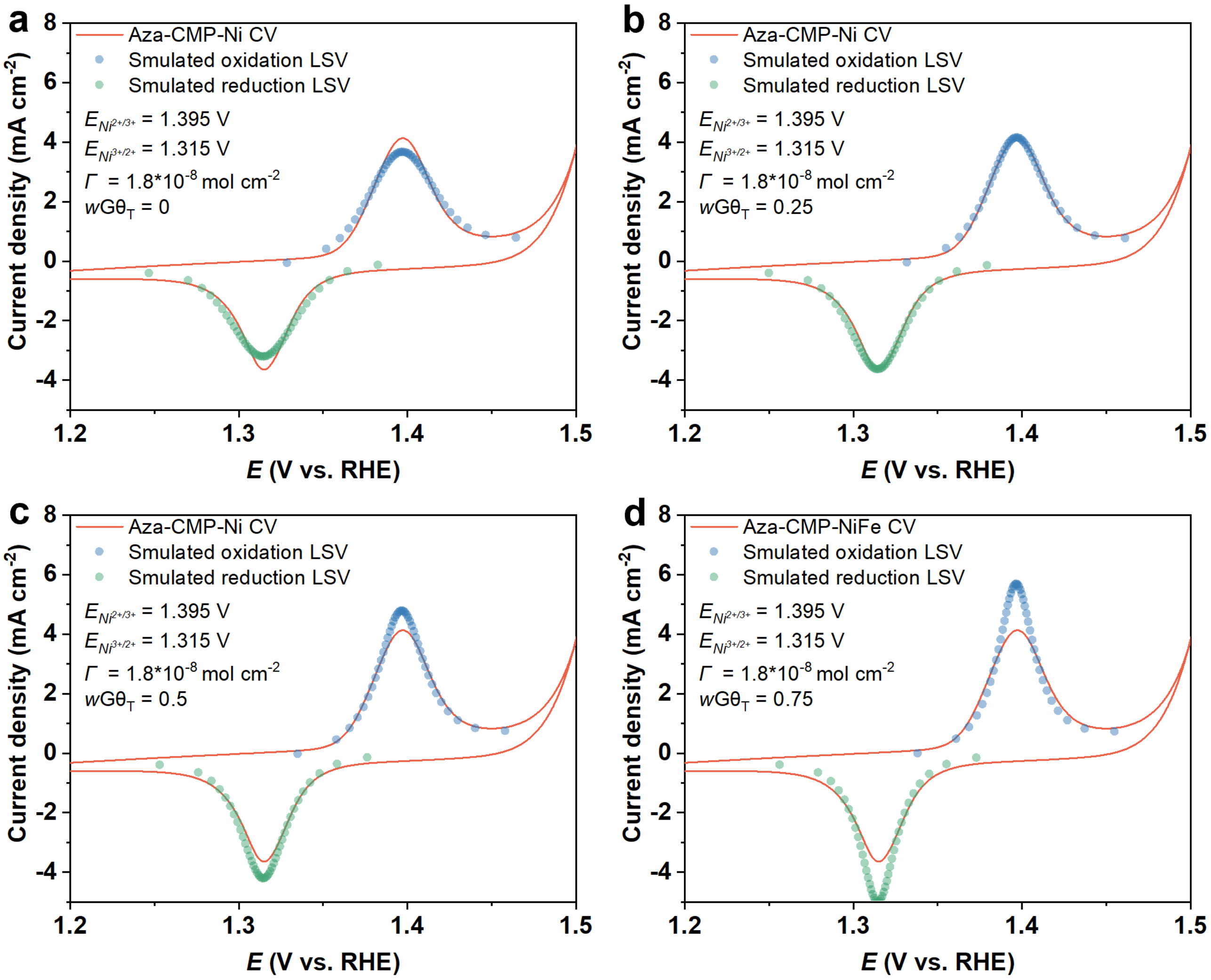

Supplementary Fig. 116 Calculated LSV data of $Ni^{2+/3+}$ region for Aza-CMP-NiFe based on *Supplementary Eqns. 33-38*. Experimental CV was obtained from 1.0 M NaOH with a scan rate of 50 mV s^{-1}). (a) $wG\theta_T = 0$. (b) $wG\theta_T = 0.25$. (c) $wG\theta_T = 0.5$. (d) $wG\theta_T = 0.75$.

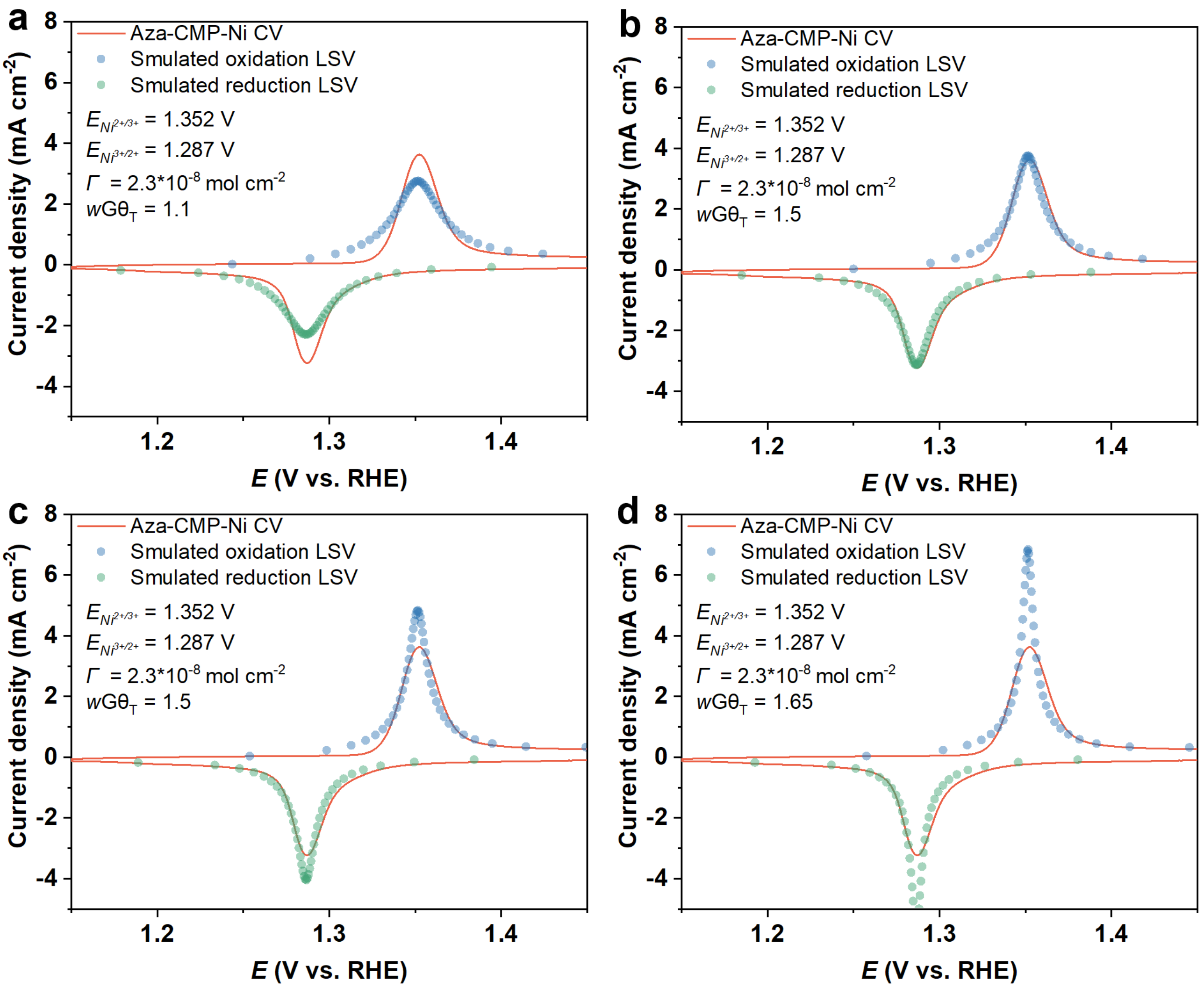

Supplementary Fig. 117 Calculated LSV data of $Ni^{2+/3+}$ region for Aza-CMP-Ni based on *Supplementary Eqns. 33-38.* Experimental CV was obtained from 1.0 M NaOH with a scan rate of 50 mV s^{-1}). (a) $wG\theta_T = 1.1$. (b) $wG\theta_T = 1.35$. (c) $wG\theta_T = 1.5$. (d) $wG\theta_T = 1.65$.

3.2 OER at the foot of the wave area

The OER current-potential behavior at the foot of the wave area is simulated according to our previous report (***Supplementary Eqn. 39***).[3]

$$i_{\mathrm{OER}} = 4k_{\mathrm{ap}}\mathrm{F}A\Gamma_{\mathrm{o}}^{'} = 4k_{\mathrm{ap}}\mathrm{F}A\frac{\Gamma_{\mathrm{T}}\left\{1-\exp\left\{-\exp\left[\frac{(1-\alpha)\mathrm{F}}{\mathrm{R}T}\left(E-E_{\mathrm{p}}\right)\right]\right\}\right\}}{1+e^{\left(E-E^{0''}\right)\mathrm{F}/\mathrm{R}T}} \tag{39}$$

Supplementary Fig. 118a displays the calculated LSV data using the value of $A\Gamma_T = 1.8 \times 10^{-8}$ mol, α = 0.5, E_p = 1.395 V, $E^{0''}$ = 1.77 V, (note that $E^{0''}$ was determined from the redox waves under non-aqueous conditions in ***Supplementary Fig. 118b***; $E^{0''} = E_p + 0.377$ V). The values of the apparent rate constant k_{ap} should range from 20000 s^{-1} to 26000 s^{-1} according to the simulation, indicating extremely fast kinetics, even higher than those of molecular catalysts[106] (note that the determination of k_{ap} will dramatically change with different $E^{0''}$; thus, the value obtained here is not the strictly apparent rate constant, as $E^{0''}$ is obtained in a non-aqueous solvent).

Supplementary Notes 3

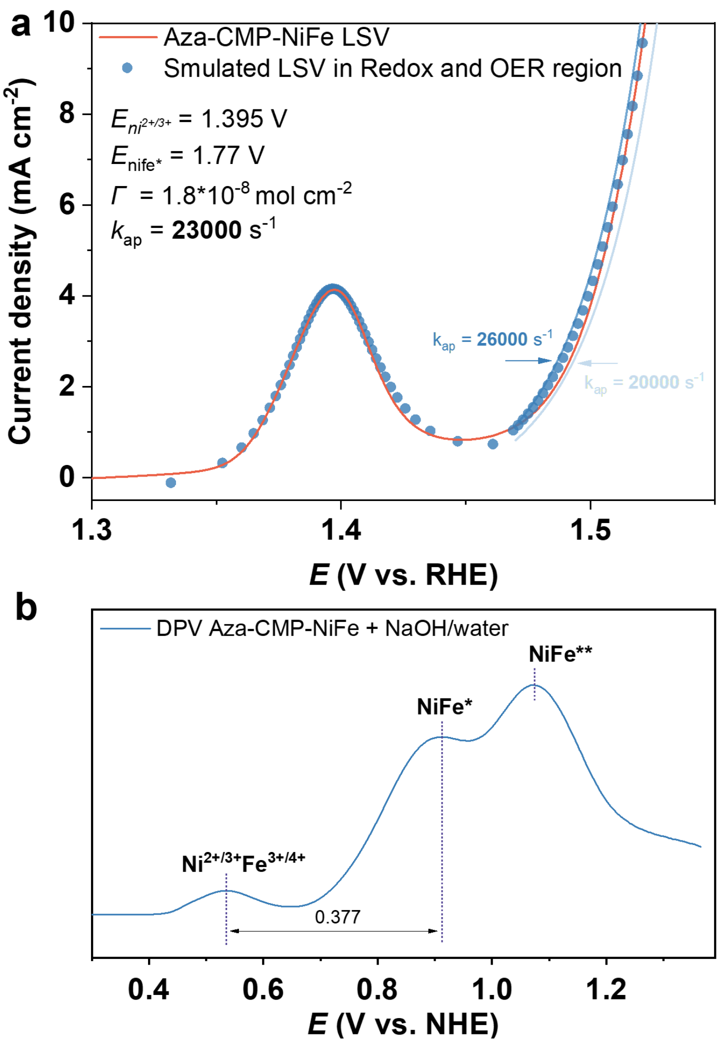

Supplementary Fig. 118 OER at the foot of the wave area. (a) Calculated forward scan LSV in $Ni^{2+/3+}$ and OER region for Aza-CMP-NiFe (scan rate, 50 mV s^{-1}). The OER current was calculated using ***Supplementary Eqn. 39.*** (b) DPV curves of Aza-CMP-NiFe/CP in water/NaOH added acetonitrile electrolytes.

Supplementary Table 8 Glossary of symbols used in this section.

i_{23}	Current intensity contributes from $Ni^{2+/3+}$	E_p	Peak potential
i_{OER}	Current intensity contributes from OER	$E_{p,O}$	Peak potential of oxidation wave
n_e	Number of electrons transferred	$E_{p,R}$	Peak potential of reduction wave
F	Faradaic constant	$E^{0\prime}$	Standard potential of a redox couple
v	Scan rate	$E_O^{0\prime}$	Standard potential of oxidation ($Ni^{2+/3+}$)
A	Geometric surface of the electrode	$E_R^{0\prime}$	Standard potential of reduction ($Ni^{3+/2+}$)
α	transfer coefficient	$\Delta E_{p,1/2}$	Width at half-height of redox wave
R	Gas constant	θ_O	Coverages of the oxidized molecules on the surface
T	Temperature	θ_R	Coverages of the reduced molecules on the surface
G	Global attraction constant	w	Number of water molecules displaced by one molecule of O or R
f	Fraction of the oxidized molecules on the surface	Γ_T	Surface concentration of catalyst
E	Electrode potential	Γ_T'	Concentration of catalysts that participated in the OER
k_{ap}	Apparent rate constant	$E^{0\prime\prime}$	Formal potential of $NiFe^*$

Supplementary Notes 4

Supplementary Notes 4: pH dependency of OER activity

OER catalysts with pH-dependent activity on the RHE scale have recently been reported.[96, 101, 107, 108, 109, 110] Measuring the pH dependence of the OER activity could provide insights into the proton-electron transfer features of RDS in the specific reaction pathways. Different mechanisms have been proposed for catalysts exhibiting pH-dependent OER activity. This behavior has been explained by different mechanisms, e.g., the exchange of catalyst lattice oxygen (for perovskite-type cobalt oxides),[96, 107] pH-dependent deprotonations leading to the formation of negatively charged oxygenated intermediates "active oxygen sites" (for Fe/Ni- and Mn-based oxides),[101, 108] and pH-dependent redox processes generating additional catalytically active sites (for RuO_2).[109] Moreover, pH-dependent OER activity has also been observed for homogeneous catalysts produced by the radical coupling of two metal oxo-species with oxyl radical character.[111]

The RDS of a specific water oxidation catalysis system may be classified into three types: electron transfer (ET) controlled RDS, proton transfer (PT) controlled RDS, and concerted proton-electron transfer (CPET) controlled RDS. The order of reaction of OER activity in pH shows how OER kinetics depend on proton activity. When excluding the change in the number of catalytic sites under different pH,[112] measuring the pH dependence of the OER activity could provide insights into the proton-electron transfer features of RDS in the specific reaction pathways. Theoretically, a zeroth-order reaction would imply no dependence, which is usually observed in catalysis that is controlled by a CPET step; when a sequential proton-electron transfer step is the rate-determining step (proton transfer and electron transfer steps are decoupled), an OER catalyst could exhibit pH-dependent OER activity. Fractional reaction orders may also be expected when the mechanism consists of complex chain/side reactions.[113, 114, 115, 116]

4.1 Theoretical explanation of pH-dependent OER activity

To gain insights into the origin of the pH dependence of the catalytic current, the pH-dependent OER activity (i.e., reaction order of pH) is quantitatively analyzed by rational quantitative CV analysis as established below.[3] To simplify the analysis, we assume that the pre-RDS reaction $\mathrm{M^{n+} - e^- \rightarrow M^{(n+1)+}}$ is oxidized in an irreversible one-step, one-electron reaction, and $\mathrm{M^{(n+1)+}}$ will be consumed for OER by another irreversible reaction $\mathrm{M^{(n+1)+} - e^- \rightarrow M^{(n+2)+}}$ that was assigned as the RDS for the whole reaction. Then, the expected current-potential behavior of the pre-RDS reaction is given by the following equation:[3, 117]

$$i_{\text{pre}} = \left(\frac{\mathrm{F}^2}{\mathrm{R}T}\right)(1-\alpha)v\Gamma * \exp\left[\frac{(1-\alpha)\mathrm{F}}{\mathrm{R}T}\left(E - E_{\text{p}}\right)\right] * \exp\left\{-\exp\left[\frac{(1-\alpha)\mathrm{F}}{\mathrm{R}T}\left(E - E_{\text{p}}\right)\right]\right\} \quad (40)$$

The OER current-potential behavior based on the RDS reaction in the foot of the wave area could be written as the equation below:[3]

$$i_{\text{OER}} = 4k_{\text{ap}}[a]\mathrm{F}\Gamma \frac{\left\{1 - \exp\left\{-\exp\left[\frac{(1-\alpha)\mathrm{F}}{\mathrm{R}T}\left(E - E_{\text{p}}\right)\right]\right\}\right\}}{1 + \exp\left[(E - E_{\text{r}})\mathrm{F}/\mathrm{R}T\right]} \quad (41)$$

Where F is the Faradaic constant, k_{ap} is the apparent rate constant, v is scan rates, [a] is the activity of the reactant in solution, Γ is the surface concentration of the catalyst, α is the transfer coefficient of $\mathrm{M^{n+}}$ oxidation reaction, R is the gas constant, T is the reaction temperature, E is the electrode potential, E_{p} is the peak potential of $\mathrm{M^{n+}}$ oxidation reaction, and E_{r} is the peak potential of $\mathrm{M^{(n+1)+}}$ oxidation reaction. ***Supplementary Fig. 119*** shows the demonstrative LSV calculation at different pH levels, in which k_{ap} was set to 10 s^{-1}, v = 0.01 V s^{-1}, $\Gamma = 5\times10^{-8}$ mol, α = 0.5, E_{p} = 1.35 V vs. RHE (pH = 14), and E_{r} = 1.60 V vs. RHE (pH = 14). To further simplify the simulation, the pre-RDS reaction was

defined as a $1H^+/1e^-$ redox that the potential will not change with pH at the RHE scale. All calculations do not involve changes in intermediate pK_a.

PT-controlled RDS. The RDS should be at least a $1H^+/1e^-$ oxidation; thus, the value of E_r would not change with pH at the RHE scale. The pH dependence is, therefore, induced by changes in substrate concentration [a]. As shown in ***Supplementary Fig. 119d***, the potential requirements to reach a specific current density shifted with pH values; a slope of approximately –61 mV pH^{-1} was anticipated. Meanwhile, the reaction order of pH was calculated to be 1 at different potentials (***Supplementary Fig. 119g***).

ET-controlled RDS. The RDS is not related to proton transfer and also the change of proton activity; thus, [a] in ***Supplementary Eqn. 41*** is neglected. The value of E_r, in this case, would be constant at the NHE scale and thus change with pH at the RHE scale (–59 mV pH^{-1}). The pH dependence is therefore induced by changes in E_r. As shown in ***Supplementary Fig. 119e***, the relationship between potential requirements and pH exhibits a slope of +59 mV pH^{-1}; the reaction order of pH is calculated to be –1 at different potentials, which indicates an inverse pH effect that the reaction activity decreases when increasing the pH of the electrolyte (***Supplementary Fig. 119h***).

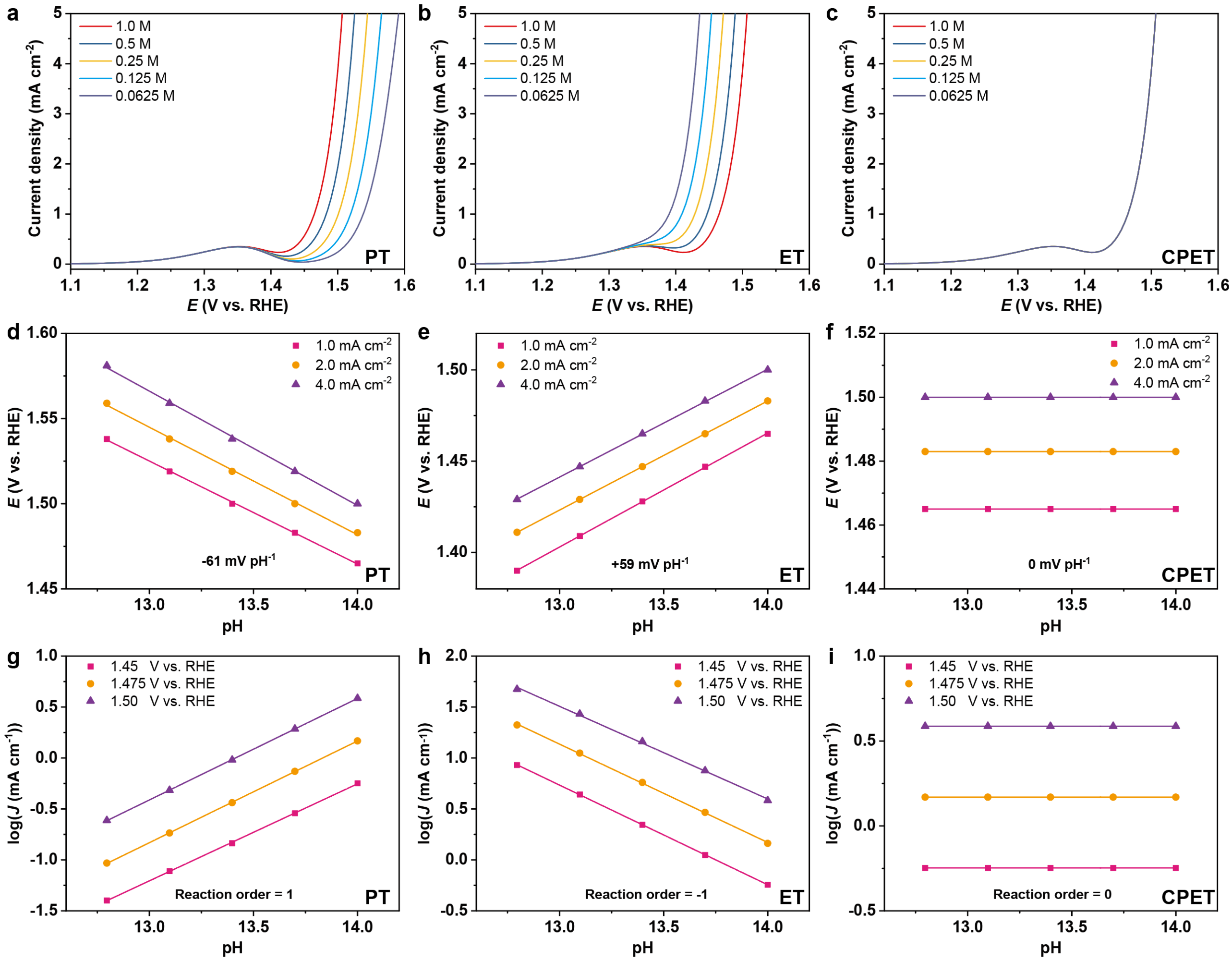

Supplementary Fig. 119 pH dependence simulation. Simulated LSV at different concentrations of $[OH^-]$ **using *Supplementary Eqns. 40* and *41***: (a) RDS reaction is a PT step; (b) RDS reaction is an ET step; (c) RDS reaction is a CPET step. The pH-dependent OER activity at different current densities: (d) RDS reaction is a PT step; (e) RDS reaction is an ET step; (f) RDS reaction is a CPET step. The reaction order of pH at different potentials: (g) RDS reaction is a PT step; (h) RDS reaction is an ET step; (i) RDS reaction is a CPET step.

Supplementary Notes 4

CPET-controlled RDS. The value of E_r would not change with pH at the RHE scale, and the reaction rate was not affected by the change of proton activity since electron transfer would never be decoupled with proton transfer. Thus, the slope of the potential-pH relationship is 0 mV pH^{-1}, and the reaction order of pH is 0 (***Supplementary Figs. 119f*** and ***119i***).

Referring to the concept of Pourbaix, the relationship between pH and potential requirements to reach a specific current density during catalysis was plotted in ***Supplementary Fig. 120***. Reactions with ET-controlled RDS, PT-controlled RDS, and CPET-controlled RDS show significantly different slopes, which means that the properties of RDS might be identified from the linear pH dependence of activity. Under the RHE scale, a half-cell reaction that exhibits pH dependence inherently suggests the presence of decoupled proton-electron transfer pathways: a negative slope (positive reaction order of pH) indicates the domination of proton transfer in RDS; a zero slope (zero reaction order of pH) suggests a concerted PCET feature of RDS; a positive slope (negative reaction order of pH) implies that the RDS is controlled by an ET step.

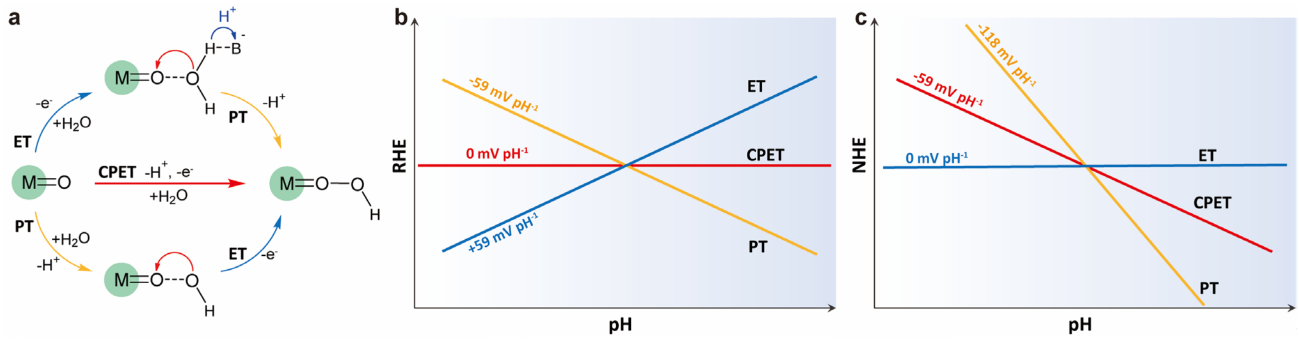

Supplementary Fig. 120 Relationship between pH and potential requirements. (a) Schematic of proton-coupled electron transfer processes in the O–O bond formation step. The pH-dependent catalytic activity of PT, ET, and CPET steps under (b) RHE and (c) NHE scale.

4.2 pH dependence of a decoupled PT-ET system

We next consider a complex system when the pH range is across the pK_a of RDS intermediates. More specifically, when the irreversible reaction $MH - e^- \rightarrow M + H^+$ that was assigned as the RDS for the whole reaction. We will discuss simple kinetic models for the situation with the oxidation of a molecule MH, in which proton transfer (or deprotonation) precedes ET (PTET, ***Supplementary Fig. 121*** and ***Supplementary Eqns. 42-43***).

$$MH \rightleftarrows H^+ + M^- \qquad (42)$$

$$M^- \xrightarrow{k_2} M + e^- \qquad (43)$$

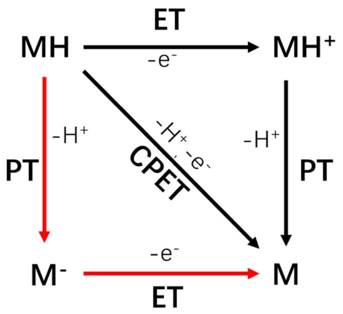

Supplementary Fig. 121 Scheme of PCET. Red arrows indicate the sequential PTET pathway.

Supplementary Notes 4

If in this mechanism, the reaction in ***Supplementary Eqn. 43*** is irreversible, the OER current-potential behavior based on the RDS reaction in the foot of the wave area could be written as the equation below:[118]

$$i = \mathrm{F}k_2[\mathrm{M}^-] \quad (44)$$

With a steady-state approximation, according to the equilibration between reaction intermediates, the generation rate of M^- is equal to its consumption rate.

$$k_{-1}[\mathrm{M}^-][\mathrm{H}^+] + k_2[\mathrm{M}^-] = k_1[\mathrm{MH}] \quad (45)$$

Where k_1 and k_{-1} are the rate constants corresponding to the reaction in ***Supplementary Eqn. 42***, k_2 is the rate constant for the reaction in ***Supplementary Eqn. 43***. Since the number of surface-active sites is fixed, thus:

$$[\mathrm{M}^-] + [\mathrm{MH}] = [\mathrm{MH}]^0 \quad (46)$$

Where $[\mathrm{MH}]^0$ is the initial concentration of MH. Together with ***Supplementary Eqn. 45*** and ***46***, the concentration of $[\mathrm{M}^-]$ can be written as:

$$[\mathrm{M}^-] = \frac{[M\mathrm{H}]^0}{1 + \frac{k_{-1}}{k_1}[\mathrm{H}^+] + \frac{k_2}{k_1}} \quad (47)$$

Thus,

$$i = \mathrm{F}k_2[\mathrm{M}^-] = \frac{\mathrm{F}k_2[\mathrm{MH}]^0}{1 + \frac{k_{-1}}{k_1}[\mathrm{H}^+] + \frac{k_2}{k_1}} = \frac{\mathrm{F}k_2^0[\mathrm{MH}]^0\exp\left[(\frac{(1-\alpha)\mathrm{F}(E - E^0_{\mathrm{M/M}})}{\mathrm{R}T})\right]}{1 + \frac{k_{-1}}{k_1}[\mathrm{H}^+] + \frac{k_2^0}{k_1}\exp\left[(\frac{(1-\alpha)\mathrm{F}(E - E^0_{\mathrm{M/M^-}})}{\mathrm{R}T})\right]} \quad (48)$$

Where k_2^0 is the standard rate constant for reaction in ***Supplementary Eqn. 43***, $E^0_{\mathrm{M/M^-}}$ is the peak potential for reaction in ***Supplementary Eqn. 43***, and α, F, R, and T have their usual meaning (note that all the potential E represents the voltage under the NHE scale).

During the deprotonation equilibrium:

$$k_1[\mathrm{MH}] = k_{-1}[\mathrm{M}^-][\mathrm{H}^+] \quad (49)$$

Considering that $10^{\mathrm{p}K_\mathrm{a}-\mathrm{pH}} = \frac{[\mathrm{MH}]}{[\mathrm{M}^-]}$, the expression for the current density in the steady-state approximation is:

$$i = \mathrm{F}k_2[\mathrm{M}^-] = \frac{\mathrm{F}k_2^0[\mathrm{MH}]^0\exp\left[(\frac{(1-\alpha)\mathrm{F}(E - E^0_{\mathrm{M/M^-}})}{\mathrm{R}T})\right]}{1 + 10^{\mathrm{p}K_\mathrm{a}-\mathrm{pH}} + \frac{k_2^0}{k_1}\exp\left[(\frac{(1-\alpha)\mathrm{F}(E - E^0_{\mathrm{M/M^-}})}{\mathrm{R}T})\right]} \quad (50)$$

When this system is analog to a nearly real catalytic water oxidation reaction, we assume that the pre-RDS reaction $\mathrm{MOH} \rightarrow \mathrm{MO} + \mathrm{e}^- + \mathrm{H}^+$ is oxidized in an irreversible concerted $1\mathrm{H}^+/1\mathrm{e}^-$ redox reaction, and MO will be consumed for OER via water nucleophilic attack pathway, by another irreversible reaction, $\mathrm{MOOH_2} \rightarrow \mathrm{MOOH} + \mathrm{e}^- + \mathrm{H}^+$, that is assigned as the RDS for the OER reaction. Therefore, the OER current-potential behavior in the foot of the wave area could be written as the equation below:

Supplementary Notes 4

$$i_{\mathrm{OER}} = \frac{\mathrm{F}k_{\mathrm{ET}}^{0}[\mathrm{MOOH_2}]^{0}\exp\left[\left(\frac{(1-\alpha)\mathrm{F}(E-E_{\mathrm{MOOH/MOOH^-}}^{0})}{\mathrm{R}T}\right)\right]}{1+10^{\mathrm{p}K_a-\mathrm{pH}}+\frac{k_{\mathrm{ET}}^{0}}{k_{\mathrm{PT}}}\exp\left[\left(\frac{(1-\alpha)\mathrm{F}(E-E_{\mathrm{MOOH/MOOH^-}}^{0})}{\mathrm{R}T}\right)\right]} \quad (51)$$

Where k_{PT} are the rate constants corresponding to the reaction of $\mathrm{MOOH_2} \rightarrow \mathrm{MOOH^-} + \mathrm{H^+}$, k_{ET}^{0} is the standard rate constant for the reaction of $\mathrm{MOOH^-} \rightarrow \mathrm{MOOH} + \mathrm{e^-}$, $E_{\mathrm{MOOH/MOOH^-}}^{0}$ is the peak potential for the reaction of $\mathrm{MOOH^-} \rightarrow \mathrm{MOOH} + \mathrm{e^-}$, $[\mathrm{MOOH_2}]^0$ is the initial concentration of $\mathrm{MOOH_2}$, and α, F, R, and T have their usual meaning (note that E represents the voltage under the NHE scale). The initial concentration of $\mathrm{MOOH_2}$ is determined by the pre-RDS reaction, which can be written as:[3]

$$[\mathrm{MOOH_2}]^0 = \Gamma\left\{1-\exp\left\{-\exp\left[\frac{(1-\alpha)\mathrm{F}}{\mathrm{R}T}(E-E_{\mathrm{MOH/MO}})\right]\right\}\right\} \quad (52)$$

The current contributed by irreversible pre-RDS oxidation is given in ***Supplementary Eqn. 40***. Thus, the overall current behavior is the sum of all currents.

$$i_{(\mathrm{NHE})} = i_{\mathrm{pre}} + i_{\mathrm{OER}} = \left(\frac{\mathrm{F}^2}{\mathrm{R}T}\right)(1-\alpha)\upsilon\Gamma * \exp\left[\frac{(1-\alpha)\mathrm{F}}{\mathrm{R}T}(E-E_{\mathrm{MOH/MO}})\right] * \exp\left\{-\exp\left[\frac{(1-\alpha)\mathrm{F}}{\mathrm{R}T}(E-E_{\mathrm{MOH/MO}})\right]\right\}$$
$$+\frac{\mathrm{F}k_{\mathrm{ET}}^{0}\Gamma\left\{1-\exp\left\{-\exp\left[\frac{(1-\alpha)\mathrm{F}}{\mathrm{R}T}(E-E_{\mathrm{MOH/MO}})\right]\right\}\right\} * \exp\left[\left(\frac{(1-\alpha)\mathrm{F}(E-E_{\mathrm{MOOH/MOOH^-}}^{0})}{\mathrm{R}T}\right)\right]}{1+10^{\mathrm{p}K_a-\mathrm{pH}}+\frac{k_{\mathrm{ET}}^{0}}{k_{\mathrm{PT}}}\exp\left[\left(\frac{(1-\alpha)\mathrm{F}(E-E_{\mathrm{MOOH/MOOH^-}}^{0})}{\mathrm{R}T}\right)\right]} \quad (53)$$

The NHE potential is related to the RHE potential by:

$$E_{\mathrm{NHE}} = E_{\mathrm{RHE}} + \frac{\mathrm{R}T}{\mathrm{F}}ln[\mathrm{H^+}] \quad (54)$$

Thus, when using E at the RHE scale, the equation for the current-potential behavior becomes (note that the value of $E_{\mathrm{MOH/MO}}$ and $E_{\mathrm{MOOH/MOOH^-}}^{0}$ in the equation still indicates the oxidation potential at the NHE scale):[118]

$$i_{(\mathrm{RHE})} = i_{\mathrm{pre}} + i_{\mathrm{OER}}$$
$$= \left(\frac{\mathrm{F}^2}{\mathrm{R}T}\right)(1-\alpha)\upsilon\Gamma * (10^{-\mathrm{pH}})^{1-\alpha} * \exp\left[\frac{(1-\alpha)\mathrm{F}}{\mathrm{R}T}(E-E_{\mathrm{MOH/MO}})\right] * \exp\left\{-(10^{-\mathrm{pH}})^{1-\alpha} * \exp\left[\frac{(1-\alpha)\mathrm{F}}{\mathrm{R}T}(E-E_{\mathrm{MOH/MO}})\right]\right\}$$
$$+\frac{\mathrm{F}k_{\mathrm{ET}}^{0}\Gamma\left\{1-\exp\left\{-(10^{-\mathrm{pH}})^{1-\alpha} * \exp\left[\frac{(1-\alpha)\mathrm{F}}{\mathrm{R}T}(E-E_{\mathrm{MOH/MO}})\right]\right\}\right\} * (10^{-\mathrm{pH}})^{1-\alpha} * \exp\left[\left(\frac{(1-\alpha)\mathrm{F}(E-E_{\mathrm{MOOH/MOOH^-}}^{0})}{\mathrm{R}T}\right)\right]}{1+10^{\mathrm{p}K_a-\mathrm{pH}}+\frac{k_{\mathrm{ET}}^{0}}{k_{\mathrm{PT}}} * (10^{-\mathrm{pH}})^{1-\alpha} * \exp\left[\left(\frac{(1-\alpha)\mathrm{F}(E-E_{\mathrm{MOOH/MOOH^-}}^{0})}{\mathrm{R}T}\right)\right]} \quad (55)$$

Supplementary Figs. 122a and ***122b*** depict the current-potential behavior as a function of pH, measured under both NHE and RHE scales, in which k_{ET}^{0} and k_{PT} were set to 10 and 1 $\mathrm{s^{-1}}$, respectively, v = 0.01 V $\mathrm{s^{-1}}$, Γ = 5×10^{-8} mol, α = 0.5, $\mathrm{p}K_\mathrm{a} = 11.5$, $E_{\mathrm{MOH/MO}}$ = 1.35 V vs. RHE (pH = 14, CPET process) and $E_{\mathrm{MOOH/MOOH^-}}^{0}$ = 1.60 V vs. RHE (pH = 14, ET process). Under the NHE scale, the reaction exhibits pH-independent overpotential requirements to achieve a specific current density when pH > pK_a, as the $\mathrm{M^-}$ has reached saturation. However, when pH < pK_a, the reaction kinetics are constrained by the deprotonation of MH. Thus, the correlation between potential requirements and pH displays a slope of +114 mV $\mathrm{pH^{-1}}$, suggesting a PT-controlled RDS

(***Supplementary Figs. 122d***). There are no fundamental discrepancies between plots under the NHE and RHE scales. Employing the RHE reference, the relationship between potential requirements and pH demonstrates a slope of +57 mV pH^{-1} when pH > pK_a, whereas the slope shifts to –57 mV pH^{-1} when pH < pK_a (***Supplementary Figs. 122c***). It is worth noting that the corresponding reaction order under the RHE scale does not conform to an integer number (***Supplementary Figs. 122e***). When pH < pK_a, the reaction order with respect to $[OH^-]$ is positive, and conversely, it becomes negative when pH > pK_a. These fractional reaction orders bear a strong correlation to the rate constants of proton transfer (k_{PT}) and electron transfer (k_{ET}).

For water oxidation systems that involve deprotonation and electron transfer processes, a necessary condition for validating an RDS with decoupled proton-electron transfer is the pH dependence of catalytic activity (current) on the RHE scale. This dependence does not follow a straightforward linear relationship (i.e., heightened activity with increased pH) but is associated with the RDS characteristics. In an ET-controlled RDS, an elevated pH signifies that the rate of ET is evaluated at an increasingly higher overpotential due to the pH-dependent reference potential, consequently resulting in decreased activity at the same overpotential. Thus, for a decoupled PT-ET system, the optimized pH yielding the highest performance coincides with the pK_a value of the protonated intermediates in the RDS. The aforementioned analysis also indicates that the reaction order of activity in relation to pH need not be an integer. In fact, for a typical water oxidation system, the reaction order is predominantly a fraction.[119] Therefore, relying solely on the reaction order to ascertain the participation of protons or electrons may not yield accurate results since the reaction properties of protonated intermediates are hard to obtain directly. The pH-activity dependency serves as a crucial tool in determining whether the RDS of water oxidation is a concerted or decoupled PCET process. Concurrently, the choice of reference electrode in the experiment carries significant importance. Therefore, it is essential to clearly annotate the reference electrode utilized in the pH dependence measurements.

This pK_a-induced, volcano-shaped activity plot has been experimentally identified in both homogeneous[120, 121] and heterogeneous[122, 123] systems. In this work, we also observed this volcano-type pattern in water oxidation using Aza-CMP-Ni. As illustrated in ***Supplementary Figs. 122f***, the potential of the oxidation current remains consistent as the pH decreases from 14 to 12.5, subsequently escalating significantly at lower pH values. Such behavior is almost identical to that depicted in ***Supplementary Figs. 122d***, suggesting that water oxidation on Aza-CMP-Ni follows a decoupled PT-ET process, wherein the pK_a of the key intermediates, NiO–OH_2, is approximately 12.5.

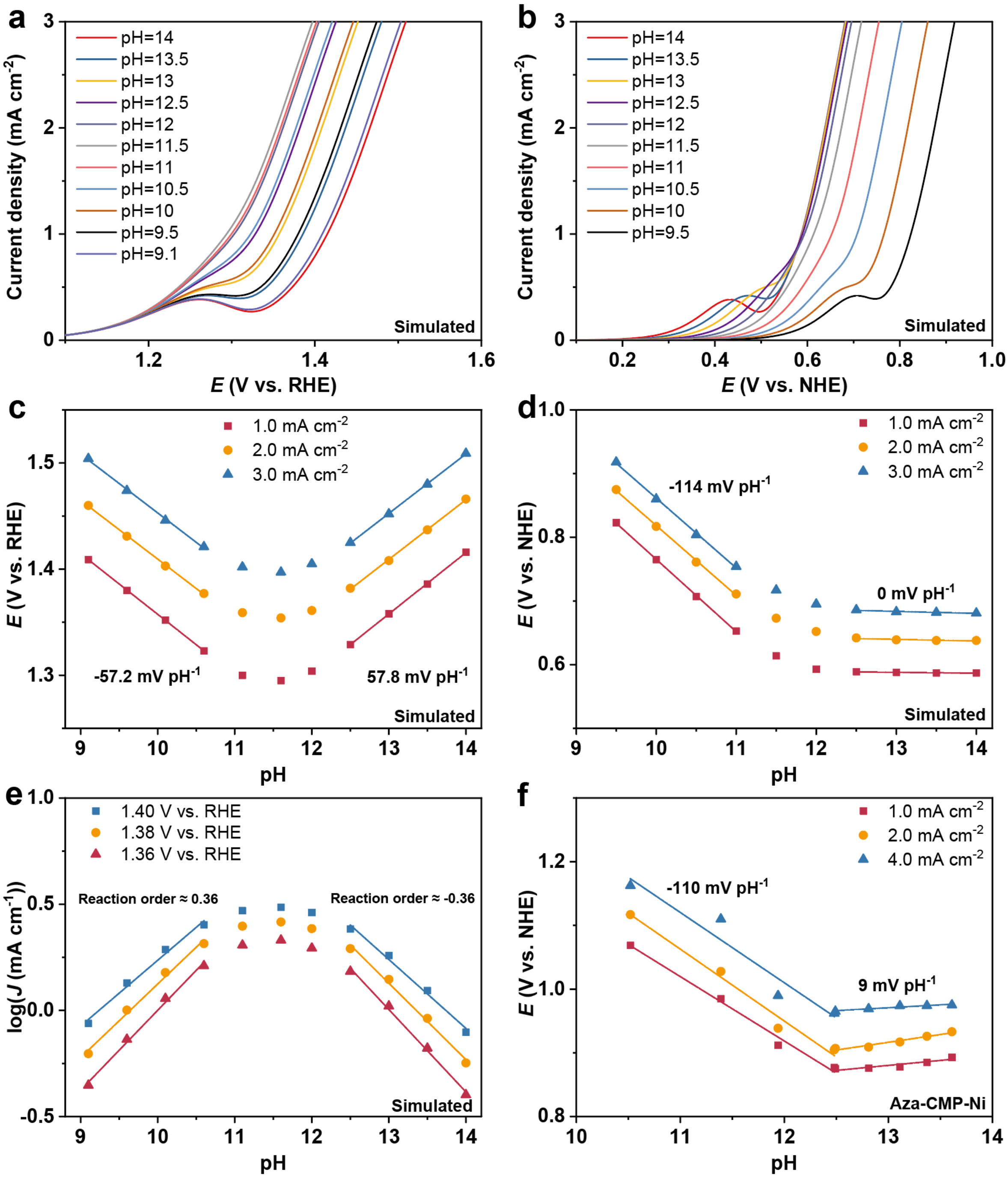

Supplementary Fig. 122 Simulated current-potential behavior at different pH using *Supplementary Eqns. 53* and *55*. (a) LSV under RHE scale; (b) LSV under NHE scale. The pH-dependent OER activity at different current densities: (c) RHE scale; (d) NHE scale. (e) The reaction order of pH at different potentials under the RHE scale. (f) The experimental pH-dependent OER activity of Aza-CMP-Ni at different current densities.

4.3 Pre-RDS oxidation limited pH dependence

In practical catalysis, the pH dependence of the catalytic current may also be constrained by the pre-RDS redox. When assuming a situation that the pre-RDS oxidation $M^{n+} - e^- \rightarrow M^{(n+1)+}$ is a $2H^+/1e^-$ transfer process, and the RDS oxidation $M^{(n+1)+} - e^- \rightarrow M^{(n+2)+}$ is a single electron transfer process. Thus, under the NHE scale, E_p would show a super-Nernst shift (–118 mV pH^{-1}) with pH, and E_r would be constant. It is a consensus that the value of E_p must be less than that of E_r (i.e., $M^{(n+1)+}$ still needs more driving force to reach a higher oxidation state of $M^{(n+2)+}$). Therefore, with the increase of E_p when

decreasing the pH, E_r would eventually shift with pH, even though it is an ET step. When reflected on the catalytic current, the current would exhibit a pH dependence for an ET-controlled RDS (***Supplementary Figs. 123a***). ***Supplementary Figs. 123b-g*** summarized the possible pH dependence of pre-RDS redox and catalytic current. When the slope of the pre-RDS redox is larger than that of the catalytic current, the inflection point may occur due to the constrained potential by the shifted pre-RDS redox, which makes it unreliable to conclude the properties of RDS via pH dependence of current (***Supplementary Figs. 123b-d***). Therefore, additional evidence (like kinetic isotopic effects) would be required to screen the nature of RDS. The detailed criteria for the pH dependency of OER activity are listed in ***Supplementary Fig. 124***. Features of both pre-RDS and RDS redox would affect the determination of proton and electron transfer properties in RDS, which will further affect the inference of the entire catalytic cycle. Especially for ET-controlled RDS (like the radical coupling mechanism for O–O bond formation), the pH-current relationship is only a sufficient but not necessary condition, which needs evidence for KIE experiments to make a solid distinguishment.

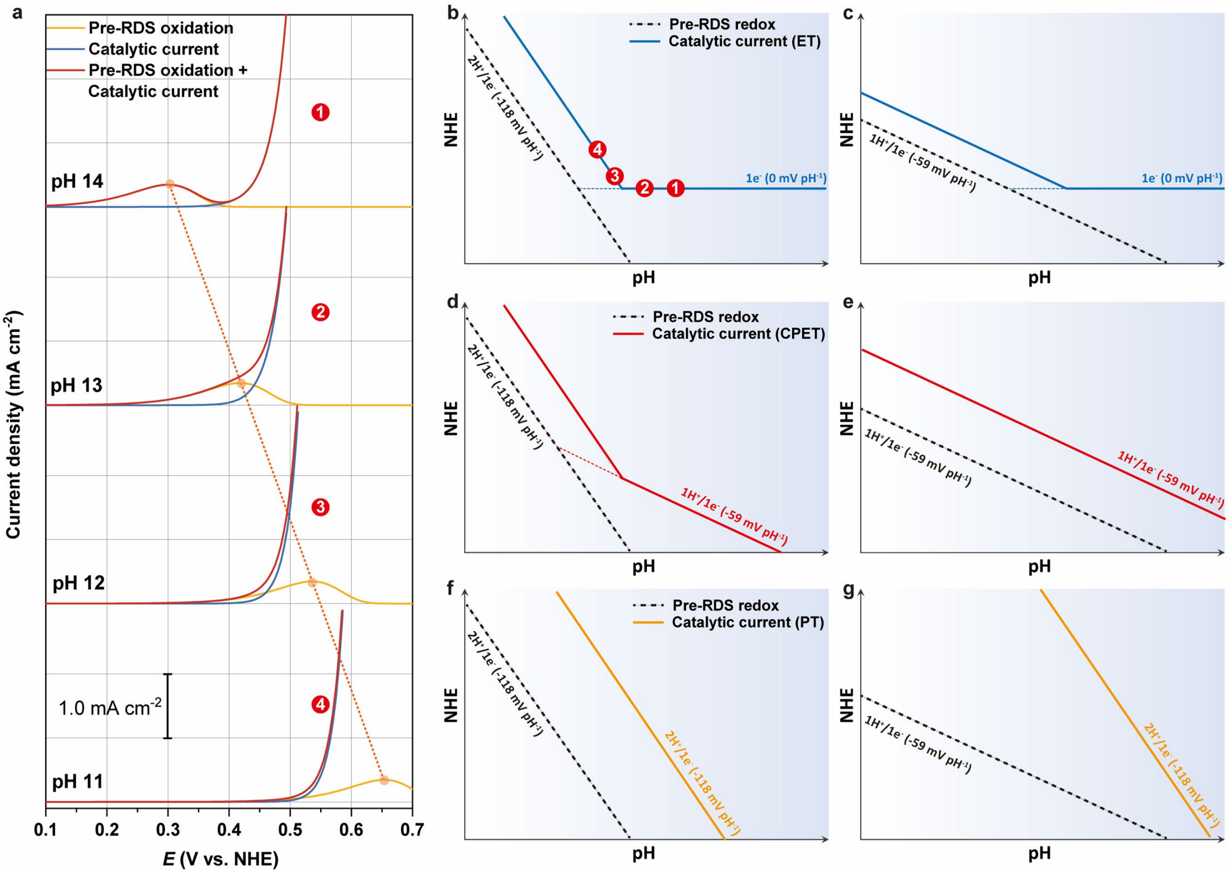

Supplementary Fig. 123 Simulated current-potential behavior at different pH using *Supplementary Eqns. 40* and *41*. (a) Simulated LSV curves of catalytic events that have an ET-controlled RDS and pre-RDS oxidation with super-Nernst behavior ($2H^+/1e^-$). (b) Schematic pH response of a pre-RDS redox with a super-Nernst behavior and catalytic current with an ET-controlled RDS. (c) Schematic pH response of a pre-RDS redox with a Nernst behavior and catalytic current with an ET-controlled RDS. (d) Schematic pH response of a pre-RDS redox with a super-Nernst behavior and catalytic current with a CPET-controlled RDS. (e) Schematic pH response of a pre-RDS redox with a Nernst behavior and catalytic current with a CPET-controlled RDS. (f) Schematic pH response of a pre-RDS redox with a super-Nernst behavior and catalytic current with a PT-controlled RDS. (g) Schematic pH response of a pre-RDS redox with a Nernst behavior and catalytic current with a PT-controlled RDS.

Supplementary Notes 4

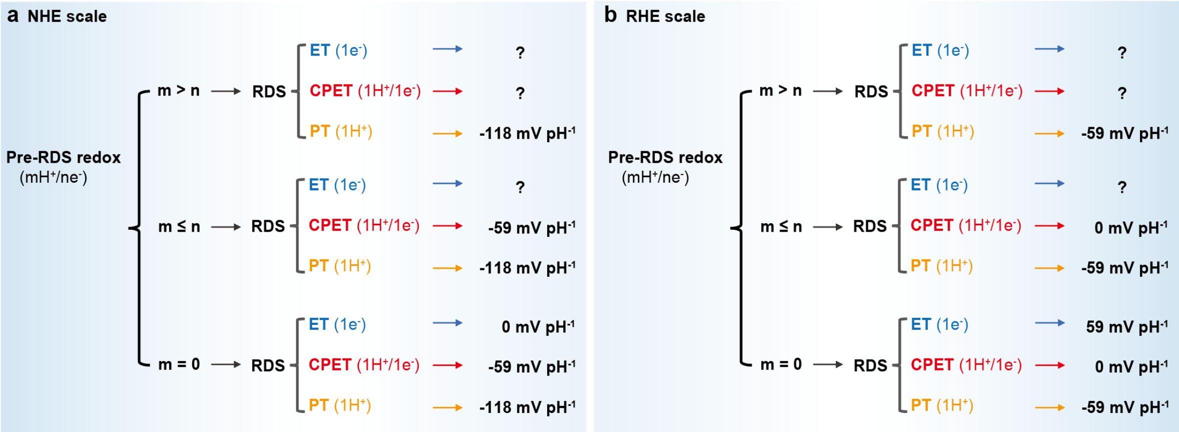

Supplementary Fig. 124 Slopes of pH-current relationship. (a) NHE scale and (b) RHE scale (question mark indicates that the slope may change over a specific pH range).

The pre-RDS limited-pH dependence was verified experimentally in our case of Aza-CMP-NiFe. As illustrated in ***Supplementary Fig. 125a***, the slope of the $Ni^{2+/3+}$ redox in Aza-CMP-NiFe is −89 mV pH^{-1}, aligning with a three-proton/two-electron transfer process ($3H^+/2e^-$). The current-pH dependency under alkaline conditions demonstrated a slope of −53 mV pH^{-1}, suggesting a CPET-controlled RDS during water oxidation catalysis. However, an inflection point appeared around a pH of 12.5 in the current-pH curve, followed by an increased slope of −89 mV pH^{-1} upon further pH reduction. In the classical interpretation, this process would be ascribed to the $3H^+/2e^-$ transfer process. Such a process implies a transfer of six protons and four electrons from the initial state to trigger water oxidation, which is infeasible. A more plausible interpretation posits that the pH dependence of the water oxidation catalytic current is contingent on the pH dependence of the pre-RDS oxidation. This system still necessitates an external driving force exceeding 100 mV to prompt the pre-RDS state towards OER, which results in a slope change owing to the potential constraint by the super-Nernst shifted pre-RDS redox. In the case of Aza-CMP-NiFe, practical measurements fully corroborate the preceding theoretical analysis, thereby validating the crucial roles of pre-RDS steps in the pH dependence test. Hence, prior to conducting a pH-dependent test of the catalyzed current, it is highly recommended to investigate the pH-potential dependency of the pre-RDS redox peak and explore potential constraints between the two pH dependencies.

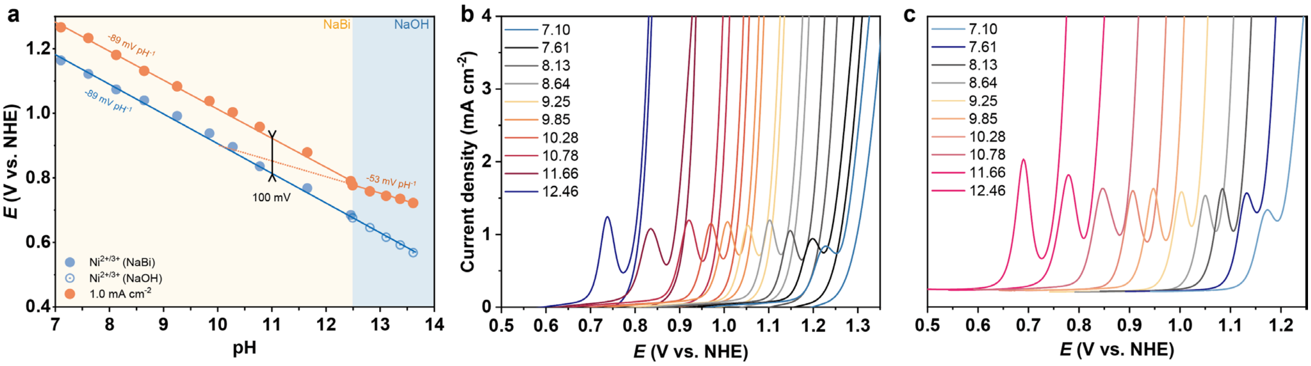

Supplementary Fig. 125 Pre-RDS limited pH dependence. (a) pH dependency of pre-RDS redox potential and catalytic current of Aza-CMP-NiFe under NHE scale. The oxidation peak potential was extracted from the DPV data, while the catalytic current was obtained from the CV curves. (b) CV and (c) DPV curves of Aza-CMP-NiFe in different pH conditions (0.5 M borate buffered solutions, CV scan rate: 50 mV s^{-1}).

Supplementary Notes 5

Supplementary Notes 5: Shapes of proton inventory curves

The proton inventory method is especially beneficial in enzymology for studying the kinetics of proton delivery to active enzymatic centers.[124, 125] This involves measuring the kinetic isotope effects on proton transfer reactions in mixed deuterium D_2O-H_2O as a function of solvent deuterium content. In a proton inventory experiment, the rate of isotope effects on proton transfer reactions in mixtures of protium and deuterium oxides is seldom a linear function of solvent deuterium content when the fraction of deuterium in the solvent is different from that of reactants and transition states. This behavior results in curved plots of isotope effects versus solvent deuterium content, which have a characteristic sag towards isotope effects of unity. The interpretation of the proton inventory results is also quite straightforward in most simple systems. In short, the form of the curve that relates rates v_n in each solvent with a deuterium fraction of n conveys the number of active protonic sites. A linear curve indicates a single Φ site, a quadratic curve for two Φ sites, a cubic curve for three Φ sites, and so on, culminating in an exponential curve for the "infinite-site" model.[125] Meanwhile, when considering the solvent isotope effects that arise from small contributions at a large number of identical hydrogenic sites in weakly interacting water molecules (i.e., Z-sites), the shape of proton inventory curves also changes depending on the Z-sites effects: a bowl-shaped line with a normal isotope contribution or a dome-shaped line with an inverse isotope contribution.[124]

In contemporary electrochemical systems, proton inventories have been effectively employed in straightforward cases (such as proton relay effects, i.e., Z-sites effects) based on the abovementioned simplified models.[12, 73, 126] However, the O–O bond formation process within the hydrogen bond network and the catalyst surface involves the transfer of multiple protons and the formation of various reaction intermediates. Consequently, overly simplified models are inappropriate for complex water oxidation systems. In light of this, the design of a unique system for O–O bond formation should be contemplated and established, drawing on the well-established Kresge Gross-Butler methodology.

5.1 Proton inventory of O–O bond formation process

The fundamental expression of proton inventory effects was known as the Kresge Gross-Butler equation, which represents the isotope effects arising from a combination of pronounced isotope effects at internal or external Φ sites, and from medium effects contributed from Z-Sites.[124, 125, 127] By using the transition state simplification treatment, the ratio of two rate constants (i.e., kinetic isotopic effects) is approximately equal to a ratio of two equilibrium constants between the reactants and transition states.[127, 128] Then the kinetic problem thus becomes an equilibrium one:

$$\frac{k_{\mathrm{n}}}{k_{\mathrm{H}}} = \frac{K_{\mathrm{n}}^{\ddagger}}{K_{\mathrm{H}}^{\ddagger}} = \frac{\prod_i^{v}(1 - n + n\Phi_i^{\mathrm{T}})}{\prod_i^{v}(1 - n + n\Phi_i^{\mathrm{R}})} Z^n \qquad (56)$$

where $K_{\mathrm{H}}^{\ddagger}$ is the equilibrium constant between the reactants and transition states of the protic solution, $K_{\mathrm{n}}^{\ddagger}$ is the corresponding equilibrium constant in a solution containing a deuterium mole fraction of n, k_{H} is the kinetic rate constant in the protic solution, k_n is the kinetic rate constant in a solution containing a deuterium mole fraction of n, v is the number of hydrogenic sites involved in the reactant(s) or transition state(s), Φ_i^{T} and Φ_j^{R} are the isotopic fractionation factors for the hydrogenic site in the transition and reactant state(s), respectively. Z reflects the medium Z-effect.

Supplementary Notes 5

5.2 Curves of "MOOH₂" step

In a base-participated reaction with a reactant absorbed on the substrate, followed by a pre-equilibrium proton transfer to the solvent, the transition state of the reaction in which proton transfer is the RDS was shown in the following equation:

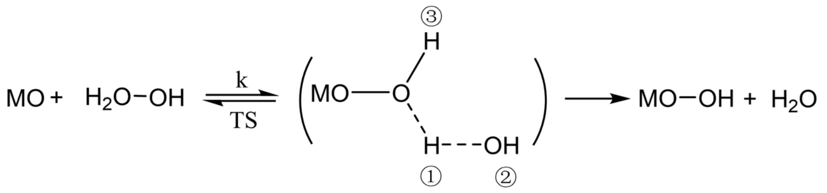

(57)

As the reactant of RDS in the adsorbate evolution mechanism (AEM) pathway, OH^- should be considered as an aggregated molecule in the form of the hydrogen-bonded network, solvated with a water molecule and potentially coordinated with a cation. When referring to the hydroxide aggregates, the OH^- part would be surrounded by several water molecules,[127] and the equilibrium between the reactants and transition state may be written as:

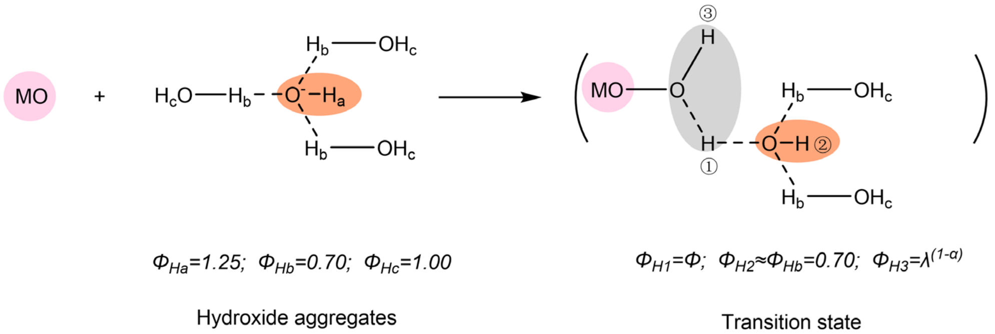

(58)

For reaction kinetics, the rate of reaction is controlled by the equilibrium between the reactants and the transition state. In this case, the transition state has three different kinds of hydrogen, in which proton 1 is in transit, and its fractionation factor was defined to Φ ($\Phi_{H1} = \Phi$); proton 2 belongs to the external proton acceptor (or water molecule network), and it is in the process of becoming hydrogens of the solvent water; proton 3 belongs to the un-transferred proton in water.

In hydroxide aggregates, proton c in the water molecules shows similar features to bulk water and thus has a unity fractionation factor. The loosening of the potential ($\Phi < 1$) on the "intermediate" b protons comes from the strong hydrogen bonding with negatively charged oxygen. For the internal proton a, the tightening of the internal stretching motion, presumably induced by the negative charge on oxygen, leads to a relatively high fractionation factor of 1.25.[127, 129] Inspired by the water solvation structure, we assume the fractionation factor of proton 2 remains unchanged ($\Phi_{H2} = \Phi_{Ha} = 1.25$) during the transition state due to the additional stabilization effect from the solvation waters. The structure of the water-like portion of this transition state (proton 3) will be intermediate between the structures of the solvated proton ($\Phi_{H3} = \Phi_{Hc} = 1$) and the solvent ($\Phi_{H3} = \lambda$, λ was set to 0.7 for hydrogen bonds to the oxygen of high basicity[125]). If the parameter α is defined as the degree to which the water-like portion of the transition state resembles the solvent, the fractionation factor for proton 3 (Φ_{H3}) in the transition state will be $\lambda^{1-\alpha}$.[128]

Then, the kinetic isotope effects on this reaction can be written:

$$\frac{k_n}{k_H} = \frac{(1-n+n\phi_{H1})(1-n+n\phi_{H2})(1-n+n\phi_{H3})(1-n+n\phi_{Hb})^2(1-n+n\phi_{Hc})^2}{(1-n+n\phi_{Ha})(1-n+n\phi_{Hb})^3(1-n+n\phi_{Hc})^3}$$

$$= \frac{(1-n+\phi n)(1-n+1.25n)\left(1-n+0.7^{1-\alpha}\times n\right)}{(1-n+1.25n)(1-n+0.7n)(1-n+1n)} \quad (59)$$

When n = 1, this equation reduces to:

$$\left(\frac{k_{\mathrm{n}}}{k_{\mathrm{H}}}\right)_{n=1}=\frac{k_{\mathrm{D}}}{k_{\mathrm{H}}}=\frac{\phi\times 1.25\times 0.7^{1-\alpha}}{1.25\times 0.7} \qquad (60)$$

$$\phi=\frac{k_{\mathrm{D}}}{k_{\mathrm{H}}}\times 0.7^{\alpha} \qquad (61)$$

Thus:

$$\frac{k_{\mathrm{n}}}{k_{\mathrm{H}}}=\frac{\left(1-n+\frac{k_{\mathrm{D}}}{k_{\mathrm{H}}}\times 0.7^{\alpha}\times n\right)\times\left(1-n+0.7^{1-\alpha}\times n\right)}{(1-n+0.7n)} \qquad (62)$$

Supplementary Fig. 126 shows that with $\frac{k_{\mathrm{D}}}{k_{\mathrm{H}}}=2$, the line shape is not sensitive to changes when α is between 0 and 1. The semi-linear relationship exhibits almost no difference from the classical one-site proton transfer when considering the possible errors in experiments and the data fitting section.[124, 125]

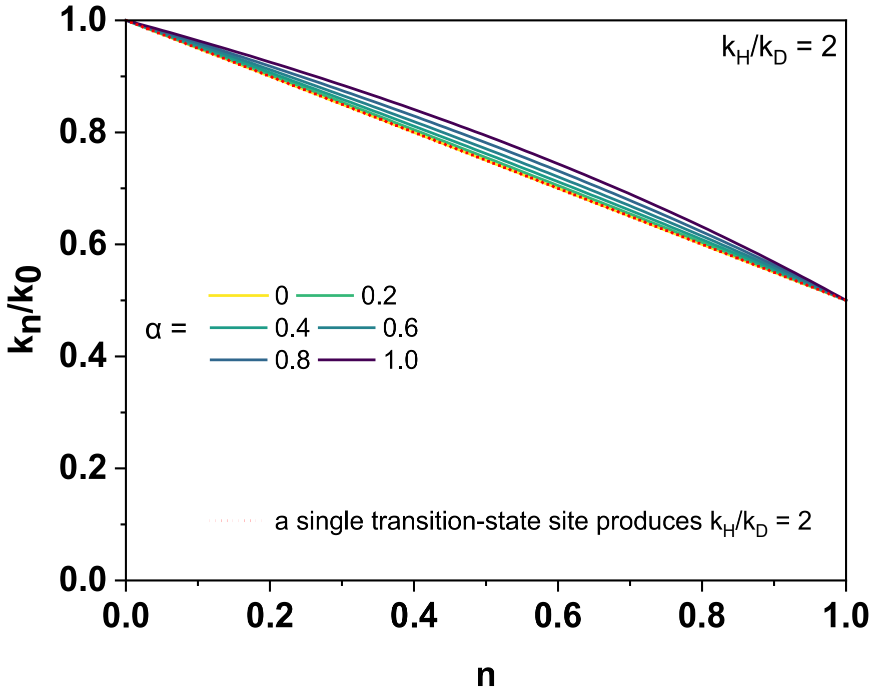

Supplementary Fig. 126 Solutions of *Supplementary Eqn. 62*. The changes in α have a minimal impact on the relationship between k_{n}/k_{0} and n for representative values of $k_{\mathrm{H}}/k_{\mathrm{D}}=2$.

5.3 Curves of "MOOH" step

Similarly, the proton transfer reaction between an additional proton relay (R) and a MO–OH fragment can be treated in a similar way:

$$\mathrm{MO{-}OH} + \mathrm{R} \xrightarrow[\mathrm{TS}]{\mathrm{k}} \mathrm{MO{-}OH{-}{-}{-}R} \longrightarrow \mathrm{MO{-}O} + \mathrm{RH} \qquad (63)$$

The proton transfer process of the transition state is the rate-determining step; the equilibrium between the reactants and transition state may be written as:

Supplementary Notes 5

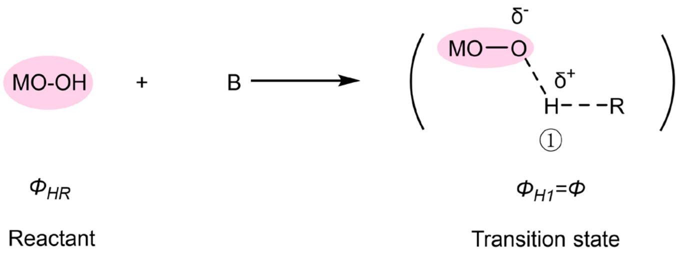

(64)

Where the fractionation factor of the transit proton was defined as Φ ($\Phi_{H1} = \Phi$). The fractionation factor of the reactant MO–OH was set to Φ_{HR}. The value of Φ_{HR} was determined by bonding "strength" between the negative charged MO–O^{-} and the proton; the "tighter" the bonding, the higher the fractionation factor. As shown in ***Supplementary Fig. 127***, with an additional positive charge, the oxonium has an average fractionation factor of 0.69 when compared to the bulk water (Φ = 1); in contrast, the covalently bonded proton in the negatively charged hydroxide ion shows a fractionation factor of 1.25 due to the strong bonding strength compared to other hydrogen bonds (0.70). When considering the case of MO–OH_2 belonging to peroxo ($MOOH_2$), the analog water oxide structure shows a medium-charged dipole between that of bulk water and oxonium, and thus the proposed $0.7 < \Phi_{H3} = 0.7^{1-\alpha} < 1$ for MO–OH_2 in ***Supplementary Eqn. 59*** is reasonable. A tighter bond corresponds to a higher fractionation factor. Consequently, the fractionation factor is found to correlate closely with the atomic charge and dipole moment of the transition state intermediates (***Supplementary Fig. 128***). Regarding the $MOOH^{-}$ with peroxo (OO^{2-}) or superoxo (OO^{-})-like structure, the strong bonding between negatively charged MOO^{-} and proton is anticipated, which could lead to a higher fractionation factor ($\Phi > 1$).

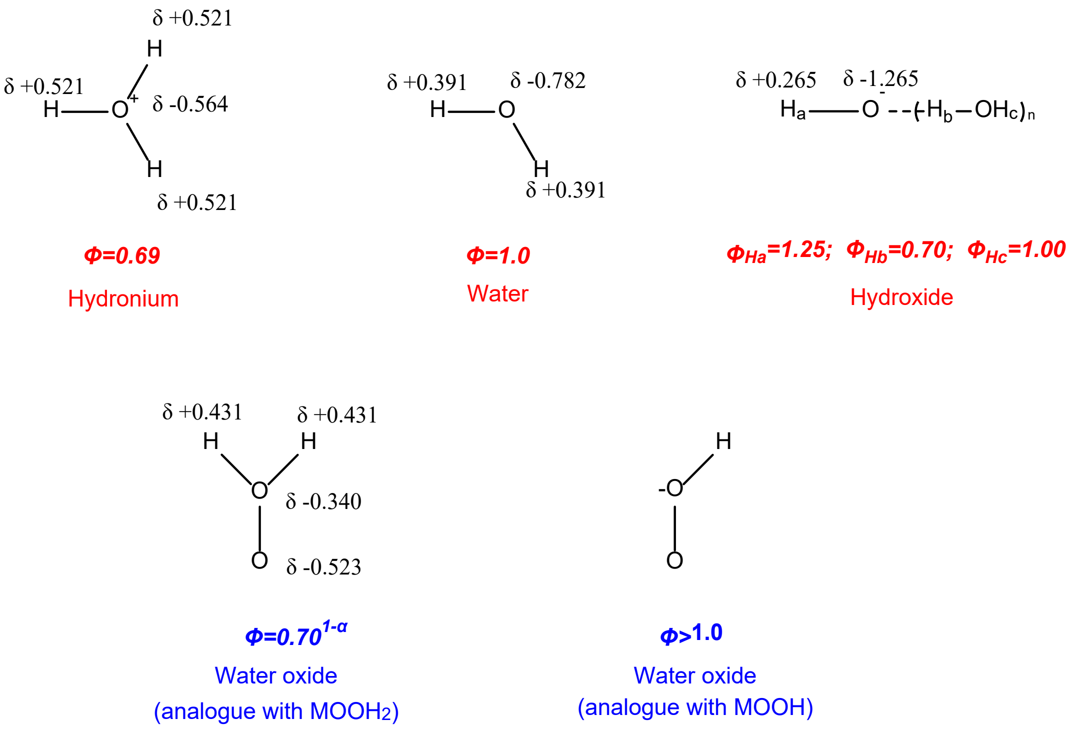

Supplementary Fig. 127 Atomic charges and fractionation factor of reactants.[127] (Charge data source from https://www.colby.edu/chemistry/webmo).

Supplementary Notes 5

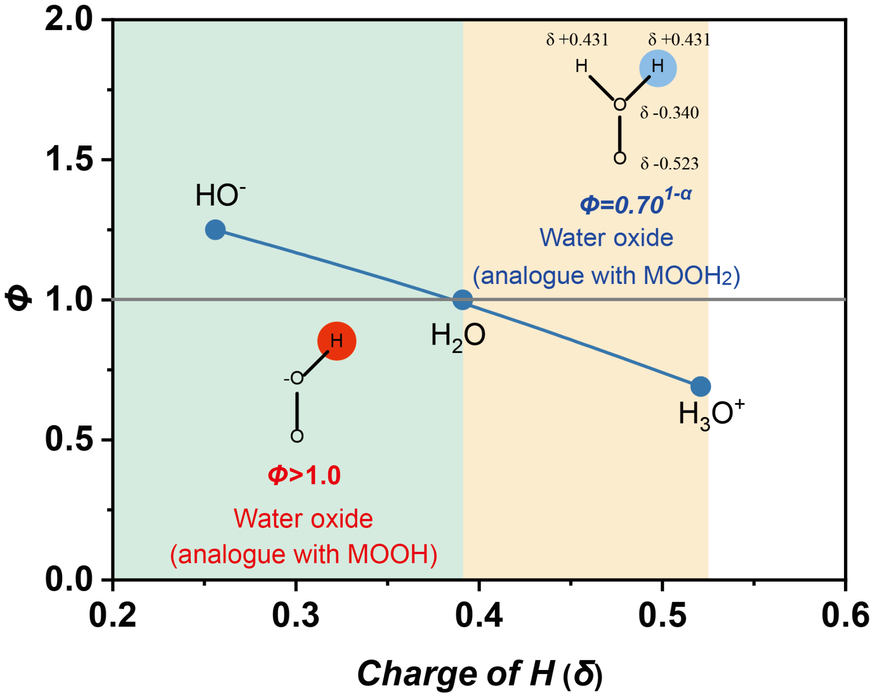

Supplementary Fig. 128 Atomic charges and fractionation factor of reactants.

Then, the kinetic isotope effects on this reaction can be written:

$$\frac{k_n}{k_H} = \frac{(1 - n + n\phi)}{(1 - n + n\phi_{HR})} \quad (65)$$

When n = 1, this equation reduces to:

$$\left(\frac{k_n}{k_H}\right)_{n=1} = \frac{k_D}{k_H} = \frac{\phi}{\phi_{HR}} \quad (66)$$

$$\phi = \frac{k_D}{k_H} \times \phi_{HR} \quad (67)$$

Thus:

$$\frac{k_n}{k_H} = \frac{\left(1 - n + n \times \frac{k_D}{k_H} \times \phi_{HR}\right)}{(1 - n + n \times \phi_{HR})} \quad (68)$$

Supplementary Fig. 129 shows that with $\frac{k_H}{k_D} = 2$, the line shape is sensitive to changes of Φ_{HR}. When $\Phi_{HR} < 1$, a dome-shaped curve could be obtained; when $\Phi_{HR} > 1$, a bowl-shaped curve could be obtained. Since the reactant contains a proton that is not directly from the solvent, the proton inventory curve is controlled by the reactant state as well as the transition state; the competition between two states determines the shape of the curve: if the reactant state is dominant, a bowl-shaped curve is observed; if the transition state is dominant, a dome-shaped curve is observed.

Supplementary Notes 5

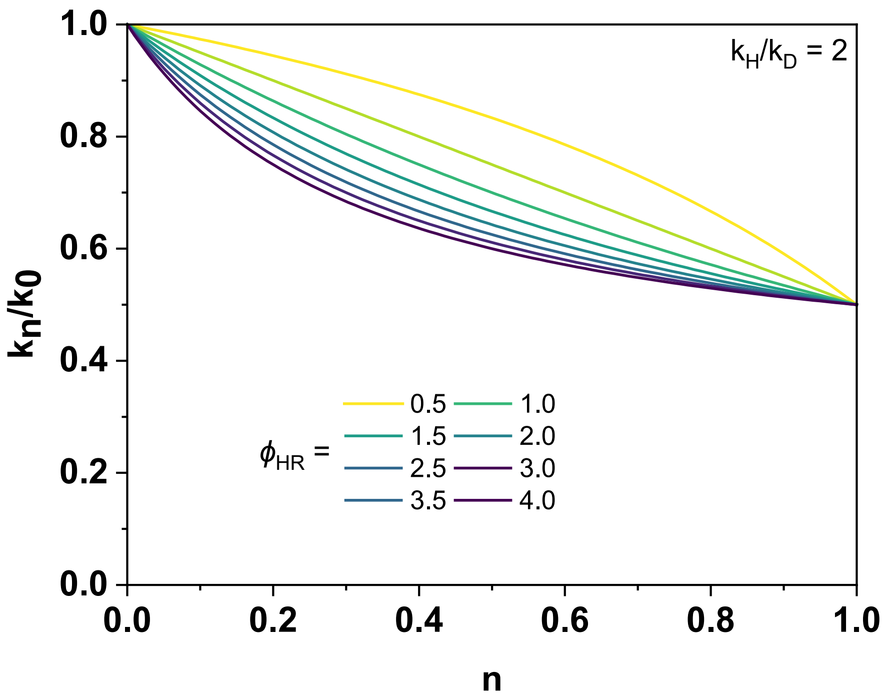

Supplementary Fig. 129 Solutions of *Supplementary Eqn. 68*. The changes in Φ_{HR} have a significant impact on the relationship between k_n/k_0 and n for representative values of $k_H/k_D = 2$.

If the proton acceptor contains other proton(s) in the reactant state, for example, the proton acceptor is the solvent. A similar treatment could be done as follows. For a reaction between an alkaline solvent (OH^-) and a MO–OH fragment followed by a pre-equilibrium proton transfer to the solvent, the equilibrium between reactants and transition state may be written as:

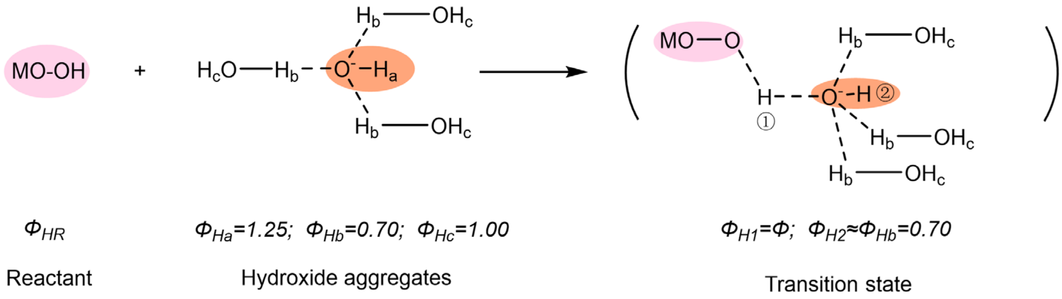

(69)

The kinetic isotope effects on this reaction can be written:

$$\frac{k_n}{k_H} = \frac{(1-n+n\phi_{H1})(1-n+n\phi_{H2})(1-n+n\phi_{Hb})^3(1-n+n\phi_{Hc})^3}{(1-n+n\phi_{HR})(1-n+n\phi_{Ha})(1-n+n\phi_{Hb})^3(1-n+n\phi_{Hc})^3} \quad (70)$$

Since we hypothesize that the fractionation factor of proton 2 remains unchanged ($\Phi_{H2} = \Phi_{Ha} = 1.25$) during the transition state, the protons from the solvent do not actually contribute to isotopic effects. Thus:

$$\frac{k_n}{k_H} = \frac{\left(1-n+n\times\frac{k_D}{k_H}\times\phi_{HR}\right)}{(1-n+n\times\phi_{HR})} \quad (71)$$

The results are the same as in the case of the internal proton acceptor (R).

Supplementary Notes 5

5.4 Curves of successive deprotonation steps

Under the virtual transition state assumption, the proton inventory curvature can be modeled using a more complex scenario in which the M–OOH_2 species is converted to MOO through a two-step reaction process.

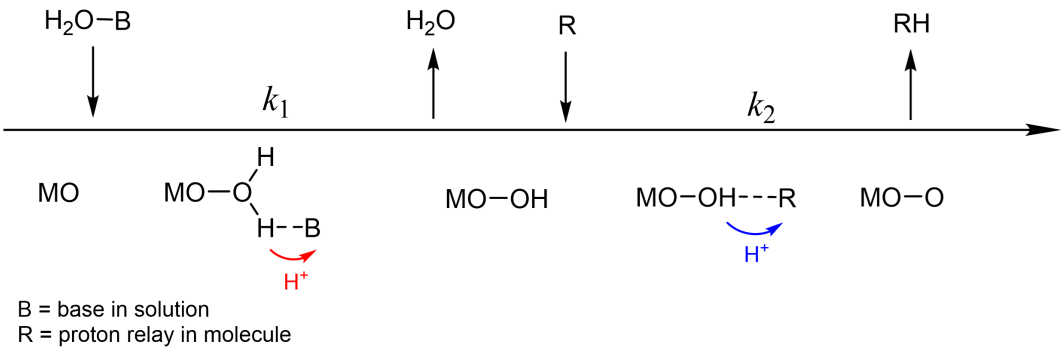

(72)

It is assumed that both the k_1 and k_2 steps significantly contribute to isotope effects, and only the Φ-site (excluding any Z-sites) participates in each of these steps. Analogous to the forward direction in the Ping Pong mechanism, the reaction rates for the forward steps, denoted as k, are written as:[127]

$$k = \frac{k_1 k_2}{k_1 + k_2} \qquad (73)$$

Then, the expected form of k_n can be deduced by writing the expressions according to ***Supplementary Eqn. 73*** for each of the rate constants and then adding their reciprocals.

$$\left(\frac{k_n}{k_H}\right)^{-1} = w_1\left(\frac{k_{1n}}{k_{1H}}\right)^{-1} + w_2\left(\frac{k_{2n}}{k_{2H}}\right)^{-1} \qquad (74)$$

Note that the w terms are weighting factors.

$$w_1 = \left(\frac{k}{k_1}\right) \qquad (75)$$

$$w_2 = \left(\frac{k}{k_2}\right) \qquad (76)$$

With

$$\frac{k_{1n}}{k_{1H}} = \frac{\left(1 - n + \frac{k_D}{k_H} \times 0.7^{\alpha} \times n\right) \times \left(1 - n + 0.7^{1-\alpha} \times n\right)}{(1 - n + 0.7n)} \qquad (77)$$

$$\frac{k_{2n}}{k_{2H}} = \frac{\left(1 - n + n \times \frac{k_D}{k_H} \times \phi_{HR}\right)}{(1 - n + n \times \phi_{HR})} \qquad (78)$$

Thereby:

$$\frac{k_n}{k_H} = \frac{1}{w_1\left[\frac{(1 - n + 0.7n)}{\left(1 - n + \frac{k_D}{k_H} \times 0.7^{\alpha} \times n\right) \times (1 - n + 0.7^{1-\alpha} \times n)}\right] + (1 - w_1)\left[\frac{(1 - n + n \times \phi_{HR})}{\left(1 - n + n \times \frac{k_D}{k_H} \times \phi_{HR}\right)}\right]} \qquad (79)$$

The curve simulation according to ***Supplementary Eqn. 79*** was plotted in ***Supplementary Fig. 130***. When $\frac{k_{1D}}{k_{1H}} \leq \frac{k_{2D}}{k_{2H}}$, the curvature of plots increased with the decrease of the weighting factor w_1. A good match between the simulated data and the experimental data is possible with a relatively small value of w_1. When $\frac{k_{1D}}{k_{1H}} > \frac{k_{2D}}{k_{2H}}$, unique *S*-shape curves are obviously inconsistent with the experimental

data. It would be worth mentioning that the actual weights change with the deuteration of the solvent, and this may introduce a detectable change in the apparent isotope effects that is not reflected in ***Supplementary Eqn. 79*** since the weighting factors were set to be constant. In some cases, if the isotopic effects (or proton inventory plots) of the two transition states are not very different from one of the single states, additional evidence should be provided to distinguish the "one step" model or the "two steps in succession" model. It is important to mention that the proton inventory model employed in this study is still based on a simplified ideal interfacial reaction model. Given that the structural characteristics of heterogeneous electrochemical interfaces remain an active area of research, future investigations should focus on refining proton inventory models by incorporating emerging insights into interfacial structures in heterogeneous electrocatalysis.

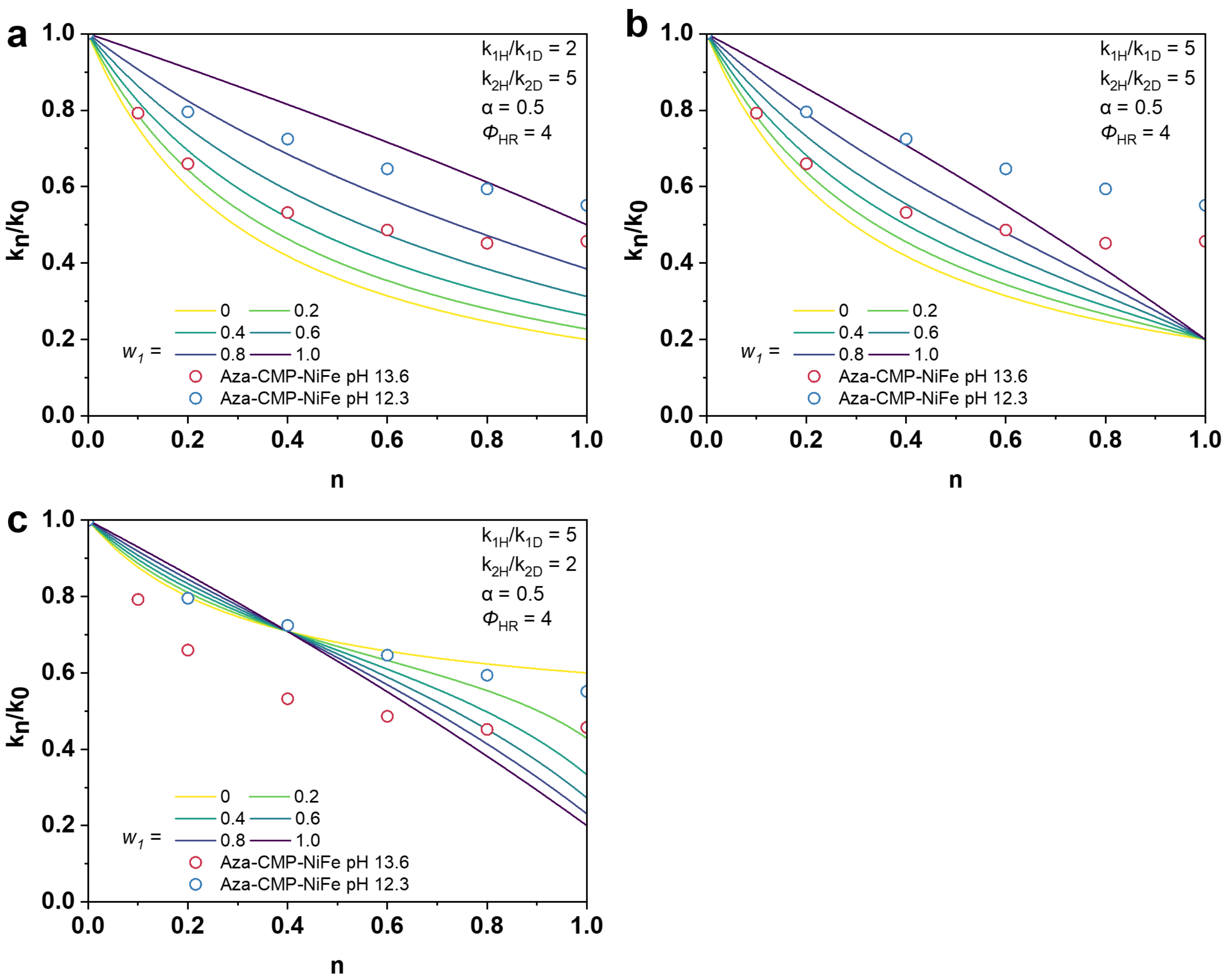

Supplementary Fig. 130 Solutions of *Supplementary Eqn. 79* show the response when changing the weighting factor and kinetic isotopic value of each step. (a) $\frac{k_{1D}}{k_{1H}} < \frac{k_{2D}}{k_{2H}}$. (b) $\frac{k_{1D}}{k_{1H}} = \frac{k_{2D}}{k_{2H}}$. (c) $\frac{k_{1D}}{k_{1H}} > \frac{k_{2D}}{k_{2H}}$.

Supplementary Notes 5

5.5 Evaluating the potential impact of equilibrium isotope effects

The observed KIE from H_2O/D_2O exchange can be primarily attributed to the direct involvement of proton transfer in the catalytic RDS. However, shifts in the redox potential within the pre-catalytic region may also induce a similar KIE effect.[130] Specifically, shifts in the oxidation potential for the pre-oxidation process before OER catalysis, resulting from differences in the binding energies of protons and deuterons (equilibrium isotope effect, EIE), can lead to an apparent anodic shift in the observed catalytic current during KIE testing. This phenomenon is fundamentally driven by variations in the coverage of high-valent species (e.g., Co^{4+} in CoO_x) in H_2O versus D_2O environments, which in turn alter the apparent catalytic current. From a thermodynamic perspective, the extent to which the EIE affects KIE measurements should depend on the potential difference between the pre-oxidation potential and the OER onset potential (i.e., the potential at which the RDS oxidation occurs). If the pre-oxidation potential is close to the OER onset potential, a shift in the pre-oxidation potential (caused by EIE) can significantly alter the coverage of high-valent species, which in turn affects the OER catalytic current. This has been observed by Pasquini et al. for Co-based catalysts.[130] However, if the pre-oxidation potential is far from the OER onset potential, the high-valent species will already reach near-saturation coverage at the OER onset, meaning that a shift in the pre-oxidation potential has minimal impact on the OER catalytic current.

To better understand the extent of the EIE effect on KIE measurements, we can further examine this phenomenon within a quantitative thermodynamic framework. In ***Supplementary Notes 4.3***, we discuss the influence of the pre-oxidation process preceding the RDS on the pH dependence of OER activity. This theoretical model is also applicable to evaluate how the redox potential variations in the pre-oxidation process influence the OER onset and the discussion of EIE in KIE measurements. We assume a model system that the pre-RDS oxidation $M^{n+} - e^- \rightarrow M^{(n+1)+}$ is an irreversible $1e^-$ transfer process, and the RDS oxidation $M^{(n+1)+} - e^- \rightarrow M^{(n+2)+}$ is a single electron transfer process capable of driving OER. The ideal LSV for this system is presented in ***Supplementary Fig. 131a***, simulated using ***Supplementary Eqns. 40*** and ***41*** with the following parameters: $k_{ap} = 10\ s^{-1}$, $v = 0.01\ V\ s^{-1}$, $\Gamma = 5\times10^{-8}$ mol, α = 0.5, $E_p = 1.3$ V vs. RHE, and $E_r = 1.60$ V vs. RHE. When the oxidation potential of $M^{(n+1)+}$ (E_r) is fixed at 1.6 V while varying the oxidation potential of M^{n+} (E_p), the corresponding LSV responses are shown in ***Supplementary Fig. 131b***. The potential at different OER current levels was extracted to evaluate the correlation between E_p and OER activity (***Supplementary Fig. 131c***). The analysis reveals that when $E_{pre\text{-}RDS} < 1.45$ V, the OER catalytic current remains largely unaffected by changes in $E_{pre\text{-}RDS}$. However, when $E_{pre\text{-}RDS} > 1.45$ V, a noticeable anodic shift in the OER catalytic current emerges. This effect is further supported by the coverage profiles of key catalytic species (***Supplementary Fig. 131d***): The observed decrease in OER catalytic current is attributed to the reduced coverage of the RDS-active species, which, in turn, stems from a decrease in the coverage of the pre-RDS species. Therefore, the simulated framework supports the conclusion that when the pre-oxidation potential is sufficiently close to the OER onset potential, an EIE-induced anodic shift in the pre-oxidation potential can indeed cause a reduction in OER catalytic current.

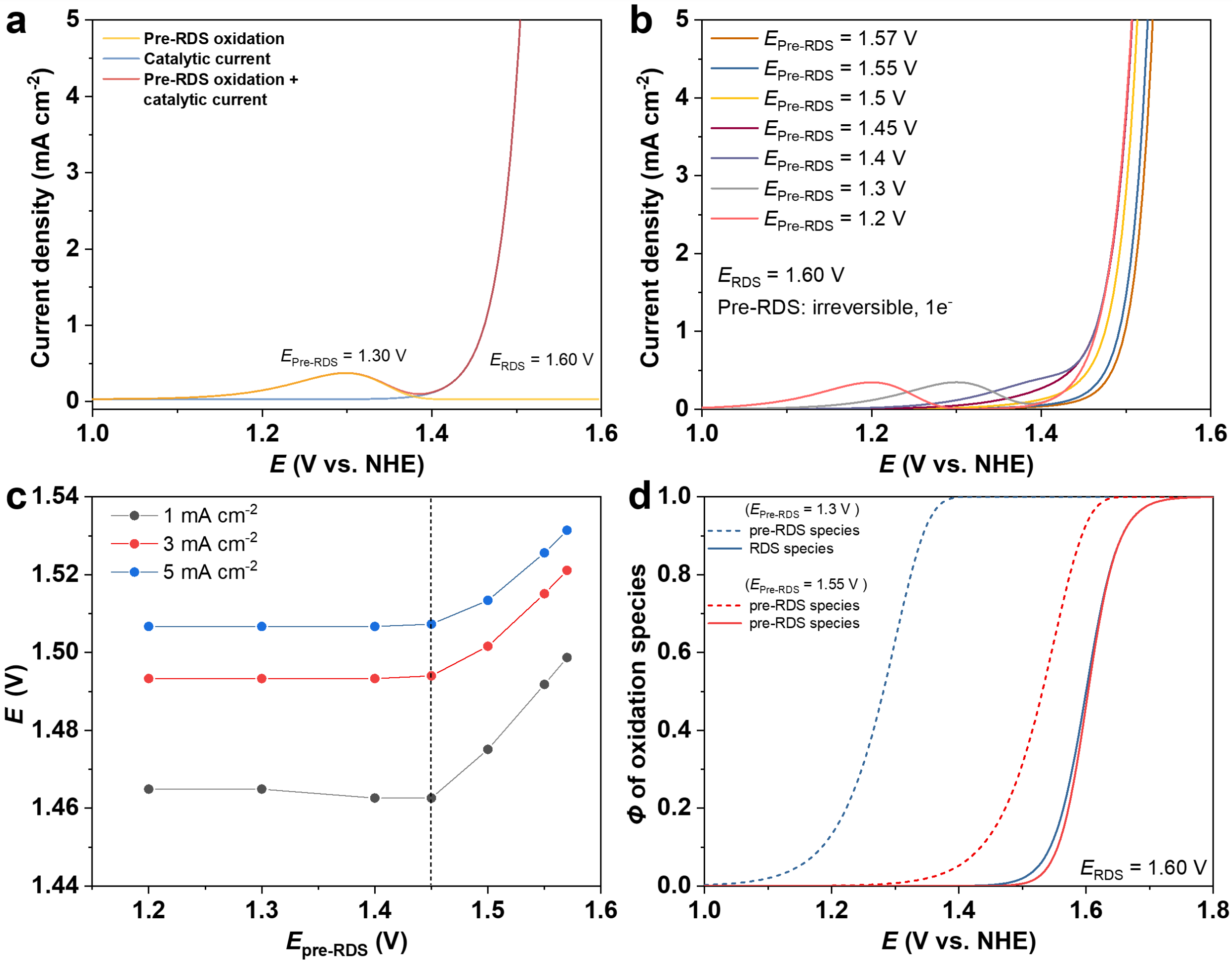

Supplementary Fig. 131 Simulated current-potential behavior using *Supplementary Eqns. 40* and *41.* (a) Simulated LSV curve of catalytic events that have a $1e^-$ RDS (E_r = 1.6 V) and $1e^-$ irreversible pre-RDS (E_p = 1.3 V) oxidation (scan rate: 10 mV s^{-1}). (b) Simulated LSV curves of catalytic events with varying E_p. (c) The relationship between catalytic current and E_p. (d) Relationship between surface species coverage and potential.

Furthermore, two additional scenarios were examined: (1) pre-RDS oxidation is a single-electron reversible oxidation process, and (2) pre-RDS oxidation is a two-electron reversible oxidation process. As illustrated in ***Supplementary Fig. 132***, a similar trend is observed—EIE effects only lead to apparent KIE effects when the pre-oxidation potential is sufficiently close to the OER onset potential. Specifically, for a single-electron irreversible process, a single-electron reversible process, and a two-electron reversible process, the threshold potential difference between the pre-RDS and RDS oxidation steps for the EIE effect to become significant is approximately 0.15 V, 0.15 V, and 0.1 V, respectively. Based on these findings, the following conclusion can be generalized: As long as the oxidation peak of the pre-oxidation process is clearly distinguishable in experimental CV curves, the impact of the EIE effect on KIE measurements can be considered negligible. This observation is consistent with the findings of Pasquini et al., who reported that in their system, the Co^{3+} to Co^{4+} oxidation process did not exhibit a distinct/separate oxidation peak (possibly overwhelmed by the OER current).[130] Consequently, the EIE effect strongly influenced the observed KIE phenomenon in their study.

Supplementary Notes 5

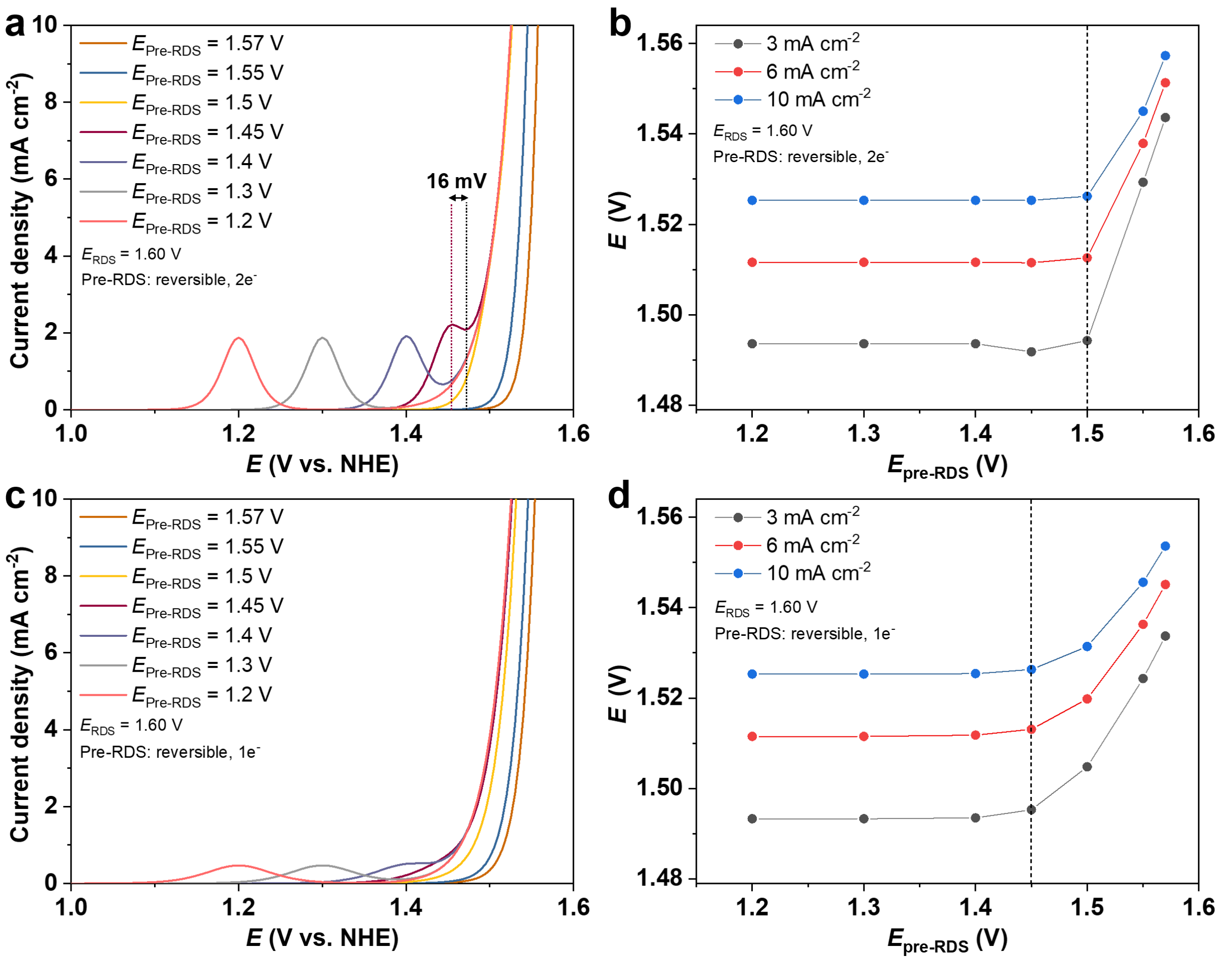

Supplementary Fig. 132 Simulated current-potential behavior. Simulated LSV curves of catalytic events that have (a) 2e$^-$ reversible pre-RDS and (c) 1e$^-$ reversible pre-RDS oxidation; E_r was fixed to 1.6 V (scan rate: 10 mV s^{-1}). Corresponding relationship between catalytic current and E_p: (b) 2e$^-$ reversible pre-RDS and (d) 1e$^-$ reversible pre-RDS.

In the Aza-CMP-NiFe system, ***Supplementary Fig. 133a*** presents the CV curves of Aza-CMP-NiFe in NaOH and NaOD, plotted at an equivalent driving force (overpotential). The $Ni^{2+/3+}$ oxidation peak exhibits a slight cathodic shift in NaOD, indicating that shifted redox potentials in the pre-catalytic potential regime are unlikely to contribute to the observed KIE phenomenon. Similar cathodic shifts of the $Ni^{2+/3+}$ oxidation peak in NaOD have also been reported in other NiFe systems.[12, 99] At near-neutral pH, the CV curve of Aza-CMP-NiFe in NaBi/D_2O shows only a minor anodic shift and separates from the OER catalytic current (***Supplementary Fig. 133b***), suggesting that EIE effects from the pre-catalytic oxidation process are not a major factor in the observed KIE. Additionally, across a broad pH range (pH 9-14), the $Ni^{2+/3+}$ oxidation peak remains clearly distinguishable and well-separated from the OER catalytic current (***Supplementary Fig. 74***), minimizing the potential impact of EIE on KIE of OER current. Based on these observations, we conclude that EIE effects induced by deuterium substitution do not significantly influence the KIE measurements or proton inventory analysis in this study.

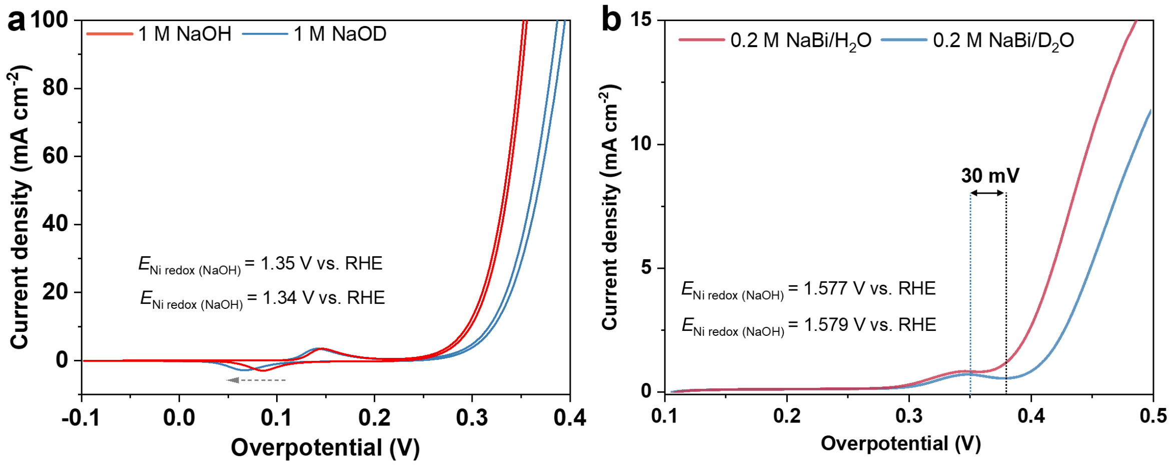

Supplementary Fig. 133 KIE measurements of Aza-CMP-NiFe. CV curves of Aza-CMP-NiFe in 1.0 M Fe-free NaOD/D_2O and NaOH/H_2O solutions (scan rate: 20 mV s^{-1}). (b) LSV curves of Aza-CMP-NiFe in 0.2 M NaBi/D_2O and NaBi/H_2O solutions (scan rate: 10 mV s^{-1}).

Supplementary Notes 6

Supplementary Notes 6: DFT calculations

6.1 Testing of DFT functionals

The selection of the M06-L functional is rationalized through testing the pK_a and oxidation potential values involving multiple functionals including GGA (PBE-D3, BLYP-D3), meta-GGA (M06-L), hybrid-GGA (PBE0-D3, B3LYP-D3, wB97X-D), and meta-hybrid-GGA (M06, M06-2X, wB97M-V) exchange-correlation functionals. The calculated pK_a values and redox potentials using these functionals are compared with experimental values (***Supplementary Table 9***).

Supplementary Table 9 Oxidation potentials of Ni^{2+}/Ni^{3+} and Ni^{3+}/Ni^{4+} (eV) at pH of 14.0 and pK_a of Ni^{2+} and Ni^{3+} species were calculated using different functionals and compared with experimental values. Among all the functionals, the M06-L functional provides the closest values to the experimental data, with 0.60 eV for Ni^{2+}/Ni^{3+} redox potential, 0.82 for Ni^{3+}/Ni^{4+} couples at pH of 14.0, 12.8 for the pK_a of $[Ni^{2+}(OH)(H_2O)_3]^+_T$, and 11.2 for the p$K_a$ of $[Ni^{3+}(OH)_2(H_2O)_2]^+_D$.

	$E([Ni^{2+}(OH)_2(H_2O)_2]_T$ $/[Ni^{3+}(OH)_3(H_2O)]_Q)$	$E([Ni^{3+}(OH)_3(H_2O)]_Q/[$ $Ni^{4+}{=}O(OH)_2(H_2O)]_T)$	pK_a $[Ni^{2+}(H_2O)_4]^{2+}_T$	pK_a $[Ni^{2+}(OH)(H_2O)_3]^+_T$	pK_a $[Ni^{3+}(OH)_2(H_2O)_2]^+_D$
BLYP-D3	0.07	0.98	5.2	10.8	−1.3
PBE-D3	0.08	1.03	3.5	9.8	−0.3
PBE0-D3	0.79	1.08	8.0	14.3	8.9
M06	0.59	1.34	6.8	13.0	3.8
B3LYP-D3	0.69	1.37	8.2	11.9	5.6
**M06-L**	**0.60**	**0.82**	**8.0**	**12.8**	**11.2**
wB97X-D	1.62	0.84	8.6	9.6	11.9
M06-2X	1.87	1.28	9.3	10.7	8.3
wB97M-V	1.46	0.57	4.7	6.6	9.8
**Exp. (vs RHE)**	**0.5**	**0.8**		**12.5**	**11.5**

The selection of the M06-L functional is further validated by calculating the electronic energy difference (kcal mol^{-1}) between low- and high-spin states of Ni^{2+} and Ni^{3+} species using multiple functionals, including GGA (PBE-D3, BLYP-D3), meta-GGA (M06-L), hybrid-GGA (PBE0-D3, B3LYP-D3), and meta-hybrid-GGA (M06) exchange-correlation functionals. Despite the diverse values of the electronic energy difference, the stable state of the Ni^{2+} and Ni^{3+} species remains the same regardless of the DFT functionals used (***Supplementary Table 10***).

Supplementary Notes 6

Supplementary Table 10 The electronic energy difference (kcal mol^{-1}) between low- and high-spin states of Ni^{2+} and Ni^{3+} species. The electronic energy difference is defined as *E*(species) = *E*(low-spin state) − *E*(high-spin state), where singlet and triplet are for Ni^{2+} site, and doublet and quartet are for Ni^{3+} site. Multiconfigurational pair density functional theory was used as the reference for the estimation of the energies of the Ni spin states. We used the translated tB3LYP functional,[131] which gives good agreement with experiments for metal atoms.[132] The def2-SVP basis set was used and the active space was selected by including the 12 (Ni^{2+}) or 11 (Ni^{3+}) highest energy electrons in orbitals with significant nickel contribution. We also included the lowest unoccupied orbitals to a final CAS space of (12,8) for Ni^{2+} and (11,8) for Ni^{3+}.

	E $[Ni^{2+}(H_2O)_4]^{2+}$	*E* $[Ni^{2+}(OH)(H_2O)_3]^{+}$	*E* $[Ni^{3+}(OH)_2(H_2O)_2]^{+}$
PBE-D3	4.8	0.1	−13.5
PBE0-D3	25.2	19.8	−0.3
M06-L	18.9	19.6	−7.7
M06	15.8	9.0	−0.1
BLYP-D3	6.6	0.2	−10.7
B3LYP-D3	20.3	13.8	−0.3
MC-PDFT (tB3LYP)	4.3	4.9	−17.3

To determine the structure with the lowest energy, various spin states of the catalytic metal atom and their different configurations are computed using the validated M06-L functional. Subsequently, the most stable structure of a certain oxidation state is applied to calculate the reaction pathways.

Regarding spin contamination issues, a restraint was added to singlet state structures to address spin contamination. From our calculations, the S^2 value is 0 for both $[Ni^{2+}(H_2O)_4]^{2+}$ and $[Ni^{2+}(OH)(H_2O)_3]^{+}$ at the singlet state. For the triplet states, $[Ni^{2+}(H_2O)_4]^{2+}$ has an S^2 value of 2.004 and $[Ni^{2+}(OH)(H_2O)_3]^{+}$ has 2.005 (***Supplementary Table 11***). The S^2 value for $[Ni^{3+}(OH)_2(H_2O)_2]^{+}$ at the doublet state is 0.851 and for the quartet state it is 3.762. All calculated S^2 values are in close alignment with the theoretical values, with the doublet state of the $[Ni^{3+}(OH)_2(H_2O)_2]^{+}$ species being a slight deviation from the expected value.

Supplementary Table 11 The calculated S^2 value for each species and the expected values are given in parentheses.

	Low	High
$[Ni^{2+}(H_2O)_4]^{2+}$	0 (0)	2.004 (2)
$[Ni^{2+}(OH)(H_2O)_3]^{+}$	0 (0)	2.005 (2)
$[Ni^{3+}(OH)_2(H_2O)_2]^{+}$	0.851 (0.75)	3.762 (3.75)

6.2 Calculations on Aza-CMP-Ni

Ni^{2+} species

At the $[Ni^{2+}(OH_2)_4]^{2+}$ state, the optimized structure (***Supplementary Fig. 134a***) in the triplet state is energetically favored, being 18.9 kcal mol^{-1} lower in energy compared to its singlet state (the energy difference of varying spin states is detailed in ***Supplementary Table 12***). For $[Ni^{2+}(OH)(OH_2)_3]^{+}$, the most stable configuration is the triplet state with the –OH group in the axial position (***Supplementary Fig. 134b***). This configuration is 19.6 kcal mol^{-1} lower in energy compared to its singlet state, and 6.9 kcal mol^{-1} and 0.7 kcal mol^{-1} lower in energy than the singlet and triplet state of the configuration with

the equatorial –OH group, respectively (the energy difference of varying configuration states is detailed in ***Supplementary Table 13***). Using the optimized structures of $[Ni^{2+}(OH_2)_4]^{2+}{}_T$ and $[Ni^{2+}(OH)(OH_2)_3]^{+}{}_T$, the calculated p$K_a$ for the $[Ni^{2+}(OH_2)_4]^{2+}{}_T/[Ni^{2+}(OH)(OH_2)_3]^{+}{}_T$ pair is 8.0. Therefore, $[Ni^{2+}(OH)(OH_2)_3]^{+}{}_T$ could be further deprotonated to form $[Ni^{2+}(OH)_2(OH_2)_2]$ with further increasing pH. For $[Ni^{2+}(OH)_2(OH_2)_2]$, the most stable configuration is the triplet state with one –OH group in the axial position and one in the equatorial position (***Supplementary Fig. 134c***). This configuration is 15.7 kcal mol^{-1} lower compared to its singlet state, and 17.0 kcal mol^{-1} and 1.0 kcal mol^{-1} lower than the singlet and triplet state of the configuration with two axial –OH groups, respectively. The calculated pK_a for the $[Ni^{2+}(OH)(OH_2)_3]^{+}{}_T/[Ni^{2+}(OH)_2(OH_2)_2]_T$ pair is 12.8, which aligns well with the experimental value of 12.5 obtained from the Pourbaix diagram. Hence, $[Ni^{2+}(OH)_2(OH_2)_2]_T$ with one –OH in the axial position and one in the equatorial position is identified as the ground state of the Ni^{2+} species at the experimental 14.0 pH, the octahedron Ni–N_2O_4 configuration is also consistent with the Ni moiety observed in EXAFS analysis (***Supplementary Figs. 12*** and ***13***).

Ni^{3+} species

The oxidation of $[Ni^{2+}(OH)_2(OH_2)_2]_T$ could form $[Ni^{3+}(OH)_2(OH_2)_2]^{+}$ via one-electron oxidation or produce $[Ni^{3+}(OH)_3(OH_2)]$ via electron-coupled proton transfer. For $[Ni^{3+}(OH)_2(OH_2)_2]^{+}$, the doublet structure with one –OH group in the equatorial and another –OH group in the axial position is 7.7 kcal mol^{-1} lower in energy compared to its quartet state. It should be noted that this configuration is 0.7 kcal mol^{-1} higher and 7.5 kcal mol^{-1} lower than the structure of the doublet and quartet state with two –OH groups in the axial position, respectively, although these configurations cannot be formed through the oxidation of $[Ni^{2+}(OH)_2(OH_2)_2]_T$. Therefore, the lower spin state configuration $[Ni^{3+}(OH)_2(OH_2)_2]^{+}{}_D$ (***Supplementary Fig. 134d***) is the stable structure for $[Ni^{3+}(OH)_2(OH_2)_2]^{+}$. The ground state for $[Ni^{3+}(OH)_3(OH_2)]$ (***Supplementary Fig. 134e***) is the quartet with the H_2O molecule coordinated in the equatorial position. This configuration is 6.9 kcal mol^{-1} and 9.0 kcal mol^{-1} lower than the doublet and quartet states of the structure with the H_2O molecule in the axial position. The calculated pK_a for $[Ni^{3+}(OH)_2(OH_2)_2]^{+}{}_D/[Ni^{3+}(OH)_3(OH_2)]_Q$ pair is 11.2. Hence, at pH 14.0, $[Ni^{3+}(OH)_3(OH_2)]_Q$ with the equatorial H_2O is the stable state of Ni^{3+}, leading to an oxidation potential of 0.61 eV for $[Ni^{2+}(OH)_2(OH_2)_2]^{+}{}_T/Ni^{3+}(OH)_3(OH_2)]_Q$ redox couple.

Ni^{4+} species

The oxidation of $[Ni^{3+}(OH)_3(OH_2)]_Q$ species leads to the formation of $[Ni^{4+}{=}O(OH)_2(OH_2)]$. The ground state of $[Ni^{4+}{=}O(OH)_2(OH_2)]_T$ (***Supplementary Fig. 134f***) is the triplet state with the oxo positioned equatorially. This structure is 14.6 kcal mol^{-1} lower in energy compared to its singlet state, and 0.4 kcal mol^{-1} lower than the structure with the oxo positioned axially. Further deprotonation of $[Ni^{4+}{=}O(OH)_2(OH_2)]_T$ forms $[Ni^{4+}{=}O(OH)_3]$ triplet state, which is 9.9 kcal mol^{-1} lower than its singlet state. However, the calculated pK_a of $[Ni^{4+}{=}O(OH)_2(OH_2)]_T/[Ni^{4+}{=}O(OH)_3]_T$ pair is 20.3. Hence, the $[Ni^{4+}{=}O(OH)_2(OH_2)]_T$ with the equatorial oxo is the stable state of Ni^{4+}, and calculated oxidation potential for the $[Ni^{3+}(OH)_3(OH_2)]_Q/[Ni^{4+}{=}O(OH)_2(OH_2)]_T$ pair is 0.82 eV at pH 14.0. As proved by experiments, the further oxidation of Ni^{4+} to Ni^{5+} is not studied.

In summary, the optimal configuration of Aza-CMP-Ni under the Ni^{2+} state is identified as the $[Ni^{2+}(OH)_2(OH_2)_2]_T$ with one –OH in both the axial and equatorial position at pH 14. This species undergoes oxidation to $[Ni^{3+}(OH)_3(OH_2)]_Q$ with the equatorial H_2O via a $1H^{+}/1e^{-}$ PCET process, exhibiting a calculated potential of 0.61 eV. Subsequent oxidation leads to the formation of $Ni^{4+}{=}O(OH)_2(OH_2)]_T$, with the oxo group equatorially positioned. Hence, $[Ni^{4+}{=}O(OH)_2(OH_2)]_T$ is the

favorable state at the Ni^{4+} and it will be involved in the key O–O bond formation step.

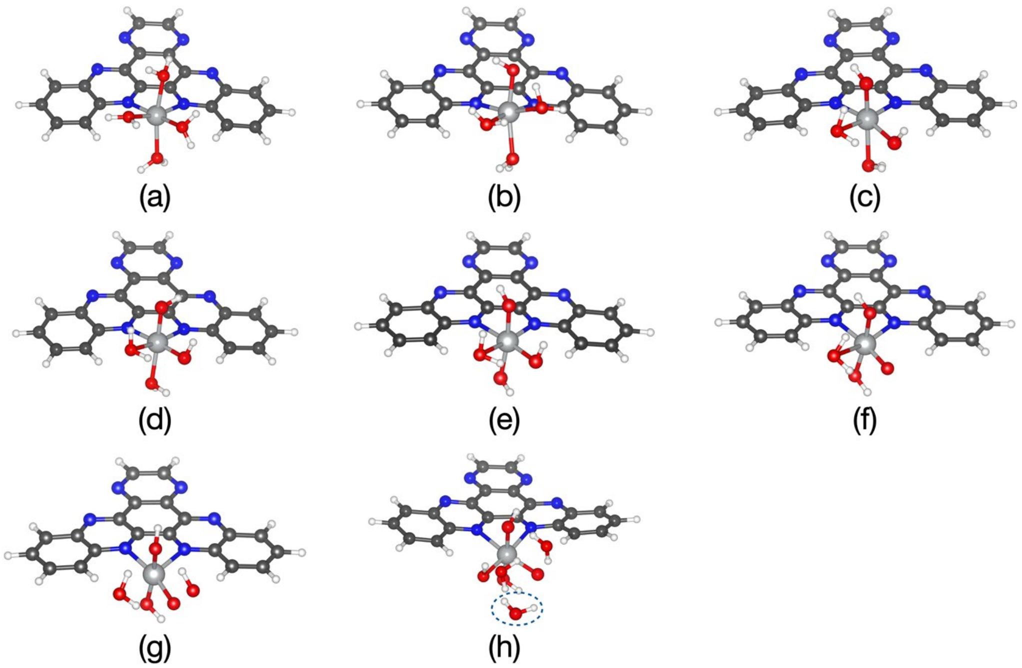

Supplementary Fig. 134 The optimized structure of various species. (a) $[Ni^{2+}(OH_2)_4]^{2+}{}_T$. (b) $[Ni^{2+}(OH)(OH_2)_3]^{+}{}_T$. (c) $[Ni^{2+}(OH)_2(OH_2)_2]_T$. (d) $[Ni^{3+}(OH)_2(OH_2)_2]^{+}{}_D$. (e) $[Ni^{3+}(OH)_3(OH_2)]_Q$. (f) $[Ni^{4+}{=}O(OH)_2(OH_2)]_T$. (g) $[Ni^{4+}{=}O(OH)_2(OH_2)$--$OH]$. (h) Product of OH nucleophilic attack on $[Ni^{4+}{=}O(OH)_2(OH_2)]_T$.

Supplementary Table 12 The energy difference (kcal mol^{-1}) between low- and high-spin states of various species at their optimal configurations was calculated using M06-L functionals. The energy difference is defined as E(species) = E(low-spin state) − E(high-spin state).

E $[Ni^{2+}(H_2O)_4]^{2+}$	18.9
E $[Ni^{2+}(OH)(H_2O)_3]^{+}$	19.6
E $[Ni^{2+}(OH)_2(H_2O)_2]$	15.7
E $[Ni^{3+}(OH)_2(H_2O)_2]^{+}$	−7.7
E $[Ni^{3+}(OH)_3(H_2O)]$	−13.2
E $[Ni^{4+}{=}O(OH)(H_2O)_2]^{+}$	−3.0
E $[Ni^{4+}{=}O(OH)_2(H_2O)]$	14.6

Supplementary Table 13 The free energy difference (kcal mol^{-1}) between different configuration states of various species was calculated using M06-L functionals. The free energy difference is defined as E(species) = E(axial) − E(equatorial), considering only the optimal spin state. Axial represents –OH group positioned axially and equatorial represents –OH group positioned equatorially in $[Ni^{2+}(OH)(H_2O)_3]^{+}$; H_2O group positioned axially and equatorial represents –OH group positioned equatorially in $[Ni^{3+}(OH)_3(H_2O)]$; Axial represents =O group positioned axially and equatorial represents –OH group positioned equatorially in $[Ni^{4+}{=}O(OH)(H_2O)_2]^{+}$, $[Ni^{4+}{=}O(OH)_2(H_2O)]$.

E $[Ni^{2+}(OH)(H_2O)_3]^{+}$	−0.7
E $[Ni^{3+}(OH)_3(H_2O)]$	19.0
E $[Ni^{4+}{=}O(OH)(H_2O)_2]^{+}$	2.8
E $[Ni^{4+}{=}O(OH)_2(H_2O)]$	−0.4

Supplementary Notes 6

O–O bond formation on single Ni sites

The formation of the O–O bond from $[Ni^{4+}{=}O(OH)_2(OH_2)]_T$ is initially computed via the WNA pathway, with an additional H_2O molecule coordinated to the complex. The deprotonation of the coordinated H_2O molecule in $[Ni^{4+}{=}O(OH)_2(OH_2)\text{--}OH_2]_T$ produces $[Ni^{4+}{=}O(OH)_2(OH_2)\text{--}OH]$ species (***Supplementary Fig. 134g***), where the triplet state is found to be energetically favored by -6.8 kcal mol^{-1} compared to its singlet state. The pK_a for $[Ni^{4+}{=}O(OH)_2(OH_2)\text{--}OH_2]_T/[Ni^{4+}{=}O(OH)_2(OH_2)\text{--}OH]_T$ is calculated to be 13.1, in good agreement with the experimental value of 12.5. The reaction is found to be endergonic and requires reaction free energy and activation free energy of 2.3 kcal mol^{-1} and 20.5 kcal mol^{-1}, respectively (***Supplementary Fig. 135***). The proton generated from the WNA process is subsequently transferred to the –OH group coordinated to Ni, forming $[Ni^{2+}\text{–}OOH(OH)(OH_2)_2]_T$. In comparison, the direct OH nucleophilic attack on $[Ni^{4+}{=}O(OH)_2(OH_2)]_T$ will lead to the deprotonation of the coordinated H_2O (***Supplementary Fig. 134h***) rather than attacking the oxo, as the calculated energy of the former is 6.0 kcal mol^{-1} lower in free energy than the latter.

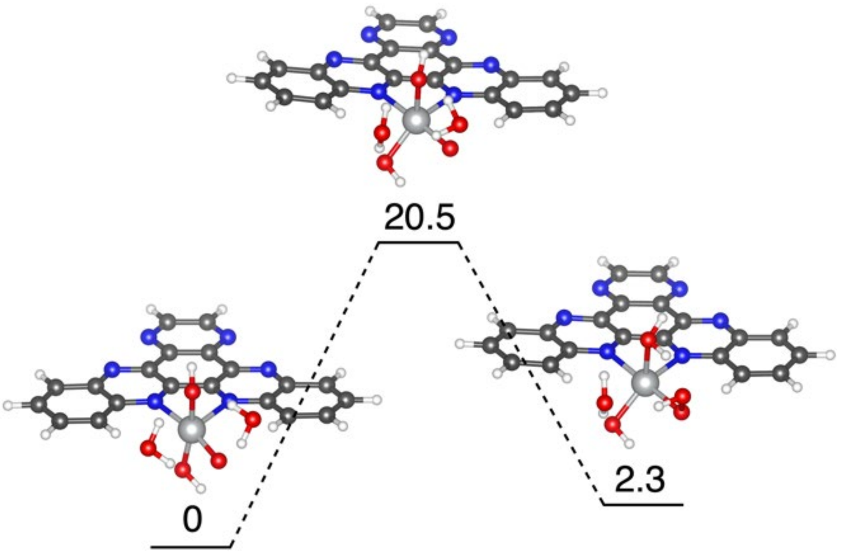

Supplementary Fig. 135 The reaction energy profile of the WNA pathway with optimized structures. The values are given in units of kcal mol^{-1}.

6.3 Calculations on Aza-CMP-NiFe with unprotonated bridging oxygen atoms

The structure of Ni-Fe sites is first optimized using the $Ni^{2+}Fe^{3+}$ state with two oxygen atoms utilized as bridges between Ni^{2+} and Fe^{3+} as the initial oxidation state, using the M06-L functional.

$Ni^{2+}Fe^{3+}$

The $Ni^{2+}Fe^{3+}$ state can manifest as either $[Ni^{2+}(OH_2)_2O_2Fe^{3+}(OH_2)_4]^{1+}$ or $[Ni^{2+}(OH_2)_2O_2Fe^{3+}(OH_2)_3(OH)]$. The quartet state $[Ni^{2+}(OH_2)_2O_2Fe^{3+}(OH_2)_4]^{1+}$ (***Supplementary Fig. 136a***) is 19.1 kcal mol^{-1} lower in energy compared to its doublet state. The optimal structure for $[Ni^{2+}(OH_2)_2O_2Fe^{3+}(OH_2)_3(OH)]$ (***Supplementary Fig. 136b***) is the doublet state with the –OH group axially coordinated to the Fe^{3+} center. This configuration is 12.0 kcal mol^{-1} lower in energy compared to its quartet state, and 6.5 kcal mol^{-1} and 30.4 kcal mol^{-1} lower than the doublet and quartet state with the –OH group equatorially positioned to the Fe^{3+}, respectively. The calculated pK_a for $[Ni^{2+}(OH_2)_2O_2Fe^{3+}(OH_2)_4]^{1+}{}_Q/[Ni^{2+}(OH_2)_2O_2Fe^{3+}(OH_2)_3(OH)]_D$ is 16.4. Hence, $[Ni^{2+}(OH_2)_2O_2Fe^{3+}(OH_2)_4]^{1+}{}_Q$ is the ground state of the $Ni^{2+}Fe^{3+}$ state at the experimental pH 14.0,

corresponding to the proposed Ni-Fe moiety in EXAFS analysis (***Supplementary Notes 1***).

$Ni^{3+}Fe^{3+}$/$Ni^{2+}Fe^{4+}$

The oxidation of the $Ni^{2+}Fe^{3+}$ state will produce a $Ni^{3+}Fe^{3+}$ or $Ni^{2+}Fe^{4+}$ state. In the optimized structure of the $Ni^{2+}Fe^{3+}$ oxidized state, the –H_2O coordinated with Fe rather than Ni, undergoes deprotonation, forming a geometry where the –OH group on the axial position bonds to the Fe site while two H_2O coordinated with the Ni. The spin density of the Fe is 3.17 and −1.55 for the Ni, indicating the $Ni^{2+}Fe^{4+}$ state $[Ni^{2+}(OH_2)_2O_2Fe^{4+}(OH_2)_3(OH)]^{1+}$. Its triplet state (***Supplementary Fig. 136c***) is 10.2 kcal mol^{-1} lower in energy compared to its singlet state and 16.3 kcal mol^{-1} lower than the quintet state. However, this $Ni^{2+}Fe^{4+}$ state ($[Ni^{2+}(OH_2)_2O_2Fe^{4+}(OH_2)_3(OH)]^{1+}{}_T$) could be further deprotonated, forming various configurations.

Deprotonation of the –OH group leads to $[Ni^{2+}(OH_2)_2O_2Fe^{4+}(OH_2)_3(O)]_{Qu}$ (Qu represents quintet, ***Supplementary Fig. 136d***), which is 23.9 kcal mol^{-1} and 14.1 kcal mol^{-1} lower in energy compared to its singlet state and triplet state, respectively. However the pK_a of ($[Ni^{2+}(OH_2)_2O_2Fe^{4+}(OH_2)_3(OH)]^{1+}{}_T$/$[Ni^{2+}(OH_2)_2O_2Fe^{4+}(OH_2)_3(O)]_{Qu}$ pair is 20.6. Deprotonation of the Ni^{2+} H_2O, which forms a hydrogen bond with the –OH group of Fe site, leads to $[Ni^{3+}(OH)(OH_2)O_2Fe^{3+}(OH_2)_3(OH)]_{Qu}$ with a pK_a of 20.3 (***Supplementary Fig. 136e***). Its quintet state is 1.7 kcal mol^{-1} and 3.8 kcal mol^{-1} lower in energy than its singlet state and triplet state, respectively. Deprotonation of another Ni^{2+} H_2O leads to the formation of $[Ni^{3+}(OH)(OH_2)O_2Fe^{3+}(OH_2)_3(OH)]_{Qu}$ with a pK_a of 15.3 (***Supplementary Fig. 136f***). While deprotonation of the Fe^{4+} H_2O group is more favored, forming $[Ni^{2+}(OH_2)_2O_2Fe^{4+}(OH_2)_2(OH)_2]_T$ (***Supplementary Fig. 136g***) with two –OH groups axially coordinated to the Fe^{4+}. The triplet state is 30.2 kcal mol^{-1} and 11.4 kcal mol^{-1} lower in energy than its singlet state and quintet state, respectively. The calculated pK_a of $[Ni^{2+}(OH_2)_2O_2Fe^{4+}(OH_2)_3(OH)]^{1+}{}_T$/$[Ni^{2+}(OH_2)_2O_2Fe^{4+}(OH_2)_2(OH)_2]_T$ is 7.0. Hence, $[Ni^{2+}(OH_2)_2O_2Fe^{4+}(OH_2)_2(OH)_2]_T$ is the stable state for the $Ni^{2+}Fe^{4+}$ under experimental pH conditions. The oxidation of the $[Ni^{2+}(OH_2)_2O_2Fe^{3+}(OH_2)_4]^{1+}{}_Q$ will form the $[Ni^{2+}(OH_2)_2O_2Fe^{4+}(OH_2)_2(OH)_2]_T$, through two proton-coupled one-electron transfer, with an oxidation potential of 0.14 eV at pH 14.

$Ni^{3+}Fe^{4+}$

The oxidation of $[Ni^{2+}(OH_2)_2O_2Fe^{4+}(OH_2)_2(OH)_2]_T$ forms different structures. Among these structures, the proton-coupled electron transfer on the Ni^{2+} site will form the $Ni^{3+}Fe^{4+}$. Its doublet state structure $[Ni^{3+}(OH_2)(OH)O_2Fe^{4+}(OH_2)_2(OH)_2]_D$ (***Supplementary Fig. 136h***) is -8.7 kcal mol^{-1} lower in energy than its quartet state. However, this $Ni^{3+}Fe^{4+}$ state with two –OH groups bonded to Fe^{4+} ion, disagrees with experimental results that the Fe^{4+}=O state is responsible for water oxidation. Alternatively, the proton-coupled electron transfer on the Fe^{4+} site forms the doublet state of $[Ni^{2+}(OH_2)_2O_2Fe^{5+}(OH_2)_2(OH)(O)]_D$ (***Supplementary Fig. 136i***). This doublet state is 5.9 kcal mol^{-1} lower in energy than the quartet state. In its quartet state structure, one water hydrogen in Ni^{2+} transfers to the –OH group of Fe^{5+} site, forming the geometry of $[Ni^{3+}(OH_2)(OH)O_2Fe^{4+}(OH_2)_3(O)]_Q$ (***Supplementary Fig. 136j***). The calculated oxidation potential for the $[Ni^{2+}(OH_2)_2O_2Fe^{4+}(OH_2)_2(OH)_2]_T$/$[Ni^{2+}(OH_2)_2O_2Fe^{5+}(OH_2)_2(OH)(O)]_D$ couple is 0.63 eV at pH 14. From a numerical standpoint, this closely aligns with the experimental oxidation potential of $Ni^{2+}Fe^{3+}$. However, there is a significant discrepancy between this and the experimental structure.

Supplementary Notes 6

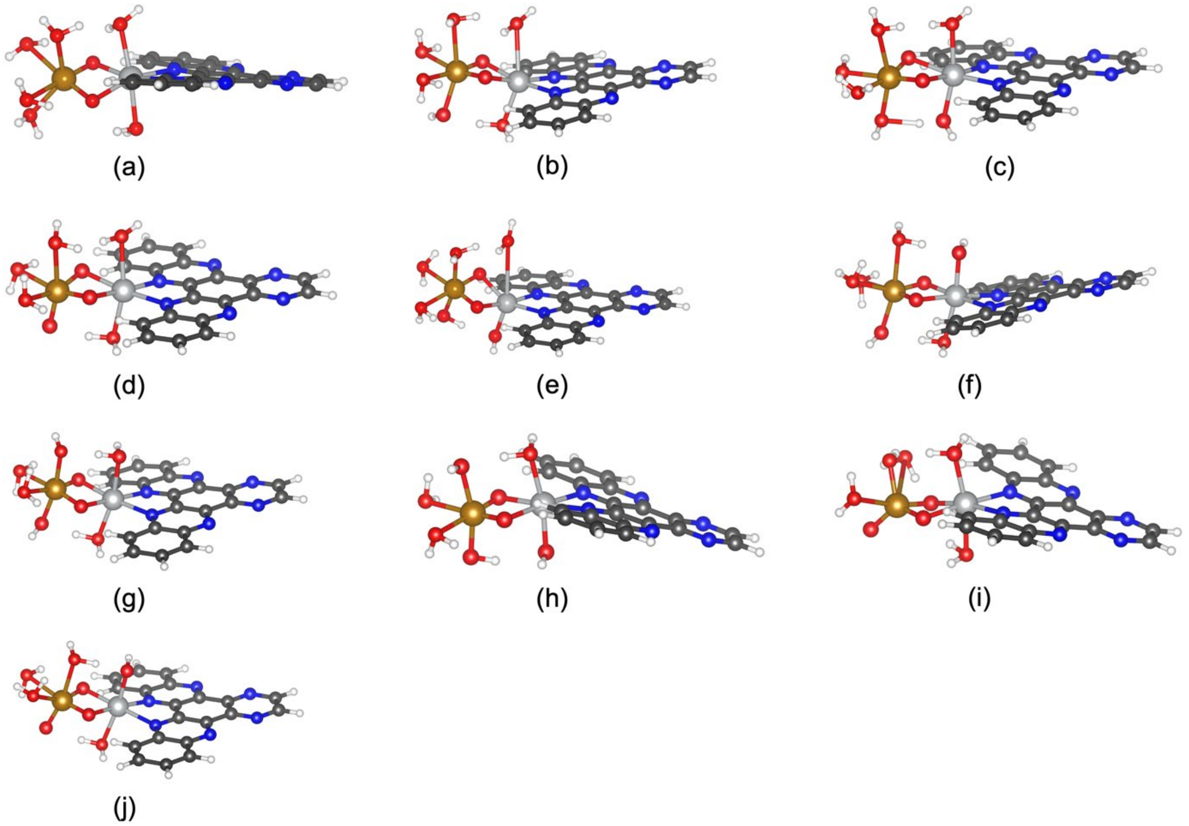

Supplementary Fig. 136 The optimized structures of various Ni–(*μ*–O)–Fe species. (a) $[Ni^{2+}(OH_2)_2O_2Fe^{3+}(OH_2)_4]^{1+}{}_{Q}$, (b) $[Ni^{2+}(OH_2)_2O_2Fe^{3+}(OH_2)_3(OH)]_{D}$, (c) $[Ni^{2+}(OH_2)_2O_2Fe^{4+}(OH_2)_3(OH)]^{1+}{}_{T}$. (d) $[Ni^{2+}(OH_2)_2O_2Fe^{4+}(OH_2)_3(O)]_{Qu}$, (e) $[Ni^{3+}(OH)(OH_2)O_2Fe^{3+}(OH_2)_3(OH)]_{Qu}$, (f) $[Ni^{3+}(OH)(OH_2)O_2Fe^{3+}(OH_2)_3(OH)]_{Qu}$, (g) $[Ni^{2+}(OH_2)_2O_2Fe^{4+}(OH_2)_2(OH)_2]_{T}$, (h) $[Ni^{3+}(OH_2)(OH)O_2Fe^{4+}(OH_2)_2(OH)_2]_{D}$, (i) $[Ni^{2+}(OH_2)_2O_2Fe^{5+}(OH_2)_2(OH)(O)]_{D}$, (j) $[Ni^{3+}(OH_2)(OH)O_2Fe^{4+}(OH_2)_3(O)]_{Q}$.

6.4 Calculations on Aza-CMP-NiFe with protonated bridging oxygen atoms

The oxygen atoms bridging Ni and Fe atoms have been reported to be protonated,[37, 38, 133] hence the full reaction processes based on the protonated bridge oxygen atoms have been further calculated.

$Ni^{2+}Fe^{3+}$

In the first protonated structure $[Ni^{2+}(OH_2)_2OOHFe^{3+}(OH_2)_4]^{2+}{}_{Q}$ (***Supplementary Fig. 137a***), the quartet state is 1.7 kcal mol^{-1} lower in energy compared to its double state. With further protonation, the quartet state $[Ni^{2+}(OH_2)_2(OH)_2Fe^{3+}(OH_2)_4]^{3+}{}_{Q}$ (***Supplementary Fig. 137b***) is also more stable than the doublet state, 10.7 kcal mol^{-1} lower in energy. The calculated first and second hydrogenation steps have reaction energies of −1.7 kcal mol^{-1} and −13.6 kcal mol^{-1}, respectively, indicating that protonation on the two bridging oxygen atoms is feasible.

The deprotonation of the –H_2O group coordinated with Fe^{3+} was calculated with different spin states and configurations, similar to the model in which bridging oxygen atoms are not protonated. The most stable structure is the $[Ni^{2+}(OH_2)_2(OH)_2Fe^{3+}(OH_2)_3(OH)]^{2+}{}_{Q}$ (***Supplementary Fig. 137c***) with the –OH group axially positioned to the Fe^{3+} site. This configuration is 0.2 kcal mol^{-1} lower compared to its doublet state, and 12.3 kcal mol^{-1} and 1.6 kcal mol^{-1} lower than the doublet and quartet state with the –OH group equatorially coordinated to the Fe^{3+} site, respectively. The calculated pK_a for $[Ni^{2+}(OH_2)_2(OH)_2Fe^{3+}(OH_2)_4]^{3+}{}_{Q}$/$[Ni^{2+}(OH_2)_2(OH)_2Fe^{3+}(OH_2)_3(OH)]^{2+}{}_{Q}$ is 14.4. Hence, $[Ni^{2+}(OH_2)_2(OH)_2Fe^{3+}(OH_2)_4]^{3+}{}_{Q}$ is the stable state at pH 14.

Supplementary Notes 6

$Ni^{3+}Fe^{3+}$

The oxidation of $[Ni^{2+}(OH_2)_2(OH)_2Fe^{3+}(OH_2)_4]^{3+}{}_Q$ leads to a $Ni^{3+}Fe^{3+}$ state, of which the stable state is the quintet state with the Ni^{3+}–OH group forming a hydrogen bond with the Fe^{3+}–OH group (***Supplementary Fig. 137d***). This state is 8.7 kcal mol^{-1} and 9.8 kcal mol^{-1} lower in energy compared to its singlet and triplet state, and 1.7 kcal mol^{-1}, 9.1 kcal mol^{-1}, 7.5 kcal mol^{-1} lower than the singlet, triplet, and quartet state with the –OH group on the opposite side of Ni^{3+} site (***Supplementary Fig. 137e***), respectively. The calculated oxidation potential for $[Ni^{2+}(OH_2)_2(OH)_2Fe^{3+}(OH_2)_4]^{3+}{}_Q/[Ni^{3+}(OH)(OH_2)(OH)_2Fe^{3+}(OH_2)_3(OH)]^{2+}{}_Q$ pair with a $2H^+/1e^-$ process is 0.77 eV at pH 14.

$Ni^{3+}Fe^{4+}$=O

The further oxidation of the $Ni^{3+}Fe^{3+}$ forms the $Ni^{3+}Fe^{4+}$=O. The quartet state $[Ni^{3+}(OH)(OH_2)(OH)_2Fe^{3+}(OH_2)_3{=}O]^{2+}{}_Q$ (***Supplementary Fig. 137f***) is 6.4 kcal mol^{-1} lower in energy compared to its doublet state. The oxidation of $Ni^{3+}Fe^{3+}$ through a $1H^+/1e^-$ process results in the $Ni^{3+}Fe^{4+}$=O state with an oxidation potential of 0.84 eV at pH 14, a value very close to that required to generate **$Ni^{3+}Fe^{3+}$**. When the pH is decreased to around 13, the oxidation potential for $Ni^{2+}Fe^{3+}$/**$Ni^{3+}Fe^{3+}$** shifts to 0.89 eV, while the oxidation potential for $Ni^{3+}Fe^{3+}$/ $Ni^{3+}Fe^{4+}$=O changes to 0.90 eV. This indicates that the two oxidation processes occur almost concurrently, facilitating direct oxidation from the ground state $Ni^{2+}Fe^{3+}$ to the active $Ni^{3+}Fe^{4+}$=O via a $3H^+/2e^-$ process, consistent with experimental observations in ***Supplementary Fig. 72***. Since the spin is typically located in Fe–O *dπ-pπ* orbitals distributed over the two atoms, it is therefore difficult to be absolutely conclusive about the oxidation state of the metal and the oxygen. The short Fe–O bond length of 1.61 Å indicates the oxidation state is better assigned as Fe^{4+}=O in DFT calculations.

In summary, during the water activation processes, the species $[Ni^{2+}(OH_2)_2(OH)_2Fe^{3+}(OH_2)_4]^{3+}{}_Q$ emerges as the stable configuration at pH 14. This state undergoes oxidation to yield a quintet $Ni^{3+}Fe^{3+}$ state, wherein the most stable form is achieved when the Ni^{3+}–OH group hydrogen-bonds with the Fe^{3+}–OH group. Subsequent oxidation of the $Ni^{3+}Fe^{3+}$ configuration leads to the formation of a quartet state $[Ni^{3+}(OH)(OH_2)(OH)_2Fe^{4+}(OH_2)_3{=}O]^{2+}{}_Q$, which exhibits an oxidation potential nearly equivalent to that of the $Ni^{2+}Fe^{3+}/Ni^{3+}Fe^{3+}$ couple. This facilitates a uniform oxidation pathway from $Ni^{2+}Fe^{3+}$ to $Ni^{3+}Fe^{4+}$ through an integrated $3H^+/2e^-$ process.

Supplementary Notes 6

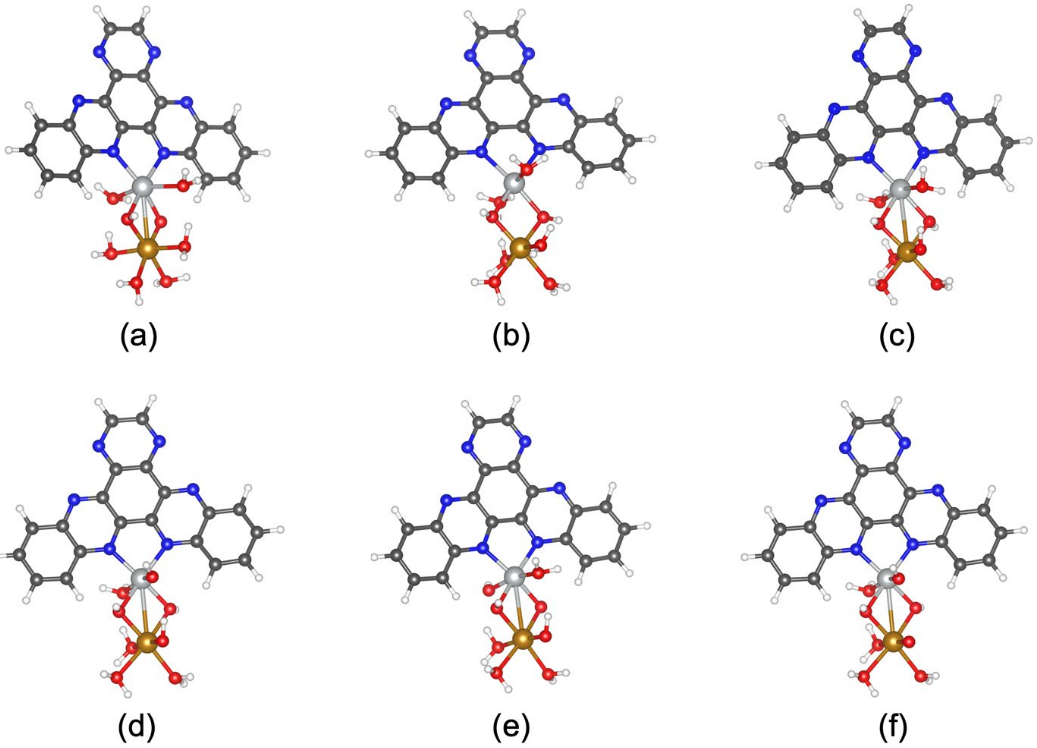

Supplementary Fig. 137 The optimized structures of various Ni–(*μ*–OH)–Fe species. (a) $[Ni^{2+}(OH_2)_2OOHFe^{3+}(OH_2)_4]^{2+}_Q$, (b) $[Ni^{2+}(OH_2)_2(OH)_2Fe^{3+}(OH_2)_3(OH)]^{2+}_Q$, (c) $[Ni^{3+}(OH)(OH_2)(OH)_2Fe^{3+}(OH_2)_3(OH)]^{2+}_Q$, (d) $[Ni^{3+}(OH)(OH_2)(OH)_2Fe^{3+}(OH_2)_3(OH)]^{2+}_Q$. (e) isomer of $[Ni^{3+}(OH)(OH_2)(OH)_2Fe^{3+}(OH_2)_3(OH)]^{2+}_Q$. (f) $[Ni^{3+}(OH)(OH_2)(OH)_2Fe^{3+}(OH_2)_3{=}O]^{2+}_Q$.

We calculated the Natural Population Analysis (NPA) and Hierschfield method at the m06l/LANL2DZ level of theory using Gaussian software, as these calculations are not available in the Jaguar software package, which was used for the main calculations in this manuscript (***Supplementary Table 14*** and ***Supplementary Fig. 138***). The following analysis is based on Hierschfield spin density:

(e) $[Ni^{2+}(OH_2)_2(OH)_2Fe^{3+}(OH_2)_4]^{3+}_D$ (S = 1/2, multiplicity = 2). Ni(II) (d^8) state maintains a spin density of 1.66, indicating two unpaired electrons and some spin delocalization by the Fe interaction. Fe(III) (d^5) state maintains a spin density of likely −0.49, indicating only one unpaired electron and the Fe center is antiferromagnetically coupled to Ni. This suggests antiferromagnetic (AFM) coupling where Fe's spin is spin up while Ni's spin is spin down. The AFM coupling gives a net S = 1/2 doublet state.

(f) $[Ni^{2+}(OH_2)_2(OH)_2Fe^{3+}(OH_2)_4]^{3+}_Q$ (S = 3/2, multiplicity = 4). The calculated spin density of Ni(II) (d^8) state is 1.66, meaning it has two unpaired electrons and is partially magnetized by the Fe interaction and also with ligands. Fe(III) (d^5) state maintains a spin density is 1.55, indicating three unpaired electrons and delocalized. Both Ni and Fe have unpaired spins aligned in the same direction. This indicates a FM coupling between Ni and Fe centers. The total spin (S = 3/2) is likely consistent with two unpaired electrons on Ni and one unpaired electron on Fe, with some delocalization.

(g) $[Ni^{3+}(OH)(OH_2)(OH)_2Fe^{3+}(OH_2)_3(OH)]^{2+}_T$ (S = 1, multiplicity = 3). The computed spin density of Ni(III) (d^7) state is 1.29, with some spin delocalization. The calculated spin density of Fe(III) (d^5) state is 0.90, which is lower than expected, suggesting some delocalization or AFM coupling with Ni. Ni and Fe are likely FM coupled. The total spin (S = 1) is likely consistent with one unpaired electron on Ni and one unpaired electron on Fe.

(h) $[Ni^{3+}(OH)(OH_2)(OH)_2Fe^{3+}(OH_2)_3(OH)]^{2+}_{Qu}$, (S = 2, multiplicity = 5). The computed spin density of Ni(III) (d^7) state is 1.29, with some spin delocalization. The calculated spin density of Fe(III)

(d^5) state is 2.67, indicating three unpaired electrons and delocalized. Ni and Fe are FM coupled. The total spin (S = 2) is likely consistent with one unpaired electron on Ni and three unpaired electrons on Fe.

(i) $[Ni^{3+}(OH)(OH_2)(OH)_2Fe^{4+}(OH_2)_3{=}O]^{2+}{}_D$, (S = 1/2, multiplicity = 2). The calculated spin density of Ni(III) (d^7) state is −1.33, suggesting it is one unpaired electron and AFM coupled with Fe. Fe(IV) (d^4) state has a spin density of 1.11, while its coordinated oxo has a spin density of 0.98, indicating strong FM interactions with the oxo. Ni and Fe are AFM coupled. The total spin (S = 1/2) is likely consistent with spin down for Ni and spin up for Fe and spin delocalized to the oxo.

(j) $[Ni^{3+}(OH)(OH_2)(OH)_2Fe^{4+}(OH_2)_3{=}O]^{2+}{}_Q$, (S = 3/2, multiplicity = 4). The Ni^{3+} (d^7) state has a spin of 1.39, meaning it is one unpaired electron. In the Fe(IV) (d^4) state, the calculated Fe spin density is 1.25, while its coordinated oxo has a spin density of 0.78, indicating strong FM interactions with the oxo. The short Fe=O bond length of 1.61 Å indicates the oxidation state is better assigned as Fe(IV). A Fe=O double bond with the unpaired electron on oxygen due to the electron polarization toward the oxygen atom from the highly charged Fe^{4+} center. Ni and Fe are FM coupled. The total spin (S = 3/2) is likely consistent with spin up for Ni and spin up for Fe and spin density delocalized to the oxo.

Since the spin is typically located in Fe=O *dπ-pπ* orbitals, and it is distributed over the two atoms, it is therefore difficult to be absolutely conclusive about the oxidation state of the metal and the oxygen. In this Valence Bond study,[134] they conclude that the electronic structure has significant contributions from several resonance forms, and depending on the ligands that the dominant resonance form could change. What is clearer, on the other hand, is that the spin density of the species (i) and (j) is similar and that the change in multiplicity is due to a change at the adjacent Ni-site.

Supplementary Table 14 The charges of atoms from NPA and Hierschfield method.

Species	Ni		Fe	
	NPA	Hirshfeld spin densities	NPA	Hirshfeld spin densities
a	1.20203	1.558986		
b	0.87844	0.910705		
c	1.30234	1.783260		
d	0.81283	0.898136		
e	1.31694	1.656309	0.23513	−0.493792
f	1.32562	1.657366	1.35190	1.554223
g	1.08077	1.290829	0.89632	0.898749
h	1.07753	1.293842	1.86412	2.667769
i	−0.18098	−1.329640	0.88928	1.112694
j	1.15388	1.393580	0.91898	1.245915

Supplementary Notes 6

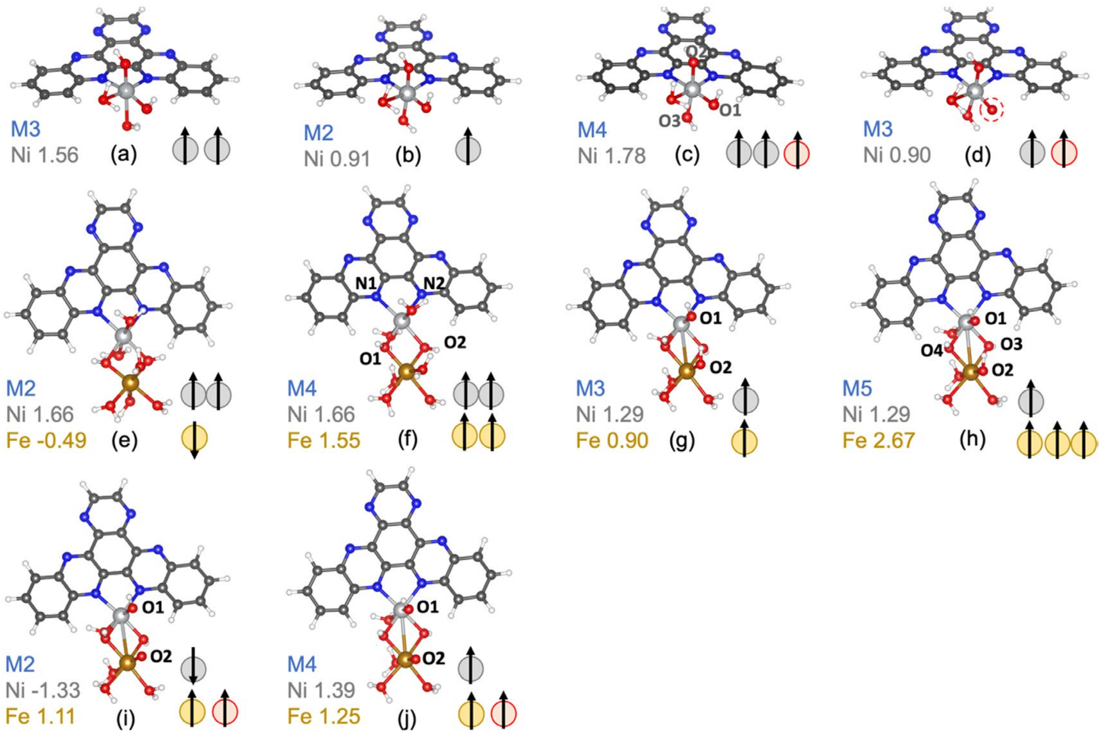

Supplementary Fig. 138 The optimized structures of various species. (a) $[Ni^{2+}(OH)_2(OH_2)_2]_T$. (b) $[Ni^{3+}(OH)_3(OH_2)]_D$. (c) $[Ni^{3+}(OH)_3(OH_2)]_Q$. (d) $[Ni^{4+}{=}O(OH)_2(OH_2)]_T$. (e) $[Ni^{2+}(OH_2)_2(OH)_2Fe^{3+}(OH_2)_4]^{3+}{}_D$, (f) $[Ni^{2+}(OH_2)_2(OH)_2Fe^{3+}(OH_2)_4]^{3+}{}_Q$, (g) $[Ni^{3+}(OH)(OH_2)(OH)_2Fe^{3+}(OH_2)_3(OH)]^{2+}{}_T$, (h) $[Ni^{3+}(OH)(OH_2)(OH)_2Fe^{3+}(OH_2)_3(OH)]^{2+}{}_{Qu}$, (i) $[Ni^{3+}(OH)(OH_2)(OH)_2Fe^{4+}(OH_2)_3{=}O]^{2+}{}_D$, (j) $[Ni^{3+}(OH)(OH_2)(OH)_2Fe^{4+}(OH_2)_3{=}O]^{2+}{}_Q$.

O–O bond formation on Ni-Fe sites

The nucleophilic attack by OH^- on Fe^{4+}=O to facilitate O–O bond formation is initially studied to model the reaction under strongly alkaline conditions. In this treatment, the OH^- group is associated with an additional water molecule to form an OH^---H_2O complex. This approach essentially mimics the limiting case where the rate constant for the solution-anion mediated process (k_B) is sufficiently high (thereby favoring the OH^- nucleophilic attack over the H_2O attack, which necessitates initial deprotonation), ensuring that the entire reaction is governed by the rate constant for the relay step (k_R). With the $Ni^{3+}Fe^{4+}$=O, the process of OH^- nucleophilic attacking the Fe^{4+}=O involves a reaction free energy and activation free energy of −0.8 kcal mol^{-1} and 13.5 kcal mol^{-1}, respectively (***Supplementary Fig. 139***). Throughout the reaction process, the OH^- species is positioned between the Fe^{4+}=O and the Ni^{3+}–OH group, and stabilized by a hydrogen bond network involving the –OH group and an H_2O molecule. The hydrogen atom of the OH^- species donates hydrogen bonds to the oxygen atom of the Ni^{3+}–OH group and accepts hydrogen bonds from the hydrogen atom of an H_2O molecule. At the reactant state, the distance between the OH^- hydrogen atom and the oxygen atom of the Ni^{3+}–OH group is 2.23 Å, which decreases to 1.92 Å at the transition state and 1.75 Å at the product state. Meanwhile, the distance between the OH^- oxygen atom and the H_2O hydrogen atom is 2.25 Å at the reactant state, decreasing to 2.12 Å at the transition state and 2.11 Å at the product state. Once the O–O bond is formed, the hydrogen of Fe–OOH species can be released and transferred to the Ni^{3+}–OH group with –OO coordinated with the Fe atom, forming a Ni^{3+}–OH_2 group. During the –OOH hydrogen transfer, the Fe–O distance increases from 1.61 Å to 1.78 Å. The quartet state is −19.4 kcal mol^{-1} lower in energy compared to its doublet state, providing a reaction free energy of −38.0 kcal mol^{-1}. In this structure, the terminal oxygen atom of Fe–OO forms hydrogen bonds with the Ni^{3+}–OH_2 and one H_2O

molecule, with a distance of 1.70 Å and 1.93 Å, respectively. Hence, the reaction process involves protonation on the two bridging oxygen atoms, one step of $2H^+/1e^-$ transfer to form $Ni^{3+}Fe^{3+}$, and subsequently one step of $1H^+/1e^-$ transfer to form the $Ni^{3+}Fe^{4+}$=O state. This state will then be attacked by –OH species with the aid of hydrogen bonds by the Ni^{3+}–OH group and one H_2O molecule. Subsequently, the proton transfer from Fe–OOH will be facilitated by the hydrogen bond formed with the Ni^{3+}–OH group.

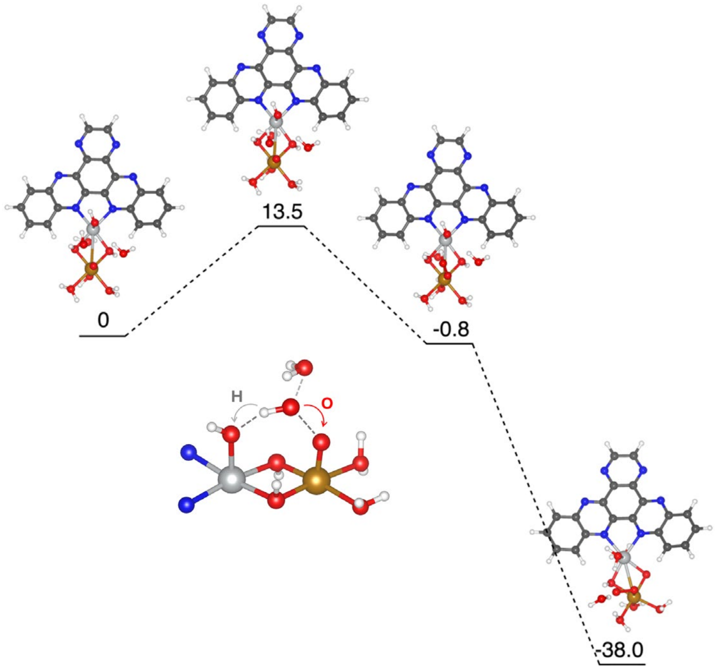

Supplementary Fig. 139 The reaction energy profile of the OH^- nucleophilic attacking pathway with optimized structures. The values are given in units of kcal mol^{-1}.

For comparison, consider another scenario under extreme conditions where water directly attacks Fe^{4+}=O to form the O–O bond. In this case, the reaction rate is limited by the $MOOH_2$ deprotonation process with no proton transfer mediator (anions) in the solution. This reaction pathway has a reaction free energy and activation free energy of 3.9 kcal mol^{-1} and 21.2 kcal mol^{-1}, respectively (***Supplementary Fig. 140***). The transition state is stabilized by a hydrogen bonding network, wherein the –OH group coordinated with the Ni^{3+} site and one bridging hydrogen forms hydrogen bonds with the attacking H_2O (1.72 Å and 1.75 Å) (***Supplementary Fig. 141a***). Additionally, the O–O bond formation in a borate buffer solvent has also been modeled by incorporating a $B(OH)_4^-$ molecule into the system, simulating near-neutral pH conditions. This reaction pathway shows a reaction free energy and activation free energy of −9.5 kcal mol^{-1} and 18.7 kcal mol^{-1}, respectively (***Supplementary Fig. 140***). In the transition state, similar to the transition state of the OH^- nucleophilic attacking pathway, the –OH group coordinated with the Ni^{3+} site, and one bridging hydrogen formed hydrogen bonds with the attacking H_2O molecule (1.65 Å and 2.22 Å). The H_2O is further stabilized by another hydrogen bond with the $B(OH)_4^-$ anion (akin to OH^---H_2O complex mentioned above) (***Supplementary Fig. 141b***). Overall, these calculations illustrate the O–O bonding process under various solution conditions. Under buffered conditions (with borate as the proton acceptor) and strong alkaline conditions (with OH^- as the proton acceptor), the activation energy required for the reaction is significantly lower compared to the direct water nucleophilic attack pathway. Furthermore, in all scenarios discussed above, the adjacent Ni^{3+}–OH sites form hydrogen bonds with the attacking H_2O/OH^- molecules, assisting the O–O bond formation and serving as intramolecular proton transfer relays. These calculations align with experimental results observed under different buffered conditions, thereby

supporting the validity of the virtual transition state concept and highlighting the critical role of proton transfer sites within the system.

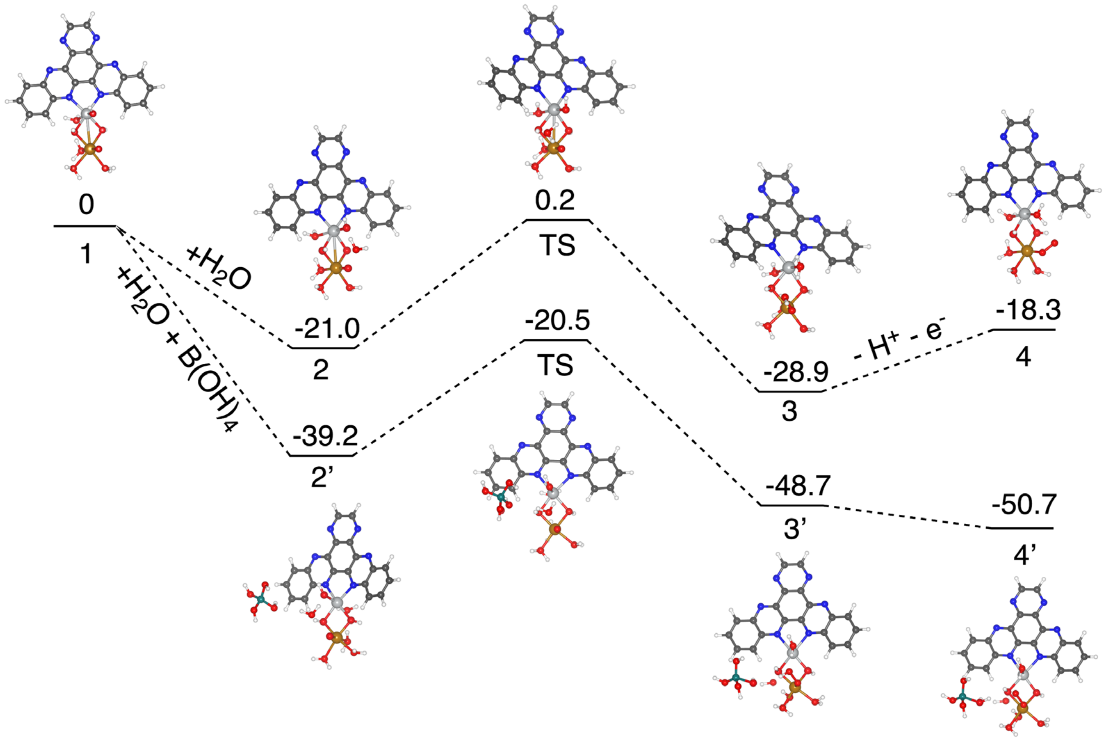

Supplementary Fig. 140 Reaction energy profile of H_2O nucleophilic attack on the Fe^{4+}=O with and without an adjacent $B(OH)_4^-$ molecule. Energy values are given in kcal mol^{-1}.

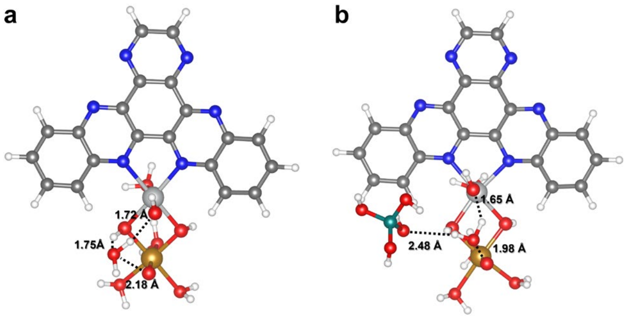

Supplementary Fig. 141 Transition state structures of H_2O nucleophilic attack on the Fe^{4+}=O. (a) without and (b) with an adjacent $B(OH)_4^-$ molecule. Distances are given in Å.

The counterpose corrections for water attack in both Ni species and Ni–(μ–O)–Fe species have been calculated. The calculated values are 1.32 kcal mol^{-1} and −0.98 kcal mol^{-1} for Ni and Ni–(μ–O)–Fe species, respectively (***Supplementary Figs. 142a*** and ***142b***). Gas-phase DFT models generally overestimate entropy contributions with respect to solvation. Wertz has suggested empirically reducing the translational and rotational components of the entropy.[135] We used the Wertz model to obtain the solvent-corrected free energy at standard conditions using the following equation and update the energetic profile of Ni and Ni–(μ–O)–Fe species (***Supplementary Figs. 143a*** and ***143b***).

$$\Delta G_w^{\ddagger} = \Delta G_{DFT}^{\ddagger} + (1 - 0.46)\, T\, (S_{TS}^{o} - S_{RC}^{o}) \qquad (80)$$

Where $\Delta G_w^{\ddagger}$ is solvent-corrected free energy at standard conditions, $S°$ is gas-phase entropy, and T is temperature. Generally, for the Ni species, the entropy increases from species 1 to 4, while it decreases from species 4 to 5 during the O–O bond formation. As a result, the Wertz model gives a free energy correction of around ±1.6 kcal mol^{-1}. For the Ni–(μ–O)–Fe species, the overall correction given by the Wertz model is approximately ±1.3 kcal mol^{-1}. A slightly larger correction of −2.3 kcal

mol^{-1} was observed from species 3 to 4, likely due to the reaction occurring at high pH 14 conditions.

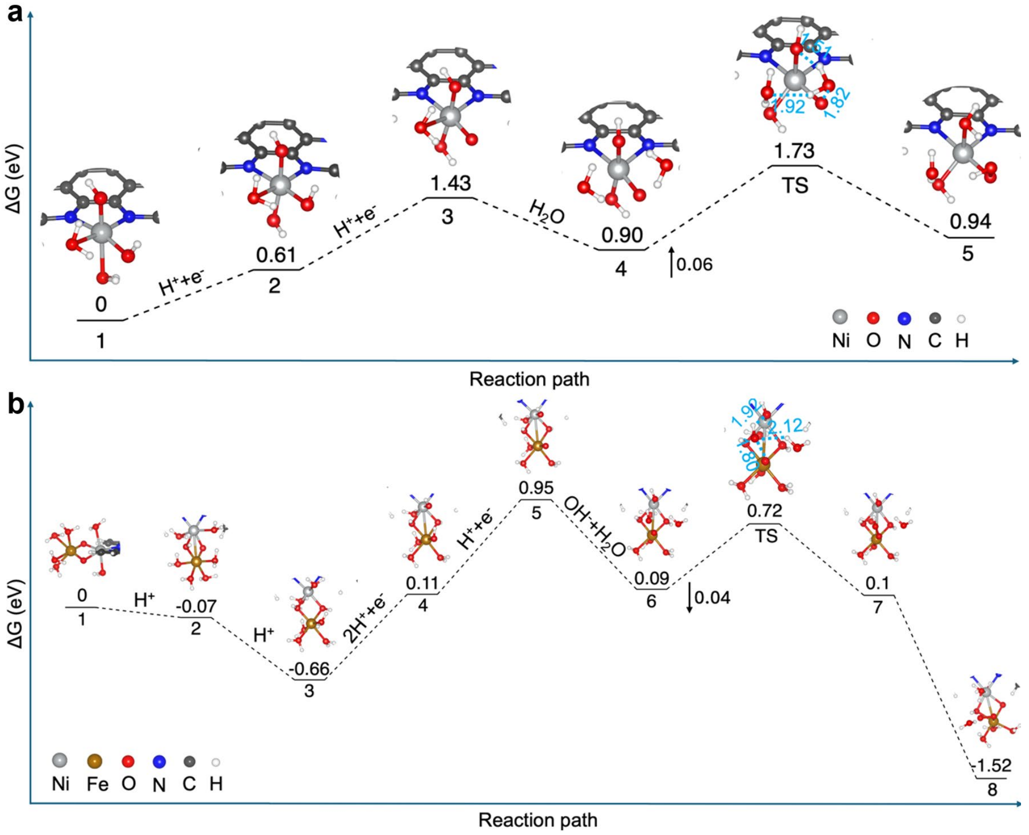

Supplementary Fig. 142 The energetic profile. (a) Ni species and (b) Ni–(μ–O)–Fe species with counterpoise corrections.

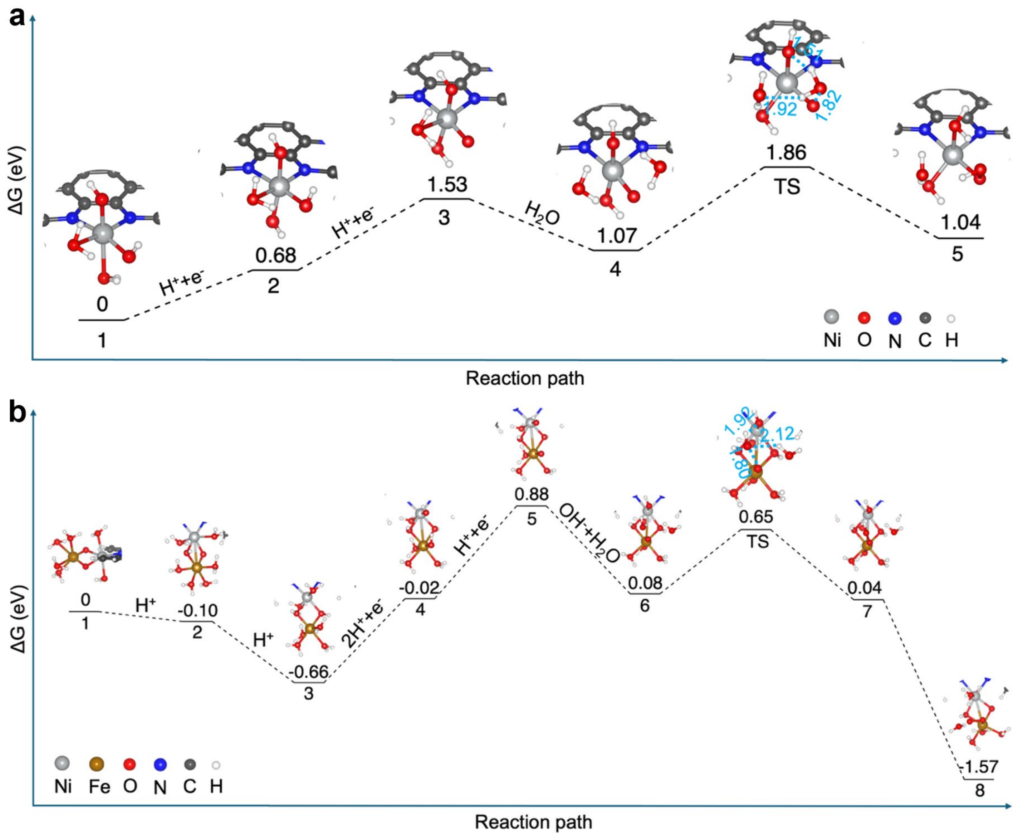

Supplementary Fig. 143 The energetic profile. (a) Ni species and (b) Ni–(μ–O)–Fe species with Wertz model and counterpoise corrections.

Supplementary References

References

1. Briega-Martos V, Ferre-Vilaplana A, de la Peña A, Segura JL, Zamora F, Feliu JM*, et al.* An Aza-Fused π-Conjugated Microporous Framework Catalyzes the Production of Hydrogen Peroxide. *ACS Catal* 2017, **7**(2)**:** 1015-1024.

2. Trotochaud L, Young SL, Ranney JK, Boettcher SW. Nickel–Iron Oxyhydroxide Oxygen-Evolution Electrocatalysts: The Role of Intentional and Incidental Iron Incorporation. *J Am Chem Soc* 2014, **136**(18)**:** 6744-6753.

3. Yang H, Li F, Zhan S, Liu Y, Li W, Meng Q*, et al.* Intramolecular hydroxyl nucleophilic attack pathway by a polymeric water oxidation catalyst with single cobalt sites. *Nat Catal* 2022, **5**(5)**:** 414-429.

4. Ye C, Fu R, Hu J, Hou L, Ding S. Carbon-13 chemical shift anisotropies of solid amino acids. *Magn Reson Chem* 1993, **31**(8)**:** 699-704.

5. Miyazato I, Takahashi L, Takahashi K. Automatic oxidation threshold recognition of XAFS data using supervised machine learning. *Mol Syst Des Eng* 2019, **4**(5)**:** 1014-1018.

6. Bunău O, Joly Y. Self-consistent aspects of x-ray absorption calculations. *J Phys: Condens Matter* 2009, **21**(34)**:** 345501.

7. Li W, Gao X, Wang X, Xiong D, Huang P-P, Song W-G*, et al.* From water reduction to oxidation: Janus Co-Ni-P nanowires as high-efficiency and ultrastable electrocatalysts for over 3000 h water splitting. *J Power Sources* 2016, **330:** 156-166.

8. Anantharaj S, Ede SR, Karthick K, Sam Sankar S, Sangeetha K, Karthik PE*, et al.* Precision and correctness in the evaluation of electrocatalytic water splitting: revisiting activity parameters with a critical assessment. *Energy Environ Sci* 2018, **11**(4)**:** 744-771.

9. Stevens MB, Enman LJ, Batchellor AS, Cosby MR, Vise AE, Trang CDM*, et al.* Measurement Techniques for the Study of Thin Film Heterogeneous Water Oxidation Electrocatalysts. *Chem Mater* 2017, **29**(1)**:** 120-140.

10. Wang J, Gan L, Zhang W, Peng Y, Yu H, Yan Q*, et al.* In situ formation of molecular Ni-Fe active sites on heteroatom-doped graphene as a heterogeneous electrocatalyst toward oxygen evolution. *Sci Adv* 2018, **4**(3).

11. Bard AJ, Faulkner LR. *Electrochemical methods: fundamentals and applications*, vol. 2. Wiley New York, 1980.

12. Li W, Li F, Yang H, Wu X, Zhang P, Shan Y*, et al.* A bio-inspired coordination polymer as outstanding water oxidation catalyst via second coordination sphere engineering. *Nat Commun* 2019, **10**(1)**:** 5074.

13. Li F, Yang H, Zhuo Q, Zhou D, Wu X, Zhang P*, et al.* A Cobalt@Cucurbit[5]uril Complex as a Highly Efficient Supramolecular Catalyst for Electrochemical and Photoelectrochemical Water Splitting. *Angew Chem Int Ed* 2021, **60**(4)**:** 1976-1985.

Supplementary References

14. Imberti S, Botti A, Bruni F, Cappa G, Ricci MA, Soper AK. Ions in water: The microscopic structure of concentrated hydroxide solutions. *J Chem Phys* 2005, **122**(19)**:** 194509.

15. Yang C, Fontaine O, Tarascon J-M, Grimaud A. Chemical Recognition of Active Oxygen Species on the Surface of Oxygen Evolution Reaction Electrocatalysts. *Angew Chem Int Ed* 2017, **56**(30)**:** 8652-8656.

16. Kuang Z, Liu S, Li X, Wang M, Ren X, Ding J*, et al.* Topotactically constructed nickel–iron (oxy)hydroxide with abundant in-situ produced high-valent iron species for efficient water oxidation. *J Energy Chem* 2021, **57:** 212-218.

17. Bochevarov AD, Harder E, Hughes TF, Greenwood JR, Braden DA, Philipp DM*, et al.* Jaguar: A high-performance quantum chemistry software program with strengths in life and materials sciences. *Int J Quantum Chem* 2013, **113**(18)**:** 2110-2142.

18. Zhao Y, Truhlar DG. A new local density functional for main-group thermochemistry, transition metal bonding, thermochemical kinetics, and noncovalent interactions. *J Chem Phys* 2006, **125**(19)**:** 194101.

19. Hay PJ, Wadt WR. Ab initio effective core potentials for molecular calculations. Potentials for K to Au including the outermost core orbitals. *J Chem Phys* 1985, **82**(1)**:** 299-310.

20. Wehrens-Dijksma M, Notten PHL. Electrochemical Quartz Microbalance characterization of Ni(OH)2-based thin film electrodes. *Electrochim Acta* 2006, **51**(18)**:** 3609-3621.

21. Corrigan DA. The Catalysis of the Oxygen Evolution Reaction by Iron Impurities in Thin Film Nickel Oxide Electrodes. 1987, **134**(2)**:** 377-384.

22. Gong L, Yang H, Douka AI, Yan Y, Xia BY. Recent Progress on NiFe-Based Electrocatalysts for Alkaline Oxygen Evolution. *Adv Sustain Syst* 2021, **5**(1)**:** 2000136.

23. Mohammed-Ibrahim J. A review on NiFe-based electrocatalysts for efficient alkaline oxygen evolution reaction. *J Power Sources* 2020, **448:** 227375.

24. Gong M, Dai H. A mini review of NiFe-based materials as highly active oxygen evolution reaction electrocatalysts. *Nano Res* 2015, **8**(1)**:** 23-39.

25. Louie MW, Bell AT. An Investigation of Thin-Film Ni–Fe Oxide Catalysts for the Electrochemical Evolution of Oxygen. *J Am Chem Soc* 2013, **135**(33)**:** 12329-12337.

26. Abbott DF, Fabbri E, Borlaf M, Bozza F, Schäublin R, Nachtegaal M*, et al.* Operando X-ray absorption investigations into the role of Fe in the electrochemical stability and oxygen evolution activity of Ni1−xFexOy nanoparticles. *J Mater Chem A* 2018, **6**(47)**:** 24534-24549.

27. Görlin M, Chernev P, Ferreira de Araújo J, Reier T, Dresp S, Paul B*, et al.* Oxygen Evolution Reaction Dynamics, Faradaic Charge Efficiency, and the Active Metal Redox States of Ni–Fe Oxide Water Splitting Electrocatalysts. *J Am Chem Soc* 2016, **138**(17)**:** 5603-5614.

Supplementary References

28. Li N, Bediako DK, Hadt RG, Hayes D, Kempa TJ, von Cube F, *et al.* Influence of iron doping on tetravalent nickel content in catalytic oxygen evolving films. *Proc Natl Acad Sci USA* 2017, **114**(7)**:** 1486-1491.

29. Lee S, Bai L, Hu X. Deciphering Iron-Dependent Activity in Oxygen Evolution Catalyzed by Nickel–Iron Layered Double Hydroxide. *Angew Chem Int Ed* 2020, **59**(21)**:** 8072-8077.

30. Hunter BM, Thompson NB, Müller AM, Rossman GR, Hill MG, Winkler JR, *et al.* Trapping an Iron(VI) Water-Splitting Intermediate in Nonaqueous Media. *Joule* 2018, **2**(4)**:** 747-763.

31. Burke MS, Kast MG, Trotochaud L, Smith AM, Boettcher SW. Cobalt–Iron (Oxy)hydroxide Oxygen Evolution Electrocatalysts: The Role of Structure and Composition on Activity, Stability, and Mechanism. *J Am Chem Soc* 2015, **137**(10)**:** 3638-3648.

32. Zou S, Burke MS, Kast MG, Fan J, Danilovic N, Boettcher SW. Fe (Oxy)hydroxide Oxygen Evolution Reaction Electrocatalysis: Intrinsic Activity and the Roles of Electrical Conductivity, Substrate, and Dissolution. *Chem Mater* 2015, **27**(23)**:** 8011-8020.

33. Friebel D, Louie MW, Bajdich M, Sanwald KE, Cai Y, Wise AM, *et al.* Identification of Highly Active Fe Sites in (Ni,Fe)OOH for Electrocatalytic Water Splitting. *J Am Chem Soc* 2015, **137**(3)**:** 1305-1313.

34. Anantharaj S, Kundu S, Noda S. "The Fe Effect": A review unveiling the critical roles of Fe in enhancing OER activity of Ni and Co based catalysts. *Nano Energy* 2021, **80:** 105514.

35. Roger I, Shipman MA, Symes MD. Earth-abundant catalysts for electrochemical and photoelectrochemical water splitting. *Nat Rev Chem* 2017, **1:** 0003.

36. Stevens MB, Trang CDM, Enman LJ, Deng J, Boettcher SW. Reactive Fe-Sites in Ni/Fe (Oxy)hydroxide Are Responsible for Exceptional Oxygen Electrocatalysis Activity. *J Am Chem Soc* 2017, **139**(33)**:** 11361-11364.

37. Wang J, Gan L, Zhang W, Peng Y, Yu H, Yan Q, *et al.* In situ formation of molecular Ni-Fe active sites on heteroatom-doped graphene as a heterogeneous electrocatalyst toward oxygen evolution. *Sci Adv* 2018, **4**(3)**:** eaap7970.

38. Bai L, Hsu C-S, Alexander DTL, Chen HM, Hu X. Double-atom catalysts as a molecular platform for heterogeneous oxygen evolution electrocatalysis. *Nat Energy* 2021, **6**(11)**:** 1054-1066.

39. Zhu X, Zhang D, Chen C-J, Zhang Q, Liu R-S, Xia Z, *et al.* Harnessing the interplay of Fe–Ni atom pairs embedded in nitrogen-doped carbon for bifunctional oxygen electrocatalysis. *Nano Energy* 2020, **71:** 104597.

40. Ying Y, Luo X, Qiao J, Huang H. "More is Different:" Synergistic Effect and Structural Engineering in Double-Atom Catalysts. *Adv Funct Mater* 2021, **31**(3)**:** 2007423.

41. Sun J-F, Xu Q-Q, Qi J-L, Zhou D, Zhu H-Y, Yin J-Z. Isolated Single Atoms Anchored on N-Doped Carbon Materials as a Highly Efficient Catalyst for Electrochemical and Organic Reactions. *ACS Sustain Chem Eng*

2020, **8**(39)**:** 14630-14656.

42. Tavakkoli M, Nosek M, Sainio J, Davodi F, Kallio T, Joensuu PM*, et al.* Functionalized Carbon Nanotubes with Ni(II) Bipyridine Complexes as Efficient Catalysts for the Alkaline Oxygen Evolution Reaction. *ACS Catal* 2017, **7**(12)**:** 8033-8041.

43. Downie TC, Harrison W, Raper ES, Hepworth MA. A three-dimensional study of the crystal structure of nickel acetate tetrahydrate. *Acta Cryst B* 1971, **27**(3)**:** 706-712.

44. Colpas GJ, Maroney MJ, Bagyinka C, Kumar M, Willis WS, Suib SL*, et al.* X-ray spectroscopic studies of nickel complexes, with application to the structure of nickel sites in hydrogenases. *Inorg Chem* 1991, **30**(5)**:** 920-928.

45. Wang J, Ge X, Liu Z, Thia L, Yan Y, Xiao W*, et al.* Heterogeneous Electrocatalyst with Molecular Cobalt Ions Serving as the Center of Active Sites. *J Am Chem Soc* 2017, **139**(5)**:** 1878-1884.

46. Yamamoto M, Nishizawa Y, Chabera P, Li F, Pascher T, Sundstrom V*, et al.* Visible light-driven water oxidation with a subporphyrin sensitizer and a water oxidation catalyst. *Chem Commun* 2016, **52**(94)**:** 13702-13705.

47. Lei L, Guo X, Han X, Fei L, Guo X, Wang D-G. From Synthesis to Mechanisms: In-Depth Exploration of the Dual-Atom Catalytic Mechanisms Toward Oxygen Electrocatalysis. *Adv Mater* 2024, **36**(37)**:** 2311434.

48. Pu T, Ding J, Zhang F, Wang K, Cao N, Hensen EJM*, et al.* Dual Atom Catalysts for Energy and Environmental Applications. *Angew Chem Int Ed* 2023, **62**(40)**:** e202305964.

49. Mansour AN, Melendres CA. Characterization of Electrochemically Prepared γ-NiOOH by XPS. *Surf Sci Spectra* 1994, **3**(3)**:** 271-278.

50. Mansour AN, Melendres CA. Characterization of $Ni_2O_3{\cdot}6H_2O$ by XPS. *Surf Sci Spectra* 1994, **3**(3)**:** 263-270.

51. Mansour AN, Melendres CA. Characterization of Slightly Hydrated $Ni(OH)_2$ by XPS. *Surf Sci Spectra* 1994, **3**(3)**:** 247-254.

52. Wilson SA, Chen J, Hong S, Lee Y-M, Clémancey M, Garcia-Serres R*, et al.* $[Fe^{IV}{=}O(TBC)(CH_3CN)]^{2+}$: Comparative Reactivity of Iron(IV)-Oxo Species with Constrained Equatorial Cyclam Ligation. *J Am Chem Soc* 2012, **134**(28)**:** 11791-11806.

53. Wilke M, Farges Fo, Petit P-E, Brown GE, Jr., Martin Fo. Oxidation state and coordination of Fe in minerals: An Fe K-XANES spectroscopic study. *Am Mineral* 2001, **86**(5-6)**:** 714-730.

54. Bai L, Hsu C-S, Alexander DTL, Chen HM, Hu X. A Cobalt–Iron Double-Atom Catalyst for the Oxygen Evolution Reaction. *J Am Chem Soc* 2019, **141**(36)**:** 14190-14199.

55. Roy C, Sebok B, Scott SB, Fiordaliso EM, Sørensen JE, Bodin A*, et al.* Impact of nanoparticle size and lattice oxygen on water oxidation on $NiFeO_xH_y$. *Nat Catal* 2018, **1**(11)**:** 820-829.

Supplementary References

56. Ou Y, Twight LP, Samanta B, Liu L, Biswas S, Fehrs JL*, et al.* Cooperative Fe sites on transition metal (oxy)hydroxides drive high oxygen evolution activity in base. *Nat Commun* 2023, **14**(1)**:** 7688.

57. Liu L, Corma A. Identification of the active sites in supported subnanometric metal catalysts. *Nat Catal* 2021, **4**(6)**:** 453-456.

58. Finzel J, Sanroman Gutierrez KM, Hoffman AS, Resasco J, Christopher P, Bare SR. Limits of Detection for EXAFS Characterization of Heterogeneous Single-Atom Catalysts. *ACS Catal* 2023**:** 6462-6473.

59. González-Flores D, Klingan K, Chernev P, Loos S, Mohammadi MR, Pasquini C*, et al.* Nickel-iron catalysts for electrochemical water oxidation – redox synergism investigated by in situ X-ray spectroscopy with millisecond time resolution. *Sustainable Energy & Fuels* 2018, **2**(9)**:** 1986-1994.

60. Rohde J-U, Torelli S, Shan X, Lim MH, Klinker EJ, Kaizer J*, et al.* Structural Insights into Nonheme Alkylperoxoiron(III) and Oxoiron(IV) Intermediates by X-ray Absorption Spectroscopy. *J Am Chem Soc* 2004, **126**(51)**:** 16750-16761.

61. McDonald AR, Guo Y, Vu VV, Bominaar EL, Münck E, Que L. A mononuclear carboxylate-rich oxoiron(iv) complex: a structural and functional mimic of TauD intermediate 'J'. *Chem Sci* 2012, **3**(5)**:** 1680-1693.

62. Riggs-Gelasco PJ, Price JC, Guyer RB, Brehm JH, Barr EW, Bollinger JM, Jr.*, et al.* EXAFS Spectroscopic Evidence for an Fe═O Unit in the Fe(IV) Intermediate Observed during Oxygen Activation by Taurine:α-Ketoglutarate Dioxygenase. *J Am Chem Soc* 2004, **126**(26)**:** 8108-8109.

63. Olimov K, Falk M, Buse K, Woike T, Hormes J, Modrow H. X-ray absorption near edge spectroscopy investigations of valency and lattice occupation site of Fe in highly iron-doped lithium niobate crystals. *J Phys: Condens Matter* 2006, **18**(22)**:** 5135.

64. Trześniewski BJ, Diaz-Morales O, Vermaas DA, Longo A, Bras W, Koper MTM*, et al.* In Situ Observation of Active Oxygen Species in Fe-Containing Ni-Based Oxygen Evolution Catalysts: The Effect of pH on Electrochemical Activity. *J Am Chem Soc* 2015, **137**(48)**:** 15112-15121.

65. Takashima T, Ishikawa K, Irie H. Detection of Intermediate Species in Oxygen Evolution on Hematite Electrodes Using Spectroelectrochemical Measurements. *J Phys Chem C* 2016, **120**(43)**:** 24827-24834.

66. Chen JYC, Dang L, Liang H, Bi W, Gerken JB, Jin S*, et al.* Operando Analysis of NiFe and Fe Oxyhydroxide Electrocatalysts for Water Oxidation: Detection of Fe4+ by Mössbauer Spectroscopy. *J Am Chem Soc* 2015, **137**(48)**:** 15090-15093.

67. Mehmood R, Fan W, Hu X, Li J, Liu P, Zhang Y*, et al.* Confirming High-Valent Iron as Highly Active Species of Water Oxidation on the Fe, V-Coupled Bimetallic Electrocatalyst: In Situ Analysis of X-ray Absorption and Mössbauer Spectroscopy. *J Am Chem Soc* 2023, **145**(22)**:** 12206-12213.

68. Menil F. Systematic trends of the 57Fe Mössbauer isomer shifts in (FeOn) and (FeFn) polyhedra. Evidence of a new correlation between the isomer shift and the inductive effect of the competing bond TX (→

Fe)(where X is O or F and T any element with a formal positive charge). *J Phys Chem Solids* 1985, **46**(7): 763-789.

69. Garcia AC, Touzalin T, Nieuwland C, Perini N, Koper MTM. Enhancement of Oxygen Evolution Activity of Nickel Oxyhydroxide by Electrolyte Alkali Cations. *Angew Chem Int Ed* 2019, **58**(37): 12999-13003.

70. Goyal A, Louisia S, Moerland P, Koper MTM. Cooperative Effect of Cations and Catalyst Structure in Tuning Alkaline Hydrogen Evolution on Pt Electrodes. *J Am Chem Soc* 2024, **146**(11): 7305-7312.

71. Monteiro MCO, Goyal A, Moerland P, Koper MTM. Understanding Cation Trends for Hydrogen Evolution on Platinum and Gold Electrodes in Alkaline Media. *ACS Catal* 2021, **11**(23): 14328-14335.

72. Weber DJ, Janssen M, Oezaslan M. Effect of Monovalent Cations on the HOR/HER Activity for Pt in Alkaline Environment. *J Electrochem Soc* 2019, **166**(2): F66.

73. Liu Y, McCrory CCL. Modulating the mechanism of electrocatalytic CO_2 reduction by cobalt phthalocyanine through polymer coordination and encapsulation. *Nat Commun* 2019, **10**(1): 1683.

74. Quinn DM. Acetylcholinesterase: enzyme structure, reaction dynamics, and virtual transition states. *Chem Rev* 1987, **87**(5): 955-979.

75. Gandour R. *Transition states of biochemical processes*. Springer Science & Business Media, 2013.

76. Chen Z, Concepcion JJ, Hu X, Yang W, Hoertz PG, Meyer TJ. Concerted O atom–proton transfer in the O–O bond forming step in water oxidation. *Proc Natl Acad Sci USA* 2010, **107**(16): 7225-7229.

77. Agmon N. The Grotthuss mechanism. *Chem Phys Lett* 1995, **244**(5): 456-462.

78. Marx D, Chandra A, Tuckerman ME. Aqueous Basic Solutions: Hydroxide Solvation, Structural Diffusion, and Comparison to the Hydrated Proton. *Chem Rev* 2010, **110**(4): 2174-2216.

79. Roberts ST, Ramasesha K, Petersen PB, Mandal A, Tokmakoff A. Proton Transfer in Concentrated Aqueous Hydroxide Visualized Using Ultrafast Infrared Spectroscopy. *J Phys Chem A,* 2011, **115**(16): 3957-3972.

80. Govind Rajan A, Carter EA. Microkinetic model for pH- and potential-dependent oxygen evolution during water splitting on Fe-doped β-NiOOH. *Energy Environ Sci* 2020, **13**(12): 4962-4976.

81. Li J, Noll BC, Schulz CE, Scheidt WR. Comparison of Cyanide and Carbon Monoxide as Ligands in Iron(II) Porphyrinates. *Angew Chem Int Ed* 2009, **48**(27): 5010-5013.

82. Ogata H, Mizoguchi Y, Mizuno N, Miki K, Adachi S-i, Yasuoka N*, et al.* Structural Studies of the Carbon Monoxide Complex of [NiFe]hydrogenase from Desulfovibrio vulgaris Miyazaki F: Suggestion for the Initial Activation Site for Dihydrogen. *J Am Chem Soc* 2002, **124**(39): 11628-11635.

83. Sheridan MV, Sherman BD, Fang Z, Wee K-R, Coggins MK, Meyer TJ. Electron Transfer Mediator Effects in the Oxidative Activation of a Ruthenium Dicarboxylate Water Oxidation Catalyst. *ACS Catal* 2015, **5**(7):

4404-4409.

84. Chen Z, Concepcion JJ, Hu X, Yang W, Hoertz PG, Meyer TJ. Concerted O atom-proton transfer in the O-O bond forming step in water oxidation. *Proc Natl Acad Sci USA* 2010, **107**(16)**:** 7225.

85. Chen Z, Vannucci AK, Concepcion JJ, Jurss JW, Meyer TJ. Proton-coupled electron transfer at modified electrodes by multiple pathways. *Proc Natl Acad Sci USA* 2011, **108**(52)**:** E1461.

86. Takashima T, Ishikawa K, Irie H. Efficient oxygen evolution on hematite at neutral pH enabled by proton-coupled electron transfer. *Chem Commun* 2016, **52**(97)**:** 14015-14018.

87. Boudart M, Djéga-Mariadassou G. *Kinetics of heterogeneous catalytic reactions*. Princeton University Press, 2014.

88. Goldsmith ZK, Lam YC, Soudackov AV, Hammes-Schiffer S. Proton Discharge on a Gold Electrode from Triethylammonium in Acetonitrile: Theoretical Modeling of Potential-Dependent Kinetic Isotope Effects. *J Am Chem Soc* 2019, **141**(2)**:** 1084-1090.

89. Fletcher S. Tafel slopes from first principles. *J Solid State Electrochem* 2009, **13**(4)**:** 537-549.

90. Marcus RA. ON THE THEORY OF OXIDATION—REDUCTION REACTIONS INVOLVING ELECTRON TRANSFER. V. COMPARISON AND PROPERTIES OF ELECTROCHEMICAL AND CHEMICAL RATE CONSTANTS1. *J Phys Chem* 1963, **67**(4)**:** 853-857.

91. Shinagawa T, Garcia-Esparza AT, Takanabe K. Insight on Tafel slopes from a microkinetic analysis of aqueous electrocatalysis for energy conversion. *Sci Rep* 2015, **5**(1)**:** 13801.

92. van der Heijden O, Park S, Eggebeen JJJ, Koper MTM. Non-Kinetic Effects Convolute Activity and Tafel Analysis for the Alkaline Oxygen Evolution Reaction on NiFeOOH Electrocatalysts. *Angew Chem Int Ed* 2023, **62**(7)**:** e202216477.

93. Chung DY, Lopes PP, Farinazzo Bergamo Dias Martins P, He H, Kawaguchi T, Zapol P*, et al.* Dynamic stability of active sites in hydr(oxy)oxides for the oxygen evolution reaction. *Nat Energy* 2020, **5**(3)**:** 222-230.

94. Lopes PP, Chung DY, Rui X, Zheng H, He H, Farinazzo Bergamo Dias Martins P*, et al.* Dynamically Stable Active Sites from Surface Evolution of Perovskite Materials during the Oxygen Evolution Reaction. *J Am Chem Soc* 2021, **143**(7)**:** 2741-2750.

95. Ravel B, Newville M. ATHENA, ARTEMIS, HEPHAESTUS: data analysis for X-ray absorption spectroscopy using IFEFFIT. *J Synchrotron Rad* 2005, **12**(4)**:** 537-541.

96. Huang Z-F, Song J, Du Y, Xi S, Dou S, Nsanzimana JMV*, et al.* Chemical and structural origin of lattice oxygen oxidation in Co–Zn oxyhydroxide oxygen evolution electrocatalysts. *Nat Energy* 2019, **4**(4)**:** 329-338.

97. Hao Y, Li Y, Wu J, Meng L, Wang J, Jia C*, et al.* Recognition of Surface Oxygen Intermediates on NiFe

Oxyhydroxide Oxygen-Evolving Catalysts by Homogeneous Oxidation Reactivity. *J Am Chem Soc* 2021, **143**(3)**:** 1493-1502.

98. Tao HB, Xu Y, Huang X, Chen J, Pei L, Zhang J*, et al.* A General Method to Probe Oxygen Evolution Intermediates at Operating Conditions. *Joule* 2019, **3**(6)**:** 1498-1509.

99. Li W, Li F, Zhao Y, Liu C, Li Y, Yang H*, et al.* Promotion of the oxygen evolution performance of Ni-Fe layered hydroxides via the introduction of a proton-transfer mediator anion. *Sci China Chem* 2022, **65**(2)**:** 382-390.

100. Ahn HS, Bard AJ. Surface Interrogation Scanning Electrochemical Microscopy of $Ni_{1-x}Fe_xOOH$ (0 < x < 0.27) Oxygen Evolving Catalyst: Kinetics of the "fast" Iron Sites. *J Am Chem Soc* 2016, **138**(1)**:** 313-318.

101. Trześniewski BJ, Diaz-Morales O, Vermaas DA, Longo A, Bras W, Koper MTM*, et al.* In Situ Observation of Active Oxygen Species in Fe-Containing Ni-Based Oxygen Evolution Catalysts: The Effect of pH on Electrochemical Activity. *J Am Chem Soc* 2015, **137**(48)**:** 15112-15121.

102. Khan MMT, Mirza SA, Bajaj HC. Oxidation of triethylamine by molecular oxygen catalyzed by Ru(III)-ion. *React Kinet Catal Lett* 1987, **33**(1)**:** 67-74.

103. Khan MMT, Chatterjee D, Sanal KS, Merchant RR, Bhatt KN. Kinetics and mechanism of oxidation of diethylamine and triethylamine by $[Ru^{V}=O(EDTA)]^{-}$ in aqueous medium. *J Mol Catal* 1991, **67**(3)**:** 317-322.

104. Brown AP, Anson FC. Cyclic and differential pulse voltammetric behavior of reactants confined to the electrode surface. *Anal Chem* 1977, **49**(11)**:** 1589-1595.

105. Laviron E. Surface linear potential sweep voltammetry: Equation of the peaks for a reversible reaction when interactions between the adsorbed molecules are taken into account. *J Electroanal Chem Interf Electrochem* 1974, **52**(3)**:** 395-402.

106. Liu T, Zhan S, Shen N, Wang L, Szabó Z, Yang H*, et al.* Bioinspired Active Site with a Coordination-Adaptive Organosulfonate Ligand for Catalytic Water Oxidation at Neutral pH. *J Am Chem Soc* 2023, **145**(21)**:** 11818-11828.

107. Grimaud A, Diaz-Morales O, Han B, Hong WT, Lee Y-L, Giordano L*, et al.* Activating lattice oxygen redox reactions in metal oxides to catalyse oxygen evolution. *Nat Chem* 2017, **9:** 457.

108. Huynh M, Bediako DK, Nocera DG. A functionally stable manganese oxide oxygen evolution catalyst in acid. *J Am Chem Soc* 2014, **136**(16)**:** 6002-6010.

109. Stoerzinger KA, Rao RR, Wang XR, Hong WT, Rouleau CM, Shao-Horn Y. The Role of Ru Redox in pH-Dependent Oxygen Evolution on Rutile Ruthenium Dioxide Surfaces. *Chem* 2017, **2**(5)**:** 668-675.

110. Pan Y, Xu X, Zhong Y, Ge L, Chen Y, Veder J-PM*, et al.* Direct evidence of boosted oxygen evolution over perovskite by enhanced lattice oxygen participation. *Nat Commun* 2020, **11**(1)**:** 2002.

Supplementary References

111. Matheu R, Neudeck S, Meyer F, Sala X, Llobet A. Foot of the Wave Analysis for Mechanistic Elucidation and Benchmarking Applications in Molecular Water Oxidation Catalysis. *ChemSusChem* 2016, **9**(23)**:** 3361-3369.

112. Yang C, Batuk M, Jacquet Q, Rousse G, Yin W, Zhang L*, et al.* Revealing pH-Dependent Activities and Surface Instabilities for Ni-Based Electrocatalysts during the Oxygen Evolution Reaction. *ACS Energy Lett* 2018, **3**(12)**:** 2884-2890.

113. Lyons MEG, Doyle RL, Brandon MP. Redox switching and oxygen evolution at oxidized metal and metal oxide electrodes: iron in base. *Phys Chem Chem Phys* 2011, **13**(48)**:** 21530-21551.

114. Bockris JOM. Kinetics of Activation Controlled Consecutive Electrochemical Reactions: Anodic Evolution of Oxygen. *J Chem Phys* 1956, **24**(4)**:** 817.

115. Doyle RL, Godwin IJ, Brandon MP, Lyons MEG. Redox and electrochemical water splitting catalytic properties of hydrated metal oxide modified electrodes. *Phys Chem Chem Phys* 2013, **15**(33)**:** 13737-13783.

116. Shinagawa T, Garcia-Esparza AT, Takanabe K. Insight on Tafel slopes from a microkinetic analysis of aqueous electrocatalysis for energy conversion. *Sci Rep* 2015, **5:** 13801.

117. Laviron E. Adsorption, autoinhibition and autocatalysis in polarography and in linear potential sweep voltammetry. *J Electroanal Chem Interf Electrochem* 1974, **52**(3)**:** 355-393.

118. Koper MTM. Volcano Activity Relationships for Proton-Coupled Electron Transfer Reactions in Electrocatalysis. *Top Catal* 2015, **58**(18)**:** 1153-1158.

119. Giordano L, Han B, Risch M, Hong WT, Rao RR, Stoerzinger KA*, et al.* pH dependence of OER activity of oxides: Current and future perspectives. *Catal Today* 2016, **262:** 2-10.

120. Bonin J, Costentin C, Robert M, Routier M, Savéant J-M. Proton-Coupled Electron Transfers: pH-Dependent Driving Forces? Fundamentals and Artifacts. *J Am Chem Soc* 2013, **135**(38)**:** 14359-14366.

121. Fukuzumi S, Kobayashi T, Suenobu T. Unusually Large Tunneling Effect on Highly Efficient Generation of Hydrogen and Hydrogen Isotopes in pH-Selective Decomposition of Formic Acid Catalyzed by a Heterodinuclear Iridium−Ruthenium Complex in Water. *J Am Chem Soc* 2010, **132**(5)**:** 1496-1497.

122. Joo J, Uchida T, Cuesta A, Koper MTM, Osawa M. Importance of Acid–Base Equilibrium in Electrocatalytic Oxidation of Formic Acid on Platinum. *J Am Chem Soc* 2013, **135**(27)**:** 9991-9994.

123. Brimaud S, Solla-Gullón J, Weber I, Feliu JM, Behm RJ. Formic Acid Electrooxidation on Noble-Metal Electrodes: Role and Mechanistic Implications of pH, Surface Structure, and Anion Adsorption. *ChemElectroChem* 2014, **1**(6)**:** 1075-1083.

124. Schowen RL. The use of solvent isotope effects in the pursuit of enzyme mechanisms. *J Labelled Compd Radiopharm* 2007, **50**(11-12)**:** 1052-1062.

125. Venkatasubban KS, Schowen RL. The proton inventory technique. *CRC Crit Rev Biochem* 1984, **17**(1)**:** 1-44.

Supplementary References

126. Han S-G, Zhang M, Fu Z-H, Zheng L, Ma D-D, Wu X-T*, et al.* Enzyme-Inspired Microenvironment Engineering of a Single-Molecular Heterojunction for Promoting Concerted Electrochemical CO2 Reduction. *Adv Mater* 2022, **34**(34)**:** 2202830.

127. Barbara Schowen K, Schowen RL. [29] Solvent isotope effects on enzyme systems. In: Purich DL (ed). *Methods in Enzymology*, vol. 87. Academic Press, 1982, pp 551-606.

128. Kresge AJ. Solvent isotope effect in H2O-D2O mixtures. *Pure Appl Chem* 1964, **8**(3-4)**:** 243-258.

129. More O'Ferrall RA, Koeppl GW, Kresge AJ. Solvent isotope effects upon proton transfer from the hydronium ion. *J Am Chem Soc* 1971, **93**(1)**:** 9-20.

130. Pasquini C, Zaharieva I, González-Flores D, Chernev P, Mohammadi MR, Guidoni L*, et al.* H/D Isotope Effects Reveal Factors Controlling Catalytic Activity in Co-Based Oxides for Water Oxidation. *J Am Chem Soc* 2019, **141**(7)**:** 2938-2948.

131. Scott M, Rodrigues GLS, Li X, Delcey MG. Variational Pair-Density Functional Theory: Dealing with Strong Correlation at the Protein Scale. *Journal of Chemical Theory and Computation* 2024, **20**(6)**:** 2423-2432.

132. Feng R, Zhang IY, Xu X. A cross-entropy corrected hybrid multiconfiguration pair-density functional theory for complex molecular systems. *Nat Commun* 2025, **16**(1)**:** 235.

133. Zhang H-T, Guo Y-H, Xiao Y, Du H-Y, Zhang M-T. Heterobimetallic NiFe Cooperative Molecular Water Oxidation Catalyst. *Angew Chem Int Ed* 2023, **62**(18)**:** e202218859.

134. Zhang E, Hirao H. Exploring the Bonding Nature of Iron(IV)-Oxo Species through Valence Bond Calculations and Electron Density Analysis. *J Phys Chem A,* 2024, **128**(34)**:** 7167-7176.

135. Wertz DH. Relationship between the gas-phase entropies of molecules and their entropies of solvation in water and 1-octanol. *J Am Chem Soc* 1980, **102**(16)**:** 5316-5322.
