# Supplementary material for: Metal-hydroxyls mediate intramolecular proton transfer in heterogeneous O–O bond formation
Source: Nat Chem. 2025 Nov 14;18(2):335–44. doi: 10.1038/s41557-025-01993-8 (PMC12872446; doi:10.1038/s41557-025-01993-8)
Supplement: Supplementary file 2 — The atomic coordinates of key intermediates. [file 41557_2025_1993_MOESM2_ESM.pdf]

Ni-COF

Complex 1

47

C	-6.386000	-6.611000	0.784000
C	-6.359000	-8.028000	0.668000
C	-5.183000	-8.691000	0.411000
C	-5.242000	-5.861000	0.647000
C	-3.976000	-7.957000	0.266000
C	-4.014000	-6.523000	0.391000
N	-2.864000	-5.811000	0.281000
N	-2.826000	-8.620000	0.018000
C	-1.734000	-6.482000	0.072000
C	-1.716000	-7.904000	-0.080000
C	0.759000	-6.379000	-0.193000
C	-0.491000	-5.720000	0.036000
N	-0.563000	-4.410000	0.251000
C	0.594000	-3.701000	0.265000
C	1.842000	-4.377000	0.027000
C	3.044000	-3.621000	0.051000
C	0.570000	-2.309000	0.537000
C	2.998000	-2.272000	0.311000
C	1.759000	-1.619000	0.558000
C	-0.434000	-8.578000	-0.346000
C	0.769000	-7.835000	-0.407000
C	1.925000	-9.748000	-0.829000
C	0.730000	-10.487000	-0.766000
Ni	-2.592000	-3.645000	0.255000
O1	-2.415000	-1.704000	0.691000
N	-0.441000	-9.912000	-0.526000
N	1.949000	-8.434000	-0.650000
N	1.900000	-5.707000	-0.203000
O1	-2.860000	-3.404000	-1.667000
O	-2.598000	-3.454000	2.487000
O	-4.722000	-2.894000	0.016000
H	-5.133000	-9.771000	0.315000
H	-5.241000	-4.776000	0.711000
H	3.976000	-4.145000	-0.135000
H	-4.293000	-2.081000	0.360000
H	-2.960000	-4.238000	-2.145000
H	-2.289000	-1.162000	-0.097000
H	3.917000	-1.694000	0.333000
H	-7.282000	-8.589000	0.784000
H	-7.330000	-6.113000	0.984000
H	0.732000	-11.565000	-0.914000
H	2.875000	-10.240000	-1.029000
H	1.754000	-0.553000	0.767000
H	-0.406000	-1.849000	0.715000
H	-1.717000	-3.683000	2.810000
H	-2.535000	-2.500000	2.236000
H	-4.315000	-2.981000	-0.901000

Complex 2

46

C	-6.358000	-6.610000	0.813000

C	-6.338000	-8.019000	0.671000
C	-5.169000	-8.682000	0.393000
C	-5.214000	-5.864000	0.669000
H	-5.123000	-9.761000	0.286000
H	-5.203000	-4.782000	0.783000
C	-3.964000	-7.953000	0.246000
C	-3.998000	-6.523000	0.380000
N	-2.859000	-5.810000	0.246000
N	-2.820000	-8.620000	-0.007000
C	-1.730000	-6.479000	0.038000
C	-1.711000	-7.901000	-0.100000
C	0.752000	-6.377000	-0.197000
C	-0.499000	-5.717000	-0.001000
N	-0.574000	-4.402000	0.192000
C	0.582000	-3.701000	0.231000
C	1.832000	-4.383000	0.027000
C	3.031000	-3.634000	0.071000
C	0.559000	-2.312000	0.492000
C	2.986000	-2.285000	0.320000
C	1.750000	-1.629000	0.535000
C	-0.434000	-8.572000	-0.346000
C	0.766000	-7.828000	-0.396000
H	3.963000	-4.166000	-0.089000
C	1.926000	-9.738000	-0.781000
C	0.735000	-10.476000	-0.730000
Ni	-2.581000	-3.605000	0.327000
H	-4.099000	-1.972000	0.418000
H	-2.759000	-4.260000	-2.030000
O1	-2.398000	-1.615000	0.520000
H	-2.259000	-1.330000	-0.392000
H	3.908000	-1.713000	0.359000
H	-7.261000	-8.578000	0.790000
H	-7.296000	-6.113000	1.045000
H	0.740000	-11.556000	-0.869000
H	2.881000	-10.230000	-0.961000
N	-0.440000	-9.906000	-0.515000
N	1.951000	-8.424000	-0.616000
N	1.896000	-5.711000	-0.193000
H	1.745000	-0.562000	0.741000
H	-0.416000	-1.839000	0.654000
O1	-2.814000	-3.422000	-1.559000
O1	-2.681000	-3.758000	2.197000
H	-2.767000	-2.838000	2.492000
O	-4.704000	-2.735000	0.204000
H	-4.565000	-2.830000	-0.755000

Complex 3
45

C	-6.366000	-6.467000	0.522000
C	-6.361000	-7.880000	0.428000
C	-5.189000	-8.571000	0.244000
C	-5.203000	-5.741000	0.437000
H	-5.156000	-9.652000	0.166000
H	-5.192000	-4.650000	0.501000
C	-3.964000	-7.868000	0.152000

C	-3.987000	-6.432000	0.255000
N	-2.820000	-5.753000	0.174000
N	-2.822000	-8.560000	-0.032000
C	-1.694000	-6.446000	0.031000
C	-1.696000	-7.865000	-0.093000
C	0.787000	-6.360000	-0.201000
C	-0.459000	-5.701000	0.008000
N	-0.542000	-4.390000	0.190000
C	0.597000	-3.668000	0.147000
C	1.847000	-4.344000	-0.088000
C	3.034000	-3.576000	-0.141000
C	0.564000	-2.268000	0.329000
C	2.976000	-2.215000	0.031000
C	1.741000	-1.563000	0.268000
C	-0.417000	-8.550000	-0.296000
C	0.791000	-7.815000	-0.357000
H	3.967000	-4.100000	-0.319000
C	1.939000	-9.744000	-0.682000
C	0.742000	-10.471000	-0.618000
Ni	-2.553000	-3.748000	0.534000
H	-4.557000	-2.069000	0.678000
H	-2.916000	-4.084000	-1.761000
O2	-2.461000	-2.052000	1.021000
H	3.888000	-1.627000	-0.011000
H	-7.300000	-8.421000	0.501000
H	-7.308000	-5.946000	0.665000
H	0.740000	-11.555000	-0.724000
H	2.891000	-10.247000	-0.841000
N	-0.431000	-9.887000	-0.428000
N	1.972000	-8.427000	-0.553000
N	1.923000	-5.681000	-0.250000
H	1.728000	-0.486000	0.404000
H	-0.403000	-1.806000	0.525000
O1	-2.786000	-3.261000	-1.275000
O1	-2.613000	-4.228000	2.318000
H	-2.444000	-3.420000	2.819000
O	-5.073000	-2.628000	0.080000
H	-4.447000	-2.712000	-0.671000

Complex 4
48

C	-6.365000	-6.501000	0.433000
C	-6.345000	-7.891000	0.163000
C	-5.163000	-8.545000	-0.079000
C	-5.208000	-5.761000	0.456000
H	-5.118000	-9.610000	-0.282000
H	-5.211000	-4.689000	0.671000
C	-3.943000	-7.828000	-0.060000
C	-3.982000	-6.413000	0.204000
N	-2.823000	-5.720000	0.215000
N	-2.789000	-8.489000	-0.282000
C	-1.687000	-6.382000	0.017000
C	-1.669000	-7.782000	-0.238000
C	0.798000	-6.250000	-0.127000
C	-0.463000	-5.621000	0.072000

N	-0.573000	-4.318000	0.309000
C	0.557000	-3.577000	0.346000
C	1.827000	-4.235000	0.158000
C	3.008000	-3.458000	0.210000
C	0.496000	-2.180000	0.547000
C	2.925000	-2.103000	0.416000
C	1.670000	-1.467000	0.574000
C	-0.376000	-8.436000	-0.452000
C	0.826000	-7.690000	-0.395000
H	3.954000	-3.970000	0.074000
C	2.009000	-9.574000	-0.831000
C	0.818000	-10.313000	-0.888000
Ni	-2.568000	-3.775000	0.833000
H	-4.557000	-2.208000	1.317000
H	-2.632000	-3.552000	-1.556000
O2	-2.500000	-2.192000	1.623000
H	3.833000	-1.506000	0.450000
H	-7.280000	-8.443000	0.152000
H	-7.313000	-6.009000	0.630000
H	0.834000	-11.383000	-1.092000
H	2.973000	-10.055000	-0.987000
N	-0.368000	-9.756000	-0.702000
N	2.021000	-8.272000	-0.588000
N	1.926000	-5.560000	-0.072000
H	1.632000	-0.391000	0.715000
H	-0.475000	-1.695000	0.642000
O1	-2.991000	-3.007000	-0.849000
O1	-2.524000	-4.511000	2.520000
H	-2.250000	-3.790000	3.102000
O	-5.205000	-2.601000	0.714000
H	-4.701000	-2.622000	-0.119000
O	-2.102000	-0.495000	-0.257000
H	-2.692000	-0.373000	0.494000
H	-2.413000	-1.348000	-0.634000

Complex TS

48

C	-6.230000	-6.123000	-0.064000
C	-6.221000	-7.504000	-0.374000
C	-5.040000	-8.174000	-0.582000
C	-5.059000	-5.415000	0.045000
H	-5.007000	-9.231000	-0.826000
H	-5.044000	-4.357000	0.287000
C	-3.811000	-7.480000	-0.491000
C	-3.831000	-6.080000	-0.166000
N	-2.665000	-5.401000	-0.092000
N	-2.660000	-8.147000	-0.720000
C	-1.538000	-6.060000	-0.349000
C	-1.535000	-7.451000	-0.658000
C	0.930000	-5.919000	-0.632000
C	-0.314000	-5.293000	-0.331000
N	-0.410000	-3.989000	-0.053000
C	0.736000	-3.261000	-0.093000
C	1.978000	-3.906000	-0.425000
C	3.158000	-3.127000	-0.488000

C	0.708000	-1.874000	0.177000
C	3.103000	-1.779000	-0.235000
C	1.878000	-1.156000	0.101000
C	-0.252000	-8.107000	-0.912000
C	0.949000	-7.359000	-0.901000
H	4.083000	-3.635000	-0.741000
C	2.119000	-9.243000	-1.372000
C	0.928000	-9.984000	-1.384000
Ni	-2.397000	-3.436000	0.584000
H	-4.735000	-2.363000	1.173000
H	-2.579000	-2.909000	-1.903000
O2	-2.119000	-1.717000	1.292000
H	4.009000	-1.182000	-0.286000
H	-7.165000	-8.037000	-0.450000
H	-7.177000	-5.616000	0.088000
H	0.938000	-11.055000	-1.581000
H	3.078000	-9.724000	-1.558000
N	-0.252000	-9.428000	-1.159000
N	2.138000	-7.940000	-1.135000
N	2.060000	-5.229000	-0.680000
H	1.862000	-0.090000	0.308000
H	-0.242000	-1.433000	0.474000
O1	-3.004000	-2.570000	-1.111000
O1	-3.472000	-3.735000	2.074000
H	-3.325000	-2.961000	2.637000
O	-5.083000	-1.770000	0.486000
H	-4.680000	-2.142000	-0.320000
O	-2.687000	-0.437000	0.133000
H	-3.609000	-0.545000	0.468000
H	-2.693000	-1.185000	-0.589000

Complex 5
48

C	-6.311000	-5.909000	-0.223000
C	-6.293000	-7.293000	-0.513000
C	-5.108000	-7.946000	-0.747000
C	-5.147000	-5.183000	-0.166000
H	-5.063000	-9.007000	-0.972000
H	-5.143000	-4.127000	0.080000
C	-3.887000	-7.233000	-0.703000
C	-3.914000	-5.827000	-0.406000
N	-2.756000	-5.124000	-0.384000
N	-2.736000	-7.892000	-0.957000
C	-1.627000	-5.788000	-0.632000
C	-1.618000	-7.184000	-0.923000
C	0.840000	-5.648000	-0.940000
C	-0.401000	-5.024000	-0.616000
N	-0.483000	-3.728000	-0.289000
C	0.673000	-3.009000	-0.311000
C	1.905000	-3.651000	-0.681000
C	3.092000	-2.883000	-0.722000
C	0.663000	-1.637000	0.024000
C	3.056000	-1.547000	-0.407000
C	1.841000	-0.930000	-0.029000
C	-0.339000	-7.836000	-1.208000

C	0.858000	-7.084000	-1.226000
H	4.009000	-3.391000	-1.004000
C	2.023000	-8.960000	-1.737000
C	0.837000	-9.707000	-1.716000
Ni	-2.444000	-3.144000	0.333000
H	-4.822000	-2.255000	0.959000
H	-2.343000	-2.386000	-2.210000
O1	-2.125000	-1.286000	1.004000
H	3.969000	-0.959000	-0.437000
H	-7.230000	-7.842000	-0.550000
H	-7.259000	-5.414000	-0.037000
H	0.847000	-10.778000	-1.914000
H	2.979000	-9.435000	-1.955000
N	-0.340000	-9.157000	-1.457000
N	2.043000	-7.658000	-1.496000
N	1.974000	-4.965000	-0.979000
H	1.839000	0.125000	0.232000
H	-0.286000	-1.201000	0.353000
O	-2.999000	-2.259000	-1.518000
O1	-3.626000	-3.384000	1.778000
H	-3.402000	-2.621000	2.332000
O	-5.245000	-1.589000	0.371000
H	-5.074000	-1.934000	-0.513000
O	-2.783000	-0.314000	0.117000
H	-3.719000	-0.426000	0.397000
H	-2.863000	-1.335000	-1.146000

NiFe-COF

Complex 1
60
PDB File

C	-6.410000	-6.598000	1.037000
C	-6.419000	-7.969000	0.682000
C	-5.264000	-8.581000	0.265000
C	-5.269000	-5.834000	0.932000
H	-5.221000	-9.637000	0.018000
H	-5.264000	-4.792000	1.235000
C	-4.061000	-7.837000	0.176000
C	-4.071000	-6.422000	0.473000
N	-2.920000	-5.704000	0.335000
N	-2.932000	-8.481000	-0.156000
C	-1.793000	-6.373000	0.044000
C	-1.808000	-7.782000	-0.186000
C	0.679000	-6.325000	-0.255000
C	-0.549000	-5.637000	-0.018000
N	-0.579000	-4.306000	0.164000
C	0.600000	-3.624000	0.079000
C	1.831000	-4.360000	-0.111000
C	3.065000	-3.664000	-0.154000
C	0.654000	-2.216000	0.148000
C	3.083000	-2.296000	-0.053000
C	1.871000	-1.576000	0.081000
C	-0.556000	-8.480000	-0.460000

C	0.666000	-7.768000	-0.479000
H	3.968000	-4.250000	-0.283000
C	1.794000	-9.706000	-0.917000
C	0.565000	-10.422000	-0.903000
Ni	-2.566000	-3.618000	0.521000
H	-2.968000	-3.753000	-2.256000
H	4.024000	-1.758000	-0.092000
H	-7.339000	-8.537000	0.764000
H	-7.325000	-6.147000	1.408000
H	0.544000	-11.495000	-1.080000
H	2.736000	-10.220000	-1.100000
N	-0.566000	-9.797000	-0.676000
N	1.812000	-8.412000	-0.708000
N	1.842000	-5.692000	-0.270000
H	1.908000	-0.491000	0.126000
H	-0.273000	-1.658000	0.228000
O	-2.571000	-3.096000	-1.667000
H	-1.656000	-3.009000	-1.971000
O	-2.624000	-3.866000	2.650000
H	-3.082000	-4.677000	2.908000
H	-1.774000	-3.909000	3.109000
Fe	-4.157000	-0.934000	0.721000
O	-3.773000	1.168000	0.984000
O	-6.153000	-0.169000	0.398000
O	-4.765000	-0.915000	2.773000
H	-2.945000	1.654000	1.081000
H	-6.575000	-0.167000	-0.471000
H	-4.767000	-1.743000	3.268000
H	-3.506000	-1.426000	-1.733000
H	-3.671000	0.127000	-1.803000
O	-4.023000	-0.671000	-1.390000
H	-4.583000	-0.207000	3.403000
H	-4.473000	1.822000	0.863000
H	-6.858000	-0.203000	1.056000
O1	-4.483000	-2.828000	0.505000
H	-5.225000	-3.183000	0.001000
O1	-2.328000	-1.581000	0.891000
H	-1.805000	-1.345000	1.666000

Complex 2
58
NiIII-OHH2O-2O-FeIII-OH3H2O-ch2-m1-2H

C	-6.434000	-6.531000	1.065000
C	-6.402000	-7.935000	0.904000
C	-5.241000	-8.554000	0.524000
C	-5.328000	-5.750000	0.818000
H	-5.167000	-9.631000	0.408000
H	-5.367000	-4.673000	0.930000
C	-4.069000	-7.794000	0.289000
C	-4.119000	-6.351000	0.402000
N	-2.973000	-5.637000	0.182000
N	-2.937000	-8.460000	0.023000
C	-1.838000	-6.333000	-0.009000
C	-1.826000	-7.755000	-0.099000
C	0.632000	-6.271000	-0.302000

C	-0.610000	-5.593000	-0.103000
N	-0.683000	-4.257000	0.016000
C	0.475000	-3.546000	-0.119000
C	1.711000	-4.260000	-0.335000
C	2.926000	-3.538000	-0.421000
C	0.511000	-2.137000	-0.028000
C	2.928000	-2.169000	-0.330000
C	1.714000	-1.473000	-0.132000
C	-0.557000	-8.450000	-0.294000
C	0.646000	-7.730000	-0.389000
H	3.833000	-4.116000	-0.571000
C	1.779000	-9.675000	-0.645000
C	0.569000	-10.391000	-0.553000
Ni	-2.596000	-3.648000	0.297000
H	-2.443000	-3.151000	-2.462000
H	3.861000	-1.619000	-0.389000
H	-7.295000	-8.522000	1.096000
H	-7.355000	-6.055000	1.389000
H	0.552000	-11.478000	-0.624000
H	2.722000	-10.198000	-0.791000
N	-0.593000	-9.788000	-0.376000
N	1.824000	-8.353000	-0.563000
N	1.767000	-5.599000	-0.407000
H	1.733000	-0.391000	-0.048000
H	-0.405000	-1.605000	0.201000
O	-2.322000	-2.793000	-1.576000
H	-1.380000	-2.588000	-1.506000
O1	-2.807000	-3.838000	2.147000
H	-2.246000	-4.152000	2.870000
Fe	-4.103000	-1.089000	0.750000
O	-3.508000	1.021000	1.075000
O	-6.000000	-0.190000	0.624000
O1	-4.528000	-1.360000	2.447000
H	-2.842000	1.189000	1.756000
H	-6.564000	-0.190000	-0.161000
H	-4.133000	-2.193000	2.773000
H	-3.095000	-1.261000	-1.602000
H	-3.496000	0.241000	-1.326000
O	-3.787000	-0.673000	-1.215000
H	-4.289000	1.512000	1.363000
H	-6.527000	-0.502000	1.375000
O1	-4.428000	-2.996000	0.162000
H	-4.753000	-3.135000	-0.736000
O1	-2.271000	-1.784000	0.881000
H	-1.965000	-1.862000	1.798000

Complex 3
57
NiIII-OHH2O-2O-FeIII-OH3H2O-ch2-m1-2H

C	-6.434000	-6.531000	1.065000
C	-6.402000	-7.935000	0.904000
C	-5.241000	-8.554000	0.524000
C	-5.328000	-5.750000	0.818000
H	-5.167000	-9.631000	0.408000
H	-5.367000	-4.673000	0.930000

C	-4.069000	-7.794000	0.289000
C	-4.119000	-6.351000	0.402000
N	-2.973000	-5.637000	0.182000
N	-2.937000	-8.460000	0.023000
C	-1.838000	-6.333000	-0.009000
C	-1.826000	-7.755000	-0.099000
C	0.632000	-6.271000	-0.302000
C	-0.610000	-5.593000	-0.103000
N	-0.683000	-4.257000	0.016000
C	0.475000	-3.546000	-0.119000
C	1.711000	-4.260000	-0.335000
C	2.926000	-3.538000	-0.421000
C	0.511000	-2.137000	-0.028000
C	2.928000	-2.169000	-0.330000
C	1.714000	-1.473000	-0.132000
C	-0.557000	-8.450000	-0.294000
C	0.646000	-7.730000	-0.389000
H	3.833000	-4.116000	-0.571000
C	1.779000	-9.675000	-0.645000
C	0.569000	-10.391000	-0.553000
Ni	-2.596000	-3.648000	0.297000
H	-2.443000	-3.151000	-2.462000
H	3.861000	-1.619000	-0.389000
H	-7.295000	-8.522000	1.096000
H	-7.355000	-6.055000	1.389000
H	0.552000	-11.478000	-0.624000
H	2.722000	-10.198000	-0.791000
N	-0.593000	-9.788000	-0.376000
N	1.824000	-8.353000	-0.563000
N	1.767000	-5.599000	-0.407000
H	1.733000	-0.391000	-0.048000
H	-0.405000	-1.605000	0.201000
O	-2.322000	-2.793000	-1.576000
H	-1.380000	-2.588000	-1.506000
O1	-2.807000	-3.838000	2.147000
H	-2.246000	-4.152000	2.870000
Fe	-4.103000	-1.089000	0.750000
O	-3.508000	1.021000	1.075000
O	-6.000000	-0.190000	0.624000
O2	-4.528000	-1.360000	2.447000
H	-2.842000	1.189000	1.756000
H	-6.564000	-0.190000	-0.161000
H	-3.095000	-1.261000	-1.602000
H	-3.496000	0.241000	-1.326000
O	-3.787000	-0.673000	-1.215000
H	-4.289000	1.512000	1.363000
H	-6.527000	-0.502000	1.375000
O1	-4.428000	-2.996000	0.162000
H	-4.753000	-3.135000	-0.736000
O1	-2.271000	-1.784000	0.881000
H	-1.965000	-1.862000	1.798000

Complex 4
62
NiIII-OHH2O-2O-FeIV=O3H2O-ch1-m2-2H-OHscan

C	-6.446000	-6.548000	0.811000
C	-6.446000	-7.946000	0.577000
C	-5.286000	-8.587000	0.219000
C	-5.318000	-5.783000	0.654000
H	-5.245000	-9.652000	0.016000
H	-5.316000	-4.718000	0.855000
C	-4.083000	-7.848000	0.093000
C	-4.115000	-6.409000	0.270000
N	-2.967000	-5.700000	0.139000
N	-2.932000	-8.505000	-0.117000
C	-1.817000	-6.378000	-0.001000
C	-1.808000	-7.798000	-0.126000
C	0.678000	-6.321000	-0.180000
C	-0.564000	-5.641000	0.002000
N	-0.604000	-4.310000	0.145000
C	0.556000	-3.611000	-0.016000
C	1.791000	-4.322000	-0.229000
C	2.981000	-3.581000	-0.453000
C	0.561000	-2.193000	-0.054000
C	2.956000	-2.205000	-0.494000
C	1.738000	-1.515000	-0.298000
C	-0.537000	-8.497000	-0.247000
C	0.683000	-7.782000	-0.279000
H	3.894000	-4.147000	-0.598000
C	1.818000	-9.735000	-0.508000
C	0.597000	-10.449000	-0.470000
Ni	-2.574000	-3.667000	0.398000
H	-2.636000	-3.022000	-3.029000
H	3.860000	-1.643000	-0.708000
H	-7.362000	-8.508000	0.741000
H	-7.370000	-6.076000	1.127000
H	0.572000	-11.537000	-0.550000
H	2.762000	-10.264000	-0.616000
N	-0.551000	-9.835000	-0.342000
N	1.849000	-8.427000	-0.413000
N	1.827000	-5.664000	-0.275000
H	1.726000	-0.431000	-0.373000
H	-0.366000	-1.651000	0.112000
O	-2.399000	-2.669000	-2.156000
H	-1.474000	-2.395000	-2.266000
O1	-2.697000	-4.135000	2.253000
H	-2.037000	-4.641000	2.759000
Fe	-4.124000	-1.125000	0.872000
O	-3.691000	0.947000	1.239000
O	-6.090000	-0.461000	0.743000
O2	-4.367000	-1.381000	2.442000
H	-3.092000	1.120000	1.979000
H	-6.628000	-0.533000	-0.056000
H	-3.377000	-1.116000	-1.651000
H	-3.732000	0.326000	-1.185000
O	-4.013000	-0.593000	-1.110000
H	-4.524000	1.394000	1.456000
H	-6.575000	-0.884000	1.467000
O1	-4.467000	-2.935000	0.351000
H	-4.853000	-2.915000	-0.539000

O1 -2.304000 -1.697000 0.853000
H -2.061000 -1.707000 1.784000
O1 -3.181000 -2.325000 4.430000
H -2.557000 -3.007000 4.169000
O -5.936000 -3.330000 3.734000
H -6.263000 -4.018000 3.163000
H -5.006000 -3.516000 3.876000

Complex 5
62
NiIII-OHH2O-2O-FeIVOO-3H2O-ch1-m2-2H-OHscan
C -6.354000 -6.437000 1.112000
C -6.435000 -7.796000 0.717000
C -5.344000 -8.436000 0.179000
C -5.198000 -5.713000 0.937000
H -5.374000 -9.485000 -0.106000
H -5.119000 -4.672000 1.230000
C -4.123000 -7.736000 0.020000
C -4.063000 -6.342000 0.374000
N -2.898000 -5.665000 0.226000
N -3.038000 -8.404000 -0.431000
C -1.818000 -6.359000 -0.130000
C -1.890000 -7.741000 -0.470000
C 0.668000 -6.417000 -0.252000
C -0.539000 -5.685000 -0.043000
N -0.533000 -4.409000 0.346000
C 0.665000 -3.846000 0.628000
C 1.872000 -4.616000 0.481000
C 3.108000 -4.037000 0.857000
C 0.734000 -2.524000 1.122000
C 3.140000 -2.759000 1.362000
C 1.950000 -1.999000 1.491000
C -0.663000 -8.441000 -0.850000
C 0.587000 -7.797000 -0.734000
H 4.008000 -4.636000 0.748000
C 1.622000 -9.684000 -1.473000
C 0.378000 -10.322000 -1.593000
Ni -2.563000 -3.545000 0.276000
H -2.093000 -4.237000 -2.536000
H 4.086000 -2.320000 1.670000
H -7.367000 -8.337000 0.860000
H -7.221000 -5.965000 1.569000
H 0.315000 -11.348000 -1.951000
H 2.539000 -10.210000 -1.733000
N -0.759000 -9.713000 -1.281000
N 1.733000 -8.433000 -1.044000
N 1.854000 -5.881000 0.007000
H 2.004000 -0.990000 1.894000
H -0.188000 -1.955000 1.213000
O -2.138000 -3.448000 -1.974000
H -1.329000 -2.960000 -2.195000
H -1.591000 -3.814000 2.819000
Fe -4.514000 -1.344000 0.418000
O -4.472000 0.554000 1.263000
O -6.622000 -1.369000 0.455000

O -4.580000 -1.938000 2.104000
H -4.088000 0.232000 2.123000
H -6.953000 -2.125000 -0.058000
H -4.873000 -0.961000 -2.302000
H -3.604000 -0.250000 -1.796000
O -4.500000 -0.509000 -1.528000
H -5.355000 0.889000 1.497000
H -6.893000 -1.566000 1.369000
O1 -4.499000 -3.129000 -0.294000
H -4.404000 -3.057000 -1.256000
O1 -2.559000 -1.479000 0.333000
H -2.350000 -1.224000 1.251000
O -3.598000 -1.283000 2.897000
O -3.297000 -0.607000 5.682000
H -2.758000 0.178000 5.521000
H -3.573000 -0.867000 4.783000
O -2.469000 -3.638000 2.452000
H -2.740000 -2.748000 2.804000

Complex TS_{OH^-}
62
NiIII-OHH2O-2O-FeIV=O3H2O-ch1-m2-2H-OHscan
C -6.359000 -6.641000 0.985000
C -6.350000 -8.040000 0.765000
C -5.186000 -8.677000 0.412000
C -5.235000 -5.870000 0.819000
H -5.140000 -9.745000 0.220000
H -5.240000 -4.804000 1.010000
C -3.988000 -7.930000 0.276000
C -4.027000 -6.490000 0.439000
N -2.882000 -5.775000 0.296000
N -2.834000 -8.583000 0.069000
C -1.729000 -6.449000 0.159000
C -1.715000 -7.869000 0.049000
C 0.766000 -6.388000 -0.023000
C -0.478000 -5.709000 0.152000
N -0.519000 -4.379000 0.281000
C 0.642000 -3.681000 0.115000
C 1.876000 -4.396000 -0.091000
C 3.072000 -3.669000 -0.321000
C 0.655000 -2.265000 0.063000
C 3.053000 -2.293000 -0.375000
C 1.837000 -1.598000 -0.187000
C -0.442000 -8.564000 -0.066000
C 0.775000 -7.848000 -0.106000
H 3.979000 -4.247000 -0.455000
C 1.915000 -9.800000 -0.315000
C 0.694000 -10.515000 -0.268000
Ni -2.491000 -3.738000 0.535000
H -2.581000 -3.039000 -2.972000
H 3.963000 -1.742000 -0.590000
H -7.262000 -8.608000 0.931000
H -7.286000 -6.173000 1.295000
H 0.668000 -11.604000 -0.338000
H 2.858000 -10.330000 -0.417000

N	-0.454000	-9.901000	-0.146000
N	1.944000	-8.491000	-0.233000
N	1.915000	-5.736000	-0.125000
H	1.833000	-0.517000	-0.273000
H	-0.271000	-1.721000	0.225000
O	-2.363000	-2.683000	-2.096000
H	-1.430000	-2.431000	-2.182000
O1	-2.613000	-4.222000	2.387000
H	-1.953000	-4.732000	2.888000
Fe	-4.028000	-1.192000	1.021000
O	-3.582000	0.853000	1.501000
O	-5.989000	-0.512000	0.904000
O2	-4.278000	-1.477000	2.586000
H	-2.988000	0.980000	2.256000
H	-6.516000	-0.539000	0.095000
H	-3.230000	-1.184000	-1.475000
H	-3.577000	0.267000	-1.011000
O	-3.871000	-0.649000	-0.953000
H	-4.413000	1.294000	1.736000
H	-6.484000	-0.971000	1.598000
O1	-4.381000	-2.996000	0.487000
H	-4.768000	-2.982000	-0.402000
O1	-2.212000	-1.774000	0.999000
H	-1.968000	-1.793000	1.930000
O1	-3.440000	-2.192000	4.009000
H	-2.813000	-2.868000	3.737000
O	-5.954000	-3.407000	3.689000
H	-6.274000	-4.090000	3.107000
H	-5.022000	-3.586000	3.829000

Complex 4’
69
Scratch

C	-6.525000	-7.050000	0.119000
C	-6.356000	-8.437000	-0.128000
C	-5.107000	-8.944000	-0.380000
C	-5.472000	-6.168000	0.069000
C	-3.986000	-8.079000	-0.392000
C	-4.179000	-6.652000	-0.216000
N	-3.103000	-5.817000	-0.263000
N	-2.770000	-8.625000	-0.499000
C	-1.875000	-6.406000	-0.249000
C	-1.729000	-7.819000	-0.388000
C	0.624000	-6.193000	0.006000
C	-0.675000	-5.596000	-0.014000
N	-0.794000	-4.278000	0.170000
C	0.329000	-3.514000	0.253000
C	1.620000	-4.142000	0.233000
C	2.778000	-3.334000	0.351000
C	0.240000	-2.106000	0.379000
C	2.670000	-1.961000	0.462000
C	1.393000	-1.354000	0.477000
C	-0.410000	-8.432000	-0.323000
C	0.743000	-7.640000	-0.135000
C	2.037000	-9.502000	-0.244000

C	0.881000	-10.297000	-0.433000
Ni	-2.805000	-3.783000	0.104000
N	-0.314000	-9.764000	-0.467000
N	1.961000	-8.197000	-0.097000
N	1.736000	-5.476000	0.134000
O	-2.347000	-2.968000	-2.158000
O1	-2.650000	-4.225000	1.972000
Fe	-4.744000	-1.465000	0.218000
O	-4.825000	0.677000	0.352000
O	-6.752000	-1.323000	-0.305000
O2	-5.217000	-1.730000	1.732000
O	-4.390000	-1.076000	-1.765000
O1	-4.742000	-3.313000	-0.292000
O1	-2.887000	-1.809000	0.546000
O	-4.081000	-2.893000	3.894000
O	-1.310000	-1.061000	7.577000
O	0.864000	-1.401000	6.352000
O	-0.519000	-3.220000	6.980000
B1	-0.551000	-1.803000	6.539000
O	-1.244000	-1.543000	5.251000
H	-4.930000	-9.994000	-0.598000
H	-5.601000	-5.114000	0.282000
H	3.730000	-3.854000	0.353000
H	-2.351000	-3.454000	-2.994000
H	3.561000	-1.346000	0.525000
H	-7.220000	-9.096000	-0.077000
H	-7.517000	-6.682000	0.357000
H	0.941000	-11.380000	-0.557000
H	3.023000	-9.954000	-0.216000
H	1.328000	-0.272000	0.546000
H	-0.741000	-1.646000	0.483000
H	-1.462000	-2.575000	-2.130000
H	-1.924000	-4.745000	2.370000
H	-4.426000	1.060000	1.149000
H	-7.104000	-1.617000	-1.152000
H	-3.594000	-1.535000	-2.100000
H	-4.311000	-0.123000	-1.900000
H	-5.760000	0.941000	0.359000
H	-7.259000	-1.763000	0.391000
H	-4.964000	-3.322000	-1.238000
H	-2.810000	-1.788000	1.505000
H	-3.891000	-3.704000	3.420000
H	-3.242000	-2.658000	4.285000
H	-1.628000	-0.290000	7.102000
H	1.329000	-1.928000	7.004000
H	-1.350000	-3.318000	7.440000
H	-0.568000	-1.669000	4.580000

Complex TS_{Bi}
69
PDB File

C	-6.525000	-7.050000	0.119000
C	-6.356000	-8.437000	-0.128000
C	-5.107000	-8.944000	-0.380000
C	-5.472000	-6.168000	0.069000

C	-3.986000	-8.079000	-0.392000
C	-4.179000	-6.652000	-0.216000
N	-3.103000	-5.817000	-0.263000
N	-2.770000	-8.625000	-0.499000
C	-1.875000	-6.406000	-0.249000
C	-1.729000	-7.819000	-0.388000
C	0.624000	-6.193000	0.006000
C	-0.675000	-5.596000	-0.014000
N	-0.794000	-4.278000	0.170000
C	0.329000	-3.514000	0.253000
C	1.620000	-4.142000	0.233000
C	2.778000	-3.334000	0.351000
C	0.240000	-2.106000	0.379000
C	2.670000	-1.961000	0.462000
C	1.393000	-1.354000	0.477000
C	-0.410000	-8.432000	-0.323000
C	0.743000	-7.640000	-0.135000
C	2.037000	-9.502000	-0.244000
C	0.881000	-10.297000	-0.433000
Ni	-2.805000	-3.783000	0.104000
N	-0.314000	-9.764000	-0.467000
N	1.961000	-8.197000	-0.097000
N	1.736000	-5.476000	0.134000
O	-2.456000	-3.365000	-1.833000
O1	-2.650000	-4.225000	1.972000
Fe	-4.744000	-1.465000	0.218000
O	-4.825000	0.677000	0.352000
O	-6.752000	-1.323000	-0.305000
O2	-5.217000	-1.730000	1.732000
O	-4.390000	-1.076000	-1.765000
O1	-4.742000	-3.313000	-0.292000
O1	-2.887000	-1.809000	0.546000
O	-4.098000	-2.582000	3.127000
O	-1.327000	-0.750000	6.809000
O	0.847000	-1.090000	5.584000
O	-0.536000	-2.909000	6.213000
B1	-0.568000	-1.492000	5.771000
O	-1.261000	-1.232000	4.483000
H	-4.930000	-9.994000	-0.598000
H	-5.601000	-5.114000	0.282000
H	3.730000	-3.854000	0.353000
H	-2.556000	-3.959000	-2.590000
H	3.561000	-1.346000	0.525000
H	-7.220000	-9.096000	-0.077000
H	-7.517000	-6.682000	0.357000
H	0.941000	-11.380000	-0.557000
H	3.023000	-9.954000	-0.216000
H	1.328000	-0.272000	0.546000
H	-0.741000	-1.646000	0.483000
H	-1.554000	-3.025000	-1.930000
H	-1.924000	-4.745000	2.370000
H	-4.426000	1.060000	1.149000
H	-7.104000	-1.617000	-1.152000
H	-3.594000	-1.535000	-2.100000
H	-4.311000	-0.123000	-1.900000

H	-5.760000	0.941000	0.359000
H	-7.259000	-1.763000	0.391000
H	-4.964000	-3.322000	-1.238000
H	-2.810000	-1.788000	1.505000
H	-3.907000	-3.393000	2.653000
H	-3.259000	-2.348000	3.518000
H	-1.645000	0.021000	6.334000
H	1.312000	-1.617000	6.237000
H	-1.367000	-3.007000	6.672000
H	-0.585000	-1.358000	3.812000

Complex 5’
69
NiIII-OHH2O-2O-FeIV=O3H2O-ch1-m2-2H-BOH4-H2O.02

C	-6.035000	-7.681000	0.736000
C	-5.925000	-9.092000	0.608000
C	-4.693000	-9.686000	0.473000
C	-4.924000	-6.868000	0.714000
C	-3.522000	-8.886000	0.467000
C	-3.642000	-7.450000	0.571000
N	-2.522000	-6.680000	0.556000
N	-2.322000	-9.497000	0.374000
C	-1.343000	-7.303000	0.528000
C	-1.244000	-8.727000	0.427000
C	1.141000	-7.113000	0.644000
C	-0.147000	-6.492000	0.649000
N	-0.297000	-5.171000	0.810000
C	0.831000	-4.424000	0.951000
C	2.121000	-5.067000	0.921000
C	3.289000	-4.281000	1.075000
C	0.751000	-3.025000	1.134000
C	3.183000	-2.921000	1.249000
C	1.910000	-2.295000	1.276000
C	0.076000	-9.358000	0.410000
C	1.239000	-8.572000	0.520000
C	2.514000	-10.461000	0.430000
C	1.354000	-11.244000	0.320000
Ni	-2.346000	-4.548000	0.910000
N	0.141000	-10.702000	0.310000
N	2.464000	-9.136000	0.533000
N	2.252000	-6.403000	0.771000
O	-1.486000	-1.340000	-1.838000
O	-2.572000	-5.204000	2.855000
Fe	-4.261000	-2.233000	1.471000
O	-4.507000	-0.225000	2.246000
O	-6.326000	-2.052000	1.054000
O	-4.722000	-2.761000	3.128000
O	-3.850000	-1.148000	-0.313000
O1	-4.214000	-3.948000	0.607000
O1	-2.340000	-2.596000	1.675000
O	-3.811000	-3.255000	4.042000
O	-1.654000	-0.066000	7.663000
O	0.000000	0.385000	5.873000
O	-0.150000	-1.753000	6.934000
B1	-0.801000	-0.527000	6.622000

O1 -1.993000 -0.858000 5.331000
H -4.576000 -10.767000 0.392000
H -5.005000 -5.785000 0.824000
H 4.251000 -4.790000 1.049000
H -1.318000 -1.324000 -2.802000
H 4.080000 -2.317000 1.367000
H -6.825000 -9.705000 0.628000
H -7.019000 -7.234000 0.864000
H 1.423000 -12.330000 0.242000
H 3.497000 -10.932000 0.440000
H 1.852000 -1.217000 1.411000
H -0.235000 -2.558000 1.148000
H -0.818000 -0.726000 -1.502000
H -1.985000 -5.718000 3.429000
H -4.135000 -0.061000 3.129000
H -6.562000 -2.142000 0.115000
H -2.958000 -1.206000 -0.739000
H -3.929000 -0.202000 -0.092000
H -5.464000 -0.126000 2.396000
H -6.816000 -2.774000 1.489000
H -4.486000 -3.918000 -0.322000
H -2.285000 -2.776000 2.630000
H -3.051000 -4.518000 3.393000
H -2.544000 -1.655000 5.508000
H -2.262000 0.636000 7.346000
H 0.853000 0.494000 6.308000
H -0.666000 -2.252000 7.582000
H -1.603000 -1.004000 4.455000

Complex 4”
60
Scratch
C -6.474000 -6.508000 0.801000
C -6.459000 -7.914000 0.623000
C -5.289000 -8.560000 0.315000
C -5.344000 -5.742000 0.642000
H -5.232000 -9.632000 0.165000
H -5.355000 -4.671000 0.803000
C -4.092000 -7.818000 0.172000
C -4.130000 -6.374000 0.295000
N -2.986000 -5.667000 0.108000
N -2.947000 -8.481000 -0.043000
C -1.842000 -6.349000 -0.029000
C -1.829000 -7.771000 -0.106000
C 0.645000 -6.297000 -0.206000
C -0.597000 -5.610000 -0.054000
N -0.651000 -4.273000 0.049000
C 0.513000 -3.577000 -0.107000
C 1.747000 -4.299000 -0.285000
C 2.959000 -3.588000 -0.454000
C 0.546000 -2.163000 -0.119000
C 2.956000 -2.216000 -0.475000
C 1.744000 -1.507000 -0.306000
C -0.560000 -8.473000 -0.213000
C 0.656000 -7.757000 -0.266000

H	3.861000	-4.186000	-0.548000
C	1.802000	-9.709000	-0.449000
C	0.585000	-10.427000	-0.390000
Ni	-2.622000	-3.622000	0.311000
H	-2.484000	-3.221000	-2.633000
H	3.877000	-1.665000	-0.633000
H	-7.378000	-8.478000	0.752000
H	-7.407000	-6.033000	1.085000
H	0.577000	-11.515000	-0.447000
H	2.743000	-10.244000	-0.548000
N	-0.568000	-9.812000	-0.273000
N	1.828000	-8.398000	-0.385000
N	1.791000	-5.643000	-0.305000
H	1.765000	-0.422000	-0.319000
H	-0.370000	-1.609000	0.068000
O	-2.334000	-2.790000	-1.781000
H	-1.391000	-2.555000	-1.783000
O1	-2.649000	-4.071000	2.177000
H	-1.917000	-4.500000	2.652000
Fe	-4.123000	-1.050000	0.822000
O	-3.615000	1.031000	0.979000
O	-6.052000	-0.279000	0.727000
O2	-4.356000	-1.273000	2.404000
H	-2.972000	1.257000	1.669000
H	-6.638000	-0.386000	-0.031000
H	-3.274000	-1.154000	-1.571000
H	-3.619000	0.339000	-1.222000
O	-3.935000	-0.572000	-1.154000
H	-4.422000	1.520000	1.203000
H	-6.521000	-0.598000	1.511000
O1	-4.496000	-2.847000	0.271000
H	-4.893000	-2.833000	-0.611000
O1	-2.315000	-1.660000	0.820000
H	-2.075000	-1.704000	1.758000
O	-3.182000	-2.532000	4.629000
H	-3.950000	-3.093000	4.527000
H	-2.432000	-3.068000	4.379000

Complex TS_{H2O}
60
PDB File

C	-5.515000	-6.713000	1.774000
C	-5.537000	-8.085000	1.422000
C	-4.440000	-8.681000	0.853000
C	-4.410000	-5.927000	1.537000
C	-3.269000	-7.923000	0.618000
C	-3.273000	-6.514000	0.945000
N	-2.147000	-5.795000	0.719000
N	-2.179000	-8.535000	0.124000
C	-1.048000	-6.434000	0.309000
C	-1.074000	-7.822000	-0.012000
C	1.414000	-6.334000	-0.062000
C	0.195000	-5.698000	0.312000
N	0.163000	-4.441000	0.765000
C	1.344000	-3.797000	0.934000

C	2.564000	-4.445000	0.505000
C	3.793000	-3.757000	0.647000
C	1.407000	-2.531000	1.552000
C	3.819000	-2.515000	1.229000
C	2.626000	-1.913000	1.701000
C	0.162000	-8.458000	-0.480000
C	1.374000	-7.728000	-0.510000
C	2.465000	-9.580000	-1.261000
C	1.255000	-10.308000	-1.229000
Ni	-1.898000	-3.748000	0.848000
N	0.127000	-9.742000	-0.845000
N	2.511000	-8.311000	-0.907000
N	2.570000	-5.692000	0.003000
O	-1.713000	-3.442000	-1.314000
O1	-2.061000	-3.708000	2.719000
Fe	-3.441000	-1.209000	0.702000
O	-2.965000	0.767000	0.302000
O	-5.391000	-0.530000	0.612000
O2	-3.441000	-1.003000	2.321000
O	-3.626000	-1.319000	-1.395000
O1	-3.846000	-3.042000	0.691000
O1	-1.641000	-1.739000	0.627000
O	-1.462000	-1.172000	3.208000
H	-4.421000	-9.733000	0.589000
H	-4.364000	-4.883000	1.833000
H	4.694000	-4.258000	0.307000
H	-1.495000	-4.172000	-1.909000
H	4.763000	-1.995000	1.356000
H	-6.429000	-8.670000	1.621000
H	-6.385000	-6.278000	2.256000
H	1.221000	-11.352000	-1.532000
H	3.390000	-10.046000	-1.592000
H	2.681000	-0.952000	2.203000
H	0.503000	-2.094000	1.964000
H	-0.926000	-2.876000	-1.270000
H	-1.477000	-4.376000	3.102000
H	-2.145000	1.056000	0.723000
H	-5.944000	-0.891000	-0.093000
H	-3.004000	-1.998000	-1.710000
H	-3.373000	-0.490000	-1.824000
H	-3.660000	1.341000	0.655000
H	-5.838000	-0.733000	1.447000
H	-4.322000	-3.262000	-0.122000
H	-1.309000	-1.361000	1.472000
H	-1.924000	-0.666000	3.881000
H	-1.747000	-2.115000	3.299000

Complex 5”
60
Scratch

C	-5.766000	-7.119000	2.283000
C	-5.824000	-8.482000	1.907000
C	-4.712000	-9.114000	1.411000
C	-4.621000	-6.378000	2.110000
C	-3.500000	-8.398000	1.257000

C	-3.473000	-6.989000	1.559000
N	-2.329000	-6.290000	1.342000
N	-2.402000	-9.064000	0.862000
C	-1.230000	-6.978000	1.012000
C	-1.265000	-8.389000	0.788000
C	1.234000	-6.953000	0.660000
C	0.016000	-6.252000	0.922000
N	-0.008000	-4.927000	1.129000
C	1.175000	-4.255000	1.050000
C	2.394000	-4.995000	0.834000
C	3.629000	-4.304000	0.802000
C	1.235000	-2.849000	1.160000
C	3.657000	-2.940000	0.941000
C	2.454000	-2.214000	1.105000
C	-0.025000	-9.095000	0.473000
C	1.196000	-8.396000	0.422000
C	2.282000	-10.324000	-0.050000
C	1.060000	-11.023000	-0.003000
Ni	-1.989000	-4.212000	1.478000
N	-0.088000	-10.419000	0.256000
N	2.356000	-9.019000	0.158000
N	2.399000	-6.325000	0.645000
O	-1.951000	-3.719000	-0.700000
O1	-2.206000	-4.400000	3.554000
Fe	-3.608000	-1.594000	1.759000
O	-3.231000	0.572000	1.826000
O	-5.559000	-0.801000	1.151000
O1	-3.964000	-1.397000	3.615000
O	-3.321000	-1.180000	-0.385000
O1	-3.901000	-3.444000	1.339000
O1	-1.723000	-2.172000	1.783000
O1	-4.254000	-2.637000	4.263000
H	-4.707000	-10.168000	1.156000
H	-4.552000	-5.352000	2.457000
H	4.528000	-4.892000	0.652000
H	-2.448000	-4.336000	-1.253000
H	4.603000	-2.408000	0.912000
H	-6.748000	-9.035000	2.043000
H	-6.640000	-6.657000	2.732000
H	1.024000	-12.096000	-0.181000
H	3.214000	-10.843000	-0.262000
H	2.496000	-1.132000	1.185000
H	0.309000	-2.293000	1.265000
H	-1.044000	-3.747000	-1.032000
H	-1.502000	-4.550000	4.192000
H	-2.705000	0.933000	2.551000
H	-5.770000	-0.857000	0.211000
H	-2.800000	-1.950000	-0.678000
H	-2.759000	-0.406000	-0.519000
H	-4.060000	1.067000	1.830000
H	-6.381000	-0.914000	1.642000
H	-4.717000	-3.898000	1.108000
H	-1.310000	-2.047000	2.646000
H	-4.488000	-2.329000	5.154000
H	-2.865000	-3.796000	3.953000

Complex 6”
59
Scratch

C	-5.753000	-7.071000	2.321000
C	-5.798000	-8.454000	2.021000
C	-4.684000	-9.099000	1.546000
C	-4.616000	-6.330000	2.097000
C	-3.481000	-8.381000	1.340000
C	-3.465000	-6.960000	1.574000
N	-2.322000	-6.265000	1.332000
N	-2.380000	-9.055000	0.967000
C	-1.219000	-6.958000	1.025000
C	-1.248000	-8.376000	0.854000
C	1.243000	-6.929000	0.670000
C	0.023000	-6.228000	0.918000
N	-0.008000	-4.899000	1.100000
C	1.171000	-4.224000	1.018000
C	2.393000	-4.962000	0.801000
C	3.624000	-4.263000	0.752000
C	1.224000	-2.818000	1.128000
C	3.645000	-2.899000	0.886000
C	2.440000	-2.178000	1.063000
C	-0.005000	-9.084000	0.556000
C	1.211000	-8.379000	0.474000
C	2.306000	-10.316000	0.062000
C	1.089000	-11.021000	0.143000
Ni	-2.008000	-4.191000	1.401000
N	-0.061000	-10.415000	0.388000
N	2.372000	-9.005000	0.224000
N	2.404000	-6.294000	0.632000
O	-1.913000	-3.648000	-0.757000
O	-2.076000	-4.262000	3.505000
Fe	-3.615000	-1.590000	1.696000
O	-3.189000	0.497000	2.091000
O	-5.530000	-0.723000	1.201000
O	-4.075000	-1.808000	3.529000
O	-3.214000	-1.053000	-0.361000
O1	-3.912000	-3.408000	1.139000
O1	-1.744000	-2.162000	1.802000
O	-4.438000	-2.933000	4.022000
H	-4.673000	-10.165000	1.345000
H	-4.562000	-5.282000	2.373000
H	4.525000	-4.848000	0.599000
H	-2.495000	-4.188000	-1.308000
H	4.588000	-2.362000	0.847000
H	-6.714000	-9.007000	2.198000
H	-6.628000	-6.593000	2.750000
H	1.059000	-12.100000	0.006000
H	3.240000	-10.836000	-0.139000
H	2.480000	-1.096000	1.150000
H	0.297000	-2.268000	1.257000
H	-1.027000	-3.753000	-1.132000
H	-1.884000	-5.095000	3.951000
H	-2.507000	0.774000	2.714000

H	-5.761000	-0.638000	0.267000
H	-2.696000	-1.812000	-0.687000
H	-2.642000	-0.279000	-0.444000
H	-3.947000	1.075000	2.240000
H	-6.349000	-0.851000	1.694000
H	-4.749000	-3.852000	0.970000
H	-1.384000	-2.180000	2.700000
H	-2.911000	-3.919000	3.892000
